# Multi-locus phylogenetic analyses reveal eight novel species of *Distoseptispora* from southern China

**DOI:** 10.1128/spectrum.02468-23

**Published:** 2023-10-31

**Authors:** Ya-Fen Hu, Jing-Wen Liu, Xing-Xing Luo, Zhao-Huan Xu, Ji-Wen Xia, Xiu-Guo Zhang, Rafael F. Castañeda-Ruíz, Jian Ma

**Affiliations:** 1 College of Agronomy, Jiangxi Agricultural University, Nanchang, Jiangxi, China; 2 Shandong Provincial Key Laboratory for Biology of Vegetable Diseases and Insect Pests, College of Plant Protection, Shandong Agricultural University, Taian, Shandong, China; 3 Instituto de Investigaciones de Sanidad Vegetal, La Habana, Cuba; University of Wisconsin-Madison School of Medicine and Public Health, Madison, Wisconsin, USA

**Keywords:** asexual ascomycetes, *Distoseptisporales*, molecular phylogeny, new taxa, taxonomy

## Abstract

Plant debris are habitat favoring the growth of various microbial species. Over the course of our mycological surveys in Jiangxi and Yunnan Provinces, China, eight new *Distoseptispora* species, viz. *D. gasaensis*, *D. guanshanensis*, *D. jinghongensis*, *D. longnanensis*, *D. menghaiensis*, *D. menglunensis*, *D. nanchangensis*, and *D. yichunensis* collected on dead branches of unidentified plants, were introduced by morphological and molecular phylogenetic analyses. Multi-locus (LSU, ITS, *TEF1*, and *RPB2*) phylogenetic analyses were performed using maximum-likelihood and Bayesian inference to infer their taxonomic positions within *Distoseptispora*. Both molecular phylogenetic analyses and morphological characters supported them as eight independent taxa within *Distoseptispora*. This work improves our understanding of the diversity of *Distoseptispora* in southern China.

**IMPORTANCE:**

*Distoseptispora* as a single genus in *Distoseptisporaceae* was introduced by morphological and phylogenetic analyses. Members of this genus occur mainly as asexual morphs, forming effuse, hairy colonies on decaying wood, plant stems, bamboo culms, and fallen leaves and shafts in terrestrial and freshwater habitats. In the present study, saprobic hyphomycetes from plant debris were investigated, and eight new *Distoseptispora* species were introduced based on morphology and phylogenetic analyses of LSU, ITS, *TEF1*, and *RPB2* sequence data. This study provides important data on the species diversity, ecological environment, and geographical area of *Distoseptispora*, greatly updates the classification of *Distoseptispora*, and improves our understanding of the taxonomy of *Distoseptispora*.

## INTRODUCTION

Fungi play a critical role in many biological processes and influence ecosystems (1). So far, estimates of fungal diversity are highly uncertain, ranging from 1.5 to 12 million species, but only about 150,000 species have been named and classified, and many are still to be discovered (2 – 4). The dematiaceous *sporidesmium*-like hyphomycetes, common saprobes on decaying wood in terrestrial and freshwater habitats, are distributed on many natural substrates around the world and are an important component of natural ecosystems (5).

The genus *Distoseptispora* (*Distoseptisporaceae*, *Distoseptisporales*) is one of the *sporidesmium*-like genera introduced by Su et al. (6) based on phylogenetic support. Members of the genus occur mainly as asexual morph, and conidial characteristics, including the types (distoseptate/euseptate) and a number of conidial septation, color, shape, and size, are mainly used to identify *Distoseptispora* species (6 – 8). The generic concept of *Distoseptispora* was revised based on the morphological characters of *D. guttulata* and *D. suoloensis* by Yang et al. (9), and later more unusual species were gradually discovered, such as *D. palmarum* (10), *D. hydei* (11), and *D. appendiculata* (12), allowing the generic circumscription of *Distoseptispora* to be expanded. Subramanian (13) segregated the species with distoseptate conidia from *Sporidesmium* and introduced seven new genera, namely, *Acarocybellina*, *Ellisembia*, *Gangliophora*, *Hemicorynesporella*, *Penzigomyces*, *Repetophragma,* and *Stanjehughesia*, to accommodate some species based on conidial septation (euseptate/distoseptate) and conidiogenesis. However, considering the non-taxonomic value of euseptate and distoseptate conidia in *sporidesmium*-like species, Su et al. (6) proposed *Ellisembia* as synonymous with *Sporidesmium*.

*Distoseptispora* is known as a saprobic fungal genus from woody hosts in aquatic and terrestrial habitats (11). *Distoseptispora* currently has 65 valid species, 44 of which are freshwater species and 21 are terrestrial species (14 – 17). Most *Distoseptispora* species are distributed in Asia, mainly in China (42 species) and Thailand (23 species) (4 – 7, 10, 14 – 32), while only a little published information is recorded in other regions (14, 15, 18, 33, 34). Only two species, viz. *D. hyalina* and *D. licualae* are reported as sexual morphs based on multi-locus analysis (5, 32). *Distoseptispora* species are not restricted to any particular host and are recorded in a variety of different plants, being the only genus in the *Distoseptisporaceae*, *Distoseptisporales*, *Diaporthomycetidae,* and *Sordariomycetes* (18).

With its complex geography, warm and humid climate, abundant light and rainfall, and numerous forested nature reserves, the southern regions of China have accumulated a rich resource of plant debris and countless taxa of dark sporulating fungi over the years. In a taxonomy and diversity survey of terrestrial fungi in Jiangxi and Yunnan Provinces, we collected 57 *sporidesmium*-like taxa. Based on multi-locus phylogenetic analyses and morphological evidence, eight *Distoseptispora* species were introduced as new to science in the present study.

## RESULTS

### Molecular phylogeny

**Fig 1:**
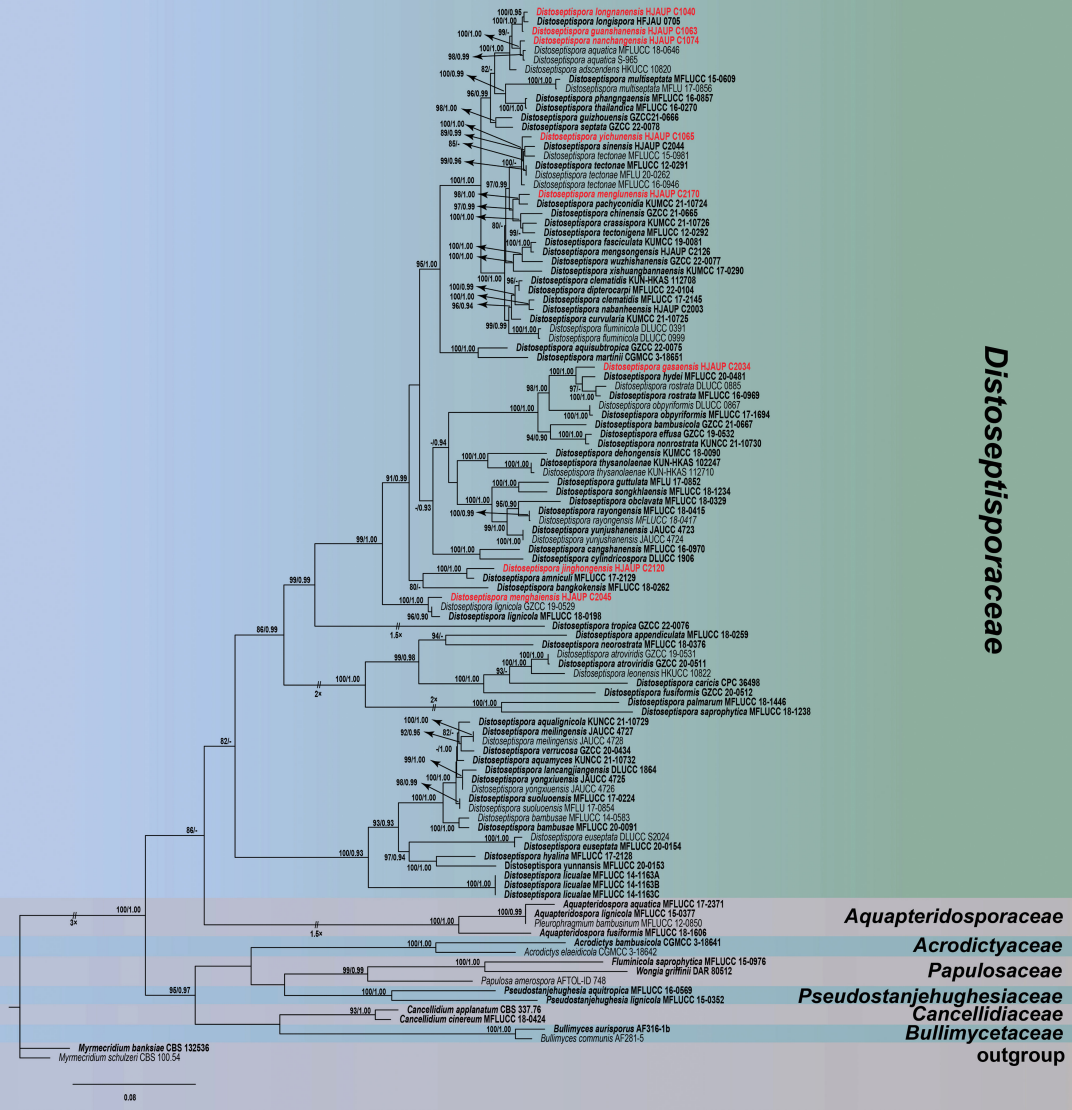
Maximum-likelihood majority rule consensus tree for *Distoseptisporaceae* and related families using LSU, ITS, *TEF1,* and *RPB2* sequence data. Bootstrap support values for maximum-likelihood greater than 70% and Bayesian posterior probabilities greater than 0.90 are shown near the nodes. The tree is rooted with *Myrmecridium schulzeri* (CBS 100.54) and *M. banksiae* (CBS 132536). The ex-type strains are in bold and the new isolates of this study are in red. Some branches were shortened according to the indicated multipliers.

Based on the combined data of LSU, ITS, *TEF1*, and *RPB2*, the phylogenetic relationships of eight *Distoseptispora* species were analyzed using regions of four genes of 110 strains representing 87 species in *Distoseptisporaceae* and related families (*Acrodictyaceae*, *Aquapteridosporaceae*, *Bullimycetaceae*, *Cancellidiaceae*, *Papulosaceae*, and *Pseudostanjehughesiaceae*). The combined data set (LSU:1–547, *TEF1*:548–1021, ITS:1022–1709, *RPB2*:1710–2506) was composed of 1,214 distinct patterns, 969 parsimony informative, 191 singleton sites, and 1,346 constant sites. A total of four single-locus data sets, ITS, LSU, *TEF1*, and *RPB2*, contained 298, 111, 231, and 329 parsimony informative sites, respectively. *Myrmecridium banksiae* (CBS 132536) and *M. schulzeri* (CBS 100.54) served as outgroup taxa. Maximum-likelihood (ML) and Bayesian inference (BI) analyses of the combined data sets resulted in phylogenetic reconstructions with largely similar topologies. The best-scoring ML consensus tree (lnL = −29062.260) with ultrafast bootstrap values from ML analyses and posterior probabilities from MrBayes analysis at the nodes are shown in Fig. 1. Our eight strains nested within the genus *Distoseptispora* representing eight species. *Distoseptispora longnanensis* (HJAUP C1040) clustered as a sister taxon to *D. longispora* (HFJAU 0705) with strong statistical support (MLBS/BPP = 100%/0.95). *Distoseptispora guanshanensis* (HJAUP C1063) formed a distinct clade sister to the clade containing *D. longispora* (HFJAU 0705) and *D. longnanensis* (HJAUP C1040) with strong statistical support (MLBS/BPP = 100%/1.00). *Distoseptispora nanchangensis* (HJAUP C1074) formed a high-support clade (MLBS/BPP = 100%/1.00) with the lineage consisting of two different strains of *D. aquatica* (MFLUCC 18–0646 and S-965). *Distoseptispora yichunensis* (HJAUP C1065) formed a distinct clade sister to the clade containing *D. tectonae* (MFLUCC 15–0981, MFLU 20–0262, MFLUCC 12–0291, and MFLUCC 16–0946) and *D. sinensis* (HJAUP C2044) with strong statistical support (MLBS/BPP = 100%/1.00). The strains of *Distoseptispora menglunensis* (HJAUP C2170) formed a distinct clade sister to *D. pachyconidia* (KUMCC 21–10724) with good statistical support (MLBS/BPP = 98%/1.00). *Distoseptispora gasaensis* (HJAUP C2034) clustered as a sister taxon to the clade containing *D. hydei* (MFLUCC 20–0481) and *D. rostrara* (DLUCC 0885 and MFLUCC 16–0969) with strong bootstrap support (MLBS/BPP = 100%/1.00). *Distoseptispora jinghongensis* (HJAUP C2120) formed a distinct clade sister to *D. amniculi* (MFLUCC 17–2129) with strong statistical support (MLBS/BPP = 100%/1.00). *Distoseptispora menghaiensis* (HJAUP C2045) was a sister to two different strains of *D. lignicola* (GZCC 19–0529 and MFLUCC 18–0198) with strong statistical support (MLBS/BPP = 100%/1.00).

### Taxonomy

**Fig 2:**
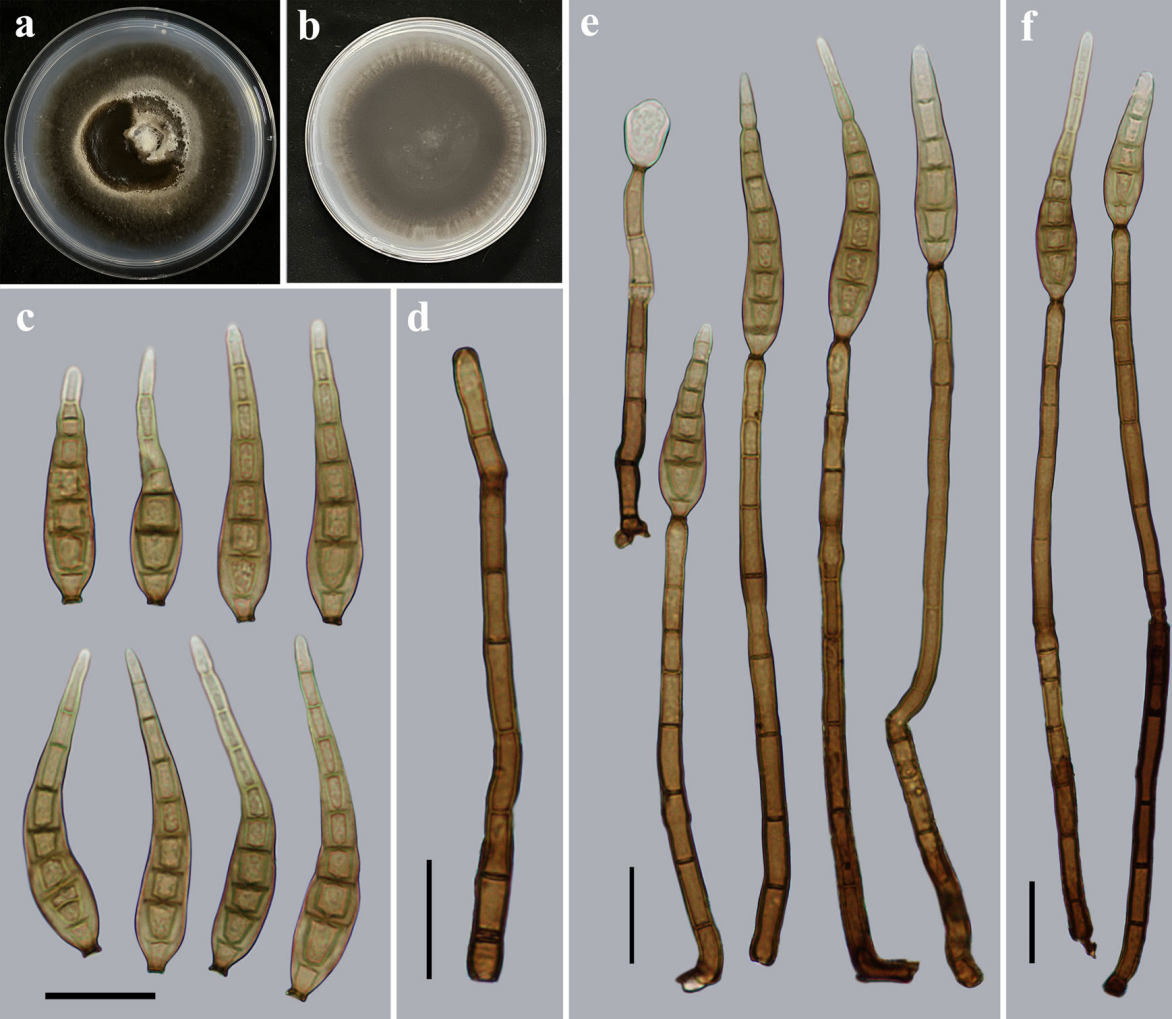
Morphology of *Distoseptispora gasaensis* (from holotype HJAUP M2034). (a) The surface of the colony after 4 weeks on potato dextrose agar (PDA); (b) reverse of the colony after 4 weeks on PDA; (c) conidia; (d) conidiophore; and (**e and f**) conidiophores, conidiogenous cells, and conidia. Scale bars (c to f), 20 µm.

*Distoseptispora gasaensis* Y.F. Hu & Jian Ma, sp. nov. (Fig. 2).

MycoBank number: MB 849128

Etymology: In reference to the locality, Gasa Township, where the fungus was collected.

Typus: China, Yunnan Province, Xishuangbanna Dai Autonomous Prefecture, Jinghong City, Gasa Township, Nabanhe National Nature Reserve, 21°57′10.02″N, 100°45′42.65″E, on dead branches of an unidentified broadleaf tree, 12 July 2021, Y.F. Hu (holotype HJAUP M2034; ex-type living culture HJAUP C2034).

Description: Saprobic on dead branches in terrestrial habitats. Sexual morph: Undetermined. Asexual morph: Hyphomycetes. Colonies on natural substrate effuse, dark brown to black, hairy, velvety. Mycelium is superficial and immersed, composed of branched, septate, smooth, subhyaline to pale brown hyphae. Conidiophores 104–204 × 4–7.2 µm (*x̅* =153.5 × 4.8 µm, SD = 31 × 1, *n* = 20), macronematous, mononematous, cylindrical, erect, unbranched, straight or slightly flexuous, single or in groups of two or three, thick-walled, smooth, 6–12-septate, brown to dark brown, and determinate or sometimes with cylindrical, enteroblastic percurrent extensions. Conidiogenous cells are monoblastic, integrated, terminal, cylindrical, pale brown or brown, and smooth. Conidia 44–72 × 6–12 µm (*x̅* =64.1 × 8.9 µm, SD = 9 × 1, *n* = 30), acrogenous, solitary, obclavate or obpyriform, straight or slightly curved, smooth, 5–9-distoseptate, brown to pale brown, rostrate, tapering and paler toward the rounded apex, and truncate at the base.

Culture characteristics: Colony on potato dextrose agar (PDA) reaching 81–86 mm diameter after 4 weeks in an incubator under dark conditions at 25°C, circular, with dense, gray mycelium in the middle, darker part of the inner ring, with sparser, white mycelium of the outer ring on the surface; reverse dark brown to black.

Notes: The phylogenetic tree showed that the strain of *D. gasaensis* (HJAUP C2034) forms an independent clade and clusters with the strains of *D. hydei* (MFLUCC 20–0481) and *D. rostrata* (MFLUCC 16–0969 and DLUCC 0885). BLASTn analysis of *D. gasaensis* (HJAUP C2034) and *D. hydei* (MFLUCC 20–0481) showed 98% identity (387/393, one gap) using ITS, 99% identity (533/536, one gap) using LSU and 90% identity (852/942, seven gaps) using *TEF1*; of *D. gasaensis* (HJAUP C2034) and *D. rostrata* (MFLUCC 16–0969) showed 97% identity (495/511, two gaps) using ITS, 99% identity (530/536, one gap) using LSU. Moreover, *D. gasaensis* is significantly different from *D. hydei* (11), which has shorter conidiophores (87–145 µm vs 104–204 µm), and shorter and wider conidia (32–58 × 10–15 µm vs 44–72 × 6–12 µm), and from *D. rostrata* (7), which has shorter conidiophores (82–126 µm vs 104–204 µm) and longer conidia (115–155 µm vs 44–72 µm) with (15–)18–23 distosepta, as well as from *D. lignicola* (12), which has shorter conidiophores (84–124 µm vs 104–204 µm), and longer and narrower conidia (60–108 × 7–9 µm vs 44–72 × 6–12 µm) with 5–9 eusepta.

**Fig 3:**
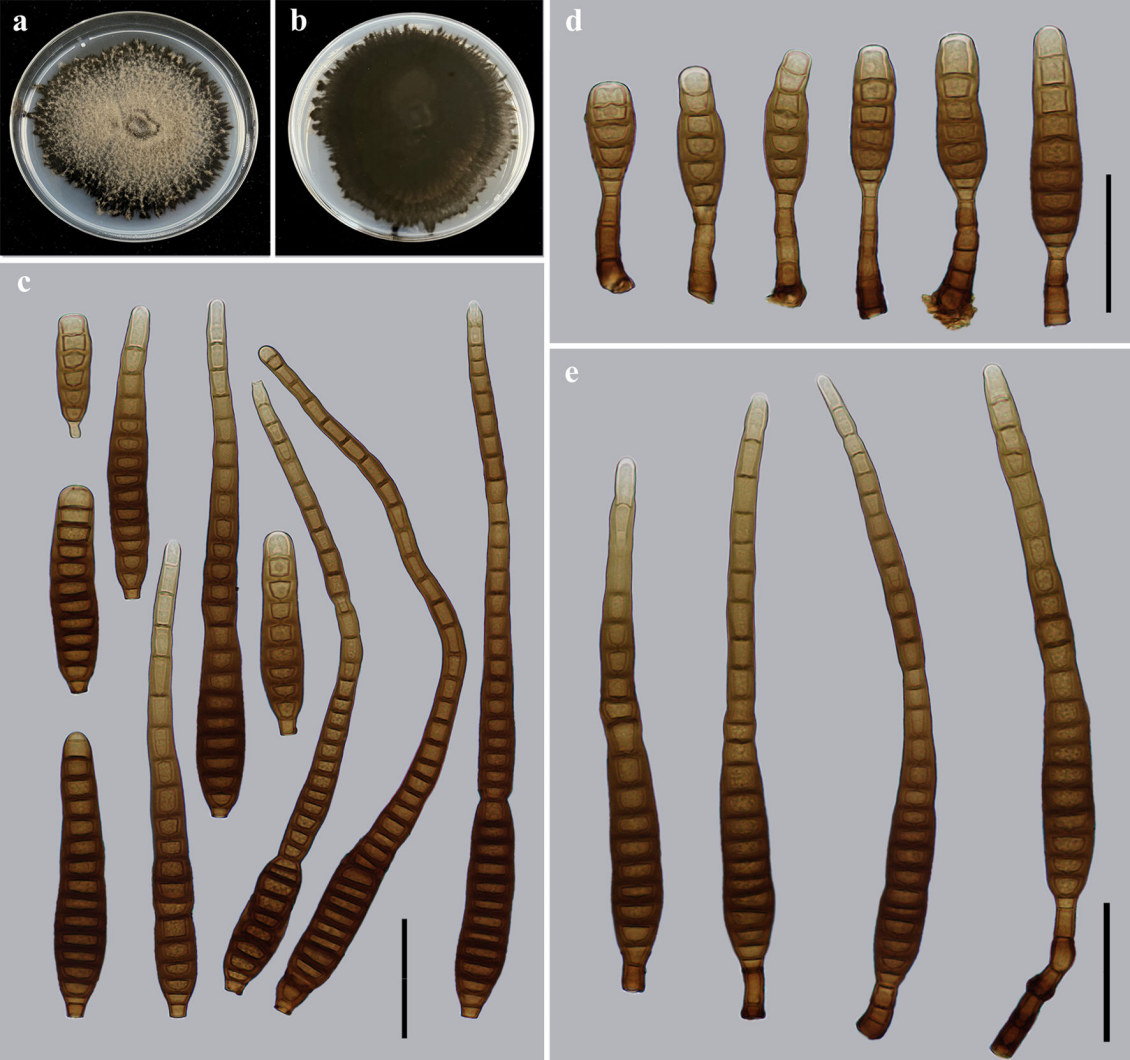
Morphology of *Distoseptispora guanshanensis* (from holotype HJAUP M1063). (**a**) The surface of the colony after 4 weeks on PDA; (**b**) reverse of the colony after 4 weeks on PDA; (**c**) conidia; and (**d and e**) conidiophores, conidiogenous cells, and conidia. Scale bars (c to e), 40 µm.

*Distoseptispora guanshanensis* Y.F. Hu & Jian Ma, sp. nov. (Fig. 3).

MycoBank number: MB849129

Etymology: The name refers to Guanshan Nature Reserve, where the fungus was collected.

Typus: China, Jiangxi Province, Yichun City, Guanshan National Nature Reserve, 28°32′21.67″N, 114°33′52.38″E, on dead branches of an unidentified broadleaf tree, 25 June 2021, Y.F. Hu (holotype HJAUP M1063; ex-type living culture HJAUP C1063).

Description: Saprobic on dead branches in terrestrial habitats. Sexual morph: Undetermined. Asexual morph: Hyphomycetes. Colonies on natural substrate effuse, scattered, dark brown to black, hairy. Mycelium is superficial and immersed, composed of branched, septate, smooth, pale brown to brown hyphae. Conidiophores 15.4–44.7 × 5.2–8.3 µm (*x̅* =30.3 × 7.1 µm, SD = 10 × 0.8, *n* = 20), macronematous, mononematous, straight or slightly flexuous, solitary, cylindrical, unbranched, smooth, brown to dark brown, 1–6-septate, robust at the base, and truncate at the apex. Conidiogenous cells are monoblastic, integrated, terminal, determinate, cylindrical, pale brown, and smooth. Conidia 96.5–255.3 × 12.3–16.5 µm (*x̅* =166.1 × 13.5 µm, SD = 52 × 1, *n* = 30), acrogenous, solitary, obclavate, straight or slightly curved, (5–)11–38-distoseptate, slightly constricted at the septa, pale brown to dark brown, smooth-walled, tapering toward the rounded apex, and truncate at the base.

Culture characteristics: Colony on PDA reaching 80–85 mm diameter after 4 weeks in an incubator under dark conditions at 25°C, irregularly circular, surface velvety, with gray-white and denser mycelium at the center, becoming black-brown and sparser toward the edge; reverse dark brown to black.

Notes: The phylogenetic tree showed that the strain of *D. guanshanensis* (HJAUP C1063) forms an independent clade and clusters with the strains of *D. longispora* (HFJAU 0705) and *D. longnanensis* (HJAUP C1040). BLASTn analysis of *D. guanshanensis* (HJAUP C1063) and *D. longispora* (HFJAU 0705) showed 99% identity (501/503, one gap) using ITS, and 99% identity (546/547, no gaps) using LSU; of *D. guanshanensis* (HJAUP C1063) and *D. longnanensis* (HJAUP C1040) showed 99% identity (596/603, five gaps) using ITS, and 99% identity (546/547, one gap) using LSU. Moreover, *D. guanshanensis* is significantly different from *D. longnanensis* in its shorter conidiophores (15.4–44.7 µm vs 77–171 µm) and bigger conidia (96.5–255.3 × 12.3–16.5 µm vs 54–87 × 8.2–14 µm) with more distosepta [(5–)11–38 vs 4–8], and from *D. longispora* (24) in its longer and narrower conidiophores (15.4–44.7 × 5.2–8.3 µm vs 17–37 × 6–10 µm) and smaller conidia (96.5–255.3 × 12.3–16.5 µm vs 189–297 × 16–23 µm) with fewer distosepta [(5–)11–38 vs 31–56]. In addition*, D. guanshanensis* morphologically differs from *D. tectonigena* (35) by its smaller conidiophores (15.4–44.7 × 5.2–8.3 µm vs up to 110 × 5–11 µm), and wider conidia [12.3–16.5 µm vs (10–)11–12(–13) µm] with fewer distosepta [(5–)11–38 vs 20–46].

**Fig 4:**
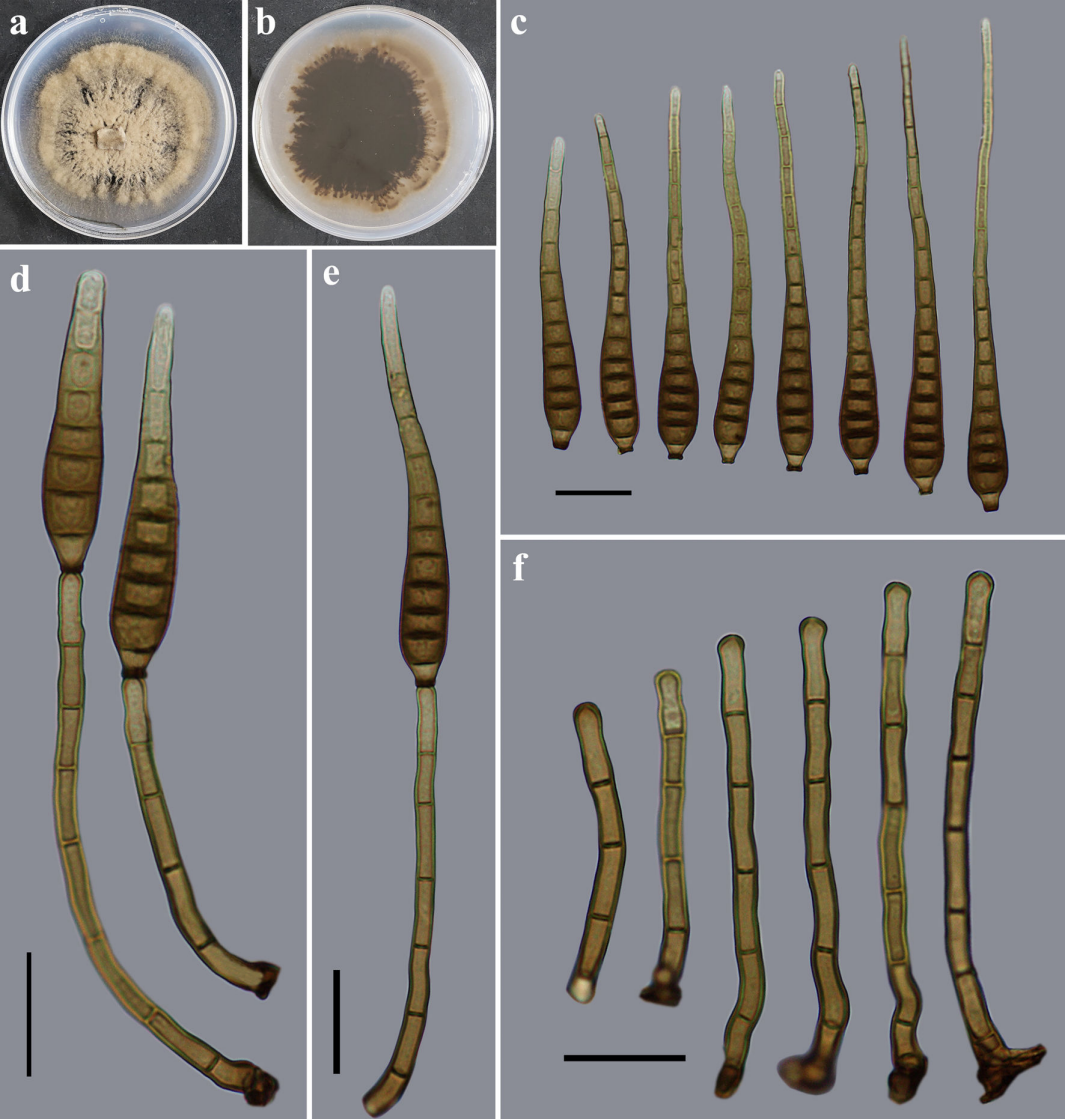
Morphology of *Distoseptispora jinghongensis* (from holotype HJAUP M2120). (**a**) The surface of the colony after 4 weeks on PDA; (**b**) reverse of the colony after 4 weeks on PDA; (**c**) conidia; (**d and e**) conidiophores, conidiogenous cells, and conidia; and (**f**) conidiophores and conidiogenous cells. Scale bars (c to f), 20 µm.

*Distoseptispora jinghongensis* Y.F. Hu & Jian Ma, sp. nov. (Fig. 4).

MycoBank number: MB849130

Etymology: In reference to the locality, Jinghong City, where the fungus was collected.

Typus: China, Yunnan Province, Xishuangbanna Dai Autonomous Prefecture, Jinghong City, Xishuangbanna Dai Nationality Garden, 21°50′53.85″N, 100°56′48.05″E, on dead branches of an unidentified broadleaf tree, 14 July 2021, Y.F. Hu (holotype HJAUP M2120; ex-type living culture HJAUP C2120).

Description: Saprobic on dead branches in terrestrial habitats. Sexual morph: Undetermined. Asexual morph: Hyphomycetes. Colonies on natural substrate effuse, pale brown or brown, and hairy or velvety. Mycelium is superficial and immersed, composed of branched, septate, smooth, and pale brown to brown hyphae. Conidiophores 54.6–94.6 × 3.6–4 µm (*x̅* =70.5 × 3.7 µm, SD = 16 × 0.1, *n* = 20), macronematous, mononematous, mid-olivaceous to brown, single or in groups of two or three, 4–7-septate, erect, cylindrical, straight or flexuous, unbranched, and smooth. Conidiogenous cells are monoblastic, integrated, terminal, determinate, pale brown, and cylindrical. Conidia 56.4–127.3 × 7.3–10.9 µm (*x̅* =90.8 × 9.8 µm, SD = 22 × 1, *n* = 30), acrogenous, solitary, obclavate, straight or slightly curved, smooth, 7–17-distoseptate, rostrate, pale brown or brown, tapering and paler toward the rounded apex, and truncate at the base.

Culture characteristics: Colony on PDA reaching 75–81 mm diameter after 4 weeks in an incubator under dark conditions at 25°C, irregularly circular, with fluffy, dense, thin brown mycelium on the surface, becoming sparse and pale brown at the entire margin; reverse dark brown, pale brown at the margin.

Notes: The phylogenetic tree showed that *D. jinghongensis* (HJAUP C2120) clusters with *D. amniculi* (MFLUCC17-2129), and they form a sister clade to *D. bangkokensis* (MFLUCC 18–0262). BLASTn analysis of *D. jinghongensis* (HJAUP C2120) and *D. amniculi* (MFLUCC17-2129) showed 97% identity (536/550, three gaps) using ITS, and 99% identity (547/548, no gap) using LSU. Moreover, *D. jinghongensis* differs from *D. amniculi* (5) by its shorter conidiophores (54.6–94.6 µm vs 90–180 µm) and smaller conidia (56.4–127.3 × 7.3–10.9 µm vs 85–167 × 9–11.8 µm) with fewer distosepta [7–17 vs (7–)12–24]. *Distoseptispora jinghongensis* is also morphologically similar to *D. bambusicola* (28), but the latter has bigger conidiophores (64–116 × 4–7 µm vs 54.6–94.6 × 3.6–4 µm), and yellowish-brown, longer conidia (72–193 µm vs 56.4–127.3 µm).

**Fig 5:**
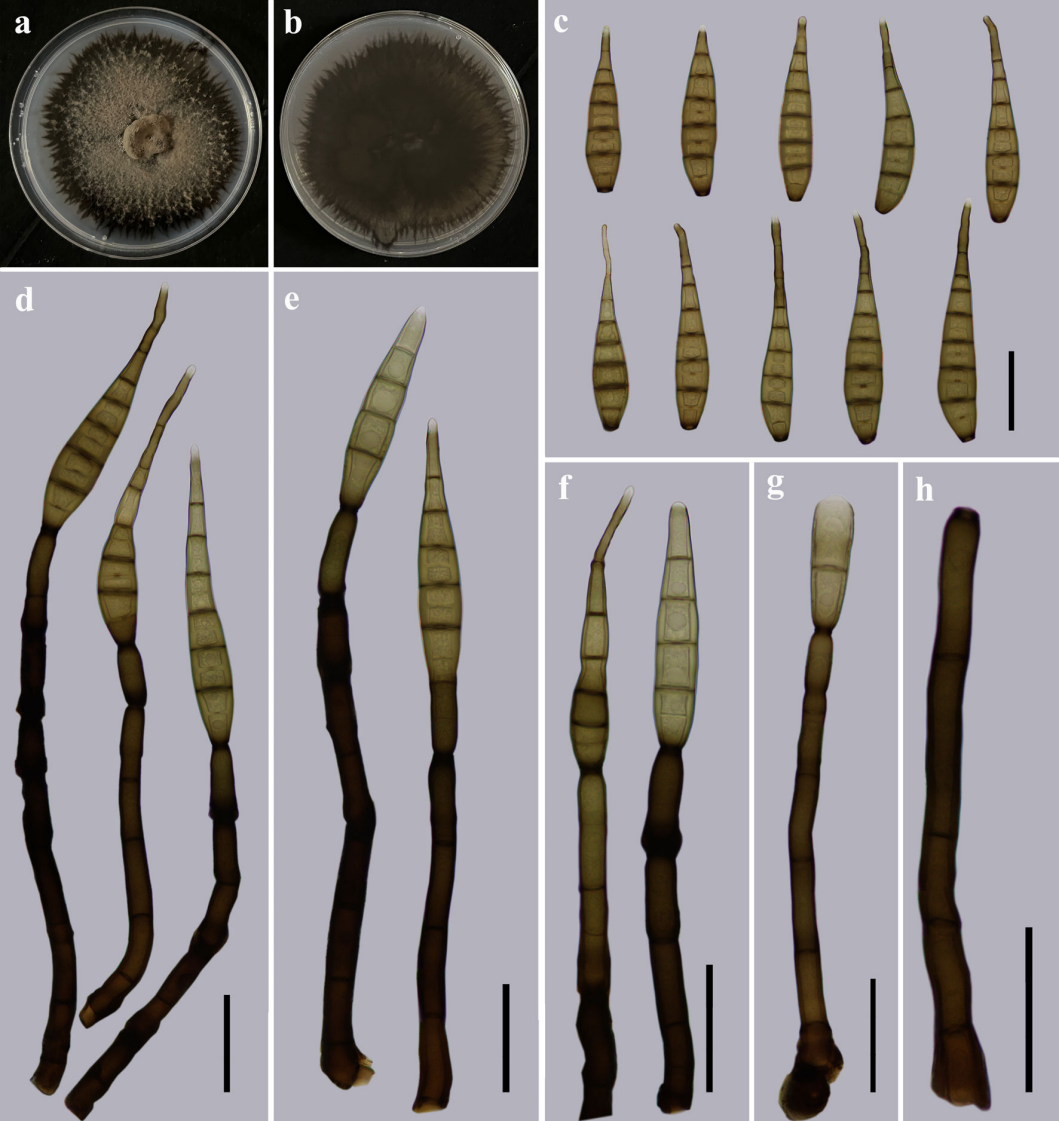
Morphology of *Distoseptispora longnanensis* (from holotype HJAUP M1040). (**a**) The surface of the colony after 4 weeks on PDA; (**b**) reverse of the colony after 4 weeks on PDA; (**c**) conidia; (d to g) conidiophores, conidiogenous cells, and conidia; and (**h**) conidiophore. Scale bars (c to h), 30 µm.

*Distoseptispora longnanensis* Y.F. Hu & Jian Ma, sp. nov. (Fig. 5).

MycoBank number: MB849131

Etymology: In reference to the locality, Longnan County, where the fungus was collected.

Typus: China, Jiangxi Province, Longnan County, Jiulianshan National Nature Reserve, 24°37′29.51″N, 114°34′4.76″E, on dead branches of an unidentified broadleaf tree, 20 June 2021, Y.F. Hu (holotype HJAUP M1040; ex-type living culture HJAUP C1040).

Description: Saprobic on dead branches in terrestrial habitats. Sexual morph: Undetermined. Asexual morph: Hyphomycetes. Colonies on natural substrate effuse, scattered, hairy, and dark brown to black. Mycelium is superficial and immersed, composed of septate, branched, smooth, and pale brown to brown hyphae. Conidiophores 77–171 × 4–8 µm (*x̅* =118.8 × 6.5 µm, SD = 33 × 1, *n* = 20), macronematous, mononematous, solitary or aggregated at the base, cylindrical, straight or slightly flexuous, 4–11-septate, olivaceous to dark brown, paler at the apical part, determinate or with several cylindrical, and enteroblastic percurrent extensions. Conidiogenous cells are monoblastic, integrated, terminal, cylindrical, and olivaceous to dark brown. Conidia 54–87 × 8.2–14 µm (*x̅* =71.5 × 11.8 µm, SD = 10 × 1, *n* = 30), acrogenous, solitary, obclavate, straight or flexuous, tapering toward the rounded apex, 4–8-septate, olivaceous to yellowish-brown or brown, becoming paler toward the apex, with a darkened scar at the truncate base.

Culture characteristics: Colony on PDA reaching 80–85 mm diameter after 4 weeks in an incubator under dark conditions at 25°C, irregularly circular, surface velvety, with gray-white and denser mycelium at the center, becoming black-brown and sparser toward the edge; reverse pale brown to black.

Notes: The phylogenetic tree showed that *D. longnanensis* (HJAUP C1040) clusters with *D. longispora* (HFJAU 0705). The BLASTn analysis of *D. longnanensis* (HJAUP C1040) and *D. longispora* (HFJAU 0705) showed 99% identity (551/555, three gaps) using LSU, and 100% identity (503/503, no gap) using ITS. Moreover, *D. longnanensis* morphologically differs from *D. longispora* (24) by its longer and narrower conidiophores (77–171 × 4–8 µm vs 17–37 × 6–10 µm), and smaller conidia (54–87 × 8.2–14 µm vs 189–297 × 16–23 µm) with fewer distosepta (4–8 vs 31–56). *D. longnanensis* is also morphologically similar to *D. verrucosa* (5), but the latter lives in freshwater habitats and has longer conidiophores (92–250 µm vs 77–171 µm), and smaller conidia (41–63 × 8.8–12.6 µm vs 54–87 × 8.2–14 µm).

**Fig 6:**
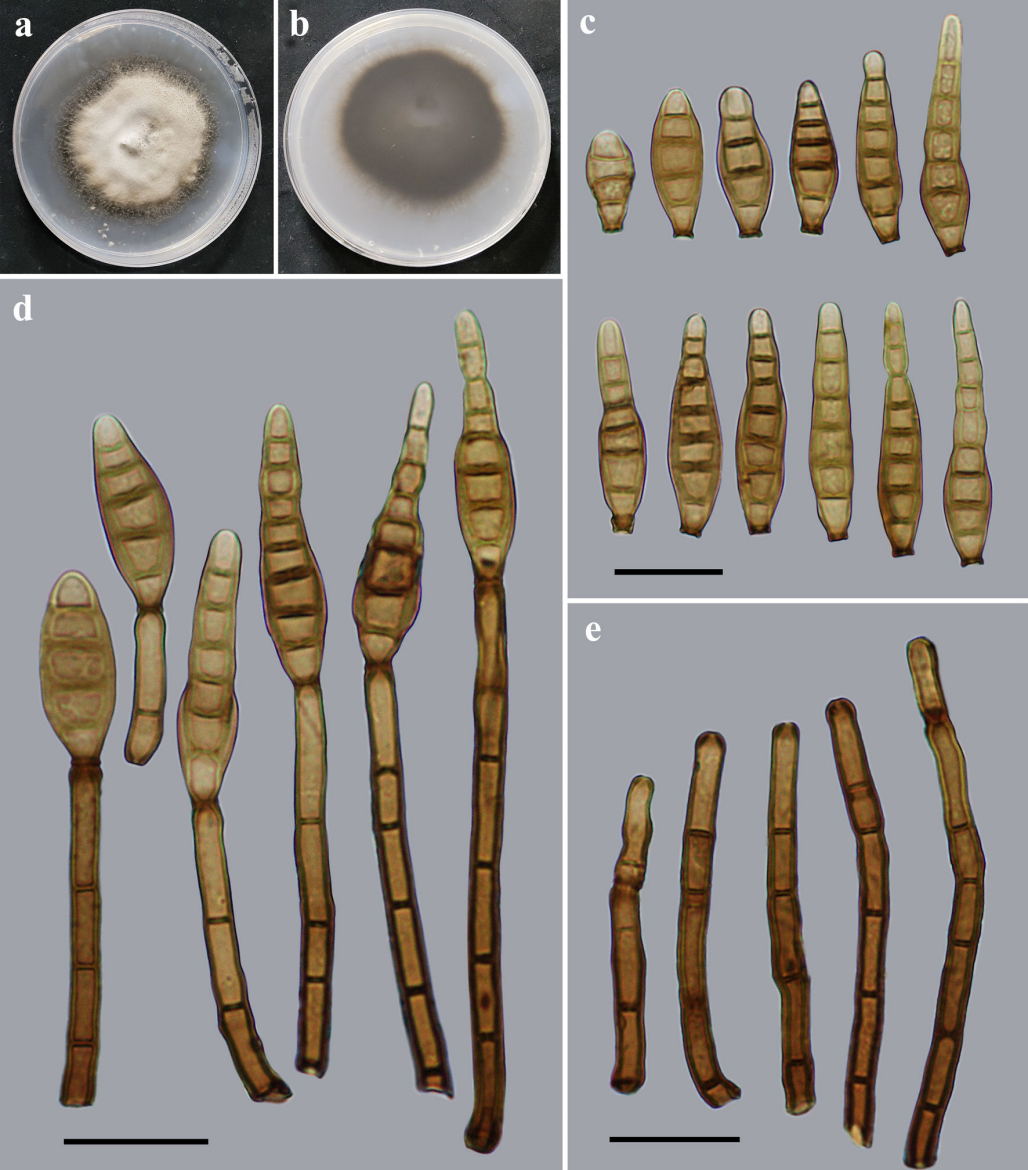
Morphology of *Distoseptispora menghaiensis* (from holotype HJAUP 2045). (**a**) The surface of the colony after 4 weeks on PDA; (**b**) reverse of the colony after 4 weeks on PDA; (**c**) conidia; (**d**) conidiophores, conidiogenous cells, and conidia; and (**e**) conidiophores and conidiogenous cells. Scale bars (c to e), 20 µm.

*Distoseptispora menghaiensis* Y.F. Hu & Jian Ma, sp. nov. (Fig. 6).

MycoBank number: MB849132

Etymology: In reference to the locality, Menghai County, where the fungus was collected.

Typus: China, Yunnan Province, Xishuangbanna Dai Autonomous Prefecture, Menghai County, Mengsong Township, Nabanhe National Nature Reserve, 21°30′11.51″N, 100°30′3.68″E, on dead branches of an unidentified broadleaf tree, 13 July 2021, Y.F. Hu (holotype HJAUP M2045; ex-type living culture HJAUP C2045).

Description: Saprobic on dead branches in terrestrial habitats. Sexual morph: Undetermined. Asexual morph: Hyphomycetes. Colonies on natural substrate effuse, scattered, hairy, and dark brown. Mycelium is superficial and immersed, composed of branched, septate, smooth-walled, and pale brown to brown hyphae. Conidiophores 45.7–82.9 × 3.4–5.1 µm (*x̅* =58.3 × 4.26 µm, SD = 11 × 0.5, *n* = 20), macronematous, mononematous, unbranched, single or in groups of two or three, cylindrical, 2–6-septate, straight or slightly flexuous, smooth, brown, determinate or sometimes with cylindrical, enteroblastic percurrent extensions. Conidiogenous cells are monoblastic, integrated, terminal, cylindrical, brown, and smooth. Conidia 35.7–48.6 × 7.2–10.9 µm (*x̅* =41.6 × 8.7 µm, SD = 3 × 0.7, *n* = 30), acrogenous, solitary, obclavate, straight or curved, smooth-walled, 4–8-distoseptate, pale brown to brown, tapering toward the rounded apex, and truncate at the base.

Culture characteristics: Colony on PDA reaching 63–69 mm diameter after 4 weeks in an incubator under dark conditions at 25°C, circular, with dense, gray mycelium in the center, darker in the outer ring, with sparser, gray-white mycelium on the surface; reverse dark brown to black.

Notes: The phylogenetic tree showed that the strain of *D. menghaiensis* (HJAUP C2045) clusters with two different strains of *D. lignicola* (MFLUCC 18–0198^T^ and GZCC 19–0529). BLASTn analysis of *D. menghaiensis* (HJAUP C2045) and *D. lignicola* (MFLUCC 18–0198^T^) showed 98% identity (517/529, three gaps) using ITS, and 99% identity (540/541, no gap) using LSU. Moreover, *D. menghaiensis* differs from *D. lignicola* (12) which has longer conidiophores (84–124 µm vs 45.7–82.9 µm) and solitary or catenate, larger conidia (60–108 × 7–9 µm vs 35.7–48.6 × 7.2–10.9 µm) with 5–9 eusepta. *Distoseptispora menghaiensis* also superficially resembles *D. obpyriformis* (7), but the latter has bigger conidiophores (97–119 × 5–7 µm vs 45.7–82.9 × 3.4–5.1 µm), and bigger conidia (53–71 × 12–16 µm vs 35.7–48.6 × 7.2–10.9 µm) with more distosepta (9–11 vs. 4–8).

**Fig 7:**
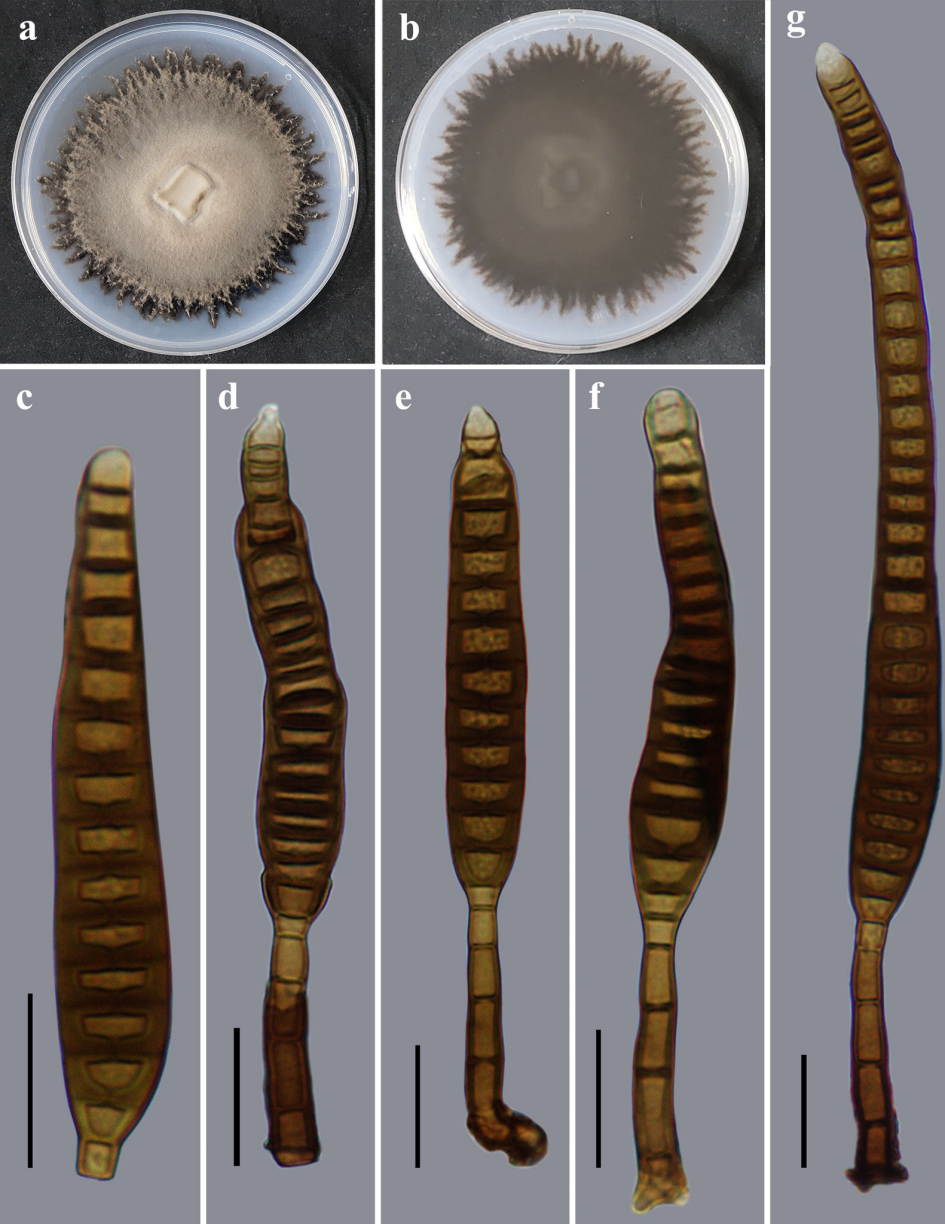
Morphology of *Distoseptispora menglunensis* (from holotype HJAUP M2170). (**a**) The surface of the colony after 4 weeks on PDA; (**b**) reverse of the colony after 4 weeks on PDA; (**c**) conidia; and (d to g) conidiophores, conidiogenous cells, and conidia. Scale bars (c to g), 20 µm.

*Distoseptispora menglunensis* Y.F. Hu & Jian Ma, sp. nov. (Fig. 7).

MycoBank number: MB849133

Etymology: In reference to the locality, Menglun Township, where the fungus was collected.

Typus: China, Yunnan Province, Xishuangbanna Dai Autonomous Prefecture, Mengla County, Menglun Township, Xishuangbanna Tropical Botanical Garden, 21°52′21.55″N, 101°19′22.20″E, on dead branches of an unidentified broadleaf tree, 15 July 2021, Y.F. Hu (holotype HJAUP M2170; ex-type living culture HJAUP C2170).

Description: Saprobic on dead branches in terrestrial habitats. Sexual morph: Undetermined. Asexual morph: Hyphomycetes. Colonies on natural substrate and effuse, scattered, hairy, and pale brown to brown. Mycelium is superficial and immersed, composed of branched, septate, smooth, and pale brown to brown hyphae. Conidiophores 35–52.5 × 6.3–7.5 µm (*x̅* =44.4 × 6.9 µm, SD = 7 × 0.5, *n* = 20), macronematous, mononematous, unbranched, cylindrical, solitary, 3–4-septate, straight or flexuous, dark brown to brown, smooth, determinate or sometimes with a cylindrical, enteroblastic percurrent extension. Conidiogenous cells are monoblastic, integrated, terminal, cylindrical, pale brown to brown, and smooth. Conidia 82.5–172.2 × 12.5–15 µm (*x̅* =103.5 × 14 µm, SD = 38 × 1, *n* = 30), acrogenous, solitary, obclavate, straight or slightly curved, brown to dark brown, smooth, 14–33-distoseptate, truncate at the base, rounded at the apex.

Culture characteristics: Colony on PDA reaching 76–85 mm diameter after 4 weeks in an incubator under dark conditions at 25°C, circular, surface velvety, dense, gray-brown mycelium with dark brown margin, reverse dark brown to black.

Notes: The phylogenetic tree showed that the strain of *D. menglunensis* (HJAUP C2170) clusters with *D. pachyconidia* (KUMCC 21–10724), and they form a sister clade to *D. chinensis* (GZCC21-0665), *D. crassispora* (KUMCC 21–10726), and *D. tectonigena* (MFLUCC 12–0292). BLASTn analysis of *D. menglunensis* (HJAUP C2170) and *D. pachyconidia* (KUMCC 21–10724) showed 98% identity (489/500, two gaps) using ITS, 99% identity (559/564, three gaps) using LSU, 98% identity (903/918, 0 gap) using *TEF1* and 99% identity (829/832, no gap) using *RPB2*. Moreover, *D. menglunensis* is significantly different from *D. pachyconidia* (18) in its longer conidiophores (35–52.5 µm vs 11–27 µm) and longer and narrower conidia (82.5–172.2 × 12.5–15 µm vs 42–136 × 14–22 µm) with more distosepta (14–33 vs 8–21). In addition, *D. menglunensis* morphologically differs from *D. multiseptata* (35), which lives in freshwater habitats and has bigger conidiophores (23–65 × 4.5–8.5 µm vs 35–52.5 × 6.3–7.5 µm), and bigger conidia (95–290 × 11–20 µm vs 82.5–172.2 × 12.5–15 µm).

**Fig 8:**
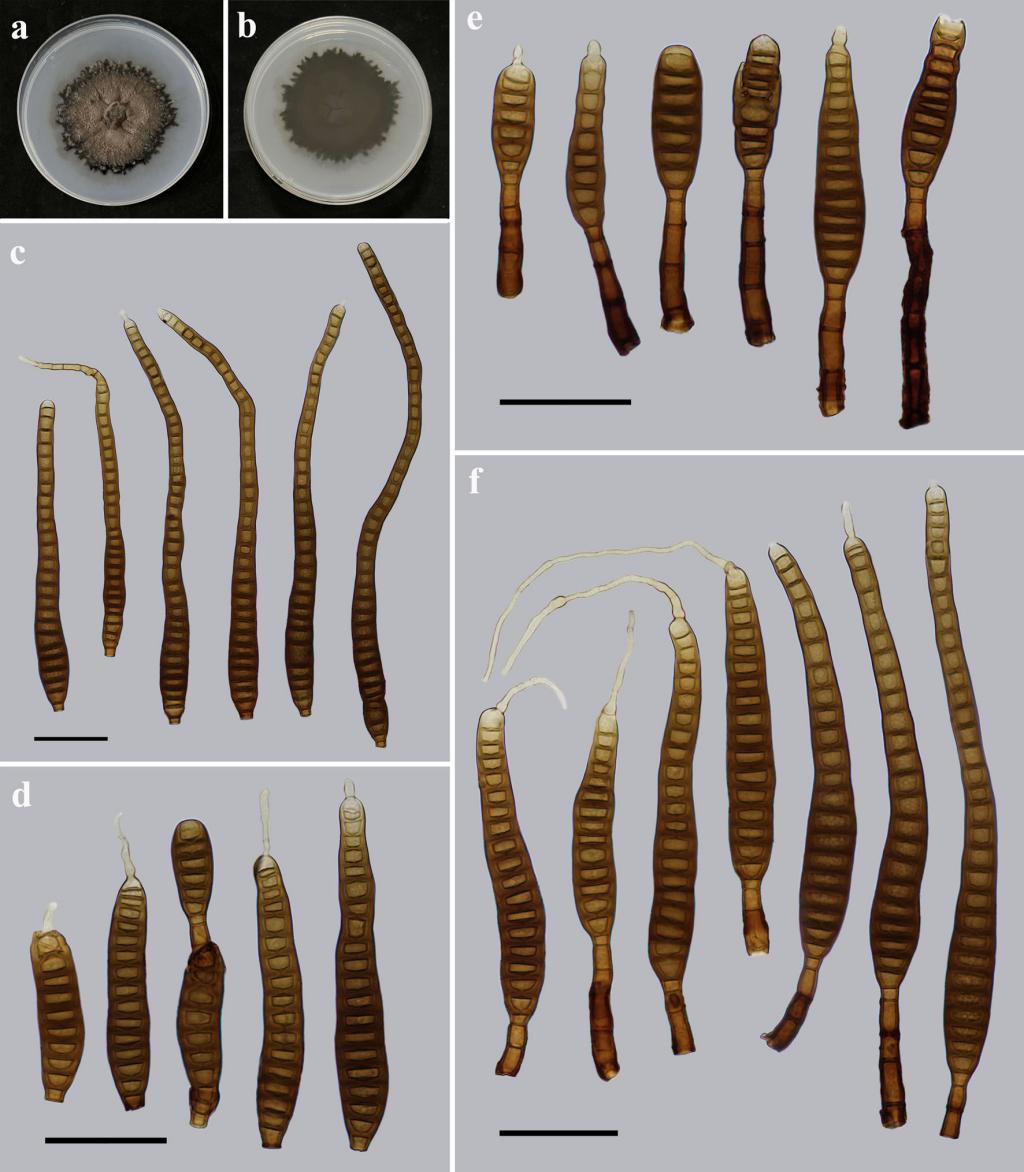
Morphology of *Distoseptispora nanchangensis* (from holotype HJAUP M1074). (**a**) The surface of the colony after 4 weeks on PDA; (**b**) reverse of the colony after 4 weeks on PDA; (**c and d**) conidia; and (**e and f**) conidiophores, conidiogenous cells, and conidia. Scale bars (c to f), 20 µm.

*Distoseptispora nanchangensis* Y.F. Hu & Jian Ma, sp. nov. (Fig. 8).

MycoBank number: MB849134

Etymology: In reference to the locality, Nanchang City, where the fungus was collected.

Typus: China, Jiangxi Province, Nanchang City, Meiling Mountain, 28°47′45.20″N, 115°45′18.76″E, on dead branches of an unidentified broadleaf tree, 28 June 2021, Y.F. Hu (holotype HJAUP M1074; ex-type living culture HJAUP C1074).

Description: Saprobic on dead branches in terrestrial habitats. Sexual morph: Undetermined. Asexual morph: Hyphomycetes. Colonies on natural substrate effuse, scattered, dark brown to black, and hairy. Mycelium is superficial and immersed, composed of branched, septate, smooth-walled, and pale brown to brown hyphae. Conidiophores 18.2–76.4 × 5.5–8.0 µm (*x̅* =40.6 × 7.2 µm, SD = 15 × 0.6, *n* = 20), macronematous, mononematous, brown to dark brown, solitary, 2–5-septate, straight or flexuous, unbranched, smooth, and cylindrical. Conidiogenous cells are monoblastic, integrated, terminal, cylindrical, determinate or sometimes with cylindrical, enteroblastic percurrent extensions, pale brown, and smooth. Conidia 149.1–292.7 × 10.9–17.8 µm (*x̅* =203.5 × 15.4 µm, SD = 45 × 1, *n* = 30), acrogenous, solitary, obclavate, straight or curved, (17–)21–43-distoseptate, brown to dark brown, smooth, thick-walled, sometimes with percurrent regeneration forming a secondary conidium from the conidial apex, truncate at the base, rounded at the apex, apex sometimes with a germ tube or short germination hypha.

Culture characteristics: Colony on PDA reaching 65–70 mm diameter. After 4 weeks in an incubator under dark conditions at 25°C, circular, raised, surface velvety, aerial, sparser, dark brown; reverse dark brown to black.

Notes: The phylogenetic tree showed that *D. nanchangensis* (HJAUP C1074) clusters with *D. aquatica* (MFLUCC 18–0646 and S-965). BLASTn analysis of *D. nanchangensis* (HJAUP C1074) and *D. aquatica* (MFLUCC 18–0646) showed 99% identity (576/578, no gap) using ITS, 100% identity (524/524, no gap) using LSU, and 99% identity (835/839, no gap) using *TEF1*. Moreover, *D. nanchangensis* is significantly different from *D. aquatica* (6) in its longer and narrower conidiophores (18.2–76.4 × 5.5–8 µm vs 29–41 × 7–9 µm), and longer conidia (149.1–292.7 µm vs 110–157 µm) with more distosepta [(17–)21–43 vs 15–28]. *Distoseptispora nanchangensis* is morphologically similar to *D. tectonae* (35) but differs by its bigger conidiophores (18.2–76.4 × 5.5–8 µm vs up to 40 × 4–6 µm), and bigger conidia [149.1–292.7 × 10.9–17.8 µm vs (90–)130–140(–170) × (11–)13–14(–16) µm] with more distosepta [(17–)21–43 vs 20–28] and a germ tube or short hyphae from the apex.

**Fig 9:**
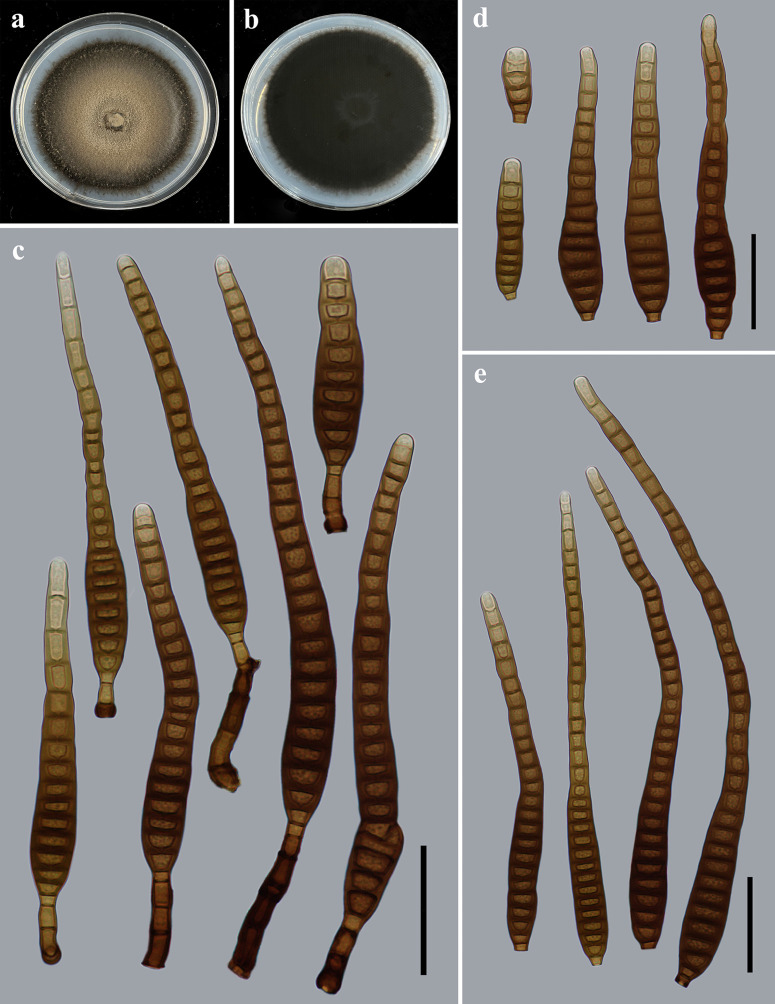
Morphology of *Distoseptispora yichunensis* (from holotype HJAUP M1065). (**a**) The surface of the colony after 4 weeks on PDA; (**b**) reverse of the colony after 4 weeks on PDA; (**c**) conidiophores, conidiogenous cells, and conidia; and (**d and e**) conidia. Scale bars (c to e), 40 µm.

*Distoseptispora yichunensis* Y.F. Hu & Jian Ma, sp. Nov. (Fig. 9).

MycoBank number: MB849135

Etymology: In reference to the locality, Yichun City, where the fungus was collected.

Typus: China, Jiangxi Province, Yichun City, Guanshan National Nature Reserve, 28°32′21.67″N, 114°33′52.38″E, on dead branches of an unidentified broadleaf tree, 25 June 2021, Y.F. Hu (holotype HJAUP M1065; ex-type living culture HJAUP C1065).

Description: Saprobic on dead branches in terrestrial habitats. Sexual morph: Undetermined. Asexual morph: Hyphomycetes. Colonies on natural substrate effuse, brown, and hairy. Mycelium is superficial and immersed, composed of branched, septate, smooth, and pale brown to brown hyphae. Conidiophores 17.9–52.7 × 5.3–6.8 µm (*x̅* =33.7 × 6.2 µm, SD = 16 × 0.5, *n* = 20), macronematous, mononematous, solitary, stralight or flexuous, 3–6-septate, unbranched, smooth, cylindrical, and brown to dark brown. Conidiogenous cells are monoblastic, integrated, terminal, cylindrical, determinate or sometimes with cylindrical, enteroblastic percurrent extensions, pale brown to brown, smooth. Conidia 113.7–272.7 × 12.2–16.9 µm (*x̅* =159.2 × 14.5 µm, SD = 48 × 1, *n* = 30), acrogenous, solitary, obclavate, straight or curved, pale brown to brown (14–)22–35-distoseptate, smooth, truncate at the base, rounded at the apex.

Culture characteristics: Colony on PDA reaching 78–83 mm diameter after 4 weeks in an incubator under dark conditions at 25°C, circular, surface velvety, with brown, denser mycelium at the center, becoming black at the entire margin; reverse dark brown to black.

Notes: The phylogenetic tree showed that the strain of *D. yichunensis* (HJAUP C1065) forms an independent clade and clusters with the strains of *D. sinensis* (HJAUP C2044) and *D. tectonae* (MFLUCC 15–0981, MFLUCC 12–0291, MFLU 20–0262, and MFLUCC 16–0946). BLASTn analysis of *D. yichunensis* (HJAUP C1065) and *D. sinensis* (HJAUP C2044) showed 97% identity (599/615, four gaps) using ITS, 99% identity (562/570, three gaps) using LSU and 99% identity (922/927, no gap) using *TEF1*; of *D. yichunensis* (HJAUP C1065) and *D. tectonae* (MFLUCC 12–0291) showed 98% identity (565/574, four gaps) using ITS, 99% identity (562/566, two gaps) using LSU, 99% identity (924/927, no gaps) using *TEF1* and 99% identity (823/830, no gaps) using *RPB2*. Moreover, *D. yichunensis* is significantly different from *D. sinensis* (15) in its shorter conidiophores (17.9–52.7 µm vs 23.5–56.5 µm) and bigger conidia [113.7–272.7 × 12.2–16.9 µm vs 40–107(–137) ×10–12 µm] with more distosepta [(14–)22–35 vs 10–25], and from *D. tectonae* (35) in its bigger conidiophores (17.9–52.7 × 5.3–6.8 µm vs up to 40 × 4–6 µm), and bigger conidia [113.7–272.7 × 12.2–16.9 µm vs (90–)130–140(–170) × (11–)13–14(–16) μm] with more distosepta [(14–)22–35 vs 20–28], as well as from *D. neorostrata* (12), which has longer conidiophores (93–117 µm vs 17.9–52.7 µm), and smaller conidia (109–147 × 13–15 µm vs 113.7–272.7 × 12.2–16.9 µm).

## DISCUSSION

In recent years, the number of new taxa in the *Distoseptispora* has steadily increased, which have been discovered mostly in freshwater and some in terrestrial habitats. However, as terrestrial habitats are a vast habitat for saprobic fungi, there may be more species in this genus to be discovered and further research are needed to elucidate whether specific species in *Distoseptispora* are specific to their habitats. The establishment of *Distoseptispora* was based on morphology and phylogenetic studies. In all, 66 epithets for *Distoseptispora* have been listed in Index Fungorum (14), and of which 65 are accepted (15 – 17). Members of this genus occur mainly as asexual morphs, forming effuse, hairy colonies on decaying wood, plant stems, bamboo poles, fallen leaves, and shafts from both terrestrial and freshwater habitats (5). The genus *Distoseptispora* (*Distoseptisporaceae*, *Distoseptisporales*) is mainly characterized by macronematous, mononematous, septate, unbranched, cylindrical, olivaceous to brown conidiophores; mono- or polyblastic, integrated, terminal, cylindrical, determinate or percurrently extending conidiogenous cells; and acrogenous, solitary, smooth or verruculose, euseptate or distoseptate conidia. However, *Distoseptispora* species show a high morphological similarity to *Sporidesmium* and *Ellisembia* with euseptate and distoseptate conidia, but it is hard to identify some *Distoseptispora* species by morphological features alone. Thus, with the availability of sequence data for *Distoseptispora* species, the introduction of molecular phylogenetic analyses led to a better understanding of the species diversity of *Distoseptispora* (5, 15, 35, 36). Studies conducted to date on *Distoseptispora* have no universally accepted standards in selecting loci for phylogenetic analyses (15). However, recent studies indicated that the use of LSU, ITS, *TEF1,* and *RPB2* shows good phylogenetic resolution in resolving the phylogeny of *Distoseptisporaceae* (18, 30). Other loci in the mitochondrial genome are a potential model for molecular evolutionary and phylogenetic studies and has been important for taxonomy, phylogenetics, and population genetics of fungi (37 – 39), but not a single mitogenome to date has been characterized in *Distoseptispora*. The characteristics of mitogenomes belonging to different representatives of *Distoseptispora* are needed to facilitate further investigations into the taxonomy, phylogenetics, conservation genetics, and evolutionary biology of this genus and other closely related species, to clarify the understanding of the evolution and potential technological uses of specific isolates of the genus.

*Distoseptispora* as a single genus in *Distoseptisporaceae* was introduced by Su et al. (6) with *D. fluminicola* as the type species based on morphological and phylogenetic analyses. The genus is known for its asexual morph, and only two sexual species, viz. *D. hyalina* and *D. licualae* are reported, but their related anamorph is still unknown (5, 32). Morphology and especially the conidial shape in *Distoseptispora* species are highly diverse. *Distoseptispora* has shown convergent evolution as a member of *sporidesmium*-like genera but has not been well represented in phylogenetic analyses to support morphological classification. A comparison of morphological characters and phylogenetic analysis of the various species in *Distoseptispora* reveals that the conidia of most species are obclavate to cylindrical or rostrate (e.g., *D. aquatica*, *D. leonensis,* and *D. longispora*), and a few are ellipsoidal to subglobose (e.g., *D. martinii* and *D. atroviridis*), and lanceolate (e.g., *D. guttulata* and *D. multiseptata*). However, these characteristics are not significantly correlated with the phylogenetic relationship. Combining the morphological characteristics and the phylogenetic evidence of all species in the genus *Distoseptispora*, we found that some *Distoseptispora* species are highly similar morphologically, but can nevertheless be well separated by molecular DNA data. For example, conidia of *D. multiseptata*, *D. phangngaensis*, and *D. xishuangbannaensis* are all characterized as acrogenous, solitary, obclavate, multi-distoseptate (6, 9, 35); *D. tropica* and *D. verrucosa* also have an identical morphology of conidiophores and conidia (5, 30), but there are have a distant phylogenetic relationship. In addition, there are a number of species that are sister clades in the phylogenetic tree but exhibit different morphological characters. For example, *D. nabanheensis* has obclavate, slightly constricted at the septa, brown to dark brown conidia, while *D. clematidis* has oblong, obclavate, cylindrical or rostrate, brown with green tinge conidia (15, 40); *D. longispora* and *D. longnanensis* have a close phylogenetic relationship, but *D. longispora* has obclavate, rounded at the apex, truncate at the base, 31–56-distoseptate, brown to yellowish brown, slightly paler toward the apex, while *D. longnanensis* has obclavate, tapering toward the rounded apex, 4–8-septate, olivaceous to yellowish-brown or brown, becoming paler toward the apex, with a darkened scar at the base. Therefore, due to the unique phenomenon of *Distoseptispora*, it needs to be verified and extended in the future by morphological characterization and phylogenetic analyses.

Among the prevalent woody litter saprobes in terrestrial and freshwater ecosystems, dematiaceous *sporidesmium*-like hyphomycetes are dominant (5). This genus is considered to be a saprobic lignicolous fungal genus that can decompose lignocellulose in wood and to participate in the decomposition and nutrient cycling of dead plant material in terrestrial and aquatic ecosystems (35, 41). The diversity in a particular area or ecosystem is usually expressed as the number of species in the system, highlighting the fundamental role of taxonomy in biodiversity assessment and biology (42, 43). However, knowledge of the role of the genus in decomposition and nutrient cycling, their geographical distribution, environment, host information, substrate specificity, and teleomorph relationships are relatively limited. Therefore, it is not possible to quantify their role in ecosystem functioning. The reports of *Distoseptispora* are mainly concentrated in Thailand (Chiang Rai, Phitsanulok, Phang Nga) and China (Yunnan and Guizhou Province) (7, 9, 12), but three *Distoseptispora* species, *D. meilingensis*, *D. yongxiuensis,* and *D. yunjushanensis*, were found in Jiangxi, China (20), and *D. septata*, *D. tropica,* and *D. wuzhishanensis* were found in Hainan, China (30). In this paper, eight taxa were identified from terrestrial habitats in Jiangxi and Yunnan Provinces, China. After morphological comparison and phylogenetic analyses, it can be shown that these eight collections are new species of *Distoseptispora*, widening the diversity of the genus. Therefore, surveys of different geographical areas, ecological environments, and vegetation types are needed to help reveal the genus diversity, increase the fungal species number curve, and further clarify their taxonomic status through phylogenetic analyses.

## MATERIALS AND METHODS

### Sample collection, isolation, and morphological observation

Samples of dead branches were collected randomly from humid environments and river banks in the forest ecosystems of Yunnan and Jiangxi Provinces, China, and returned to the laboratory in Ziploc plastic bags. Samples were processed and examined following the methods described in Ma et al. (44). Colonies on the surface of dead branches were examined and visually observed using a stereomicroscope (Motic SMZ-168, Xiamen, China) from low magnification (0.75 times) to high magnification (five times). Fresh colonies were picked with a sterile needle at 5× magnification under a stereomicroscope, placed on a slide with a drop of lactic acid-phenol solution (lactic acid, phenol, glycerol, sterile water; 1:1:2:2:1, respectively), and then placed under an Olympus BX 53 light microscope equipped with an Olympus DP 27 digital camera (Olympus Optical Co., Tokyo, Japan) for microscopic morphological characterization. The conidia of the target colony were captured directly from the specimen using the tip of a sterile toothpick dipped in sterile water. Conidia were then placed on the surface of PDA (20% potato + 2% dextrose + 2% agar, wt/vol) and incubated overnight in an incubator at 25°C. The single germinated conidia were transferred to fresh PDA plates according to the method of Goh (45) and incubated in an incubator at 25°C. Culture characteristics were examined and recorded after 3 days, followed by 3 days at regular intervals. Colony colors were assessed according to the charts of Rayner (46). All fungal strains were stored in 10% sterilized glycerin at 4°C for further studies. The studied specimens and cultures were preserved in the Herbarium of Jiangxi Agricultural University, Plant Pathology, Nanchang, China (HJAUP). The names of the new taxa names were registered in Index Fungorum (14).

### DNA extraction, PCR amplification, and sequencing

Fungal hyphae were scraped from the surface of colonies grown on PDA plates, and genomic DNA was extracted using the Solarbio Fungal Genomic DNA Extraction Kit (Beijing Solarbio Science & Technology Co., Ltd., Beijing, China) according to the manufacturer’s protocol. DNA amplification was performed by polymerase chain reaction (PCR) using the respective loci (ITS, LSU, *TEF1*, and *RPB2*). Primer sets used for these genes were as follows: ITS: ITS5/ITS4 (47), LSU: 28S1-F/28S3-R (21), *TEF1*: EF1-983F/EF1-2218R (48, 49), and *RPB2*: RPB2-5F2 (50)/fRPB2-7cR (51). The final volume of the PCR was 25 µL, containing 12.5 µL of 2 × Power Taq PCR MasterMix, 1 µL of each forward and reverse primer, 1 µL of DNA template, and 9.5 µL of ddH_2_O. The PCR thermal cycling conditions of ITS, LSU, and *TEF1* were initialized at 94°C for 3 min, followed by 35 cycles of denaturation at 94°C for 15 s, annealing at 54°C for 15 s, elongation at 72°C for 30 s, a final extension at 72°C for 10 min, and finally kept at 4°C. Regions of *RPB2* were amplified with annealing at 59°C for 15 s, elongation at 72°C for 120 s, and others consistent with the above procedure. The PCR products were checked on 1% agarose gel electrophoresis stained with ethidium bromide. Purification and DNA sequencing were carried out at Beijing Tsingke Biotechnology Co., Ltd., Beijing, China. New sequences generated in this study were deposited in the NCBI GenBank (www.ncbi.nlm.nih.gov, Table 1).

**TABLE 1:** Taxa used in the phylogenetic analyses and their GenBank accession numbers^*a*^ ^,^ ^*b*^ ^,^ ^*c*^

Species	Strain no.	GenBank accession no.			
		LSU	ITS	TEF1	RPB2
*Acrodictys bambusicola*	CGMCC 3.18641^T^	KX033564	KU999973	—	—
*A. elaeidicola*	CGMCC 3.18642	KX033569	KU999978	—	—
*Aquapteridospora aquatica*	MFLUCC 17-2371^T^	MW287767	MW286493	—	—
*A. fusiformis*	MFLUCC 18-1606^T^	MK849798	MK828652	MN194056	—
*A. lignicola*	MFLUCC 15-0377^T^	KU221018	MZ868774	MZ892980	MZ892986
*Bullimyces aurisporus*	AF316-1b^T^	JF775590	—	—	—
*B. communis*	AF281-5	JF775587	—	—	—
*Cancellidium applanatum*	CBS 337.76^T^	MH872755	MH860985	—	—
*Cancellidium cinereum*	MFLUCC 18-0424^T^	MT370363	MT370353	MT370488	MT370486
*Distoseptispora adscendens*	HKUCC 10820	DQ408561	—	—	DQ435092
*D. amniculi*	MFLUCC17-2129^T^	MZ868761	MZ868770	—	MZ892982
*D. appendiculata*	MFLUCC 18-0259^T^	MN163023	MN163009	MN174866	—
*D. aqualignicola*	KUNCC 21-10729^T^	ON400845	OK341186	OP413480	OP413474
*D. aquamyces*	KUNCC 21-10732^T^	OK341199	OK341187	OP413482	OP4 13476
*D. aquatica*	MFLUCC 18-0646	MK849793	MK828648	MN194052	—
*D.aquatica*	S-965	MK849792	MK828647	MN194051	MN124537
*D. aquisubtropica*	GZCC 22-0075^T^	ON527941	ON527933	ON533677	ON533685
*D. atroviridis*	GZCC 20-0511^T^	MZ868763	MZ868772	MZ892978	MZ892984
*D. atroviridis*	GZCC 19-0531	MZ227223	MW133915	MZ206155	—
*D. bambusae*	MFLUCC 20-0091^T^	MT232718	MT232713	MT232880	MT232881
*D. bambusae*	MFLUCC 14-0583	MT232717	MT232712	—	MT232882
*D. bambusicola*	GZCC21-0667^T^	MZ474872	MZ474873	OM272845	—
*D. bangkokensis*	MFLUCC 18-0262^T^	MZ518206	MZ518205	OK067246	—
*D. cangshanensis*	MFLUCC 16-0970^T^	MG979761	MG979754	MG988419	—
*D. caricis*	CPC 36498^T^	MN567632	MN562124	—	MN556805
*D. chinensis*	GZCC21-0665^T^	MZ474867	MZ474871	MZ501609	—
*D. clematidis*	MFLUCC 17-2145^T^	MT214617	MT310661	—	MT394721
*D. clematidis*	KUN-HKAS 112708	MW879523	MW723056	—	—
*D. crassispora*	KUMCC 21-10726^T^	OK341196	OK310698	OP413479	OP413473
*D. curvularia*	KUMCC 21-10725^T^	OK341195	OK310697	OP413478	OP413472
*D. cylindricospora*	DLUCC 1906^T^	OK513523	OK491122	OK524220	—
*D.dehongensis*	KUMCC 18-0090^T^	MK079662	MK085061	MK087659	—
*D. dipterocarpi*	MFLUCC 22–0104^T^	OP600052	OP600053	—	OP595140
*D. effusa*	GZCC19-0532^T^	MZ227224	MW133916	MZ206156	—
*D. euseptata*	MFLUCC 20–0154^T^	MW081544	MW081539	—	MW151860
*D. euseptata*	DLUCC S2024	MW081545	MW081540	MW084994	MW084996
*D. fasciculata*	KUMCC 19–0081^T^	MW287775	MW286501	MW396656	—
*D. fluminicola*	DLUCC 0391	MG979762	MG979755	MG988420	—
*D. fluminicola*	DLUCC 0999	MG979763	MG979756	MG988421	—
*D. fusiformis*	GZCC 20–0512^T^	MZ868764	MZ868773	MZ892979	MZ892985
***D. gasaensis***	**HJAUP C2034**	**OQ942891**	**OQ942896**	**OQ944455**	—
***D. guanshanensis***	**HJAUP C1063**	**OQ942898**	**OQ942894**	**OQ944452**	**OQ944458**
*D. guizhouensis*	GZCC21-0666^T^	MZ474869	MZ474868	MZ501610	MZ501611
*D. guttulata*	MFLU 17–0852^T^	MF077554	MF077543	MF135651	—
*D. hyalina*	MFLUCC 17–2128^T^	MZ868760	MZ868769	MZ892976	MZ892981
*D. hydei*	MFLUCC 20–0481^T^	MT742830	MT734661	—	MT767128
***D. jinghongensis***	**HJAUP C2120**	**OQ942893**	**OQ942897**	**OQ944456**	—
*D. lancangjiangensis*	DLUCC 1864^T^	MW879522	MW723055	—	—
*D. leonensis*	HKUCC 10822	DQ408566	—	—	DQ435089
*D. licualae*	MFLUCC 14–1163A^T^	ON650675	ON650686	ON734007	—
*D. licualae*	MFLUCC 14–1163B^T^	ON650676	ON650687	ON734008	—
*D. licualae*	MFLUCC 14–1163C^T^	ON650677	ON650688	—	—
*D. lignicola*	MFLUCC 18–0198^T^	MK849797	MK828651	—	—
*D. lignicola*	GZCC 19–0529	MZ227219	MW133911	MZ206152	—
*D. longispora*	HFJAU 0705^T^	MH555357	MH555359	—	—
***D. longnanensis***	**HJAUP C1040**	**OQ942886**	**OQ942887**	**OQ944451**	—
*D. martinii*	CGMCC 3.18651^T^	KX033566	KU999975	—	—
*D. meilingensis*	JAUCC 4727^T^	OK562396	OK562390	OK562408	—
*D. meilingensis*	JAUCC 4728	OK562397	OK562391	OK562409	—
***D. menghaiensis***	**HJAUP C2045**	**OQ942900**	**OQ942890**	—	—
***D. menglunensis***	**HJAUP C2170**	**OQ942888**	**OQ942899**	**OQ944457**	**OQ944461**
*D. mengsongensis*	HJAUP C2126^T^	OP787874	OP787876	OP961937	—
*D. multiseptata*	MFLUCC 15–0609^T^	KX710140	KX710145	MF135659	—
*D. multiseptata*	MFLU 17–0856	MF077555	MF077544	MF135652	MF135644
*D. nabanheensis*	HJAUP C2003^T^	OP787877	OP787873	OP961935	**—**
***D. nanchangensis***	**HJAUP C1074**	**OQ942895**	**OQ942889**	**OQ944454**	**OQ944460**
*D. neorostrata*	MFLUCC 18–0376^T^	MN163017	MN163008	—	—
*D. nonrostrata*	KUNCC 21–10730^T^	OK341198	OK310699	OP413481	OP413475
*D. obclavata*	MFLUCC 18–0329^T^	MN163010	MN163012	—	—
*D. obpyriformis*	MFLUCC 17–1694^T^	MG979764	—	MG988422	MG988415
*D. obpyriformis*	DLUCC 0867	MG979765	MG979757	MG988423	MG988416
*D. pachyconidia*	KUMCC 21–10724^T^	OK341194	OK310696	OP413477	OP413471
*D. palmarum*	MFLUCC 18–1446^T^	MK079663	MK085062	MK087660	MK087670
*D. phangngaensis*	MFLUCC 16–0857^T^	MF077556	MF077545	MF135653	—
*D. rayongensis*	MFLUCC 18–0415^T^	MH457137	MH457172	MH463253	MH463255
*D. rayongensis*	MFLUCC 18–0417	MH457138	MH457173	MH463254	MH463256
*D. rostrata*	MFLUCC 16–0969^T^	MG979766	MG979758	MG988424	MG988417
*D. rostrata*	DLUCC 0885	MG979767	MG979759	MG988425	—
*D. saprophytica*	MFLUCC 18–1238^T^	MW287780	MW286506	MW396651	MW504069
*D. septata*	GZCC 22–0078^T^	ON527947	ON527939	ON533683	ON533690
*D. songkhlaensis*	MFLUCC 18–1234^T^	MW287755	MW286482	MW396642	—
*D. sinensis*	HJAUP C2044^T^	OP787875	OP787878	OP961936	—
*D. suoluoensis*	MFLUCC 17-0224^T^	MF077557	MF077546	MF135654	—
*D. suoluoensis*	MFLU 17-0854	MF077558	MF077547	—	MZ945510
*D. tectonae*	MFLUCC 15-0981	MW287763	MW286489	MW396641	—
*D. tectonae*	MFLUCC 12-0291^T^	KX751713	KX751711	KX751710	KX751708
*D. tectonae*	MFLUCC 16-0946	MG979768	MG979760	MG988426	MG988418
*D. tectonae*	MFLU 20-0262	MT232719	MT232714	—	—
*D. tectonigena*	MFLUCC 12-0292^T^	KX751714	KX751712	—	KX751709
*D. thailandica*	MFLUCC 16-0270^T^	MH260292	MH275060	MH412767	—
*D. thysanolaenae*	KUN-HKAS 112710	MW879524	MW723057	MW729783	—
*D. thysanolaenae*	KUN-HKAS 102247^T^	MK064091	MK045851	MK086031	—
*D. tropica*	GZCC 22-0076^T^	ON527943	ON527935	ON533679	ON533687
*D. verrucosa*	GZCC 20-0434^T^	MZ868762	MZ868771	MZ892977	MZ892983
*D. wuzhishanensis*	GZCC 22-0077^T^	ON527946	ON527938	ON533682	—
*D. xishuangbannaensis*	KUMCC 17-0290^T^	MH260293	MH275061	MH412768	MH412754
***D. yichunensis***	**HJAUP C1065**	**OQ942892**	**OQ942885**	**OQ944453**	**OQ944459**
*D. yongxiuensis*	JAUCC 4725^T^	OK562394	OK562388	OK562406	—
*D. yongxiuensis*	JAUCC 4726	OK562395	OK562389	OK562407	—
*D. yunjushanensis*	JAUCC 4723^T^	OK562398	OK562392	OK562410	—
*D. yunjushanensis*	JAUCC 4724	OK562399	OK562393	OK562411	—
*D. yunnansis*	MFLUCC 20-0153^T^	MW081546	MW081541	MW084995	MW151861
*Fluminicola saprophytica*	MFLUCC 15-0976^T^	MF374367	MF374358	MF370956	MF370954
*Myrmecridium banksiae*	CPC 19852 = CBS 132536^T^	JX069855	JX069871	—	—
*M. schulzeri*	CBS 100.54	EU041826	EU041769	—	—
*Papulosa amerospora*	AFTOL-ID 748	DQ470950	—	DQ471069	DQ470901
*Pleurophragmium bambusinum*	MFLUCC 12-0850	KU863149	KU940161	KU940213	—
*Pseudostanjehughesia aquitropica*	MFLUCC 16-0569^T^	MF077559	MF077548	MF135655	—
*P. lignicola*	MFLUCC 15-0352^T^	MK849787	MK828643	MN194047	MN124534
*Wongia griffinii*	DAR 80512^T^	KU850471	KU850473	—	—

^*a*^ The ex-type cultures are indicated using T after strain numbers; — stands for no sequence data in GenBank.

^*b*^ AFTOL: Assembling the Fungal Tree of Life; CBS: Central Bureau voor Schimmel cultures, Utrecht, The Netherlands; CGMCC: China General Microbiological Culture Collection Center, Institute of Microbiology, Chinese Academy of Sciences, Beijing, China; CPC: Collection of P.W. Crous; DAR: Plant Pathology Herbarium, Orange Agriculture Institute, NSW, Australia; DLUCC: Dali University Culture Collection, Yunnan; GZCC: Guizhou Culture Collection China; HFJAU: Herbarium of Fungi, Jiangxi Agricultural University; HKUCC: The University of Hong Kong Culture Collection, Hong Kong, China; JAUCC: Jiangxi Agricultural University Culture Collection; KUMCC: Kunming Institute of Botany Culture Collection; KUN HKAS: Kunming Institute of Botany Academia Sinica, Yunnan, China; MFLU: the herbarium of Mae Fah Luang University, Chiang Rai, Thailand; MFLUCC: Mae Fah Luang University Culture Collection, Chiang Rai, Thailand.

^*c*^ Newly generated sequences are in bold.

### Phylogenetic analyses

The newly generated sequences were aligned with other sequences obtained from GenBank (Table 1) using MAFFTv.7 (52) on the online server (https://maffth.cbrc.jp/alignment/server/, accessed on 19 April 2023) and manually optimized where required. Phylogenetic analyses were first performed for each locus separately, and then for a combined data set of four gene loci (ITS, LSU, *TEF1,* and *RPB2*). The tandem sequences for ITS, LSU, *TEF1,* and *RPB2* were obtained by Phylosuite software v1.2.1 (53) under “Concatenate Sequence,” and missing sequence data in the comparisons were used as missing data with a question mark. Phylosuite software v1.2.1 (53) was used to construct the phylogenetic tree based on ITS, LSU, *TEF1,* and *RPB2* tandem sequence data. The concatenated aligned data set was analyzed separately using maximum-likelihood (ML) and Bayesian inference (BI). The best-fitting nucleotide substitution models for each alignment data set were selected using ModelFinder (54). Maximum-likelihood phylogenies were inferred using IQ-TREE (55) under an Edge-linked partition model for 10,000 ultrafast bootstraps (56). The final tree was selected among suboptimal trees from each run by comparing the likelihood scores using the TIM2e + I + G4 for ITS, TIM2 + F + R4 for LSU and *TEF1*, and TIM3e + I + G4 for *RPB2* substitution model. Bayesian inference phylogenies were inferred using MrBayes 3.2.6 (57) under a partition model (two parallel runs, 2,000,000 generations), in which the initial 25% of sampled data were discarded as burn-in. The best-fit model was GTR + F + I + G4 for ITS, LSU, and *TEF1*, and SYM + I + G4 for *RRPB2*. ModelFinder (54) was used to select the best fitting partition model (Edge-linked) using the BIC criterion to construct an IQ-TREE and using the AlCc criterion to construct a MrBayes. These trees were visualized using FigTree v. 1.4.4 (http://tree.bio.ed.ac.uk/software/figtree, accessed on 19 April 2023), with editing and typesetting using Adobe Illustrator CS v. 5.

## Data Availability

All sequences generated in the present study were deposited in GenBank, and the accession numbers are listed in Table 1.

## SUPPLEMENTAL MATERIAL

Fig. S1 spectrum.02468-23-s0001.pdf The concatenated ITS, LSU, RPB2, and TEF1 multiple sequence alignment.

ASM does not own the copyrights to Supplemental Material that may be linked to, or accessed through, an article. The authors have granted ASM a non-exclusive, world-wide license to publish the Supplemental Material files. Please contact the corresponding author directly for reuse.
