# Supplementary material for: Multi-locus phylogenetic analyses reveal eight novel species of Distoseptispora from southern China
Source: Microbiol Spectr. 2023 Oct 31;11(6):e02468-23. doi: 10.1128/spectrum.02468-23 (PMC10715003; doi:10.1128/spectrum.02468-23)
Supplement: Fig. S1 — The concatenated ITS, LSU, RPB2, and TEF1 multiple sequence alignment. [file spectrum.02468-23-s0001.pdf]

Figure S1: The concatenated ITS, LSU, *RPB2* and *TEF1* multiple sequence alignment.

>Distoseptispora_longnanensis_HJAUP_C1040
ACCT--------TTTATATT-------CGTTGCTTTGGCGGG-CGGCTCC-AGGGCGGAGTCACTACCCGT
TGGGTCGGGCGCCCGCCGAAGGA---CAAC-CAAAACTCGCT-AAATCTGT-ACGGATCTCAGA
GTAAAA------TAT-----CAAGCTAT-CAAAACTTTCAGCAACGGATCTCTTGGTTCTGGCATCGATG
AAGAACGCAGCGAAATGCGATAAGTAATGTGAATTGCAGAATTCAGTGAATCATCGAATCTTT
GAACGCACATTGCGCCCGCCAGCACTCTGGCGGGCATGCCTGTTCGAGCGTCATTTCAA-CC
CTCGGGCCC-TCGTT-------TGGCCCGGTGTTGGGGCGCTACGGGTCTG--TC----------------GG
AC-CTGTAGGCCCTCAAAAGTAGTGGCGGGCTCGCCAGGACT-CCGAGCGTAGTAGTTC-TCC
T--CTCGCTCAGGCGGCCT-GGCGTG--CT-TCGGCCGTTAAA---GACCCAAC------TGTAAT-----C
ACAG-AAGCCTTCCGAGTCCCC-TGGAAC------GGGGCGCC-AGAGAGGG-TGAGAGCCCCGT
ATGGTTGGACGCTGAACC-TCTGTAAAGCTCCTTCGACGAGTCGAGTAGTTTGGGAATGCTG
CTCAAAATGGGAGGTAAATTTCTTCTAAAG-CTAAATACCGGCCAGAGACCGATAGCGCACAA
GTAGAGTGATCGAAAGATGAAAAGCACTTTGAAAAGAGGGTTAAATAGCACGTGAAATTGCTG
AAAGGGAAGCGCTTGTGACCAGACTTGCGCCCGGTTGATCATCCAGCGTTCTCGCTGGTGC
ACTCTGCCGGGCTCAGGCCAGCATCGGCTCTCCCAGGGGGATAAAGGTCGCGGGAACGTA
GCTCTCTCCGGGGAGTGTTATAGCCCGTGG-CGGAATGCCCTTAGGGGGGGCCGAGGCCCG
CGTTC-G-CAAGGATGCTGGCGTAATGGTCACCAGCGACCCG-TCT?????????????????????
??
??
??
??
??
??
??
??
???ACTCGTGAGCACGCTCTGCTT
GCCTACACCTTGGGTGTGAAGCAGCTCATCGTCGCCATCAACAAGATGGACACCACCAAATG
GTCTGAGAGCCGTTACAACGAAATTATCAAGGAGACGTCCAACTTCATCAAGAAGGTCGGCT
ACAACCCCAAGCAGGTCGCCTTCGTTCCCATCTCCGGCTTCAACGGCGACAACATGCTTGC
CCCCTCCACCAACTGCCCCTGGTACAAGGGTTGGGAGAAGGAGATC---AAGGGCGGCAAGG
CCACTGGCAAGACCCTTCTCGAGGCCATCGATGCTATTGAGCCTCCCAAGCGTCCTACGGAC
AAGCCTCTGCGTCTACCTCTCCAGGATGTCTACAAGATTGGTGGTATTGGAACTGTGCCCGT
CGGCCGTATCGAGACTGGTATCCTCAAGCCCGGTATGGTCGTTACCTTCGCTCCCTCCAACG
TCACCACTGAAGTCAAGTCCGTCGAGATGCACCACGAGCAGCTGGCGGAGGGTGTCCCCG
GCGACAACGTCGGCTTCAACGTGAAGAACGTCTCCGTCAAGGAAATCCGCCGAGGCAACGT
TGCTGGTGACTCCAAGAACGATCCTCCTGCTGGTGCCGCTTCCTTCACCGCCCAGGTCATC
GTCCTCAACCACCCCGGTCAGGTCGGCGCTGGTTACGCTCCCGTCTTGGATTGCCACACTG
CCCACATCGCTTGCAAGTTCTCTGAGATTCTGGAGAAGATCGACCGCCGAACGGGCAAGTCT
GTCGAGAACAACCCCAAGTTCATCAAGTCTGGTGATGC
>Distoseptispora_guanshanensis_HJAUP_C1063
ACCT--------TT-ATATT-------CGTTGCTTTGGCGGG-CGGCTCC-AGGGCGGAGTCACTACCCGT
TGGGTCGGGCGCCCGCCGAAGGA---CAAC-CAAAACTCGCT-AAATCTGT-ACGGATCTCAGA

GTAAAA------TAT-----CAAGCTAT-CAAAACTTTCAGCAACGGATCTCTTGGTTCTGGCATCGATG
AAGAACGCAGCGAAATGCGATAAGTAATGTGAATTGCAGAATTCAGTGAATCATCGAATCTTT
GAACGCACATTGCGCCCGCCAGCACTCTGGCGGGCATGCCTGTTCGAGCGTCATTTCAA-CC
CTCGGGCCC-TCGTT-------TGGCCCGGTGTTGGGGCGCTACGGGTCTG--TC-----------------GG
AC-CTGTAGGCCCTCAAAAGCAGTGGCGGGCTCGCCAGGACT-CCGAGCGTAGTAGTTC-TCC
T--CTCGCTCAGGCGGCCT-GGCGTG--CT-TCGGCCGTTAAA---GACCCAAC------TGTAAT-----C
ACAG-AAGCCTTCCGAGTCCCC-TGGAAC------GGGGCGCC-AGAGAGGG-TGAGAGCCCCGT
ATGGTTGGACGCTGAACC-TCTGTAAAGCTCCTTCGACGAGTCGAGTAGTTTGGGAATGCTG
CTCAAAATGGGAGGTAAATTTCTTCTAAAG-CTAAATACCGGCCAGAGACCGATAGCGCACAA
GTAGAGTGATCGAAAGATGAAAAGCACTTTGAAAAGAGGGTTAAATAGCACGTGAAATTGCTG
AAAGGGAAGCGCTTGTGACCAGACTTGCGCCCGGTTGATCATCCAGCGTTCTCGCTGGTGC
ACTCTGCCGGGCTCAGGCCAGCATCGGCTCTCCCAGGGGGATAAAGGTCGCGGGAACGTA
GCTCTCTCCGGGGAGTGTTATAGCCCGTGG-CGGAATGCCCTTAGGGGGGGCCGAGGCCCG
CGTTC-G-CAAGGATGCTGGCGTAATGGTCACCAGCGACCCGTCTTTATCGCTCATGTGTTATG
TGAGCGTCGGAACGCCCGCAGAGCCAATTGTCGAGTTCATGATTGCTAGGAACATGGAGGTA
CTCGAGGAATATGAGCCCTTGAGGTACCCTAATGCAACCAAAATCTTCGTCAACGGCACATGG
GTTGGTGTTCACCAAGACCCGAAGCATCTTGTGAGCTTGGTTCAGGACTTACGCCGCAGGG
GCGTCATCAACTTCGAGGTCTCCCTCATTCGGGATATCCGCGATCGAGAGTTCAAGATTTTCT
CGGATGCTGGTCGTGTTATGCGTCCTCTGTTCGCTGTGGAGCAGTCAGAC---AACTCGACAA
AG---GGCCTTGAAAAGGGCTCTTTATTGCTCACAAAGGAGCATATCCAGAAGTTGAGAGCAGA
CGAGGATCTAACCAAGGATGAAGACGACTATTTCGGCTGGGATGGCCTTCTTAGGGCGGGC
GCCATCGAATATCTTGACGCCGAAGAGGAAGAGACAGCCATGATTTGCATGACTCCAGAAGA
CCTAGAGGCTTATCGGCTGCAAAAAGCTGGATTAGACGTGCCGGAAGACGATGACGAGGCC
G---ACCCCAATAGGCGTCTAAAGACAAAACTAAACCCCACCACTCACATGTATACACACTGCG
AGATCCATCCGAGCATGCTCTTGGGGATCTGCGCGAGCATTATCCCACTCGTGAGCACGCTC
TGCTTGCCTACACCTTGGGTGTGAAGCAGCTCATCGTCGCCATCAACAAGATGGACACCACC
AAGTGGTCTGAGAGCCGTTACAACGAAATTATCAAGGAGACGTCCAACTTCATCAAGAAGGT
CGGCTACAACCCCAAGCAGGTCGCCTTCGTTCCCATCTCCGGCTTCAACGGCGACAACATG
CTTGCCCCCTCCACCAACTGCCCCTGGTACAAGGGTTGGGAGAAGGAGATC---AAGGGCGG
CAAGGCCACTGGCAAGACCCTTCTCGAGGCCATCGATGCTATTGAGCCTCCCAAGCGTCCTA
CGGACAAGCCTCTGCGTCTACCTCTCCAGGATGTTTACAAGATTGGTGGTATTGGAACTGTG
CCCGTCGGCCGTATCGAGACTGGTATCCTCAAGCCCGGTATGGTCGTTACCTTCGCTCCCTC
CAACGTCACCACTGAAGTCAAGTCCGTCGAGATGCACCACGAGCAGCTGGCGGAGGGTGT
CCCCGGCGACAACGTCGGCTTCAACGTGAAGAACGTCTCCGTCAAGGAAATCCGCCGAGG
CAACGTTGCTGGTGACTCCAAGAACGATCCTCCTGCTGGTGCCGCTTCCTTCACCGCCCAG
GTCATCGTCCTCAACCACCCCGGTCAGGTCGGCGCTGGTTACGCTCCCGTCTTGGATTGCC
ACACTGCCCACATCGCTTGCAAGTTCTCTGAGATTCTGGAGAAGATCGACCGCCGAACGGG
CAAGTCTGTCGAGAACAACCCCAAGTTCATCAAGTCTGGTGATGC
>Distoseptispora_yichunensis_HJAUP_C1065
ACCT--------TT-ATGTT-------CGTTGCTTTGGCGGG-CGGCTCC-AGGGCGGAGCCACTGCCCG
TTGGGTCGGGCGCCCGCCGAAGGA---CAAC-CAAAAACTCGTTAAAATCTATACGGATCTCAG
AGTAAAA------TAT-----CAAGCTAT-CAAAACTTTCAGCAACGGATCTCTTGGTTCTGGCATCGAT
GAAGAACGCAGCGAAATGCGATAAGTAATGTGAATTGCAGAATTCAGTGAATCATCGAATCTT
TGAACGCACATTGCGCCCGCCAGCACTCTGGCGGGCATGCCTGTTCGAGCGTCATTTCAA-C

CCTCGGGCCC-TCGTT-------TGGCCTGGTGTTGGGGCGCTACGGGTCTGAACG-----------------
G-AC-CTGTAGGCCCTCAAAATCAGTGGCGGGCTCGCCAGGACT-CCGAGCGTAGTAGTTC-T
CCT--CTCGCTCAGGCGGTCT-GGCGTG--CT-TCGGCCGTTAAA---TACCCAGC------GGTAAT-C
GCACACA---AGCCTTCCGAGTCCCC-TGGAAC------GGGGCGCC-AGAGAGGG-TGAGAGCCC
CGTATGGTTGGACGCCGAACC-TCTGTAAAGCTCCTTCGACGAGTCGAGTAGTTTGGGAATG
CTGCTCAAAATGGGAGGTAAATTTCTTCTAAAG-CTAAATACCGGCCAGAGACCGATAGCGCA
CAAGTAGAGTGATCGAAAGATGAAAAGCACTTTGAAAAGAGGGTCAAATAGCACGTGAAATTG
CTGAAAGGGAAGCGCTTGTGACCAGACTTGCGCCCGGTTGATCATCCAGCGTTCTCGCTGG
TGCACTCTGCCGGGCTCAGGCCAGCATCGGCTCTTCCAGGGGGATAAAGGTCGCGGGAAC
GTATCTCTCTCCGGGGAGTGTTATAGCCCGTGG-CGGAATGCCCTTAGGGGGGGCCGAGGC
CCGCGTTC-G-CAAGGATGCTGGCGTAATGGTCACCAGCGACCCGTCTTTATCGCTCATGTGT
TATGTGAGCGTGGGAACGCCCGCAGAGCCAATCGTCGAGTTCATGATTGCTAGGAACATGGA
AGTACTCGAGGAATATGAGCCCTTGAGGTACCCTAATGCGACCAAAATCTTCGTTAATGGCAC
ATGGGTTGGTGTTCACCAAGACCCAAAGCATCTTGTGAGCTTGGTTCAGGACTTACGCCGCA
GAGGCGTCATCAACTTTGAGGTCTCTCTCATTCGGGATATCCGCGATCGAGAGTTCAAGATTT
TCTCGGATGCTGGTCGTGTTATGCGTCCTCTGTTCGCTGTGGAGCAGTCAGAC---AATTCGAC
AAAG---GGTCTTGAAAAGGGGTCTTTATTGCTCACAAAGGAGCATATTCAAAAATTGAGAGCT
GATGAGGAGTTATCCAAGGACGACCCAGACTATTTTGGCTGGGATGGCCTTCTCAGAGCGGG
CGCTATCGAATATCTTGACGCCGAAGAGGAAGAGACAGCCATGATTTGCATGACTCCAGAAG
ATCTAGAGGCTTATCGGCTGCAAAAAGCTGGATTAGGCGTGCCGGAAGACGATGACGAGGC
CG---ACCCCAATAGGCGTCTGAAGACGAAACTGAACCCCACGACTCACATGTATACACATTGT
GAGATTCATCCGAGCATGCTCTTGGGGATCTGTGCGAGCATTATCCCACTCGTGAGCACGCT
CTGCTCGCCTACACGCTTGGTGTGAAGCAGCTCATCGTCGCCATCAACAAGATGGACACCAC
CAAGTGGTCTGAGAGCCGTTACAACGAAATTATCAAGGAGACCTCCAACTTCATCAAGAAGG
TCGGCTACAACCCCAAGCAGGTCGCCTTCGTTCCCATCTCCGGCTTCAACGGCGACAACAT
GCTTGCCCCCTCCTCTAACTGCCCCTGGTACAAGGGCTGGGAGAAGGAGATC---AAGGGTG
GCAAGGCCACTGGCAAGACCCTCCTCGAGGCTATCGATGCCATTGAGCCTCCCAAGCGTCC
CACAGACAAGCCTCTCCGTCTCCCCCTTCAGGATGTCTACAAGATCGGTGGTATCGGAACTG
TGCCCGTCGGCCGTATCGAGACCGGTATCCTCAAGCCCGGTATGGTCGTCACGTTCGCTCC
CTCCAACGTCACCACTGAAGTCAAGTCCGTCGAGATGCACCACGAGCAGCTGCAGGAGGGT
GTCCCCGGCGACAACGTTGGCTTCAACGTGAAGAACGTCTCCGTCAAGGAAATCCGCCGAG
GCAACGTCGCTGGTGACTCCAAGAACGATCCTCCTGCTGGTGCCGCTTCCTTCACCGCTCA
GGTCATCGTCCTCAACCACCCCGGTCAGGTCGGTGCTGGTTACGCTCCCGTCTTGGATTGC
CACACAGCCCACATTGCTTGCAAGTTCGCTGAGATCCTTGAGAAGATCGACCGCCGAACGG
GCAAGTCTGTTGAGAACAACCCCAAGTTCATCAAGTCTGGTGACGC
>Distoseptispora_nanchangensis_HJAUP_C1074
ACCT--------TTTATACT-------CGTTGCTTTGGCGGG-CGGCTCC-AGGGCGGAGTCACTACCCGT
TGGGTCGGGCGCCCGCCGAAGGA---CAAC-CAAAACTCGCT-AAATCTGT-ACGGATCTCAGA
GTAAAA------TAT-----CAAGCTAT-CAAAACTTTCAGCAACGGATCTCTTGGTTCTGGCATCGATG
AAGAACGCAGCGAAATGCGATAAGTAATGTGAATTGCAGAATTCAGTGAATCATCGAATCTTT
GAACGCACATTGCGCCCGCCAGCACTCTGGCGGGCATGCCTGTTCGAGCGTCATTTCAA-CC
CTCGGGCCC-TCGTT-------TGGTCCGGTGTTGGGGCGCTACGGGTCCG--CC-----------------GG
AC-CTGTAGGCCCTCAAAAGCAGTGGCGGGCTCGCCAGGACT-CCGAGCGTAGTAGTTC-TCC
T--CTCGCTCAGGCGGCCT-GGCGTG--CT-TCGGCCGTTAAAA--GACCCAAC------TGTAAT-----C

ACAG-AAGCCTTCCGAGTCCCC-TGGAAC------GGGGCGCC-AGAGAGGG-TGAGAGCCCCGT
ATGGTTGGACGCCGAACC-TCTGTAAAGCTCCTTCGACGAGTCGAGTAGTTTGGGAATGCTG
CTCAAAATGGGAGGTAAATTTCTTCTAAAG-CTAAATACCGGCCAGAGACCGATAGCGCACAA
GTAGAGTGATCGAAAGATGAAAAGCACTTTGAAAAGAGGGTTAAATAGCACGTGAAATTGCTG
AAAGGGAAGCGCTTGTGACCAGACTTGCGCCCGGTTGATCATCCAGCGTTCTCGCTGGTGC
ACTCTGCCGGGCTCAGGCCAGCATCGGCTCTCCCAGGGGGATAAAGGTCGCGGGAACGTA
GCTCTCTCCGGGGAGTGTTATAGCCCGTGG-CGGAATGCCCTTAGGGGGGGCCGAGGCCCG
CGTTC-G-CAAGGATGCTGGCGTAATGGTCACCAGCGACCCGTCTTTATCGCTCATGTGTTATG
TGAGCGTCGGAACGCCCGCAGAGCCAATTGTCGAGTTCATGATTGCTAGGAACATGGAGGTA
CTCGAGGAATATGAGCCCTTGAGGTACCCTAATGCAACCAAAATCTTCGTCAACGGCACATGG
GTTGGTGTTCACCAAGACCCGAAGCATCTTGTAAGCTTGGTTCAGGATTTACGCCGCAGGGG
CGTCATCAACTTCGAGGTCTCCCTCATTCGGGATATCCGCGATCGAGAGTTCAAGATTTTCTC
GGATGCTGGTCGTGTTATGCGTCCTCTGTTCGCTGTGGAGCAGTCAGAT---AACTCGACAAA
G---GGCCTTGAAAAGGGCTCTTTATTGCTCACAAAGGAGCATATCCAGAAGTTGAGAGCGGA
CGAGGAGCTATCCAAGGATGAAGAAGACTATTTCGGCTGGGATGGTCTTCTTAGGGCGGGC
GCCATCGAATATCTTGACGCTGAAGAGGAAGAGACAGCCATGATTTGCATGACTCCAGAAGA
CCTAGAGGCTTATCGGCTGCAAAAAGCTGGATTAGACGTGCCGGAAGACGATGACGAGGCC
G---ACCCCAATAGGCGTCTAAAGACAAAACTAAACCCCACCACTCACATGTATACACACTGCG
AGATCCATCCGAGCATGCTCTTGGGGATCTGCGCGAGCATTATCCCACTCGTGAGCACGCTC
TGCTTGCCTACACCTTGGGTGTGAAGCAGCTCATCGTTGCCATCAACAAGATGGACACCACC
AAGTGGTCTGAGAGCCGTTACAACGAAATTATCAAGGAGACGTCCAACTTCATCAAGAAGGT
CGGCTACAACCCCAAGCAGGTCGCCTTCGTTCCCATCTCCGGCTTCAACGGCGACAACATG
CTTGCCGCCTCCTCCAACTGCCCCTGGTACAAGGGTTGGGAGAAGGAGGTC---AAGGGCGG
CAAAGCCTCTGGCAAGACCCTTCTCGAGGCCATCGATGCCATTGAGCCTCCCAAGCGTCCTA
CGGACAAGCCTCTGCGTCTCCCTCTCCAGGATGTCTACAAGATTGGTGGTATTGGAACTGTG
CCTGTCGGCCGTATCGAGACTGGTATCCTCAAGCCCGGTATGGTCGTCACATTCGCTCCCTC
CAACGTCACCACTGAAGTCAAGTCCGTCGAGATGCACCACGAGCAGCTGACGGAGGGTGTC
CCCGGCGACAACGTCGGCTTCAACGTGAAGAACGTCTCCGTCAAGGAAATCCGCCGAGGCA
ACGTTGCTGGTGACTCCAAGAACGATCCTCCTGCTGGTGCCGCTTCTTTCACCGCCCAGGT
CATCGTCCTCAACCACCCCGGTCAGGTCGGCGCTGGCTACGCTCCCGTCTTGGATTGCCAC
ACTGCCCACATCGCCTGCAAGTTCTCCGAGATCCTGGAGAAGATCGACCGCCGAACGGGCA
AGTCTGTCGAGAACAACCCCAAGTTCATCAAGTCTGGTGACGC
>Distoseptispora_gasaensis_HJAUP_C2034
ACCT--------GTC--GTT-------CGTTGCTCCGGCGGG-CGGCTCC-AGGGCGGAGCCACTGTCCG
TTAGGTCGGACGCCCGCCGGGGGA---CAAC-CAAAACTCTCC-TGATCTC-GGGCATCGTCAG
AGTATAC----AAAA------CAAGCTAT-CAAAACTTTCAGCAACGGATCTCTTGGTTCTGGCATCGA
TGAAGAACGCAGCGAAATGCGATAAGTAATGTGAATTGCAGAATTCAGTGAATCATCGAATCT
TTGAACGCACATTGCGCCCGCCAGTACTCTGGCGGGCATGCCTGTTCGAGCGTCATTTCAA-
CCCTCGGGCC-CCCGT------TT-GGCCCGGTGTTGGGGTGCTAC--GTCCTGA-------------------C
GGACCCGTAGGCCCTCAAAACCAGTGGCGGGCTCGCCATGGCTTCCGAGCGCAGTAGTTT-
TCTC--CTCGCTCAGGCGGTCT-GGCGCG--CT-CCGGCCGTTAAAA--TACCCA-C------TGTATCT
C----ACAG-AAGCCTTCCGAGTCCCC-TGGAAC------GGGGCGCC-AGAGAGGG-TGAGAGCCC
CGTATGGTCGGACGCCGAACC-TTTGTAAAGCTCCTTCGACGAGTCGAGTAGTTTGGGAATG
CTGCTCAAAATGGGAGGTAAATTTCTTCTAAAG-CTAAATACCGGCCAGAGACCGATAGCGCA

CAAGTAGAGTGATCGAAAGATGAAAAGCACTTTGAAAAGAGGGTTAAATAGCACGTGAAATTG
CTGAAAGGGAAGCGCTTGTGACCAGACTTGCGCCCGGTTGATCATCCAGCGTTCTCGCTGG
TGCACTCTGCCGGGCTCAGGCCAGCATCGGCTCTCCCAGGGGGATAAAGGTCGCGGGAAC
GTAGCTCTCTCCGGGGAGTGTTATAGCCCGTGG-CGGAATACCCTTAGGGGGGGTCGAGGC
CCGCGTTC-G-CAAGGATGCTGGCGTAATGGTCACCAGCGACCCG-CTT?????????????????
???
???
???
???
???
???
???
???
???ACTCGTGAGCATGCCCTG
CTTGCCTACACCCTCGGTGTCAAGCAGCTCATCGTCGCCATCAACAAGATGGACACCACCAA
GTGGTCTGAGGATCGTTACAACGAAATCATCAAGGAGACGTCCAACTTCATCAAGAAGGTCG
GATACAACCCCAAGCAGGTGGCTTTCGTTCCCATCTCCGGCTTCAACGGTGACAACATGTTG
ACCCCCTCCTCCAACTGCCCCTGGTACAAGGGCTGGGAGAAGGAGATT---AAGGGCAACAA
GGTCACTGGCAAGACGCTGCTCGAGGCCATCGATGCTATTGAGACTCCCAAGCGTCCCACC
GACAAGCCTCTGCGCCTGCCCCTTCAGGATGTCTACAAGATCGGTGGTATCGGCACCGTACC
GGTCGGCCGTATCGAGACTGGTGTCCTCAAGCCCGGTATGGTCGTTACATTCGCTCCCTCCA
ACGTCACCACTGAAGTCAAGTCCGTCGAGATGCACCACGAGCAGCTTGCCGAGGGTGTCCC
CGGTGACAACGTCGGCTTCAACGTGAAGAACGTCTCCGTCAAGGAAATCCGCCGTGGCAAC
GTCGCCGGTGACTCCAAGAACGATCCTCCTGCCGGTGCTGCTTCCTTCACCGCCCAGGTCA
TCGTCCTCAACCACCCTGGTCAGGTCGGCGCTGGCTACGCTCCCGTCTTGGATTGCCACAC
TGCCCACATCGCCTGCAAGTTCTCTGAGATCCTCGAGAAGATCGACCGCCGAACGGGCAAG
TCTGTCGAGAACAACCCCAAGTTCATCAAGTCTGGTGACGC
>Distoseptispora_menghaiensis_HJAUP_C2045
ACCT--------TT-TTGTAT------CGTTGCTTCGGCGGG-CGGCTCC-AGGGCGGAGTCACTGCTCG
TTGGGTCGGGTGCCCGCCGGAGGA---CAAC-CAAAAACTCAT-TA-TCTCG-GGTAACCTCAGA
GTATTATT---ATAC-----AA-GCTAT-CAAAACTTTCAGCAACGGATCTCTTGGTTCTGGCATCGATG
AAGAACGCAGCGAAATGCGATAAGTAATGTGAATTGCAGAATTCAGTGAATCATCGAATCTTT
GAACGCACATTGCGCCCGCTAGTATTCTGGCGGGCATGCCTGTTCGAGCGTCATTTCAA-CC
CTCGGGCCC-CCGTT-------TGGCCCGGTGTTGGGGTGCTACGGTCC-G--C------------------CGG
AC-CCGTAGGCCCTCAAAACCAGTGGCGGGCTCTCCAGTACC-CCGAGCGCAGTAGTTT-TCT
C--CTCGCTCAGGCGGCCT-GGCGTG--CT-TCGGCCGTTAAA---TACCCA-C------TGT-TT----ACA
CAG-AAGCCTTCCGAGTCCCC-TGGAAC------GGGGCGCC-AGAGAGGG-TGAGAGCCCCGTA
TGGTTGGACGCCGAACC-TCTGTAAAGCTCCTTCGACGAGTCGAGTAGTTTGGGAATGCTGC
TCAAAATGGGAGGTAAATGTCTTCTAAAG-CTAAATACCGGCCAGAGACCGATAGCGCACAAG
TAGAGTGATCGAAAGATGAAAAGCACTTTGAAAAGAGGGTTAAATAGCACGTGAAATTGCTGA
AAGGGAAGCGCTTGTGACCAGACTTGTGCCCGGTTGATCATCCAGCGTTCTCGCTGGTGCA
CTCTGCCGGGCTCAGGCCAGCATCGGTTCTCCCAGGGGGATAAAGGTCGCGGGAACGTAG
CTCTCTCCGGGGAGTGTTATAGCCCGCGG-CGGAATGCCCTTAGGGGGGACCGAGGCCCGC
GTTC-G-CAAGGATGCTGGCGTAATGGTCACCAGCGACCCG-TCT???????????????????????

??
??
??
??
??
??
??
??
??
??
??
??
??
??
??
??
??
??
???
>Distoseptispora_jinghongensis_HJAUP_C2120
ACCT--------TT-TTGT-T------CGTCGCTTCGGCGGG-CGGCTCC-AGGGCGGAGCCACTGCTCG
TTGGGTCGGGTGCCCGCCGAAGGA---CAGC-CAAAACTCTTT-GAATTTAG-CGTATCATCAGA
GTATTA-----AAAA-----AAAGCTAT-CAAAACTTTCAGCAACGGATCTCTTGGTTCTGGCATCGAT
GAAGAACGCAGCGAAATGCGATAAGTAATGTGAATTGCAGAATTCAGTGAATCATCGAATCTT
TGAACGCACATTGCGCCCGCCAGCACTCTGGCGGGCATGCCTGTTCGAGCGTCATTTCAA-C
CCTCGGGCCC-TTGTT-------TGGCCCGGTGTTGGGGTGCTACGGTCCGA--TT-----------------CG
GAC-CTGTAGGCCCTCAAAAGCAGTGGCGGGCTCGCCAGGGCT-CCGAGCGCAGTAGTTT-T
CCT--CTCGCTCAGGTGGCCT-GGCGTG--CT-TCGGCCGTTAAAA--CACCCA-C------TGTATT----
-CACAG-AAGCCTTCCGAGTCCCC-TGGAAC------GGGGCGCC-AGAGAGGG-TGAGAGCCCC
GTATGGTTGGACGCCGAACC-TCTGTAAAGCTCCTTCGACGAGTCGAGTAGTTTGGGAATGCT
GCTCAAAATGGGAGGTAAATTTCTTCTAAAG-CTAAATACCGGCCAGAGACCGATAGCGCACA
AGTAGAGTGATCGAAAGATGAAAAGCACTTTGAAAAGAGGGTTAAATAGCACGTGAAATTGCT
GAAAGGGAAGCGCTTGTGACCAGACTTGCGCCCGGTTGATCATCCAGCGTTCTCGCTGGTG
CACTCTGCCGGGCTCAGGCCAGCATCGGCTCTCCCAGGGGGATAAAGGTCACGGAACGTA
GCTCTCTTCGGGGAGTGTTATAGCCCGCGG-CGGAATACCCTTAGGGGGGGCCGAGGCCCG
CGTTC-G-CAAGGATGCTGGCGTAATGGTCACCAGCGACCCGTCTT?????????????????????
??
??
??
??
??
??
??
??

??ACCCGTGAGCACGCTCTGCTT
GCCTACACCCTGGGTGTGAAGCAGCTCATCGTCGCCATCAACAAGATGGACCCCACCAAGT
GGTCCGAGGATCGGTACAACGAAATCATCAAGGAGACGTCCAACTTCATCAAGAAGGTCGGC
TACAACCCCAAGCAGGTCGCTTTCGTCCCCATCTCGGGCTTCAACGGCGACAACATGCTTTC
TCCTTCCACCAACTGCCCCTGGTACAAGGGCTGGGAGAAGGAGGTCGGCAAGGGCACCAA
GGTTACTGGCAAGACCCTCCTCGAGGCCATCGATGCCATTGAGCCTCCCAAGCGCCCCACA
GACAAGCCTCTGCGTCTCCCCCTCCAGGATGTCTACAAGATCGGTGGTATCGGGACTGTACC
TGTCGGCCGTATCGAGACTGGTGTCCTCAAGCCCGGTATGGTCGTCACGTTCGCCCCGTCC
AACGTCACCACTGAAGTCAAGTCCGTCGAGATGCACCACGAGCAGCTGGCTGAGGGTGTTC
CTGGCGACAACGTTGGCTTCAACGTGAAGAACGTCTCCGTCAAGGAGATTCGCCGAGGCAA
CGTCGCTGGTGACTCCAAGAACGACCCTCCCGCTGGTGCCGCTTCTTTCACCGCCCAGGTC
ATCGTTCTGAACCACCCCGGTCAGGTCGGTGCTGGCTACGCTCCCGTCTTGGATTGCCACA
CTGCCCACATCGCTTGCCAGTTCTCTGAGATCCTCGAGAAGATCGACCGCCGAACGGGCCA
GTCCGTCGAGAACAACCCCAGGTTCATCGAGTCCGGTGACGC
>Distoseptispora_menglunensis_HJAUP_C2170
ACCT--------CT-ATCTT-------CGTTGCTTTGGCGGG-CGGCTCC-AGGGCGGAGCCACTGCTCGT
TGGGTCGGGCGCCCGCCGAAGGA---CAAC-CAAAACTCGTTTGAACCTTT-GCGGATCTCAGA
GTAAAAA-----TAT-----CAAGCTAT-TAAAACTTTCAGCAACGGATCTCTTGGTTCTGGCATCGATG
AAGAACGCAGCGAAATGCGATAAGTAATGTGAATTGCAGAATTCAGTGAATCATCGAATCTTT
GAACGCACATTGCGCCCGCCAGCACTCTGGCGGGCATGCCTGTTCGAGCGTCATTTCAA-CC
CTCAGGCCC-TCGTT-------TGGCCTGGTGTTGGGGCGCTACGGGTCTCAAAC------------------A
GAC-CTGTAGGCCCTTAAAATCAGTGGCGGGCTCGCCAGGACT-CCGAGCGTAGTAGTTC-TC
CT--CTCGCTCAGGCGGTCT-GGCGTG--CT-TCGGCCGTTAAA---TAACCAGC------GGTAAT-CG-
-CACAC-AAGCCTTCCGAGTCCCC-TGGAAC------GGGGCGCC-AGAGAGGG-TGAGAGCCCC
GTATGGTCGGACGCCGAACC-TCTGTAAAGCTCCTTCGACGAGTCGAGTAGTTTGGGAATGC
TGCTCAAAATGGGAGGTAAATTTCTTCTAAAG-CTAAATACCGGCCAGAGACCGATAGCGCAC
AAGTAGAGTGATCGAAAGATGAAAAGCACTTTGAAAAGAGGGTCAAATAGCACGTGAAATTGC
TGAAAGGGAAGCGCTTGTGACCAGACTTGCGCCCGGTTGATCATCCAGCGTTCTCGCTGGT
GCACTCTGCCGGGCTCAGGCCAGCATCGGCTCTCCCAGGGGGATAAAGGTCGCGGGAACG
TAGCTCTCTCCGGGGAGTGTTATAGCCCGTGG-CGGAATGCCCTTAGGGGGGGCCGAGGCC
CGCGTTC-G-CAAGGATGCTGGCGTAATGGTCACCAGCGACCCGTCTTTATCGCTCATGTGTT
ATGTGAGCGTGGGAACGCCCGCAGAGCCAATCGTCGAGTTCATGATTGCTAGGAACATGGAA
GTACTCGAGGAATATGAGCCCTTGAGGTACCCTAATGCGACCAAAATCTTCGTCAATGGCACA
TGGGTTGGTGTTCACCAAGACCCGAAGCATCTTGTGAGCTTGGTTCAGGACTTACGCCGCA
GAGGCGTCATCAACTTTGAGGTCTCTCTCATTCGGGATATCCGCGATCGAGAGTTCAAGATTT
TCTCGGATGCTGGTCGTGTTATGCGTCCTCTGTTCGCTGTGGAGCAGTCGGAC---AACTCGA
CAAAG---GGTCTTGAAAAGGGATCTTTATTGCTCACAAAGGAGCACATTCAAAAATTGAGAGC
TGATGAGGAGTTGTCCAAGGATGACCCAGATTATTTCGGCTGGGACGGCCTTCTCAGAGCGG
GCGCTATCGAATATCTTGACGCCGAAGAGGAAGAGACAGCCATGATTTGCATGACTCCAGAA
GATCTAGAGGCTTATCGGCTGCAAAAAGCCGGATTAGACGTGCCGGAAGACGATGACGAGG
CCG---ACCCCAATAGGCGTCTGAAGACGAAACTGAACCCCACGACTCACATGTATACACATTG
TGAGATCCATCCGAGCATGCTCTTGGGGATCTGTGCGAGCATTATCCCACTCGTGAGCACGC
TCTGCTCGCCTACACGCTGGGTGTGAAGCAGCTCATCGTCGCCATCAACAAGATGGACACCA
CCAAGTGGTCTGAGAGCCGTTACAACGAAATTATCAAGGAGACGTCCAACTTCATCAAGAAG

GTCGGCTACAACCCCAAGCAGGTCGCCTTCGTTCCCATCTCCGGCTTCAACGGCGACAACA
TGCTTGCCCCCTCCTCCAACTGCCCCTGGTACAAGGGCTGGGAGAAGGAGATC---AAGGGT
GGCAAGGCCACTGGCAAGACCCTCCTCGAGGCCATCGATGCCATTGAGCCTCCCAAGCGCC
CCACAGACAAGCCTCTCCGTCTCCCCCTTCAGGATGTCTACAAGATCGGTGGTATCGGAACT
GTGCCCGTCGGCCGTATCGAGACTGGTATCCTCAAGCCCGGCATGGTCGTCACTTTCGCTC
CCTCCAACGTCACCACTGAAGTCAAGTCCGTCGAGATGCACCACGAGCAGCTGGCGGAGG
GTGTCCCCGGCGACAACGTTGGCTTCAACGTGAAGAACGTCTCCGTCAAAGAGATCCGCCG
AGGCAACGTCGCTGGTGACTCCAAGAACGATCCTCCTGCTGGTGCCGCTTCCTTCACCGCT
CAGGTCATCGTCCTCAACCACCCCGGTCAGGTCGGTGCTGGTTACGCTCCCGTCTTGGATT
GCCACACAGCCCACATTGCTTGCAAGTTCTCTGAGATCCTCGAGAAGATCGACCGCCGAAC
GGGCAAGTCTGTTGAGAACAACCCCAAGTTCATCAAGTCTGGTGACGC
>Acrodictys_bambusicola_CGMCC_3_18641
ACCT--------GT-CTC----------GTTGCTTCGGCGGG-CGGCCCC-AGGGCGGGGCCAGTGGCCGC
AAGGT-----GCCCGCCGAAGAA---GCCT---ACAACTC----GATTTTAGTGGTC-TTCAGAATACT----
--GTATTA-A-AA--TACATCAAAACTTTCAACAACGGATCTCTTGGTTCCAGCATCGATGAAGAAC
GCAGCGAAATGCGATACGTAATGTGAATTGCAGAATTCCGTGAATCATCGAATCTTTGAACGC
ACATTGCGCCCGCTAGTATTCTGGCGGGCATGCCTGTTCGAGCGTCATTTCG-ACCCTCAGG
CCCCA----------GTGGCCTGGTGTTGGAGT---TCTAC------------------------AGCAGCT-GTAGACCC
CGAAATGCAGTGGCGGGCTCGCCTTGTTGCCTGAGCGTAGTAGTTATC--CTTCTCGTTCAGG
GCGTCA-GGCGTG--CG-CTGGCCGTTA-------------------------------------GCCTTCTGAGTTCCC-T
GGAAC------GGGACGCC-AGAGAGGG-TGAGAGCCCCGTAGAGTTGGACGCCTAACC-TCTGT
AAAGCTCCTTCGACGAGTCGAGTAGTTTGGGAATGCTGCTCAAAATGGGAGGTAAATTTCTTC
TAAAG-CTAAATACCGGCTGGAGACCGATAGCGCACAAGTAGAGTGATCGAAAGATGAAAAGC
ACTTTGAAAAGAGGGTTAAACAGCACGTGAAATTGTTGAAAGGGAAGCGCTTGTGACCAGAC
TTGTGCCTTGCCAATCATCCGGCGTTCTCGCCGGTGCACTTGGCTTGGCCCAGGCCAGCAT
CAGTTTTCTCAGGGGGAGAAAAGCGCCGGGAACGTAGCTCCCTC--GGGAGTGTTATAGCCC
GGCG-CACAATACCTCTAGG-AGGACTGAGGACCGCGCATTG-CAAGGATGCTGGCGTAATGG
TCATCAGCGACCCGTCTT??
??
??
??
??
??
??
??
??
??
??
??
??
??
??
??
??

??
??
???
>Acrodictys_elaeidicola_CGMCC_3_18642
ACCT--------GTATTC----------GTCGCTTCGGCGGG-CGGCCCC-GGGGAGGGGCCGGTGGCCG
CAAGGC-----GCCCGCCGGGGGA---CAGC---AAAGCTC----GTTTTTAGTGGTC-TTCAGAGTAC
CC-----GTATGC-A-AA--TACATCAAAACTTTCAACAACGGATCTCTTGGTTCCAGCATCGATGAA
GAACGCAGCGAAATGCGATACGTAATGTGAATTGCAGAATTCCGTGAATCATCGAATCTTTGA
ACGCACATTGCGCCCGCCAGCATTCTGGCGGGCATGCCTGTTCGAGCGTCATTTCG-ACCCT
CAGGCCCCC---------CGGGGCCTGGTGTTGGGGCATCTCTAC-------------------------AGCGTCT-G
TAGGCCCTGAAATGCAGTGGCGGGCTCGCCGCGTCGCCTGAGCGCAGTAG--ACT--TGTCTC
GCTCGGGGCGCCC-GGCAAG--CG-CTAGCCGTGA---------------------------------------GCCTTCTGA
GTTCCC-TGGAAC------GGGACGCC-ATAGAGGG-TGAGAGCCCCGTAGAGTTGGACGCCTAG
CC-TCTGTAAAGCTCCTTCGACGAGTCGAGTAGTTTGGGAATGCTGCTCAAAATGGGAGGTAA
ATTTCTTCTAAAG-CTAAATACCGGCTGGAGACCGATAGCGCACAAGTAGAGTGATCGAAAGAT
GAAAAGCACTTTGAAAAGAGGGTTAAACAGCACGTGAAATTGTTGAAAGGGAAGCGCTCGTG
ACCAGACTTGCGCCCTGCCAATCATCCGGCGTTCTCGCCGGTGCACTTGGCTGGGCCCAGG
CCAGCATCGGTTTCCCCAGGGGGAGAAAAGCGCCGGGAACGTAGCTCCCCC--GGGAGTGT
TATAGCCCGGCG-CACAATGCCTCTGGG-GGGACCGAGGACCGCGCATCG-CAAGGATGCTG
GCGTAATGGTCACCAGCGACCCGTCTT???
??
??
??
??
??
??
??
??
??
??
??
??
??
??
??
??
??
??
??
>Aquapteridospora_aquatica_MFLUCC_17_2371
AACC-ACTGTGAATCATACCAAGCCGTTGCCTCTTCGGCGGGCGGTCCCCTGGGAGGGGAC
CACTGTCTCTTCGCTGAGGCGCCCGCCGGGG----GCAGA--ATAAACTCTCGTATCTCT-AGTG
GATCTCTGAGCACTTGG-----------AAAAAATAATCACAACTTTCAACAACGGATCTCTTGGCTCT
GGCATCGATGAAGAACGCAGCGAAATGCGATACGTAATGCGAATTGCAGAATTCAGCGAGTC

ATCGAATCTTTGAACGCACATTGCGCCCGCTAGCACTCTAGCGGGCACGCCTGTCCGAGCGT
CATTTCAACCCCTCAGGCCTCGCTTG-----CGTTGCCTGGCGTTGGGGTCCTGCGTCGC--------
------------------AAGCCGCAGGCCCTGAAAATTAGTGGCGGGCCTACCTGGAAGCCCTGGCGTA
GTAGTTA----CCTCTCGCCTGGGGAGCCACCGTAGGTTCTTCCTGCCGTAAAACCCCTCTAAA
A-GCCTGCGAACTCTCGCAGCAGAA-GCCTACCAAGTCCAC-TGGAAC------GTGGCGCC-ACA
GAGGG-TGAGAGCCCCGTACTGTAGGACCCCGAGCC-TCTGTGAAGCTCCTCCGACGAGTC
GAGTAGTTTGGGAATGCTGCTCTAAATGGGAGGTAAATTTCTCCTAAAG-CTAAATACCGGCCA
GAGACCGATAGCGCACAAGTAGAGTGATCGAAAGATGAAAAGCACTTTGAAAAGAGGGTTAA
ACAGCACGTGAAATTGTTGAAAGGGAAGCGCCTGTGACCAGACTCGTGCCCGGCGGATCAT
CCAGCCTTCTGGCTGGTGCACTGCGCCGGGTCCGGGCCAGCATCGGCTTTCCCTGGGGGA
TAAAGGCCCTGGGAACGTAGCTCTCTCCGGGGAGTGTTATAGCCCATCG-CACAATGCC-TCA
GGGGGGGCCGAGGACCGCGCTTCGGCAAGGATGCTGGCGTAATGGTCATCAGCGACCCGT
CTT??
???
???
???
???
???
???
???
???
???
???
???
???
???
???
???
???
???
???
?????????????????????????
>Aquapteridospora_fusiformis_MFLUCC_18_1606
AACCCACTGTGAACCCTACCAAGTCGTTGCCTCTACGGCGGGCGGTCCCCTGGGAGGGGA
CCAGTGCCTCTTCGCTGAGGCGCCCGCCGGGGCACAACAGC--CTAAACCCTTCTGTATCTC
AGCGCATCTCTGAGCACTAAA-----------GAAAATAAATCACAACTTTCAACAACGGATCTCTTGG
CTCTGGCATCGATGAAGAACGCAGCGAAATGCGATACGTAATGCGAATTGCAGAATTCAGCG
AGTCATCGAATCTTTGAACGCACATTGCGCCCGCTAGCACTCTAGCGGGCACGCCTGTCCGA
GCGTCATTTCAACCCCTCAGGCCTCCTCCG-----CGCTGCCTGGCGTTGGGGCCCTGCGGCG
C-------------------------GAGCCGCAGGCCCTGAAAATCAGTGGCGGGCCTACCTGGAAGCCCTG
GCGTAGTAGTTC----TCTCTCGCCTGGGA--CCCCAGGGG---CCTCCTGCCGTAAAACCCCCCA
CCAAAGCCTGCGAGCACTCGCAGCGGAAAGCCTATCAAGTCCAC-TGGAAC------GTGGCGC
C-ATAGAGGG-TGAGAGCCCCGTACGGTAGGACCCCGAGCC-TCTGTGAAGCTCCTCCGACG
AGTCGAGTAGTTTGGGAATGCTGCTCTAAATGGGAGGTAAATTTCTTCTAAAG-CTAAATACCG

GCCAGAGACCGATAGCGCACAAGTAGAGTGATCGAAAGATGAAAAGCACTTTGAAAAGAGG
GTTAAACAGCACGTGAAATTGTTGAAAGGGAAGCGCCTGTGACCAGACTCGTGCCCGGCGG
ATCATCCAGCCTTCTGGCTGGTGCACTGCGCCGGGTCCGGGCCAGCATCGGCTCTCCCTGG
GGGATAAAGGCCCTGGGAACGTAGCTCTCTCCGGGGAGTGTTATAGCCCATCG-CACAATGC
C-TCAGGGGGGGCCGAGGACCGCGCTTCGGCTAGGATGCTGGCGTAATGGTCATCAGCGAC
CCGTCTT???
??
??
??
??
??
??
??
??
???ACCCGTGAGCACGCTCTGCTCGCCTACACGCTTGGTGTCCGGCAGATCATTGTCGCCAT
CAACAAGATGGACACGACCAAGTGGTCCGAGGATCGTTACAACGAAATCATCAAGGAGACGT
CCAACTTCATCAAGAAGGTCGGCTTCAACCCCAAGCAGATCGCGTTTGTGCCCATCTCTGGC
TTCAACGGCGACAACATGCTGGCGGCCTCCACCAACTGCCCCTGGTACAAGGGATGGGAGA
AGGAGG---TCAAGGGCGGCAAGGTTACTGGCAAGACGCTCCTCGAGGCCATCGATGCCATC
GAGCCCCCCCGCCGTCCCACTGACAAGCCCCTCCGTCTCCCTCTCCAGGACGTCTACAAGA
TCGGCGGTATTGGAACTGTACCGGTCGGCCGTATCGAGACCGGTGTCCTCAAGCCCGGTAT
GGTCGTTACCTTCGCTCCCTCCAACGTCACCACTGAAGTCAAGTCGGTCGAGATGCACCAC
GAGCAGCTTACCGAAGGCCAGCCGGGCGACAACGTCGGCTTCAACGTGAAGAACGTGTCC
GTCAAGGAAATCCGCCGTGGCAACGTTGCTGGCGACACCAAGAACGACCCCCCCGCTGGC
GCCGCCTCCTTCACTGCCCAGGTCATCGTCCTGAACCACCCCGGCCAGGTTGGTGCTGGGT
ACGCTCCCGTGCTGGATTGCCACACTGCCCACATTGCCTGCAAGTTCGCCGAGATCACGGA
GAAGATCGACCGCCGAACTGGTAAGTCGGTTGAGAACAACCCCAAGTTCATCAAGTCTGGTG
ACGC
>Aquapteridospora_lignicola_MFLUCC_15_0377
??
??
??
??
??
??
??
????????GCCTACCAAGTCCAC-TGGAAC------GTGGCGCC-ACAGAGGG-TGAGAGCCCCGTA
CTGTAGGACCCCGAGCC-TCTGTGAAGCTCCTCCGACGAGTCGAGTAGTTTGGGAATGCTGC
TCTAAATGGGAGGTAAATTTCTCCTAAAG-CTAAATACCGGCCAGAGACCGATAGCGCACAAGT
AGAGTGATCGAAAGATGAAAAGCACTTTGAAAAGAGGGTTAAACAGCACGTGAAATTGTTGA
AAGGGAAGCGCCTGTGACCAGACTCGTGCCCGGCGGATCATCCAGCCTTCTGGCTGGTGCA
CTGCGCCGGGTCCGGGCCAGCATCGGCTCTCCCTGGGGGATAAAGGCCCTGGGAACGTAG
CTCTCTCCGGGGAGTGTTATAGCCCATCG-CACAATGCC-TCAGGGGGGGCCGAGGACCGCG
CTTCGGCAAGGATGCTGGCGTAATGGTCATCAGCGACCCGTCTT?????????????????????

??
??
??
??
??
??
??
??
??
??
??
??
??
??
??
??
??
??
??
>Bullimyces_aurisporus_AF316_1b
??
??
??
??
??
??
??
????????GCCTACTGAGTTCCC-TGGAAC------GGGACGCC-ATAGAGGG-TGAGAGCCCCGTA
CAGTTGGACGCCGAGCC-TCTGTAATGCTCCTTCGACGAGTCGAGTAGTTTGGGAATGCTGC
TCAAAATGGGAGGTAAATTTCTTCTAAAG-CTAAATACCGGCCAGAGACCGATAGCGCACAAGT
AGAGTGATCGAAAGATGAAAAGCACTTTGAAAAGAGGGTTAAACAGCACGTGAAATTGTTGA
AAGGGAAGCGCTTATGACCAGACTTGTGCCTTGTGAATCATCCAGCGTTCTCGCTGGTGCAC
TTCGCCTGGTTCAGGCCAGCATCGGTTCTCTTCGGGGGATAAAGGCTCTGGGAATGTAGCTC
CTCC--GGGAGTGTTATAGCCCACTG-CATAATACCCTTAAG-GGGACCGAGGTTCGCGCTCTG-
CAAGGATGCTGGCGTAATGGTCATCAGCGACCCGTCTT???????????????????????????????
??
??
??
??
??
??
??
??
??

???
???
???
???
???
???
???
???
???
??
>Bullimyces_communis_AF281_5
???
???
???
???
???
???
???
????????GCCTTCTGAGTTCCC-TGGAAC------GGGACGCC-ATAGAGGG-TGAGAGCCCCGTAT
AGTTGGACGCCGAGCC-TCTGTAATGCTCCTTCGACGAGTCGAGTAGTTTGGGAATGCTGCT
CAAAATGGGAGGTAAATTTCTTCTAAAG-CTAAATACCGGCCAGAGACCGATAGCGCACAAGT
AGAGTGATCGAAAGATGAAAAGCACTTTGAAAAGAGGGTTAAACAGCACGTGAAATTGTTGA
AAGGGAAGCGCTTATGACCAGACTTGTGCCTTGTGAATCATCCAGCGTTCTCGCTGGTGCAC
TTCGCTTGGTTCAGGCCAGCATCGGTTCTCTTCGGGGGATAAAGGCTCTGGGAATGTAGCTC
CTCC--GGGAGTGTTATAGCCCACTG-CATAATACCCTTAGG-AGGACCGAGGTTCGCGCTCTG-
CAAGGATGCTGGCGTAATGGTCATCAGCGACCCGTCTT????????????????????????????????
???
???
???
???
???
???
???
???
???
???
???
???
???
???
???
???
???
???

???
>Cancellidium_applanatum_CBS_337_76
ACCT--------GT-ATC----------GTTGCTTCGGCAGG-CGGCCCC-AGGGCGGGGCCACCGCCTGTT
AGGTCAGGTGCCTGCCGAAGGA---TACC---AAAACTCTTTATATTTTAGTT----ATCTGAGTCTTA-
----CAAGAC-ACAA--TTAGTTAAAACTTTCAACAACGGATCTCTTGGTTCTGGCATCGATGAAGA
ACGCAGCGAAATGCGATAAGTAATGTGAATTGCAGAATTCAGTGAATCATCGAATCTTTGAAC
GCACATTGCGCCTGCCAGTATTCTGGCAGGCATGCCTGTTCGAGCGTCATTTCA-ACCCTCAG
GCTTAG--CTTTTTGCTGGGCCTGGCGTTGGGGCATCTGCTT------------------------GGTTTCAAG
CAGGCCCTCAAGACCAGTGGCGGGCTCGCTGTGAC-CCCGAGCGTAGTAGTTATT-CTTTCTC
GCTTTGGGTGTCCCGGTGGG--CTTCCTGCCATCAAACC-TCTC--TT------TTTTTACT-------GAA
AGCCTTCTGAGTTCCC-TGGAAC------GGGACGCC-AGAGAGGG-TGAGAGCCCCGTATAGTTG
GACACCAAGCC-TCTGTAAAGCTCCTTCGACGAGTCGAGTAGTTTGGGAATGCTGCTCAAAAT
GGGAGGTAAATTTCTTCTAAAG-CTAAATACCGGCCAGAGACCGATAGCGCACAAGTAGAGTG
ATCGAAAGATGAAAAGCACTTTGAAAAGAGGGTTAAACAGCACGTGAAATTGTTGAAAGGGA
AGCGCTTGTGACCAGACTTGCGCCTTGCGAATCATCCAGCGTTCTCGCTGGTGCACTTCGCT
TGGCTCAGGCCAGCATCGGTTTTCCCAGGGGGATAAAGGCGTCGGGAACGTAGCTCTCTAC
GGGGAGTGTTATAGCCCGGCG-TACAATATCCTTGGG-GGGACCGAGGTTCGCGCTCTG-CAA
GGATGCTGGCGTAATGGTCATCAGCGACCCGTCTT??????????????????????????????????
???
???
???
???
???
???
???
???
???
???
???
???
???
???
???
???
???
???
???
>Cancellidium_cinereum_MFLUCC_18_0424
ACCT--------GT-ATC----------GTTGCTTCGGCAGG-CGGCCCC-AGGGCGGGGCCACCGCCTGTT
AGGTCAGGTGCCTGCCGAAGGA---TACC---CAAACTCTTTATATTTTAGTTGTCTATCTGAGTCT
CA-----CAAGAC-AAAA--TAAGTCAAAACTTTCAACAACGGATCTCTTGGTTCTGGCATCGATGA
AGAACGCAGCGAAATGCGATAAGTAATGTGAATTGCAGAATTCAGTGAATCATCGAATCTTTG
AACGCACATTGCGCCTGCCAGTATTCTGGCAGGCATGCCTGTTCGAGCGTCATTTCA-ACCCT
CAGGCCCTGGCTTTTTTGCTGGGCCTGGCGTTGGGGCATCTGC-------------------------GGTTT

GAAGCAGGCCCTCAAGACCAGTGGCGGGCTCGCTGTGAC-CCCGAGCGTAGTAGTTATTTCT
TTCTCGCTTTGGGTGTCCCGGTGGG--CTTCCTGCCATCAAACC-TCTTCTTT------TTTTTACT---
----GAAAGCCTTCTGAGTTCCC-TGGAAC------GGGACGCC-AGAGAGGG-TGAGAGCCCCGTAT
AGTTGGACACCAAGCC-TCTGTAAAGCTCCTTCGACGAGTCGAGTAGTTTGGGAATGCTGCT
CAAAATGGGAGGTAAATTTCTTCTAAAG-CTAAATACCGGCCAGAGACCGATAGCGCACAAGT
AGAGTGATCGAAAGATGAAAAGCACTTTGAAAAGAGGGTTAAACAGCACGTGAAATTGTTGA
AAGGGAAGCGCTTGTGACCAGACTTGCGCCAGGCGAATCATCCAGCGTTCTCGCTGGTGCA
CTTCGCCTGGCTCAGGCCAGCATCGGTTTTCCCAGGGGGATAAAGGCGCCGGGAACGTGG
CTCTCTCCGGGGAGTGTTATAGCCCGGTG-TACAATATCCTTGGG-GGGACCGAGGTTCGCG
CTCTG-CAAGGATGCTGGCGTAATGGTCATCAGCGACCCGTCTTTCTCTCTGATGTGCTATGT
GAGTGTGGGGACGCCCGCAGAGCCCATCGTGGAGTTCATGATTGCCCGAAATATGGAGGTC
CTGGAAGAGTACGAGCCGCTTCGCTACCCGAACGCGACGAAGATCTTTGTCAACGGAACCT
GGGTTGGCGTCCACCAGGATCCAAAGCATCTTGTCAGCCTTGTCCAGGGCCTTAGACGGAG
AGGCGTGATTCAAGGCGAGGTGTCGCTGATCCGCGATATCCGAGACCGGGAATTCAAGATCT
TCTCTGACGCTGGTCGTGTCATGAGGCCCCTCTTCGCCGTAGAACAGGAGGAC---AACCCG
GAGACG---GGCCAGTCCAGTGGCAACCTGGTGCTCTCGAAGGAACATATCAAGAGGTTGGA
GAACGATCGGGACATTGGTAAAGACAACCCCGGCTACTTTGGATGGCAAGGTCTCTGCGAC
GCCGGAGCTATAGAATACCTTGACGCCGAAGAGGAGGAGACTGCCATGATTTGCATGACGCC
CGAAGACCTGGAGAATTACAGGCTGCAGAAGGCTGGATACGAAGTACCAGATGATTACGGAG
ATGAGG---ATCTCAACCGCCGCCTGAAGACGAAGATGAATCCTACTACGCACAAGTACACCCA
CTGCGAGATTCATCCGAGCATGCTTCTTGGTATCTGTGCCAGCATTATCCCACTCGTGAGCAC
GCTCTGCTCGCCTACACCCTGGGTGTCAAGCAGCTCATTGTCGCGATCAACAAGATGGACAC
CACCAAGTGGTCTGAGGATCGTTACAACGAGATCATCAAGGAGACGTCCAACTTCATCAAGA
AGGTCGGCTACAACCCCAAGCAGGTCGCCTTTGTCCCCATCTCCGGCTTCAACGGCGACAA
CATGCTTGCCCCCTCGACCAACTGCCCCTGGTACAAGGGCTGGAAGAAGGAGA---CCAAGG
GTGGTGAGGTCAGCGGCAAGACCCTCCTCGAGGCCATCGACGCCATCGAGCCCCCCAAGC
GTCCCACCGACAAGCCCCTCCGTCTCCCGCTCCAGGATGTCTACAAGATTGGCGGTATCGG
AACTGTGCCCGTCGGCCGTATCGAGACTGGTATCCTCAAGCCCGGTATGGTCGTCACTTTCG
CTCCCTCCAACGTCACCACTGAAGTCAAGTCCGTCGAGATGCACCACGAGCAGCTTACCGA
GGGTGTTCCCGGCGACAACGTCGGCTTCAACGTGAAGAACGTCTCCGTCAAGGAAATCCGC
CGTGGCAACGTCGCTGGCGACTCCAAGAACGACCCCCCGGCTGGTGCTGCCTCCTTCACC
GCCCAGGTCATCGTCCTCAACCACCCCGGCCAGGTCGGTGCTGGTTACGCCCCTGTCTTGG
ATTGCCACACTGCCCATATCGCCTGCAAGTTCGCCGAGATCCTTGAGAAGATCGACCGCCGT
ACCGGCAAGTCGGTCGAGAACAACCCCAAGTTCATCAAGTCCGGTGACGC
>Distoseptispora_adscendens_HKUCC_10820
???
???
???
???
???
???
???
????????GCCTTCCGAGTCCCC-TGGAAC------GGGACGCC-AGAGAGGG-TGAGAGCCCCGTA
TGGTCGGACGCCGAACC-TCTGTAAAGCTCCTTCGACGAGTCGAGTAGTTTGGGAATGCTGC

TCAAAATGGGAGGTAAATTTCTTCTAAAG-CTAAATACCGGCCAGAGACCGATAGCGCACAAGT
AGAGTGATCGAAAGATGAAAAGCACTTTGAAAAGAGGGTTAAATAGCACGTGAAATTGCTGAA
AGGGAAGCGCTTGTGACCAGACTTGCGCCCGGTTGATCATCCAGCGTTCTCGCTGGTGCAC
TCTGCCGGGCTCAGGCCAGCATCGGCTCTCCCAGGGGGATAAAGGTCGCGGGAACGTAGC
TCTCTCCGGGGAGTGTTATAGCCCGTGG-CGGAATGCCCTTAGGGGGGGCCGAGGCCCGCG
TTC-G-CAAGGATGCTGGCGTAATGGTCACCAGCGACCCGTCTTTATCGCTCATGTGTTATGTG
AGCGTCGGAACACCCGCAGAGCCAATTGTCGAGTTCATGATTGCTAGGAACATGGAAGTACT
CGAGGAATATGAGCCCTTGAGGTACCCTAATGCAACCAAAATCTTCGTCAACGGCACATGGG
TTGGTGTTCACCAAGACCCGAAGCATCTTGTGAGCTTGGTTCAGGACTTACGCCGCAGGGG
CGTCATCAACTTCGAGGTCTCCCTCATTCGGGATATCCGCGATCGAGAGTTCAAGATTTTCTC
GGATGCTGGTCGTGTTATGCGTCCTCTGTTCGCTGTGGAGCAGTCAGAC---AACTCGACAAA
G---GGCCTTGAAAAGGGCTCTTTATTGCTCACAAAGGAGCATATCCAGAAGTTGAGAGCAGAC
GAGGAGCTATCCAAGGATGAAGAAGACTATTTCGGCTGGGATGGCCTTCTTAGGGCGGGCG
CTATCGAATATCTTGACGCCGAAGAGGAAGAGACAGCCATGATTTGCATGACTCCAGAGGAC
CTAGAGGCTTATCGGCTGCAAAAAGCTGGATTAGACGTGCCGGAAGACGATGACGAGGCCG-
--ACCCCAATAGGCGTCTAAAGACAAAACTAAACCCCACCACTCACATGT-----------------------------
-----------------------------??
???
???
???
???
???
???
???
???
???
???
>Distoseptispora_amniculi_MFLUCC17_2129
ACCT--------TT-TTGT-T------CGTCGCTTTGGCGGG-CGGCTCC-AGGGCGGAGCCACTGCCCG
TTGGGTCGGGTGCCCGCCGAAGGA---CAGC-CAAAACTCGTT-GAATCTAG-TGTATCATCAGA
GTAATA-----AAAA-----AAAGCTAT-CAAAACTTTCAGCAACGGATCTCTTGGTTCTGGCATCGAT
GAAGAACGCAGCGAAATGCGATAAGTAATGTGAATTGCAGAATTCAGTGAATCATCGAATCTT
TGAACGCACATTGCGCCCGCCAGCACTCTGGCGGGCATGCCTGTTCGAGCGTCATTTCAA-C
CCTCGGGCCC-CTGTT-------TGGCCCGGTGTTGGGGTGCTACGGTCCGG--T----------------CG
GAC-CAGTAGGCCCTCAAAAGCAGTGGCGGGCTCGCCAGGGCT-CCGAGCGCAGTAGTTT-T
CCT--CTCGCTCAGGTGGCCT-GGCGTG--CT-TCGGCCGTTAAAA--CACCCA-C------TGTATC----
-TACAG-AAGCCTTCCGAGTCCCC-TGGAAC------GGGGCGCC-AGAGAGGG-TGAGAGCCCCG
TATGGTTGGACGCCGAACC-TCTGTAAAGCTCCTTCGACGAGTCGAGTAGTTTGGGAATGCTG
CTCAAAATGGGAGGTAAATTTCTTCTAAAG-CTAAATACCGGCCAGAGACCGATAGCGCACAA
GTAGAGTGATCGAAAGATGAAAAGCACTTTGAAAAGAGGGTTAAATAGCACGTGAAATTGCTG
AAAGGGAAGCGCTTGTGACCAGACTTGCGCCCGGTTGATCATCCAGCGTTCTCGCTGGTGC
ACTCTGCCGGGCTCAGGCCAGCATCGGCTCTCCCAGGGGGATAAAGGTCGCGGGAACGTA
GCTCTCTTCGGGGAGTGTTATAGCCCGCGG-CGGAATACCCTTAGGGGGGGCCGAGGCCCG
CGTTC-G-CAAGGATGCTGGCGTAATGGTCACCAGCGACCCGTCTTTATCGCTGATGTGTTAT

GTGAGCGTCGGAACACCAGCAGAGCCAATCGTTGAGTTCATGATTGCAAGGAACATGGAGGT
ACTCGAGGAATATGAGCCCTTGAGGTACCCAAATGCGACCAAAATCTTTGTCAACGGTACATG
GGTTGGTGTTCACCAAGACCCGAAGCATCTAGTGAGCTTGGTTCAGGACTTGCGTCGTAGG
GGCGTCATCAATTTCGAGGTCTCACTCATTCGAGATATACGCGACCGAGAATTCAAAATTTTCT
CGGATGCTGGGCGTGTCATGCGACCGCTTTTTGCTGTTGAGCAGTCAGAT---AACTCGAAAA
CG---GGTCTTGAAAAGGGTTCCTTGTTACTCACAAAAGACCACATCCAGAAGCTGAGAGCAG
ACGAGGAGTTATCCAAGGACGACCCTGACTATTTCGGCTGGGATGGTCTACTCAAAGCCGGC
GCTATCGAGTATCTTGACGCGGAAGAGGAAGAGACAGCGATGATTTGCATGACTCCGGAAGA
TTTGGAGGCTTATCGCCTGCAAAAAGCTGGATTAGATGTACCGGACGACGACGAAGACGCCG
---ACCTCAATAGGCGTTTGAAGACAAAACTGAACCCCACCACTCATATGTACACACATTGTGAG
ATCCATCCGAGCATGCTCTTGGGGATCTGCGCGAGCATTATCCC???????????????????????
???
???
???
???
???
???
???
???
???
???
????
>Distoseptispora_appendiculata_MFLUCC_18_0259
ACCT--------GT---T-TG------TACTGCTCCGGCGGG-CGACTCC-AGGGCGGAGTCACTGCTCATT
TAGTTGGGTGTTCGCCGAAGCGT-TAAAT-CACAACTCTGGCATCTTATAGAACGTCATCAGAG
CACTA------TAAA---ATAAAATAT-AAAAACTTTCAGCAACGGATCTCTTGGTTCTGGCATCGATG
AAGAACGCAGCGAAATGCGATAAGTAATGTGAATTGCAGAATTCAGTGAATCATCGAATCTTT
GAACGCACATTGCGCCCGCCAGCATTCTGGCGAGCATGCCTGTCCGAGCGTCATTTCAAAC
CCTCAGGCTCTAAAAG-------TG-CCTGGTGTTGGGGTGCTACGCCGCCTGTGCTT--------------A
ACAGGTGCGTAGGCCCTGAAATTCAGTGGCGGGTCCGTTAGGGCT-CCGGGCGTAGTAGTTT
TACCA-CCTCGCCTAGGCGGT-C-TAGTGG--CC-CCTGCCGTGAAAAA-AAACAAAA------ACAAA
AAAC-----AACTAGCCTTCTGAGTCCCC-TGGAAC------GGGGCGCC-ATAGAGGG-TGAGAGCC
CCGTATAGTTGGACACCGAGCC-TCTGTAAAGCTCCTTCGACGAGTCGAGTAGTTTGGGAATG
CTGCTCTAAATGGGAGGTAAATTTCTTCTAAAG-CTAAATACCGGCCAGAGACCGATAGCGCA
CAAGTAGAGTGATCGAAAGATGAAAAGCACTTTGAAAAGAGGGTTAAATAGCACGTGAAATTG
CTGAAAGGGAAGCGCTTGTGACCAGACTTGTGCCCAGCGGATCATCCGGCGTTCTCGCCGG
TGCACTCCACTGGGTTCAGGCCAGCATCGGTTTCGCCAGGGGGACAAAGCCTTGGGAACG
TAGCTCTCTTCGGGGAGTGTTATAGCCCGTGG-CACAATGCCCTTAGGCGGGACCGAGGTTC
GCGCTCTG-CAAGGATGCTGGCGTAATGGTCATCAGCGACCCGTCTT???????????????????
???
???
???
???
???

???
???
???
??ACTCGTGAGCACGCTTTGC
TTGCCTACACCCTCGGTGTGAAGCAGCTCATCGTGGCCATCAACAAGATGGACACCACCAAG
TGGTCCGAGGATCGTTACAACGAAATCATCAAGGAGACCTCCAACTTCATCAAGAAGGTCGG
CTACAACCCCAAGCAGGTTGCCTTCGTCCCTATCTCCGGCTTTAATGGCGACAACATGCTTAC
GGCCTCCTCCAACTGCCCCTGGTACAAGGGCTGGGAGAAGGAGA---TCAAGGGTGGCAAGG
TCACCGGCAAGACCCTCCTCGAGGCTATTGATGCCATCGAGCCTCCCAAGCGTCCCACCGA
CAAGCCTCTCCGCCTTCCCCTTCAGGATGTCTACAAGATTGGTGGTATCGGAACTGTCCCCG
TCGGCCGTATCGAGACTGGTATCCTCAAGCCTGGTATGGTCGTTACGTTCGCTCCTTCCAAC
GTCACTACTGAAGTCAAGTCCGTCGAAATGCACCACGAGCAGCTTACTGAGGGTGTTCCCG
GTGACAACGTTGGCTTCAACGTGAAGAACGTCTCCGTCAAGGAAATCCGTCGTGGCAACGT
CGCTGGTGACTCCAAGAACGACCCCCCTGCTGGTTGCGCTTCTTTCACGGCCCAGGTTATC
GTCTTGAATCACCCCGGTCAGGTCGGTGCCGGTTACGCCCCTGTTCTGGACTGCCACACCG
CACACATCGCTTGCAAGTTCTCTGAGATCCTCGAGAAGATTGATCGCCGAACGGGCAAGTCA
GTCGAGAACAACCCCAAGTTCATCAAGTCGGGCGACGC
>Distoseptispora_aqualignicola_KUNCC_21_10729
ACCC--------ATCGTCAAA------TGTTGCTTCGGCGGG-CGGCCCC-TGGGAGGGGCCAAGGCC
CGTTGGGTTGGGTGCCCGCCGGAGGA---CAGC-CGAAAACTCTTTC----ATCAGGTACTGTCT
GAGTAAA------CTATA--AAAA--CTAT-AAAAACTTTCAACAACGGATCTCTTGGTTCTGGCATCGA
TGAAGAACGCAGCGAAATGCGATAAGTAATGTGAATTGCAGAATTCAGTGAATCATCGAATCT
TTGAACGCACATTGCGCCCGCCAGCACTCTGGCGGGCATGCCTGTTCGAGCGTCATTTCA-A
CCCTCAGGCCC-TA-------GTTTGGCCTGGTGTTGGGGCACTGCCGTCCCGCCCC--------------G
TACGGGGCGCGCAGGCCCTGAAAATCAGTGGCGGGCTCGCTAGCAC-TCCGGGCGTAGTAG
A-ATCAT-ATCTCGTCTAGGTGGTCT-GGCGCG--GT-CTTGCCGTCAAAAC-CCCCC---------ATTT
TTAC-------GCAAGCCTTCTAAGTCCCCCTAGAAC------GGGGCGCCCATAGAGGG-TGAGAGCC
CCGTACAGTTGGACACCGAACCCTTTGTAAAGCTCCTTCGACGAGTCGAGTAGTTTGGGAAT
GCTGCTCAAAATGGGAGGTATATTTCTTCTAAAGGCTAAATACCGGCCAGAGACCGATAGCGC
ACAAGTAGAGTGATCGAAAGATGAAAAGCACTTTGAAAAGAGGGTTAAATAGCACGTGAAATT
GTTGAAAGGGAAGCGCTTGTGACCAGACTTGCGCCCGGTGAATCATCCAGCGTTCTCGCTG
GTGCACTTTGCCGGGCTCAGGCCAGCATCGGTTCTTCCAGGGGGATAAAGGTCTCGGGAAC
GTAGCTCTCTTCGGGGAGTGTTATAGCCCGTGG-CATAATACCTTTGGG-GGGACCGAGGACC
GCGCTCTG-CAAGGATGCTGGCGTAATGGTCATCAGCGACCCGTCTTTATCGCTCATGTGTTA
TGTCAGCGTTGGCACACCGGCAGAACCGATTATCGAGTTTATGATCGCGAGGAATATGGAAG
TCCTGGAGGAATATGAGCCCTTACGCTATCCCAACGCAACTAAGATATTTGTCAACGGGACTT
GGGTGGGCGTTCACCAGGACCCCAAACACTTGGTTGGGCTGGTACAGGAGTTGCGCCAGA
GGGGAGTTATCAACTTTGAGGTGTCCCTTGTGCGTGATATCCGAGACAGGGAGTTCAAGATC
TTCTCCGATGCAGGTCGGGTGATGCGACCCCTTTTCGCCGTGGAGCAGGAGGAC---AATTCT
AAGTCG---GGGCTTCCAAAGGGATCTTTGAGGCTCACCAAAGAGCATATCCAGAGGCTCAGA
GCGGACGAGGACTTGTCCAAAGAGGATCCGGAATACTTCGGATGGGACGGCATCGTTGAGG
CCGGCGCAGTCGAATATCCCGACGCGGAAGAAGAGGAAACGGCTATGATATGCATGACGCC
GGAAGACCTGGAGGCTTACAGACTCCAAAAAGCCGGGTTGGACATGGCAGAAGATGACGAA
GAAGCGG---ACCCAAACAGACGCCTGAAAACGAAATTAAACCCTACCACTCACATGTACACAC

ACTGCGAGATTCATCCCAGTATGCTATTGGGAATCTGCGCAAGCATCATACCACTCGTGAGCA
CGCCCTGCTTGCCTACACCCTGGGTGTCAAGCAGCTCATCTGCGCCATCAACAAGATGGACA
CCACCAAGTGGTCAGAGGACCGTTTCAATGAAATCATCAAGGAGACGTCCAACTTCATCAAG
AAGGTCGGCTACAACCCCAAGCAGGTCGCCTTCGTCCCTATCTCTGGCTTCCACGGCGACA
ACATGTTGGCTGCCTCCACCAACTGCCCCTGGTACAAGGGCTGGGAGAAGGAGGGCGCCA
AGGGTGCCAAGTCTTCTGGTAAGACCCTGCTCGAGGCCATCGACGCCATCGAGCAACCCAA
GCGTCCTACCGACAAGCCTCTGCGTCTCCCGCTCCAGGATGTCTACAAGATCGGTGGTATCG
GAACTGTCCCTGTCGGCCGTATCGAGACTGGTATCCTCAAGCCCGGTATGGTCGTCACTTTC
GCTCCCTCCAACGTCACCACTGAAGTCAAGTCCGTCGAGATGCACCACGAGCAGCTTACTG
AGGGTGTTCCCGGCGACAACGTCGGCTTCAACGTGAAGAACGTTTCCGTCAAGGAAATCCG
CCGTGGAAACGTCGCTGGTGACTCCAAGAACGACCCTCCTGCTGGCTGCGCCAACTTCACT
GCTCAGGTCATTGTCCTCAACCACCCTGGTCAGGTCGGCCCTGGTTACGCTCCCGTCTTGG
ATTGCCACACCGCCCACATCGCCTGCAAGTTCTCTGAGATCACCGAGAAGATCGACCGCCGT
ACCGGCAAGTCAGTTGAGAACAACCCCAAGTTCATCAAGTCTGGTGACGC
>Distoseptispora_aquamyces_KUNCC_21_10732
ACCC--------ATCGTCAAA------TGTTGCTTCGGCGGG-CGGCCCC-TGGGAGGGGCCAAGGCC
CGTTGGGTTGGGTGCCCGCCGGAGGA---CAGC-CGAAAACTCTTTT----ATCAGGTACTGTCTG
AGTAAA------CTATAT-AAAA--CTAT-AAAAACTTTCAACAACGGATCTCTTGGTTCTGGCATCGAT
GAAGAACGCAGCGAAATGCGATAAGTAATGTGAATTGCAGAATTCAGTGAATCATCGAATCTT
TGAACGCACATTGCGCCCGCCAGCACTCTGGCGGGCATGCCTGTTCGAGCGTCATTTCA-AC
CCTCAGGCCC-TA-------GTTTGGCCTGGTGTTGGGGCACTGCCGTCCCGCCC---------------GTA
CGGGGCGCGCAGGCCCTGAAAACCAGTGGCGGGCTCGCTAGCAC-TCCGGGCGTAGTAGA-
ATCAT-ATCTCGTCTAGGTGGTCT-GGCGCG--GT-CTTGCCGTCAAAAC-CCCCCC--------AATTT
TAC-------ACAAGCCTTCTAAGTCCCC-TAGAAC------GGGGCGCC-ACAGAGGG-TGAGAGCCC
CGTACAGTTGGACACCGAACC-TTTGTAAAGCTCCTTCGACGAGTCGAGTAGTTTGGGAATGC
TGCTCAAAATGGGAGGTATATTTCTTCTAAAG-CTAAATACCGGCCAGAGACCGATAGCGCACA
AGTAGAGTGATCGAAAGATGAAAAGCACTTTGAAAAGAGGGTTAAATAGCACGTGAAATTGTT
GAAAGGGAAGCGCTTGTGACCAGACTTGCGCCCGGTGAATCATCCAGCGTTCTCGCTGGTG
CACTTTGCCGGGCTCAGGCCAGCATCGGTTCTCCCAGGGGGATAAAGGTCTCGGGAACGTA
GCTCTCTTCGGGGAGTGTTATAGCCCGTGG-CATAATACCTTTGGG-GGGACCGAGGACCGC
GCTCTG-CAAGGATGCTGGCGTAATGGTCATCAGCGACCCGTCTTTATCGCTCATGTGTTATG
TCAGCGTTGGCACACCGGCAGAACCGATTATCGAGTTTATGATCGCGAGGAATATGGAAGTC
CTCGAGGAATATGAGCCCTTGCGCTATCCCAACGCAACGAAGATATTTGTCAACGGGACTTG
GGTGGGCGTTCACCAGGACCCCAAACACTTGGTTGGGCTGGTGCAGGAGTTGCGCCAGAG
GGGAGTTATCAACTTTGAGGTTTCCCTTGTGCGTGATATCCGAGACAGGGAGTTCAAGATCTT
CTCCGATGCAGGTCGGGTGATGCGACCCCTTTTCGCCGTGGAGCAGGAGGAC---AATTCTAA
GTCG---GGGCTTCCAAAGGGATCTTTGAGACTCACCAAAGAGCATATCCAGAGGCTCAGAGC
GGACGAAGACTTGTCCAAAGAAGATCCGGAATACTTCGGATGGGACGGCATCGTTGAGGCT
GGCGCAGTCGAATATCTCGACGCGGAAGAAGAGGAAACGGCTATGATATGCATGACGCCGG
AAGACCTGGAGGCTTACAGACTCCAAAAAGCCGGGTTGGGCATGGCCGAAGATGACGAAGA
AGCGG---ACCCAAACAGACGCCTGAAAACGAAATTAAACCCTACCACTCACATGTACACACAC
TGCGAGATTCATCCCAGTATGCTATTGGGAATCTGCGCAAGCATCATACCACTCGTGAGCACG
CCCTGCTTGCCTACACCCTGGGTGTCAAGCAGCTCATCTGCGCCATCAACAAGATGGACACC
ACCAAGTGGTCAGAGGACCGTTTCAACGAAATCATCAAGGAGACGTCCAACTTCATCAAGAA

GGTCGGCTACAACCCCAAGCAGGTCGCCTTCGTCCCTATCTCTGGCTTCCACGGCGACAAC
ATGTTGGCTGCCTCCACCAACTGCCCCTGGTACAAGGGCTGGGAGAAGGAGGGCGCCAAG
GGTGCCAAGTCTTCTGGTAAGACCCTGCTCGAGGCCATCGACGCCATCGAGCAACCCAAGC
GTCCTACCGACAAGCCTCTGCGTCTCCCACTCCAGGATGTCTACAAGATCGGTGGTATCGGA
ACTGTCCCTGTCGGCCGTATCGAGACTGGTATCCTCAAGCCTGGTATGGTCGTCACTTTTGC
TCCCTCCAACGTCACCACTGAAGTCAAGTCCGTCGAGATGCACCACGAGCAGCTTACTGAG
GGTGTTCCCGGCGACAACGTCGGCTTCAACGTGAAGAACGTCTCCGTCAAGGAAATCCGCC
GTGGAAACGTCGCTGGTGATTCCAAGAACGACCCTCCTGCTGGCTGCGCCAACTTCACTGC
TCAGGTCATTGTCCTCAACCACCCTGGTCAGGTCGGTCCTGGTTACGCTCCCGTCTTGGATT
GCCACACCGCCCACATCGCCTGCAAGTTCTCTGAGATCACCGAGAAGATCGACCGCCGTAC
CGGCAAGTCAGTTGAGAACAACCCCAAGTTCATCAAGTCTGGTGACGC
>Distoseptispora_aquatica_MFLUCC_18_0646
ACCT--------TTTATATT-------CGTTGCTTTGGCGGG-CGGCTCC-AGGGCGGAGTCACTACCCGT
TGGGTCGGGCGCCCGCCGAAGGA---CAAC-CAAAACTCGCT-AAATCTGT-ACGGATCTCAGA
GTAAAA------TAT-----CAAGCTAT-CAAAACTTTCAGCAACGGATCTCTTGGTTCTGGCATCGATG
AAGAACGCAGCGAAATGCGATAAGTAATGTGAATTGCAGAATTCAGTGAATCATCGAATCTTT
GAACGCACATTGCGCCCGCCAGCACTCTGGCGGGCATGCCTGTTCGAGCGTCATTTCAA-CC
CTCGGGCCC-TCGTT-------TGGCCCGGTGTTGGGGCGCTACGGGTCCG--CC----------------GG
AC-CTGTAGGCCCTCAAAAGCAGTGGCGGGCTCGCCAGGACT-CCGAGCGTAGTAGTTC-TCC
T--CTCGCTCAGGCGGCCT-GGCGTG--CT-TCGGCCGTTAAAA--GACCCAAC------TGTAAT-----C
ACAG-AAGCCTTCCGAGTCCCC-TGGAAC------GGGGCGCC-AGAGAGGG-TGAGAGCCCCGT
ATGGTTGGACGCCGAACC-TCTGTAAAGCTCCTTCGACGAGTCGAGTAGTTTGGGAATGCTG
CTCAAAATGGGAGGTAAATTTCTTCTAAAG-CTAAATACCGGCCAGAGACCGATAGCGCACAA
GTAGAGTGATCGAAAGATGAAAAGCACTTTGAAAAGAGGGTTAAATAGCACGTGAAATTGCTG
AAAGGGAAGCGCTTGTGACCAGACTTGCGCCCGGTTGATCATCCAGCGTTCTCGCTGGTGC
ACTCTGCCGGGCTCAGGCCAGCATCGGCTCTCCCAGGGGGATAAAGGTCGCGGGAACGTA
GCTCTCTCCGGGGAGTGTTATAGCCCGTGG-CGGAATGCCCTTAGGGGGGGCCGAGGCCCG
CG-TTCG-CAAGGATGCTGGCGTAATGGTCACCAGCGACCCGTCTT?????????????????????
??
??
??
??
??
??
??
??
???ACTCGTGAGCACGCTCTGCTT
GCCTACACCTTGGGTGTGAAGCAGCTCATCGTTGCCATCAACAAGATGGACACCACCAAGTG
GTCTGAGAGCCGTTACAACGAAATTATCAAGGAGACGTCCAACTTCATCAAGAAGGTCGGCT
ACAACCCCAAGCAGGTCGCCTTCGTTCCCATCTCCGGCTTCAACGGCGACAACATGCTTGCT
GCCTCCTCCAACTGCCCTTGGTACAAGGGCTGGGAGAAGGAGGTC---AAGGGCGGCAAAGC
CTCTGGCAAGACCCTTCTCGAGGCCATCGATGCCATTGAGCCTCCCAAGCGTCCTACGGAC
AAGCCTCTGCGTCTCCCTCTCCAGGATGTCTACAAGATTGGTGGTATTGGAACTGTGCCTGT
CGGCCGTATCGAGACTGGTATCCTCAAGCCCGGTATGGTCGTTACATTCGCTCCCTCCAACG

TCACCACTGAAGTCAAGTCCGTCGAGATGCACCACGAGCAGCTGACGGAGGGTGTCCCCG
GCGACAACGTCGGCTTCAACGTGAAGAACGTCTCCGTCAAGGAAATCCGCCGAGGCAACGT
TGCTGGTGACTCCAAGAACGATCCTCCTGCTGGTGCCGCTTCTTTCACCGCCCAGGTCATCG
TCCTCAACCACCCCGGTCAGGTCGGCGCTGGCTACGCTCCCGTCTTGGATTGCCACACTGC
CCACATCGCCTGCAAGTTCTCCGAGATCCTGGAGAAGATCGACCGCCGAACGGGCAAGTCT
GTCGAGAACAACCCCAAGTTCATCAAGTCTGGTGACGC
>Distoseptispora_aquatica_S_965
ACCT--------TTTATACT-------CGTTGCTTTGGCGGG-CGGCTCC-AGGGCGGAGTCACTACCCGT
TGGGTCGGGTGCCCGCCGAAGGA---CAAC-CAAAACTCGCT-AAATCTGT-ACGGATCTCAGA
GTAAAAA-----TAT-----CAAGCTAT-CAAAACTTTCAGCAACGGATCTCTTGGTTCTGGCATCGAT
GAAGAACGCAGCGAAATGCGATAAGTAATGTGAATTGCAGAATTCAGTGAATCATCGAATCTT
TGAACGCACATTGCGCCCGCCAGCACTCTGGCGGGCATGCCTGTTCGAGCGTCATTTCAA-C
CCTCGGGCCC-TCGTT-------TGGCCCGGTGTTGGGGCGCTACGGGTCCG--CC-----------------G
GAC-CTGTAGGCCCTCAAAAGCAGTGGCGGGCTCGCCAGGACT-CCGAGCGTAGTAGTTC-T
CCT--CTCGCTCAGGCGGCCT-GGCGTG--CT-TCGGCCGTTAAAA--GACCCAAC------TGTAAT---
--CACAG-AAGCCTTCCGAGTCCCC-TGGAAC------GGGGCGCC-AGAGAGGG-TGAGAGCCCC
GTATGGTTGGACGCCGAACC-TCTGTAAAGCTCCTTCGACGAGTCGAGTAGTTTGGGAATGCT
GCTCAAAATGGGAGGTAAATTTCTTCTAAAG-CTAAATACCGGCCAGAGACCGATAGCGCACA
AGTAGAGTGATCGAAAGATGAAAAGCACTTTGAAAAGAGGGTTAAATAGCACGTGAAATTGCT
GAAAGGGAAGCGCTTGTGACCAGACTTGCGCCCGGTTGATCATCCAGCGTTCTCGCTGGTG
CACTCTGCCGGGCTCAGGCCAGCATCGGCTCTCCCAGGGGGATAAAGGTCGCGGGAACGT
AGCTCTCTCCGGGGAGTGTTATAGCCCGTGG-CGGAATGCCCTTAGGGGGGGCCGAGGCCC
GCGTTC-G-CAAGGATGCTGGCGTAATGGTCACCAGCGACCCGTCTTTATCGCTCATGTGTTAT
GTGAGCGTTGGAACGCCCGCAGAGCCAATTGTCGAGTTCATGATTGCTAGGAACATGGAGGT
ACTCGAGGAATATGAGCCCTTGAGGTACCCTAATGCAACCAAAATCTTCGTCAACGGCACATG
GGTTGGTGTTCACCAAGACCCGAAGCATCTTGTGAGCTTGGTTCAGGACTTACGCCGCAGG
GGCGTCATCAACTTCGAGGTCTCCCTCATTCGGGATATCCGCGATCGAGAGTTCAAGATTTTC
TCGGATGCTGGTCGTGTTATGCGTCCTCTGTTCGCTGTGGAGCAGTCAGAC---AACTCGACA
AAG---GGCCTTGAAAAGGGCTCTTTATTGCTCACAAAGGAGCATATCCAGAAGTTGAGAGCGG
ACGAGGAGCTATCCAAGGATGAAGAAGACTATTTCGGCTGGGATGGCCTTCTTAGGGCGGG
CGCCATCGAATATCTTGACGCTGAAGAGGAAGAGACAGCCATGATTTGCATGACTCCAGAAG
ACCTAGAGGCTTATCGGCTGCAAAAAGCTGGATTAGACGTGCCGGAAGACGATGACGAGGC
CG---ACCCCAATAGGCGTCTAAAGACAAAACTAAACCCCACCACTCACATGTATACACACTGC
GAGATCCATCCGAGCATGCTCTTGGGGATCTGCGCGAGCATTATTCCACTCGTGAGCACGCT
CTGCTTGCCTACACCTTGGGTGTGAAGCAGCTCATCGTTGCCATCAACAAGATGGACACCAC
CAAGTGGTCTGAGAGCCGTTACAACGAAATTATCAAGGAGACGTCCAACTTCATCAAGAAGG
TCGGCTACAACCCCAAGCAGGTCGCCTTCGTTCCCATCTCCGGCTTCAACGGCGACAACAT
GCTTGCTGCCTCCTCCAACTGCCCTTGGTACAAGGGCTGGGAGAAGGAGGTC---AAGGGCG
GCAAAGCCTCTGGCAAGACCCTTCTCGAGGCCATCGATGCCATTGAGCCTCCCAAGCGTCC
TACGGACAAGCCTCTGCGTCTCCCTCTCCAGGATGTCTACAAGATTGGTGGTATTGGAACTG
TGCCTGTCGGCCGTATCGAGACTGGTATCCTCAAGCCCGGTATGGTCGTTACATTCGCTCCC
TCCAACGTCACCACTGAAGTCAAGTCCGTCGAGATGCACCACGAGCAGCTGACGGAGGGTG
TCCCCGGCGACAACGTCGGCTTCAACGTGAAGAACGTCTCCGTCAAGGAAATCCGCCGAGG
CAACGTTGCTGGTGACTCCAAGAACGATCCTCCTGCTGGTGCCGCTTCTTTCACCGCCCAG

GTCATCGTCCTCAACCACCCCGGTCAGGTCGGCGCTGGCTACGCTCCCGTCTTGGATTGCC
ACACTGCCCACATCGCCTGCAAGTTCTCCGAGATCCTGGAGAAGATCGACCGCCGAACGGG
CAAGTCTGTCGAGAACAACCCCAAGTTCATCAAGTCTGGTGACGC
>Distoseptispora_aquisubtropica_GZCC_22_0075
ACCT--------AT--TGTT-------CGTTGCTCTGGCGGG-CGGCTCC-AGGGCGGAGCCACTGTCCGT
TAGGTCGGGCGCCCGCCGGAGGA---CAGC-TAAAACTCGCT-GAATCTCG-GGTGATCTCAGA
GTACCATTA--AAAA-----CAAGCTAT-CAAAACTTTCAGCAACGGATCTCTTGGTTCTGGCATCGA
TGAAGAACGCAGCGAAATGCGATAAGTAATGTGAATTGCAGAATTCAGTGAATCATCGAATCT
TTGAACGCACATTGCGCCCGCCAGCACTCTGGCGGGCATGCCTGTTCGAGCGTCATTTCAA-
CCCTCGGGCCC-TCGTT-------TGGCCCGGTGTTGGGGTGCTACCGTCCCCCCCGC-------------
-GGGGACGCCGTAGGCCCTTAAAACCAGTGGCGGGCTCGCCAGGACT-CCGAGCGCAGTAG
TTTCTCCT--CTCGCTCAGGCGGCCT-GGCGTG--CT-TCGGCCGTTAAACC-CCCC----------------
------------GCCTTCCGAGTCCCC-TGGAAC------GGGGCGCC-AGAGAGGG-TGAGAGCCCCGTA
TGGTTGGACGCCGAACC-TCTGTAAAGCTCCTTCGACGAGTCGAGTAGTTTGGGAATGCTGC
TCAAAATGGGAGGTAAATTTCTTCTAAAG-CTAAATACCGGCCAGAGACCGATAGCGCACAAGT
AGAGTGATCGAAAGATGAAAAGCACTTTGAAAAGAGGGTTAAATAGCACGTGAAATTGCTGAA
AGGGAAGCGCTTGTGACCAGACTTGCGCCCGGTTGATCATCCAGCGTTCTCGCTGGTGCAC
TCTGCCGGGCTCAGGCCAGCATCGGCTCTCCCAGGGGGATAAAGGTCGCGGGAACGTAGC
TCTCTCCGGGGAGTGTTATAGCCCGTGG-CGGAATGCCCTTAGGGGGGGCCGAGGCCCGCG
TTC-G-CAAGGATGCTGGCGTAATGGTCACCAGCGACCCGTCTTTATCGCTCATGTGTTATGTG
AGCGTCGGAACACCCGCAGAGCCCATTGTCGAGTTCATGATTGCTAGGAACATGGAGGTACT
CGAGGAATATGAGCCCTTGCGGTACCCAAATGCGACTAAAATCTTCGTCAATGGCACCTGGG
TTGGTGTTCATCAAGACCCGAAGCATCTCGTGAGCCTAGTTCAAGACTTACGCCGTAGGGGC
GTCATCAACTTCGAGGTCTCTCTTATTCGGGATATACGCGATCGAGAATTCAAGATCTTCTCAG
ATGCTGGTCGCGTCATGCGGCCTCTTTTCGCCGTCGAGCAGTCAGAT---AACTCAAAAACG---
GGCCTTGAAAAAGGCTCTTTGTTGCTTACAAAGGACCATATCCAGAAGTTGAGAGCAGACGA
GGAGTTATCCAAGGATGACCCAGACTTTTTCGGCTGGGATGGCCTTCTCAAAGCGGGCGCTA
TCGAATACCTTGACGCAGAAGAGGAAGAGACAGCGATGATTTGCATGACTCCAGAAGACCTA
GAGGCTTATCGGCTGCAAAAAGCTGGATTGGACGTGCCGGAAGACGATGAAGAAGCCG---A
CCCCAATAGGCGTCTCAAGACGAAGTTGAACCCCACCACTCATATGTATACCCATTGCGAGAT
CCATCCGAGCATGCTCTTGGGAATCTGCGCGAGCATTATTCCACTCGTGAGCACGCTCTGCT
TGCCTACACTCTTGGTGTGAAGCAGCTCATTGTCGCCATCAACAAGATGGACACCACCAAGT
GGTCTGAGGATCGTTACAACGAGATCATCAAGGAGACGTCCAACTTCATCAAGAAGGTCGGC
TACAACCCCAAGCAGGTCGCATTCGTTCCCATCTCTGGCTTCAACGGCGACAACATGCTTAC
CCCCTCCCCCAACTGCCCCTGGTACAAGGGTTGGGAGAAGGAGATC---AAGGGCGGCAAGG
TCTCTGGCAAGACTCTTCTTGAGGCCATCGACTCCATCGAGCCTCCCAAGCGTCCCACAGAC
AAGCCTCTGCGTCTTCCGCTTCAGGATGTCTACAAGATTGGCGGTATCGGAACCGTGCCTGT
CGGCCGTATCGAGACTGGTATCCTCAAGCCCGGTATGGTCGTCACTTTCGCTCCCTCCAATG
TCACCACTGAAGTCAAGTCCGTCGAGATGCACCACGAGCAGCTGACGGAGGGTGTTCCCGG
TGACAACGTTGGCTTCAACATAAAGAACGTATCCGTCAAGGAAATCCGCCGAGGCAACGTCG
CTGGTGACTCCAAGAACGACCCTCCTGCTGGTGCTGCTTCCTTCACCGCCCAGGTCATCGT
CCTCAACCACCCCGGTCAGGTCGGTGCCGGCTACGCTCCCGTCCTGGATTGCCACACCGCC
CACATTGCTTGCAAGTTCTCCGAGATCCTTGAGAAGATCGACCGCCGAACGGGCAAGTCCGT
CGAGAACAACCCCAAGTTCATCAAGTCCGGTGACGC

>Distoseptispora_atroviridis_GZCC_20_0511
ACCT--------GT---TCAG------TACTGCTCCGGCGGG-TGGCTCC-AGGGCGGAGCCACTGCCCG
TTGGGTCGGGTGCCCGCCGAAGCG--TCAAC-CACAACTCTGGCACCC--TTGGGCGTCTTCA
GAGCACAAA-----TAAA---AAAA-CTAT-AAAAACTTTCAGCAACGGATCTCTTGGTTCTGGCATC
GATGAAGAACGCAGCGAAATGCGATAAGTAATGTGAATTGCAGAATTTAGTGAATCATCGAATC
TTTGAACGCACATTGCGCCCGCCAGCATTCTGGCGGGCATGCCTGTCCGAGCGTCATTTCAA
CCCCTCGGGCGCT-GAAG-------CG-TCCGGTGTTGGGGCGCTGCGACGCCTGTAGGC---CCC
GTGCCTG--CGGGCGGCGCAGGCCCTCAAAGTCAGTGGCGGGTCCGTCAGGGCT-CCGGGC
GCAGTAGTTT-ACCA-CCTCGCCCAGGCGGC-C-TGTCGG--CC-CCCGCCGTGAAAAC-ACCCC
--A------ACTGAAGA-------ACCAGCCTTCCGAGTCCCC-TGGAAC------GGGGCGCC-ACAGAGG
G-TGAGAGCCCCGTATGGTTGGACGCCGAGCC-TCTGTAAAGCTCCTTCGACGAGTCGAGTA
GTTTGGGAATGCTGCTCTAAATGGGAGGTAAATTTCTTCTAAAG-CTAAATACCGGCCAGAGAC
CGATAGCGCACAAGTAGAGTGATCGAAAGATGAAAAGCACTTTGAAAAGAGGGTTAAAAAGC
ACGTGAAATTGCTGAAAGGGAAGCGCTCGTGACCAGACTTGCGCCCGGCGGATCAACCGGC
GTTCTCGCCGGTGCACTCCGCCGGGTCCAGGCCAGCATCGGTTTCGCCAGGGGGACAAAA
GCCGCTGGAACGTAGCTCTCTACGGGGAGTGTTATAGCCTGCGG-CACAATGCCCTTAGGCG
GGACCGAGGCCCGCGCTCTG-CAAGGATGCTGGCGTAATGGTCACCAGCGACCCGTCTTTC
TCGCTCATGTGTTATGTCAGTGTTGGGACGCCTTCTGAGCCTATCGTAGACTTCATGGTGGCT
AGAAATATGGAACTTCTCGAGGAGTACGAGCCGCTGCGGTACCCCAACGCCACCAAGATTTT
TGTCAATGGCACATGGGTCGGTGTCCATCAGGACCCGCGGCATTTGGTGAACTCGGTGAAG
GAGCTGCGACAAAAAGGTGTCATCAACTTTGAGGTTTCTCTTATTCGGGATATCCGTGATCGA
GAATTCAAAATTTTCTCGGACGCTGGCCGTGTCATGAGGCCTCTTTTCGCCGTTGAGCAAATT
GGG---GATCCGAGCAAG---AACCGAGAGAAGGGCTCGTTGTGGCTCACTAAGCAACACATCG
AACGACTCAGAGAGGATGAGATGCTGGACCGAGATCATCCTAAATACTTTGGTTGGGACGGT
CTGACCAAAGCCGGTGTCATCGAGTACGTGGATGCGGAAGAAGAAGAGACTGCCATGATTTG
CATGTCACCGGAAGACTTGGAACAATACAGGCTTCAAAAGGCGGGTGTCGAGGTCGAAGAA
GATGATGACGAGGCCG---ATCCCAACCGTCGGCTCAAGACAAAGATGAATCCAACGACGCAC
ATGTACACACATTGTGAGATTCATCCGAGCATGCTTTTGGGGATTTGCGCAAGCATTATCCCA
CTCGTGAGCACGCTCTGCTTGCCTACACACTGGGTGTGAAGCAGCTCATCGTTGCCATCAAT
AAGATGGACACCACCAAGTGGTCTGAGGATCGTTACAACGAAATCATCAAGGAGACTTCCAA
TTTCATCAAGAAGGTCGGCTACAACCCCAAGCAGGTTGCCTTTGTCCCCATCTCTGGCTTCA
ACGGCGACAACATGCTTGCTGCCTCGAGCAACTGCCCCTGGTACAAGGGTTGGGAGAAGGA
GA---TCAAGGGTAACAAGATTACTGGCAAGACTCTCCTCGAGGCCATCGATGCTATTGAGCAA
CCCAAGCGTCCTACCGACAAGCCCCTCCGCCTTCCCCTTCAGGATGTTTACAAGATTGGTGG
TATCGGAACTGTGCCTGTCGGCCGTATCGAGACTGGCATCATCAAGCCTGGTATGGTCGTCA
CCTTCGCTCCCTCCAACGTCACCACTGAAGTCAAGTCTGTCGAGATGCACCACGAACAGCTT
ACTGAGGGTGTTCCCGGCGACAACGTTGGCTTCAACGTGAAGAACGTCTCTGTCAAGGAAA
TCCGTCGTGGCAACGTCGCCGGTGACTCCAAGAACGACCCTCCTTCTGGCTGTGCCTCATT
CACTGCACAGGTCATCGTCCTCAACCACCCTGGTCAGGTTGGTGCTGGCTACGCCCCTGTT
CTGGATTGCCACACTGCCCACATCGCCTGCAAGTTCTCTGAGATCCTCGAGAAGATTGATCG
CCGAACGGGCAAGTCGGTTGAGAACAACCCCAAGTTCATCAAGTCGGGCGACGC
>Distoseptispora_atroviridis_GZCC_19_0531
ACCT--------GT---TCAG------TACTGCTCCGGCGGG-TGGCTCC-AGGGCGGAGCCACTGCCCG
TTGGGTCGGGTGCCCGCCGAAGCG--TCAAC-CACAACTCTGGCACCC--TTGGGCGTCTTCA

GAGCACAAA-----TAAA---AAAAACTAT-AAAAACTTTCAGCAACGGATCTCTTGGTTCTGGCATC
GATGAAGAACGCAGCGAAATGCGATAAGTAATGTGAATTGCAGAATTTAGTGAATCATCGAATC
TTTGAACGCACATTGCGCCCGCCAGCATTCTGGCGGGCATGCCTGTCCGAGCGTCATTTCAA
CCCCTCGGGCGCT-GAAG-------CG-TCCGGTGTTGGGGCGCTGCGACGCCTGTAGGC---CCC
GTGCCTG--CGGGCGGCGCAGGCCCTCAAAGTCAGTGGCGGGTCCGTCAGGGCT-CCGGGC
GCAGTAGTTT-ACCA-CCTCGCCCAGGCGGC-C-TGTCGG--CC-CCCGCCGTGAAAAC-ACCCC
--A------ACTGAAGA-------ACCAGCCTTCCGAGTCCCC-TGGAAC------GGGGCGCC-ACAGAGG
G-TGAGAGCCCCGTATGGTTGGACGCCGAGCC-TCTGTAAAGCTCCTTCGACGAGTCGAGTA
GTTTGGGAATGCTGCTCTAAATGGGAGGTAAATTTCTTCTAAAG-CTAAATACCGGCCAGAGAC
CGATAGCGCACAAGTAGAGTGATCGAAAGATGAAAAGCACTTTGAAAAGAGGGTTAAAAAGC
ACGTGAAATTGCTGAAAGGGAAGCGCTCGTGACCAGACTTGCGCCCGGCGGATCAACCGGC
GTTCTCGCCGGTGCACTCCGCCGGGTCCAGGCCAGCATCGGTTTCGCCAGGGGGACAAAA
GCCGCTGGAACGTAGCTCTCTACGGGGAGTGTTATAGCCTGCGG-CACAATGCCCTTAGGCG
GGACCGAGGCCCGCGCTCTG-CAAGGATGCTGGCGTAATGGTCACCAGCGACCCCGTCTT???
???
???
???
???
???
???
???
???
???ACTCGT
GAGCACGCTCTGCTTGCCTACACACTGGGTGTGAAGCAGCTCATCGTTGCCATCAATAAGAT
GGACACCACCAAGTGGTCTGAGGATCGTTACAACGAAATCATCAAGGAGACTTCCAACTTCA
TCAAGAAGGTCGGCTACAACCCCAAGCAGGTTGCCTTTGTCCCCATCTCTGGCTTCAACGGC
GACAACATGCTTGCTGCCTCGAGCAACTGCCCCTGGTACAAGGGTTGGGAGAAGGAGA---TC
AAGGGTAACAAGATTACTGGCAAGACTCTCCTCGAGGCCATCGATGCTATTGAGCAACCCAA
GCGTCCTACCGACAAGCCCCTCCGCCTTCCCCTTCAGGATGTTTACAAGATTGGTGGTATCG
GAACTGTGCCTGTCGGCCGTATCGAGACTGGCATCATCAAGCCTGGTATGGTCGTCACCTTC
GCTCCCTCCAACGTCACCACTGAAGTCAAGTCTGTCGAGATGCACCACGAACAGCTTACTGA
GGGTGTTCCCGGCGACAACGTTGGCTTCAACGTGAAGAACGTCTCTGTCAAGGAAATCCGT
CGTGGCAACGTCGCCGGTGACTCCAAGAACGACCCTCCTTCTGGCTGTGCCTCATTCACTG
CACAGGTCATCGTCCTCAACCACCCTGGTCAGGTTGGTGCTGGCTACGCCCCTGTTCTGGAT
TGCCACACTGCCCACATCGCCTGCAAGTTCTCTGAGATCCTCGAGAAGATTGATCGCCGAAC
GGGCAAGTCGGTTGAGAACAACCCCAAGTTCATCAAGTCGGGCGACGC
>Distoseptispora_bambusae_MFLUCC_14_0583
ACCT--------ATCGTCAA-------TGTTGCTTCGGCGGG-CGGCTCC-TGGGAGGGGCCACGGCCC
ATTGGGTTGGGTGCCCGCCGGAGGA---CAGC-CGAAAACTCTTTTTT--ATCAGGTACTGTCTG
AGTAAA------TTATAA-AAAAAACTAT-AAAAACTTTCAACAACGGATCTCTTGGTTCTGGCATCGA
TGAAGAACGCAGCGAAATGCGATAAGTAATGTGAATTGCAGAATTCAGTGAATCATCGAATCT
TTGAACGCACATTGCGCCCGCCAGCACTCTGGCGGGCATGCCTGTTCGAGCGTCATTTCA-A
CCCTCAGGCCCCTA-------GTTTGGCCTGGTGTTGGGGCACTGCCGTCCCGCCC--------------G
TACGGGGCGCGCAGGCTCTGAAAATCAGTGGCGGGCTCGCTAGCAC-TCCGGGCGTAGTAG

A-ATTTTTATCTCGTCTAGGTGGTCT-GGCGCG--GT-CTTGCCGTC---------------------------------------
-GCCTTCTAAGTCCCC-TAGAAC------GGGGCGCC-ATAGAGGG-TGAGAGCCCCGTACAGTTG
GACACCGAACC-TTTGTAAAGCTCCTTCGACGAGTCGAGTAGTTTGGGAATGCTGCTCAAAAT
GGGAGGTATATTTCTTCTAAAG-CTAAATACCGGCCAGAGACCGATAGCGCACAAGTAGAGTG
ATCGAAAGATGAAAAGCACTTTGAAAAGAGGGTTAAATAGCACGTGAAATTGTTGAAAGGGAA
GCGCTTGTGACCAGACTTGCGCCCGGTGAATCATCCAGCGTTCTCGCTGGTGCACTTTGCC
GGGCTCAGGCCAGCATCGGTTCTCCTAGGGGGATAAAAGTCTCGGGAACGTAGCTCTCTTC
GGGGAGTGTTATAGCCCGTGG-CATAATACCCTTGGG-GGGACCGAGGACCGCGCTCTG-CAA
GGATGCTGGCGTAATGGTCATCAGCGACCCGTCTTTATCGCTCATGTGTTATGTCAGCGTTGG
CACACCGGCAGAGCCGATTATCGAGTTTATGATCGCGAGGAATATGGAAGTCCTCGAGGAATA
CGAGCCCTTGCGCTACCCCAACGCAACTAAGATATTTGTCAATGGGACATGGGTGGGCGTTC
ACCAGGACCCCAAACACTTGGTTGGGCTGGTACAGGAGTTGCGACAGAGGGGAGTCATCAA
CTTTGAGGTGTCCCTTGTGCGCGATATCCGAGACAGGGAGTTCAAGATCTTCTCCGATGCTG
GTCGGGTGATGCGACCCCTTTTCGCCGTGGAACAGGAGGAC---AATTCTAAGTCA---GGGCT
TCCAAAGGGATCCTTAAGGCTCACCAAGGAGCATATCCAGAGGCTCAGAGCGGACGAAGAC
TTGTCCAAAGAAGACCCGGAATACTTCGGATGGGACGGCATCGTTGAGGCTGGCGCAGTTG
AATATCTCGACGCGGAAGAAGAGGAAACGGCAATGATATGCATGACGCCGGAAGATCTGGAG
GCTTACAGACTCCAAAAAGCCGGGTTGGGCATGGCAGAAGATGACGAAGAAGCGG---ACCC
AAACAGACGCCTGAAAACGAAGTTAAACCCTACCACTCACATGTATACACACTGTGAGATTCA
TCCCAGTATGCTATTGGGAATCTGCGCAAGCATCATACC??????????????????????????????
???
???
???
???
???
???
???
???
???
??
>Distoseptispora_bambusae_MFLUCC_20_0091
ACCT--------ATCGTCAA-------TGTTGCTTCGGCGGG-CGGCCCC-TGGGAGGGGCCACGGCCC
ATTGGGTTGGGTGCCCGCCGGAGGA---CAGC-CGAAAACTCTTTTT---ATCAGGTACTGTCTG
AGTAAA------CTATAA-AA----CTAT-AAAAACTTTCAACAACGGATCTCTTGGTTCTGGCATCGAT
GAAGAACGCAGCGAAATGCGATAAGTAATGTGAATTGCAGAATTCAGTGAATCATCGAATCTT
TGAACGCACATTGCGCCCGCCAGCACTCTGGCGGGCATGCCTGTTCGAGCGTCATTTCA-AC
CCTCAGGCCCCTA-------GTTTGGCCTGGTGTTGGGGCACTGCCGTCCCGCCC--------------GT
ACGGGGCGCGCAGGCTCTGAAAATCAGTGGCGGGCTCGCTAGCAC-TCCGGGCGTAGTAGA
-ATTTT-ATCTCGTCTAGGTGGTCT-GGCGCG--GT-CTTGCCGTCAAAAC-CCCCC---------AATTT
TAT-------A---GCCTTCTAAGTCCCC-TAGAAC------GGGGCGCC-ATAGAGGG-TGAGAGCCCCGT
ACAGTTGGACACCGAACC-TTTGTAAAGCTCCTTCGACGAGTCGAGTAGTTTGGGAATGCTG
CTCAAAATGGGAGGTATATTTCTTCTAAAG-CTAAATACCGGCCAGAGACCGATAGCGCACAAG
TAGAGTGATCGAAAGATGAAAAGCACTTTGAAAAGAGGGTTAAATAGCACGTGAAATTGTTGA
AAGGGAAGCGCTTGTGACCAGACTTGCGCCCGGTGAATCATCCAGCGTTCTCGCTGGTGCA

CTTTGCCGGGCTCAGGCCAGCATCGGTTCTCCTAGGGGGATAAAAGTCTCGGGAACGTAGC
TCTCTTCGGGGAGTGTTATAGCCCGTGG-CATAATACCCTTGGG-GGGACCGAGGACCGCGC
TCTG-CAAGGATGCTGGCGTAATGGTCATCAGCGACCCGTCTTTATCGCTCATGTGTTATGTCA
GCGTTGGCACACCGGCAGAGCCGATTATCGAGTTTATGATCGCGAGGAATATGGAAGTCCTC
GAGGAATACGAGCCCTTGCGCTACCCTAACGCAACTAAGATATTTGTCAACGGGACATGGGT
GGGCGTTCACCAGGACCCCAAACACTTGGTTGGGCTGGTGCAGGAGTTGCGACAGAGGGG
AGTCATCAACTTTGAGGTTTCTCTTGTGCGCGATATCCGAGACAGGGAGTTCAAGATCTTCTC
CGACGCTGGTCGGGTGATGCGACCCCTTTTCGCCGTGGAACAGGAGGAC---AACTCTAAGT
CG---GGCCTTCCAAAGGGATCTTTAAGGCTCACCAAGGAGCATATCCAGAGGCTCAGAGCGG
ACGAAGACTTGTCCAAAGAAGACCCGGAATACTTCGGATGGGACGGCATCGTTGAGGCTGG
CGCAGTCGAATATCTCGACGCGGAAGAAGAGGAGACGGCTATGATATGCATGACGCCGGAA
GATCTGGAGGCTTACAGACTCCAAAAAGCCGGATTGGGCATGGCAGAAGATGACGAAGAAG
CGG---ACCCAAACAGACGCCTAAAAACGAAATTAAACCCTACCACTCACATGTACACACACTG
TGAGATTCATCCCAGTATGCTATTGGGAATCTGCGCAAGCATCATACCACTCGTGAGCACGCC
CTGCTTGCCTACACCCTGGGTGTCAAGCAGCTCATCTGCGCCATCAACAAGATGGATACCAC
CAAGTGGTCAGAGGACCGTTTCAACGAAATCATCAAGGAGACGTCCAACTTCATTAAGAAGG
TCGGCTACAACCCCAAGCAGGTCGCATTCGTTCCCATCTCTGGCTTCCACGGCGACAACATG
CTGGAGGCTTCCAAGAACTGCCCCTGGTACAAGGGCTGGGAGAAGGAGGGCGCCAAGGGT
GCCAAGTCTTCTGGTAAGACCCTGCTCGAGGCCATCGACGCCATCGAGCCCCCCAAGCGTC
CCACCGACAAGCCCCTGCGTCTCCCCCTCCAGGATGTCTACAAGATCGGTGGTATCGGAACT
GTCCCTGTCGGCCGTATCGAGACTGGTGTCCTCAAGCCCGGTATGGTCGTCACTTTCGCTCC
CTCCAACGTCACCACTGAAGTCAAGTCCGTCGAGATGCACCACGAGCAGCTTACTGAGGGT
GTTCCCGGCGACAACGTCGGCTTCAACGTGAAGAACGTCTCCGTCAAGGAAATCCGCCGTG
GCAACGTTGCTGGTGACTCCAAGAACGACCCTCCTTCTGGCTGCGCCAACTTCACTGCTCA
GGTCATTGTCCTCAACCACCCTGGTCAGGTCGGCCCTGGTTACGCCCCCGTCTTGGATTGC
CACACCGCCCACATCGCCTGCAAGTTCTCTGAGATCACCGAGAAGATCGACCGCCGTACCG
GCAAGGCAGTTGAGACCAACCCCAAGTTCATCAAGTCTGGTGACGC
>Distoseptispora_bambusicola_GZCC21_0667
ACCT--------TTT--GTT-------CGTTGCTCCGGCGGG-CGGCTCC-AGGGCGGAGCCACTGTCCGT
TAGGTCGGGCGCCCGCCGGAGGA---CAAC-CAAAACTCGTC-TGATCTC-GGGTATCATCAGA
GTATAT----AAAA------CAAGCTAT-CAAAACTTTCAGCAACGGATCTCTTGGTTCTGGCATCGATG
AAGAACGCAGCGAAATGCGATAAGTAATGTGAATTGCAGAATTCAGTGAATCATCGAATCTTT
GAACGCACATTGCGCCCGCCAGTACTCTGGCGGGCATGCCTGTTCGAGCGTCATTTCAA-CC
CTCGGGCC-CTCTG------TTTGGCCCGGTGCTGGGGTGCTAC--GTCCTGA--------------------CGG
ACCTGTAGGCCCTCAAAACCAGTGGCGGGCTCGCCATGGCCTCCGAGCGCAGTAGTTG-TC
TC--CTCGCTCAGGTGGTCT-GGCGCG--CT-CCGGCCGTTAAA---CACCCG-C------TGTAT-CT----
ACAG-AAGCCTTCCGAGTCCCC-TGGAAC------GGGGCGCC-AGAGAGGG-TGAGAGCCCCGT
ATGGTCGGACGCCGAACC-TTTGTAAAGCTCCTTCGACGAGTCGAGTAGTTTGGGAATGCTG
CTCAAAATGGGAGGTAAATTTCTTCTAAAG-CTAAATACTGGCCAGAGACCGATAGCGCACAA
GTAGAGTGATCGAAAGATGAAAAGCACTTTGAAAAGAGGGTTAAATAGCACGTGAAATTGCTG
AAAGGGAAGCGCTTGTGACCAGACTTGTGCCCGGTTGATCATCCAGCGTTCTCGCTGGTGC
ACTCTGCCGGGCTCAGGCCAGCATCGGCTCCCCCAGGGGGATAAAGGTCGCGGGAACGTA
GCTCTCTCCGGGGAGTGTTATAGCCCGTGG-CGGAATACCCTTAGGGGGGGTCGAGGCCCG
CGTTC-G-CAAGGATGCTGGCGTAATGGTCACCAGCGACCCGTCTT????????????????????

??
??
??
??
??
??
??
??
??ACTCGTGAGCACGCCCTGCTT
GCCTACACCCTGGGTGTCAAGCAGCTCATCGTCGCCATCAACAAGATGGACACCACCAAGT
GGTCTGAGGATCGTTACAACGAAATCATCAAGGAGACGTCCAACTTCATCAAGAAGGTCGGA
TACAACCCCAAGCAGGTGGCTTTCGTTCCCATCTCCGGCTTCAACGGCGACAACATGCTGGC
CCCCTCTTCCAACTGCCCCTGGTACAAGGGATGGGAGAAGGAGATC---AAGGGCAACAAGAT
CACTGGCAAGACCCTCCTCGAGGCCATCGATGCCATTGAGACTCCCAAGCGACCCACCGAC
AAGGCCCTCCGTCTTCCCCTTCAGGACGTCTACAAGATCGGTGGTATTGGCACTGTGCCCGT
CGGCCGTATCGAGACTGGTGTCCTCAAGCCCGGTATGGTCGTTACATTCGCTCCCTCCAACG
TCACCACTGAAGTCAAGTCCGTCGAGATGCACCACGAGCAGCTGAGTGAGGGTCTCCCTGG
CGACAATGTTGGCTTCAACGTGAAGAACGTCTCCGTCAAGGAAATCCGCCGTGGCAACGTC
GCTGGTGACTCCAAGAACGATCCTCCTGCCGGTGCTGCTTCCTTCACCGCCCAGGTCATCG
TCCTCAACCACCCTGGTCAGGTCGGCGCTGGCTACGCTCCCGTCTTGGATTGCCACACCGC
CCACATCGCCTGCAAGTTCTCTGAGATCCTCGAGAAGATTGACCGCCGAACGGGCAAGTCT
GTCGAGAACAACCCCAAGTTCATCAAGTCTGGTGACGC
>Distoseptispora_bangkokensis_MFLUCC_18_0262
ACCT--------TT-TTTGTT------CGTTGCTTTGGCGGG-CGGCTCC-AGGGCGGAGCCACTGCTCG
TTGGGTCGGGTGCCCGCCGAAGGA---CAAC-CAAAAACTCGT-TA---TTA-CGTTTCGTCAGAGT
ACTATT---ATAA-----AAAGCTAT-CAAAACTTTCAGCAACGGATCTCTTGGTTCTGGCATCGATGA
AGAACGCAGCGAAATGCGATAAGTAATGTGAATTGCAGAATTCAGTGAATCATCGAATCTTTG
AACGCACATTGCGCCCGCCAGCACTCTGGCGGGCATGCCTGTTCGAGCGTCATTTCAA-CCC
TCGGGCCC-CTGTT-------TGGCCCGGTGTTGGGGTGCTACGGTCCCG--T----------------AGGA
C-CGGTAGGCCCTTAAAACCAGTGGCGGGCTCGCCAGGACT-CCGAGCGTAGTAGTTT-TCCT-
-CTCGCTTAGGCGGTCT-GGCGTG--CT-TCGGCCGTTAAA---TACTCA-C------TGTACTT---GTAC
AG-AAGCCTTCCGAGTCCCC-TGGAAC------GGGGCGCC-AGAGAGGG-TGAGAGCCCCGTAT
GGTTGGACGCCGAACC-TCTGTAAAGCTCCTTCGACGAGTCGAGTAGTTTGGGAATGCTGCT
CAAAATGGGAGGTAAATTTCTTCTAAAG-CTAAATACCGGCCAGAGACCGATAGCGCACAAGT
AGAGTGATCGAAAGATGAAAAGCACTTTGAAAAGAGGGTTAAATAGCACGTGAAATTGCTGAA
AGGGAAGCGCTTGTGACCAGACTTGCGCCCGGTTGATCATCCAGCGTTCTCGCTGGTGCAC
TCTGCCGGGCTCAGGCCAGCATCGGCTCTCCCAGGGGGATAAAGGTCGCGGGAACGTAGC
TCTCTTCGGGGAGTGTTATAGCCCGTGG-CGGAATACCCTTAGGGGGGGCCGAGGCCCGCG
TTC-G-CAAGGATGCTGGCGTAATGGTCACCAGCGACCCGTCTT???????????????????????
??
??
??
??
??

???
???
???
??ACTCGTGAGCACGCTCTGCTTG
CCTACACCCTGGGTGTGAAGCAGCTCATCGTCGCCATCAATAAGATGGACACCACCAAGTGG
TCTGAGGATCGTTACAACGAAATCATCAAGGAGACGTCCAACTTCATCAAGAAGGTCGGCTA
CAACCCCAAGCAGGTCGCTTTCGTTCCCATCTCCGGCTTCAACGGCGACAACATGCTTGCTG
CTTCCAGCAACTGCCCCTGGTACAAGGGCTGGGAGAAGGAGATC---AAGGGCGGCAAGGTC
ACTGGCAAGACCCTCCTCGAGGCCATCGATGCCATTGAGCCTCCCAAGCGTCCCACCGACA
AGCCTCTGCGTCTTCCCCTTCAGGACGTCTACAAGATTGGTGGTATCGGAACAGTGCCTGTC
GGCCGTATCGAGACTGGTGTCCTCAAGCCCGGTATGGTCGTCACATTCGCTCCGTCCAATGT
CACCACTGAAGTCAAGTCCGTGGAGATGCACCACGAGCAGCTGCAAGAGGGTGTTCCTGGC
GATAACGTTGGCTTCAACGTGAAGAACGTCTCCGTCAAGGAGATTCGCCGAGGCAACGTTG
CCGGTGACTCCAAGAACGACCCTCCTGCTGGTGCTGCTTCCTTCACCGCCCAGGTCATCGT
CCTCAACCACCCTGGTCAGGTCGGTGCCGGCTACGCTCCTGTGTTGGATTGCCACACTGCC
CACATCGCCTGCAAGTTCTCTGAGATCCTCGAGAAGATTGACCGCCGAACGGGCAAGTCCG
TTGAGAACAACCCCAAGTTCATCAAGTCTGGTGATGC
>Distoseptispora_cangshanensis_MFLUCC_16_0970
ACCT--------TTTTTGTT-------CGTTGCTTTGGCGGG-CGGCTCC-AGGGCGGAGCCACTGCCCG
TTGAGTCGGGCGCCTGCCAAAGGA---CAAC-CAAAACTATCT-AAATCTC-GTGTAACGTCAGA
GTATTA-----AAAC-----AAAACTAT-CAAAACTTTCAGCAACGGATCTCTTGGTTCTGGCATCGATG
AAGAACGCAGCGAAATGCGATAAGTAATGTGAATTGCAGAATTCAGTGAATCATCGAATCTTT
GAACGCACATTGCGCCCGCCAGTACTCTGGCGGGCATGCCTGTTCGAGCGTCATTTCAA-CC
CTCAGGCCCTT--GT-----T-TGGCCTGGTGTTGGGGTTCTAC-GGTCCGGC---------------------GGA
CCGGTAGGCCCTTGAAACCAGTGGCGGGCTCGCCAGGGCT-CCGAGCGTAGTAGTTT-TCTT-
-CTCGCTCAGGTAACCT-GGCGTG--CT-TTGGCCGTTAAA---CACCTG-C------TGTATTTT----ACA
G-AAGCCTTCCGAGTCCCC-TGGAAC------GGGGCGCC-AGAGAGGG-TGAGAGCCCCGTATG
GTTGGACGCCGAACC-TCTGTAAAGCTCCTTCGACGAGTCGAGTAGTTTGGGAATGCTGCTC
AAAATGGGAGGTAAATTTCTTCTAAAG-CTAAATACCGGCCAGAGACCGATAGCGCACAAGTA
GAGTGATCGAAAGATGAAAAGCACTTTGAAAAGAGGGTTAAATAGCACGTGAAATTGCTGAAA
GGGAAGCGTTTGTGACCAGACTTGTGCCCGGTTGATCATCCAGCGTTCTCGCTGGTGCACT
CTGCCGGGCTCAGGCCAGCATCGGCTCTCCCAGGGGGATAAAGGTCGTGGGAACGTAGCT
CTCTTCGGGGAGTGTTATAGCCCGTGG-CGGAATACCTCTAGGGGGGGCCGAGGCCCGCG-T
TCG-CAAGGATGCTGGCGTAATGGTCACCAGCGACCCGTCTT???????????????????????????
???
???
???
???
???
???
???
???
??ACTCGTGAGCACGCTCTGCTTGCC
TACACCCTGGGTGTGAAGCAGCTCATCGTCGCCATTAACAAGATGGACACCACCAAATGGTC

TGAGAGCCGTTTCAACGAAATCATCAAGGAGACGTCCAACTTCATCAAGAAGGTCGGCTACA
ACCCCAAGACGGTCGCTTTCGTTCCCATCTCCGGCTTCAACGGCGACAACATGCTTGCCGC
CTCCAGCAACTGCCCCTGGTACAAGGGCTGGGAGAAGGAGATC---AAGGGCGGCAAGGTCA
CTGGCAAGACGCTCCTCGAGGCTATCGATGCCATTGAGCAGCCCAAGCGTCCCACAGACAA
GCCTCTGCGTCTCCCCCTTCAGGATGTCTACAAGATTGGTGGTATCGGAACTGTGCCTGTCG
GCCGTATCGAGACTGGTATCATCAAGCCCGGTATGGTCGTCACGTTCGCTCCCTCCAACGTC
ACCACTGAAGTCAAGTCCGTCGAGATGCATCACGAGCAGCTGACAGAGGGTGTTCCCGGCG
ACAACGTCGGCTTCAACGTGAAGAACGTCTCCGTCAAGGAAATCCGCCGAGGAAACGTCGC
TGGTGACTCCAAGAACGATCCTCCTGCTGCTGCCGCTTCCTTCACCGCCCAGGTCATCGTCC
TCAACCACCCCGGTCAAGTCGGTGCTGGCTACGCTCCCGTTTTGGATTGCCACACTGCCCA
CATCGCTTGCAAGTTCGCTGAGATCCTCGAGAAGATCGACCGCCGAACGGGCAAGTCGGTC
GAGAACAACCCCAAGTTCATCAAGTCTGGTGACGC
>Distoseptispora_caricis_CPC_36498
ACCT--------TC---TC-G------TACTGCTCCGGCGGG-TGGCTCC-AGGGCGGAGCCACTGCCCGT
TCGATCGGGTGCCCGCCGAAGCG--CCAAC-CAAAACTCTGGCACCT--CGGGTTGTCTTCAGA
GCACA--------AAA---CAAATATAT-AAAAACTTTCAGCAACGGATCTCTTGGTTCTGGCATCGATG
AAGAACGCAGCGAAATGCGATAAGTAATGTGAATTGCAGAATTTAGTGAATCATCGAATCTTTG
AACGCACATTGCGCCCGCCAGTATTCTGGCGGGCATGCCTGTCCGAGCGTCATTTCAAACCC
TCGGGCGCT-CCGG-------CG-CCCGGTGTTGGGGCGCTGCGACGCCTGCGGGG-CCCCCGT
GCCCCGCGAGGCGGCGCAGGCCCTCAAAGTCAGTGGCGGGTCTGTCAGGGCT-CCGGGCG
CAGTAGTAT-AACATCCTCGCCCAGGCGGT-C-TGACGG--CC-CCTGCCGTTAAATA-ATCACAT
C------AGTGACGA-------ACCAGCCTTCCGAGTCCCC-TGGAAC------GGGGCGCC-ACAGAGGG
-TGAGAGCCCCGTATGGTTGGACGCCGAGCC-TCTGTGAAGCTCCTTCGACGAGTCGAGTAG
TTTGGGAATGCTGCTCTAAATGGGAGGTAAATTTCTTCTAAAG-CTAAATACCGGCCAGAGACC
GATAGCGCACAAGTAGAGTGATCGAAAGATGAAAAGCACTTTGAAAAGAGGGTTAAAAAGCA
CGTGAAATTGCTGAAAGGGAAGCGCTTGTGACCAGACTTGCACCCGGCGGATCAACCGGCG
TTCTCGCCGGTGCACTCCGCCGGGTCCAGGCCAGCATCGGTTTCGCCAGGGGGACAAAAG
CCTTGGGAACGTAGCTCCCTCCGGGGAGTGTTATAGCCCGTGG-CACAATGCCCTTAGGCGG
GACCGAGGCCCGCGCTCTG-CAAGGATGCTGGCGTAATGGTCACCAGCGACCCGTCTTTGT
CCCTCATGTGTTATGTCAGTGTCGGCACGCCTTCGGAGCCTATCATCGACTTTATGATAGCTA
GAAACATGGAGCTTCTCGAGGAGTATGACCCGGTACGTGACCCTAACGCCACCAAGATTTTT
GTCAATGGCACATGGGTTGGTGTACACCAGGACCCGCGGCACTTGGTGAACTCAGTGAAGG
AGTTGCGGCAGAAGGGTGTCATCAATTTCGAGGTTTCTCTCATTCGCGATATCCGTGATAGAG
AGTTCAAGATTTTCTCGGACGCTGGCCGAGTCATGAGGCCGCTCTTCGCCGTTGAGCAAATC
GGC---GATCCGACCAAG---AACCGAGAGAAGGGCTCGTTGTGGCTCACCAAACAACATATCG
AACGACTGGGAGAGGATGAGGCGCTGGACCGAGCGCACCCTCAATTTTTCGGGTGGGACG
GCCTGTTAAAAGCCGGTGTCATTGAGTACTTGGATGCAGAAGAAGAAGAGACGTCCATGATT
TGCATGTCACCGGAAGACTTGGAAAACTACAGGCTTCAGAAGGCGGGAGTTGAAGTCGAAG
AGGATAATGATGAAGCCG---ATCCCAACCGCCGTCTCAAGACAAAGATGAACCCAACGACACA
CATGTATACTCATTGTGAGATTCACCCGAGTATGCTTTTGGGGATTTGCGCAAGCATTATCCC?
??
??
??
??

???
???
???
???
???
???
??????????????????????????
>Distoseptispora_chinensis_GZCC21_0665
ACCT--------TC-ATATCT------CGTTGCTTTGGCGGG-CGGCTCC-AGGGCGGAGCCACTGCTCG
TTGGGTCGGGCGCCCGCCGAAGGA---CAAC-CAAAACTCATT-GAATCTTT-GCGGATCTCAGA
GTAATAA-----TAT-----TAAGCTAT-CAAAACTTTCAGCAACGGATCTCTTGGTTCTGGCATCGATG
AAGAACGCAGCGAAATGCGATAAGTAATGTGAATTGCAGAATTCAGTGAATCATCGAATCTTT
GAACGCACATTGCGCCCGCCAGCACTCTGGCGGGCATGCCTGTTCGAGCGTCATTTCAA-CC
CTCGGGCCC-TCGTT-------TGGCCCGGTGTTGGGGCGCTACGGGTCTT-ACT------------------AG
AC-CTGTAGGCCCTTAAAACCAGTGGCGGGCTCGCCAGGACT-CCGAGCGTAGTAGTTT-TCC
T--CTCGTTCAGGCGGTCT-GGCGTG--CT-TCGGCCGTTAAA---TATCCAGC------GGTAATTCG--
CACAC-AAGCCTTCCGAGTCCCC-TGGAAC------GGGGCGCC-AGAGAGGG-TGAGAGCCCCG
TATGGTTGGACGCCGAACC-TCTGTAAAGCTCCTTCGACGAGTCGAGTAGTTTGGGAATGCTG
CTCAAAATGGGAGGTAAATTTCTTCTAAAG-CTAAATACCGGCCAGAGACCGATAGCGCACAA
GTAGAGTGATCGAAAGATGAAAAGCACTTTGAAAAGAGGGTCAAATAGCACGTGAAATTGCT
GAAAGGGAAGCGCTTGTGACCAGACTTGCGCCCGGTTGATCATCCAGCGTTCTCGCTGGTG
CACTCTGCCGGGTTCAGGCCAGCATCGGCTCTCCCAGGGGGATAAAGGTCGCGGGAACGTA
GCTCTCTCCGGGGAGTGTTATAGCCCGTGG-CGGAATGCCCTTAGGGGGGGCCGAGGCTCG
CGTTC-G-CAAGGATGCTGGCGTAATGGTCACCAGCGACCCGTCTT?????????????????????
???
???
???
???
???
???
???
???
???ACTCGTGAGCACGCTCTGCTC
GCCTACACGCTGGGTGTGAAGCAGCTCATCGTCGCCATCAACAAGATGGACACCACCAAGT
GGTCTGAGAGCCGTTACAACGAAATTATCAAGGAGACGTCCAACTTCATCAAGAAGGTCGGC
TACAACCCCAAGCAGGTCGCCTTCGTCCCCATCTCCGGCTTCAACGGCGACAACATGCTTGC
CCCGTCCTCCAACTGCCCCTGGTACAAGGGCTGGGAGAAGGAGATC---AAGGGTGGCAAGG
CCACTGGCAAGACCCTCCTCGAGGCTATTGATGCCATTGAGCAACCCAAGCGCCCCACAGA
CAAGCCTCTCCGTCTCCCCCTTCAGGATGTCTACAAGATTGGTGGTATCGGAACTGTGCCCG
TCGGCCGTATCGAGACTGGTATCCTCAAGCCCGGTATGGTCGTCACGTTCGCTCCCTCAAAC
GTCACCACTGAAGTCAAGTCCGTCGAGATGCACCACGAGCAGCTGACGGAGGGTGTCCCC
GGCGACAACGTTGGTTTCAACGTGAAGAACGTCTCCGTCAAGGAAATCCGCCGAGGCAACG
TCGCTGGTGACTCCAAGAACGATCCTCCTGCTGGTGCCGCTTCCTTCACCGCTCAGGTCATC
GTCCTCAACCACCCCGGCCAGGTCGGTGCTGGTTACGCTCCCGTCTTGGATTGCCACACAG

CCCACATTGCTTGCAAGTTCTCTGAGATCCTCGAGAAGATCGACCGCCGAACGGGCAAGTCT
GTTGAGAACAACCCCAAGTTCATCAAGTCTGGTGACGC
>Distoseptispora_clematidis_KUN_HKAS_112708
ACCT--------TT-ATATT-------CGTTGCTTTGGCGGG-CGGCTCC-AGGGCGGAGCCACTGCTCGT
TGGGTCGGGCGCCCGCCGAAGGA---CAAC-CAAAA-CTCTTTAAATCTTT-GCGGATCTCAGAG
TAAAAA-----TAT-----CAAGCTAT-CAAAACTTTCAGCAACGGATCTCTTGGTTCTGGCATCGATGA
AGAACGCAGCGAAATGCGATAAGTAATGTGAATTGCAGAATTCAGTGAATCATCGAATCTTTG
AACGCACATTGCGCCCGCCAGCACTCTGGCGGGCATGCCTGTTCGAGCGTCATTTCAA-CCC
TCGGGCCC-CCGTT-------TGGCCCGGTGTTGGGGCGCTACGGGTCCCCATC-----------------GG
AC-CTGTAGGCCCTTAAAATTAGTGGCGGGCTCGCCAGGACT-CCGAGCGTAGTAGTTT-TCCT
--CTCGTTCAGGCGGTCT-GGCGTG--CT-TCGGCCGTTAAA---TACCCAGC------GGTAATTCG--C
ACAC-AAGCCTTCCGAGTCCCC-TGGAAC------GGGGCGCC-AGAGAGGG-TGAGAGCCCCGT
ATGGTTGGACGCCGAACC-TCTGTAAAGCTCCTTCGACGAGTCGAGTAGTTTGGGAATGCTG
CTCAAAATGGGAGGTAAATTTCTTCTAAAG-CTAAATACCGGCCAGAGACCGATAGCGCACAA
GTAGAGTGATCGAAAGATGAAAAGCACTTTGAAAAGAGGGTCAAATAGCACGTGAAATTGCT
GAAAGGGAAGCGCTTGTGACCAGACTTGCGCCCGGTTGATCATCCAGCGTTCTCGCTGGTG
CACTCTGCCGGGCTCAGGCCAGCATCGGCTCTCCCAGGGGGATAAAGGTCGCGGGAACGT
AGCTCTCTCCGGGGAGTGTTATAGCCCGTGG-CGGAATGCCCTTAGGGGGGCCGAGGCCC
GCGTTC-G-CAAGGATGCTGGCGTAATGGTCACCAGCGACCCGTCTT???????????????????
??
??
??
??
??
??
??
??
??
??
??
??
??
??
??
??
??
??
??
???
>Distoseptispora_clematidis_MFLUCC_17_2145
ACCT--------TT-ATATT-------CGTTGCTTTGGCGGG-CGGCTCC-AGGGCGGAGCCACTGCTCGT
TGGGTCGGGCGCCCGCCGAAGGA---CAAC-CAAAA-CTCTTTAAATCTTT-GCGGATCTCAGAG
TAAAAA-----TAT-----CAAGCTAT-CAAAACTTTCAGCAACGGATCTCTTGGTTCTGGCATCGATGA
AGAACGCAGCGAAATGCGATAAGTAATGTGAATTGCAGAATTCAGTGAATCATCGAATCTTTG

AACGCACATTGCGCCCGCCAGCACTCTGGCGGGCATGCCTGTTCGAGCGTCATTTCAA-CCC
TCGGGCCC-TCGTT-------CGGCCCGGTGTTGGGGCGCTACGGGTCCCATCG------------------G-
AC-CTGTAGGCCCTTAAAATTAGTGGCGGGCTCGCCAGGACT-CCGAGCGTAGTAGTTT-TCCT
--CTCGTTCAGGCGGTCT-GGCGTG--CT-TCGGCCGTTAAA---AACGCAGC------GGTAAT-TCGC
ACAA----GCCTTCCGAGTCCCC-TGGAAC------GGGGCGCC-AGAGAGGG-TGAGAGCCCCGTA
TGGTTGGACGCCGAACC-TCTGTAAAGCTCCTTCGACGAGTCGAGTAGTTTGGGAATGCTGC
TCAAAATGGGAGGTAAATTCTTTCTAAAG-CTAAATACCGGCCAGAGACCGATAGCGCACAAGT
AGAGTGATCGAAAGATGAAAAGCACTTTGAAAAGAGGGTCAAATAGCACGTGAAATTGCTGA
AAGGGAAGCGCTTGTGACCAGACTTGCGCCCGGTTGATCATCCAGCGTTCTCGMTGGTGCA
CTCTGCCGGGCTCAGGCCAGCATCGGCTCTCCCAGGGGGATAAAGGTCGCGGGAACGTAG
CTCTCTCCGGGGAGTGTTATAGCCCGTGG-CGGAATGCCCTTAGGGGGGGCCGAGGCCCGC
GTTC-G-CAAGGATGCTGGCGTAATGGTCACCAGCGACCCGTCTTTATCGCTCATGTGTTATGT
GAGCGTGGGAACGCCCGCAGAGCCAATCGTCGAGTTCATGATTGCTAGGAACATGGAAGTA
CTCGAGGAATATGAGCCCTTGAGGTACCCTAATGCGACCAAAATCTTTGTCAATGGCACATGG
GTTGGTGTTCACCAAGACCCGAAGCATCTTGTGAGTTTGGTTCAGGACTTACGCCGCAGAGG
CGTCATCAACTTTGAGGTCTCTCTCATTCGGGATATCCGCGATCGAGAGTTCAAGATTTTCTC
GGATGCTGGTCGTGTTATGCGTCCTCTGTTCGCTGTGGAGCAATCGGAC---AATTCGACAAAG
---GGTCTTGAAAAGGGGTCTTTATTGCTCACAAAGGAGCATATTCAAAAATTGAGAGCCGATG
AGGAGTTATCTAAGGATGACCCAGACTATTTCGGCTGGGATGGCCTTCTTAGAGCGGGCGCT
ATCGAATATCTTGACGCCGAAGAGGAAGAGACAGCCATGATTTGCATGACTCCAGAAGATCTA
GAGGCTTATCGGCTGCAAAAAGCGGGATTAGACGTGCCGGAAGACGATGACGAGGCCG---A
CCCCAATAGGCGTCTAAAGACGAAACTAAACCCCACGACTCACATGTATACACATTGTGAGAT
CCATCCGAGCATGCTCTTGGGGATCTGTGCAAGCATTATCCC??????????????????????????
??
??
??
??
??
??
??
??
??
??
??
>Distoseptispora_crassispora_KUMCC_21_10726
ACCT--------TT-ATATTT------CGTTGCTTTGGCGGG-CGGCTCC-AGGGCGGAGCCACTGCTCGT
TGGGTCGGGCGCCCGCCGAAGGA---CAAC-CAAAACTCGTT-GAATCTTT-GCGGATCTCAGA
GTAAAAA-----TAT-----CAAGCTAT-CAAAACTTTCAGCAACGGATCTCTTGGTTCTGGCATCGAT
GAAGAACGCAGCGAAATGCGATAAGTAATGTGAATTGCAGAATTCAGTGAATCATCGAATCTT
TGAACGCACATTGCGCCCGCCAGCACTCTGGCGGGCATGCCTGTTCGAGCGTCATTTCAA-C
CCTCGGGCCC-TCGTT-------TGGCCCGGTGTTGGGGCGCTACAGGTCCCAT-C-----------------G
GAC-CGGTAGGCCCTTAAAATCAGTGGCGGGCTCGCCAGGACT-CCGAGCGTAGTAGTTT-TC
CT--CTCGTTCAGGCGGTCT-GGCGTG--CT-TCGGCCGTTAAA---CACCCAGC------GGTAAT-CG
--CACAC-AAGCCTTCCGAGTCCCC-TGGAAC------GGGGCGCC-AGAGAGGG-TGAGAGCCCC

GTATGGTTGGACGCCGAACC-TCTGTAAAGCTCCTTCGACGAGTCGAGTAGTTTGGGAATGCT
GCTCAAAATGGGAGGTAAATTTCTTCTAAAG-CTAAATACCGGCCAGAGACCGATAGCGCACA
AGTAGAGTGATCGAAAGATGAAAAGCACTTTGAAAAGAGGGTCAAATAGCACGTGAAATTGCT
GAAAGGGAAGCGCTTGTGACCAGACTTGCGCCCGGTTGATCATCCAGCGTTCTCGCTGGTG
CACTCTGCCGGGCTCAGGCCAGCATCGGCTCTCCCAGGGGGATAAAGGTCGCGGGAACGT
AGCTCTCTCCGGGGAGTGTTATAGCCCGTGG-CGGAATGCCCTTAGGGGGGGCCGAGGCCC
GCGTTC-G-CAAGGATGCTGGCGTAATGGTCACCAGCGACCCGTCTTTATCGCTCATGTGTTAT
GTGAGCGTGGGAACGCCCGCAGAGCCAATCGTCGAGTTCATGATTGCTAGGAACATGGAAG
TTCTCGAGGAATATGAGCCCTTGAGGTACCCTAATGCGACCAAAATCTTCGTCAATGGCACAT
GGGTTGGTGTTCACCAAGACCCGAAGCATCTTGTGAGCTTGGTTCAGGACCTACGCCGCAG
AGGCGTCATCAACTTTGAGGTCTCTCTCATTCGGGATATCCGCGATCGAGAGTTCAAGATTTT
CTCGGATGCTGGCCGTGTTATGCGTCCTCTGTTCGCTGTGGAGCAGTCAGAC---AACTCGAC
AAAG---GGTCTTGAGAAGGGGTCTTTATTGCTCACAAAGGAGCATATTCAAAAATTGAGAGCT
GATGAGGAGTTATCCAAGGATGACCCAGATTATTTCGGCTGGGATGGCCTTCTCAGAGCGGG
CGCTATCGAATATCTCGACGCCGAAGAGGAAGAGACAGCTATGATTTGCATGACTCCAGAAGA
TCTAGAGGCTTATCGGCTGCAAAAAGCTGGATTAGACGTGCCGGAAGAAGATGATGAGGCCG
---ACCCCAATAGGCGTCTGAAGACAAAACTGAACCCCACGACTCACATGTATACACATTGTGA
GATCCATCCGAGCATGCTCTTGGGGATCTGTGCGAGCATTATCCCACTCGCGAGCACGCTCT
GCTCGCCTACACGCTGGGTGTGAAGCAGCTCATCGTCGCCATTAACAAGATGGACACCACCA
AGTGGTCTGAGAGCCGTTACAACGAAATTATCAAGGAGACGTCCAACTTCATCAAGAAGGTC
GGCTACAACCCCAAGCAGGTCGCCTTCGTCCCCATCTCCGGCTTCAACGGCGACAACATGC
TTGCCGCATCCTCCAACTGCCCCTGGTACAAGGGCTGGGAGAAGGAGATC---AAGGGTGGC
AAGGCCACTGGCAAGACCCTCCTCGAGGCTATCGACGCCATTGAGCCTCCCAAGCGCCCCA
CAGACAAGCCTCTCCGTCTCCCCCTTCAGGATGTCTACAAGATTGGTGGTATCGGAACTGTG
CCCGTCGGGCGTATCGAGACTGGTATCCTCAAGCCCGGTATGGTCGTCACGTTCGCTCCCTC
CAACGTCACCACTGAAGTCAAGTCCGTTGAGATGCACCACGAGCAGCTGACGGAGGGTGTC
CCCGGCGACAACGTTGGTTTCAACGTGAAGAACGTCTCCGTCAAGGAAATTCGCCGAGGCA
ACGTCGCTGGTGACTCCAAGAACGATCCTCCTGCTGGTGCCGCTTCCTTCACCGCTCAGGT
CATCGTCCTCAACCACCCCGGCCAGGTCGGTGCTGGTTACGCTCCCGTATTGGATTGCCACA
CAGCCCACATTGCTTGCAAGTTCTCTGAGATCCTCGAGAAGATCGACCGCCGAACGGGCAA
GTCTGTTGAGAACAACCCCAAGTTCATCAAGTCTGGTGACGC
>Distoseptispora_curvularia_KUMCC_21_10725
ACCT--------TT-ATATT-------CGTTGCTTTGGCGGG-CGGCTCC-AGGGCGGAGCCACTGCTCGT
TGGGTCGGGCGCCCGCCGAAGGA---CAAC-CAAAACTCGTT-AAATCTTT-GCGGATCTCAGA
GTAAAAA-----TAT-----CAAGCTAT-CAAAACTTTCAGCAACGGATCTCTTGGTTCTGGCATCGAT
GAAGAACGCAGCGAAATGCGATAAGTAATGTGAATTGCAGAATTCAGTGAATCATCGAATCTT
TGAACGCACATTGCGCCCGCCAGCACTCTGGCGGGCATGCCTGTTCGAGCGTCATTTCAA-C
CCTCGGGCCC-TCGTT-------TGGTCCGGTGTTGGGGCGCTACGGGTCCT-GTT-----------------G
GAC-CCGTAGGCCCTCAAAACTAGTGGCGGGCTCGCCAAGACT-CCGAGCGTAGTAGTTT-TC
CT--CTCGTTCAGGCGGTCT-GGCGTG--CT-TCGGCCGTTAAA---TACCCAGC------GGTAATTCG
----CAC-AAGCCTTCCGAGTCCCC-TGGAAC------GGGGCGCC-AGAGAGGG-TGAGAGCCCCG
TATGGTTGGACGCCGAACC-TCTGTAAAGCTCCTTCGACGAGTCGAGTAGTTTGGGAATGCTG
CTCAAAATGGGAGGTAAATTTCTTCTAAAG-CTAAATACCGGCCAGAGACCGATAGCGCACAA
GTAGAGTGATCGAAAGATGAAAAGCACTTTGAAAAGAGGGTCAAATAGCACGTGAAATTGCT

GAAAGGGAAGCGCTTGTGACCAGACTTGCGCCCGGTTGATCATCCAGCGCTCTCGCTGGTG
CACTCTGCCGGGCCCAGGCCAGCATCGGCTCTCCCAGGGGGATAAAGGTCGCGGGAACGT
AGCTCTCTCCGGGGAGTGTTATAGCCCGTGG-CGGAATGCCCTTAGGGGGGGCCGAGGCCC
GCGTTC-G-CAAGGATGCTGGCGTAATGGTCACCAGCGACCCGTCTTTATCGCTCATGTGTTAT
GTGAGCGTGGGAACGCCCGCAGAGCCAATCGTCGAGTTCATGATTGCTAGGAACATGGAAG
TACTCGAGGAATATGAGCCCTTGAGGTACCCTAATGCGACCAAAATCTTCGTCAATGGCACAT
GGGTTGGTGTTCACCAAGACCCGAAGCATCTTGTGAGCTTGGTTCAGGACTTACGCCGCAG
AGGCGTCATCAACTTTGAGGTCTCTCTCATTCGGGATATCCGCGATCGAGAGTTCAAGATATT
CTCGGATGCTGGTCGTGTTATGCGTCCTCTGTTCGCTGTGGAGCAGTCGGAC---AATTCGAC
AAAG---GGTCTTGAAAAGGGGTCTTTATTGCTCACAAAGGAGCATATTCAAAAATTGAGAGCT
GATGAGGAGTTATCCAAGGATGACCCAGACTATTTCGGCTGGGATGGCCTTCTCAGAGCGGG
CGCTATCGAATATCTTGACGCCGAAGAGGAAGAGACAGCCATGATTTGCATGACTCCAGAAG
ATCTAGAGGCTTATCGGCTGCAAAAAGCGGGATTAGACGTGCCGGAAGACGATGACGAGGC
CG---ACCCCAATAGGCGTCTGAAGACGAAACTGAACCCCACGACTCACATGTATACACATTGT
GAGATCCATCCGAGCATGCTCTTGGGGATCTGTGCGAGCATTATCCCACTCGTGAGCACGCT
CTGCTTGCCTACACGCTAGGTGTGAAGCAGCTCATCGTTGCCATCAACAAGATGGACACCAC
CAAGTGGTCTGAGAGCCGTTACAACGAAATTATCAAGGAGACGTCCAACTTCATCAAGAAGG
TCGGCTACAACCCCAAGCAGGTCGCCTTCGTTCCCATCTCCGGCTTCAACGGCGACAACAT
GCTTGCCCCGTCCTCCAACTGCCCCTGGTACAAGGGCTGGGAGAAGGAGATC---AAGGGTG
GCAAGGCCTCTGGCAAGACCCTCCTCGAGGCTATCGATGCCATTGAGCCTCCCAAGCGTCC
CACAGACAAGCCTCTCCGTCTCCCCCTTCAGGATGTCTACAAGATCGGTGGTATCGGAACTG
TGCCCGTCGGCCGTATCGAGACTGGTATCCTCAAGCCCGGTATGGTCGTCACGTTCGCTCCC
TCCAACGTCACCACTGAAGTCAAGTCCGTCGAGATGCACCACGAGCAGCTGCAGGAGGGTG
TCCCCGGCGACAACGTTGGCTTCAACGTGAAGAACGTCTCCGTCAAGGAAATCCGCCGAGG
CAACGTCGCTGGTGACTCCAAGAACGATCCTCCTGCTGGTGCCGCTTCCTTCACCGCCCAG
GTTATCGTCCTCAACCACCCCGGTCAGGTCGGTGCTGGGTACGCTCCCGTCTTGGATTGCC
ACACTGCCCACATTGCCTGCAAGTTCTCCGAGATCCTTGAGAAGATCGACCGCCGAACGGG
CAAGTCTGTTGAGAACAACCCCAAGTTCATCAAGTCTGGTGACGC
>Distoseptispora_cylindricospora_DLUCC_1906
ACCT--------TTTT-GTT-------CGTTGCTTTGGCGGG-CGGCTTC-AGGGTGGAGCCACTGCTCGT
TGGGTCGGGCGTCCGCCAAAGGA---CAAC-CAAAACTCGCT-AAATCAT-GTGTAACGTCAGAG
TATTA-----AAAA-----AAAGCTAT-CAAAACTTTCAGCAACGGATCTCTTGGTTCTGGCATCGATGA
AGAACGCAGCGAAATGCGATAAGTAATGTGAATTGCAGAATTCAGTGAATCATCGAATCTTTG
AACGCACATTGCGCCCGCCAGTACTCTGGCGGGCATGCCTGTTCGAGCGTCATTTCAA-CCC
TCGGGCCCCTCTGT-----T-TGGCCCGGTGTTGGGGTTCTAC-GGTCCGGC--------------------GG
ATTGGTAGGCCCTTGAAATCAGTGGCGGGCTCGCCAGGGCT-CCGAGCGCAGTAGTTC-TCT
C--CTCGCTCAGGCGGCCT-GGCGTG--CT-TCGGCCGTTAAA---CACCCG-C------TGTATTTTT--
TACAG-AAGCCTTCCGAGTCCCC-TGGAAC------GGGGCGCC-AGAGAGGG-TGAGAGCCCCG
TATGGTTGGACGCCGAGCC-TCTGTAAAGCTCCTTCGACGAGTCGAGTAGTTTGGGAATGCT
GCTCAAAATGGGAGGTAAATTTCTTCTAAAG-CTAAATACCGGCCAGAGACCGATAGCGCACA
AGTAGAGTGATCGAAAGATGAAAAGCACTTTGAAAAGAGGGTTAAATAGCACGTGAAATTGCT
GAAAGGGAAGCGCTTGTGACCAGACTTGCGCCCGGTTGATCATCCAGCGTTCTCGCTGGTG
CACTCTGCCGGGCTCAGGCCAGCATCGGCTCTCCCAGGGGGATAAAGGTCGCGGGAACGT
AGCTCTCTTCGGGGAGTGTTATAGCCCGTGG-CGGAATACCTTTAGGGGGGGCCGAGGCCC

GCG-TTCG-CAAGGATGCTGGCGTAATGGTCACCAGCGACCCGTCTT???????????????????
??
??
??
??
??
??
??
??
??ACTCGAGAGCACGCTCTGC
TCGCCTACACTCTGGGTGTGAAGCAGCTCATTGTCGCTATCAACAAGATGGACACCACCAAA
TGGTCTGAGAGCCGTTTCAACGAAATCATCAAGGAGACGTCCAACTTCATCAAGAAGGTCGG
CTACAACCCCAAGACAGTCGCCTTCGTTCCCATCTCCGGCTTCAACGGCGACAACATGCTTG
CTGCCTCCCCCAACTGCCCCTGGTACAAGGGCTGGGAGAAGGAGATC---AAGGGCGGCAAG
GTCAGTGGCAAGACGCTCCTCGAGGCCATCGATGCCATTGAGCAACCCAAGCGTCCCACAG
ACAAGCCTCTGCGTCTCCCCCTTCAGGATGTCTACAAGATTGGTGGTATCGGAACTGTACCT
GTCGGCCGTATCGAGACTGGTATCATCAAGCCGGGTATGGTCGTTACGTTCGCTCCCTCCAA
CGTCACCACTGAAGTCAAGTCCGTCGAGATGCACCACGAGCAGTTGACAGAGGGTGTTCCC
GGCGACAACGTCGGCTTCAACGTGAAGAACGTCTCCGTCAAGGAAATTCGCCGAGGCAACG
TCGCTGGTGACTCCAAGAACGATCCTCCTGCTGCTGCCGCTTCCTTCACCGCCCAGGTCATC
GTCCTCAACCACCCCGGTCAAGTTGGTGCTGGCTACGCTCCAGTCTTGGATTGCCACACTG
CCCACATCGCTTGCAAGTTCGCCGAGATCCTCGAGAAGATCGATCGTCGAACGGGCAAGTC
CGTCGAGAACAACCCCAAGTTCATCAAGTCTGGTGATGC
>Distoseptispora_dehongensis_KUMCC_18_0090
ACCT--------ATT--ATT-------CGTTGCTTCGGCGGG-CGGCTCC-AGGGCGGAGCCACTGCCCGT
TGGGTCGGGCGCCCGCCGGAGGA---CACC-TAAAACTCGCA-TGATCTC-GGGTATCGTCAGA
GTTCTACAAGAAAAA-----ATAGCTAT-CAAAACTTTCAGCAACGGATCTCTTGGTTCTGGCATCG
ATGAAGAACGCAGCGAAATGCGATAAGTAATGTGAATTGCAGAATTCAGTGAATCATCGAATCT
TTGAACGCACATTGCGCCCGCCAGTACTCTGGCGGGCATGCCTGTTCGAGCGTCATTTCAA-
CCCTCGGGCCCTACTGT-----TCTGGCCCGGTGCTGGGGTGCTAC-GGTCCGCT------------------
--GGACCCGTAGGCCCTCGAAACCAGTGGCGGGGTCGTCTGGTCT-CCGAGCGCAGTAGTTC-
TCTC--CTCGCTCAGGCGGCCC-GGCGCG--CC-TCGGCCGTTAAA---CACCCA-C------TGTACT
T-----ACAG-AAGCCTTCCGAGTCCCC-TGGAAC------GGGGCGCC-AGAGAGGG-TGAGAGCCC
CGTATGGTTGGACGCCGAGCC-TCTGTAAAGCTCCTTCGACGAGTCGAGTAGTTTGGGAATG
CTGCTCAAAATGGGAGGTAAATTTCTTCTAAAG-CTAAATACCGGCCAGAGACCGATAGCGCA
CAAGTAGAGTGATCGAAAGATGAAAAGCACTTTGAAAAGAGGGTTAAATAGCACGTGAAATTG
CTGAAAGGGAAGCGCTTGTGACCAGACTTGCGCCCGGCTGATCATCCAGCGTTCTCGCTGG
TGCACTCTGCCGGGCTCAGGCCAGCATCGGCTCTCCCAGGGGGATAAAGGTCGCGGGAAC
GTAGCTCCCTCCGGGGAGTGTTATAGCCCGCGG-CGGAATGCCCTTAGGGGGGGCCGAGGC
CCGCGTTC-G-CAAGGATGCTGGCGTAATGGTCACCAGCGACCCGTCTT?????????????????
??
??
??
??

??
??
??
??
???ACCCGCGAGCACGCTCT
GCTTGCCTACACCCTGGGTGTGAAGCAGCTCATCGTCGCCATCAACAAGATGGACACCACCA
AGTGGTCTGAGGATCGTTACAACGAAATCATCAAGGAGACATCCAACTTCATCAAGAAGGTG
GGCTACAACCCGAAGCAGGTCGCCTTCGTCCCCATCTCCGGCTTCAACGGCGACAACATGC
TTGCCCCCTCCTCCAACTGCCCCTGGTACAAGGGCTGGGAGAAGGAGGTC---AAGGGCGGC
AAGGTCACTGGCAAGACCCTCCTCGAAGCCATCGACGCCATTGAGCCTCCCAAGCGCCCTA
CAGACAAGCCCCTGCGTCTCCCCCTTCAGGATGTCTACAAGATTGGTGGTATCGGAACTGTA
CCTGTCGGCCGTATCGAGACTGGTATCCTCAAGCCGGGTATGGTCGTCACGTTCGCTCCCTC
CAACGTCACCACTGAAGTGAAGTCCGTCGAGATGCACCACGAGCAGCTCCAGGAGGGCGTT
CCTGGCGACAACGTCGGCTTCAACGTGAAGAACGTCTCCGTCAAGGAAATCCGCCGAGGCA
ACGTCGCCGGTGACTCCAAGAACGATCCTCCTGCCGGTGCCGCTTCCTTCACTGCCCAGGT
CATCGTCCTCAACCACCCCGGTCAGGTTGGTGCTGGTTACGCCCCTGTCTTGGATTGCCACA
CTGCACACATCGCCTGCAAGTTCTCCGAGATCCTCGAGAAGATCGATCGACGAACGGGCAA
GTCCGTCGAGAACAACCCCAAGTTCATCAAGTCTGGTGACGC
>Distoseptispora_dipterocarpi_MFLUCC_22_0104
ACCT--------TT-ATATT-------CGTTGCTTTGGCGGG-CGGCTCC-AGGGCGGAGCCACTGCTCGT
TGGGTCGGGCGCCCGCCGAAGGA---CAAC-CAAAA-CTCTTTAAATCTTT-GCGGATCTCAGAG
TAAAAA-----TAT-----CAAGCTAT-CAAAACTTTCAGCAACGGATCTCTTGGTTCTGGCATCGATGA
AGAACGCAGCGAAATGCGATAAGTAATGTGAATTGCAGAATTCAGTGAATCATCGAATCTTTG
AACGCACATTGCGCCCGCCAGCACTCTGGCGGGCATGCCTGTTCGAGCGTCATTTCAA-CCC
TCGGGCCC-TCGTT-------TGGCCCGGTGTTGGG---
--GCCTT
CCGAGTCCCC-TGGAAC------GGGGCGCC-AGAGAGGG-TGAGAGCCCCGTATGGTTGGACG
CCGAACC-TCTGTAAAGCTCCTTCGACGAGTCGAGTAGTTTGGGAATGCTGCTCAAAATGGG
AGGTAAATTTCTTCTAAAG-CTAAATACCGGCCAGAGACCGATAGCGCACAAGTAGAGTGATC
GAAAGATGAAAAGCACTTTGAAAAGAGGGTCAAATAGCACGTGAAATTGCTGAAAGGGAAGC
GCTTGTGACCAGACTTGCGCCCGGTTGATCATCCAGCGTTCTCGCTGGTGCACTCTGCCGG
GCTCAGGCCAGCATCGGCTCTCCCAGGGGGATAAAGGTCGCGGGAACGTAGCTCTCTCCG
GGGAGTGTTATAGCCCGTGG-CGGAATGCCCTTAGGGGGGGCCGAGGCCCGCGTTC-G-CAA
GGATGCTGGCGTAATGGTCACCAGCGACCCGTCTTTATCGCTCATGTGTTATGTGAGCGTGG
GAACGCCCGCAGAGCCAATCGTCGAGTTCATGATTGCTAGGAACATGGAAGTACTCGAGGAA
TATGAGCCCTTGAGGTACCCTAATGCGACCAAAATCTTCGTCAATGGCACATGGGTTGGTGTT
CACCAAGACCCGAAGCATCTTGTGAGCTTGGTTCAGGACTTACGCCGCAGAGGCGTCATCA
ACTTTGAGGTGTCTCTAATTCGGGATATCCGCGATCGAGAGTTCAAGATTTTCTCGGATGCTG
GTCGTGTTATGCGTCCTCTGTTCGCTGTGGAGCAGTCGGAC---AATTCGACAAAG---GGTCTT
GAAAAGGGGTCTTTATTGCTCACAAAGGAGCATATTCAAAAATTGAGAGCTGATGAGGAGTTA
TCCAAGGATGACCCAGACTATTTCGGCTGGGATGGCCTTCTCAGAGCGGGCGCTATCGAATA
TCTTGACGCTGAAGAGGAAGAGACAGCCATGATTTGCATGACTCCAGAAGATCTAGAGGCTT
ATCGGCTGCAAAAAGCGGGATTAGACGTGCCGGAAGACGATGACGAGGCCG---ACCCCAATA
GGCGTCTGAAGACGAAACTGAACCCCACGACTCACATGTATACACATTGTGAGATCCATCCAA

GCATGCTCTTGGGGATCTGTGCGAGCATTATCCC??
???
???
???
???
???
???
???
???
???
???
>Distoseptispora_effusa_GZCC19_0532
ACCT--------GTC--GTT-------CGTTGCTTCGGCGGG-CGGCTCC-AGGGCGGAGCCACTGCCCG
TTAGGTCGGGCGCCCGCCGGAGGA---CACA-CAAAACTCGTC--GATCTA-GTGTATCATCAGA
GTATTA----AAAAA-----CAAGCTAT-CAAAACTTTCAGCAACGGATCTCTTGGTTCTGGCATCGAT
GAAGAACGCAGCGAAATGCGATAAGTAATGTGAATTGCAGAATTCAGTGAATCATCGAATCTT
TGAACGCACATTGCGCCCGCCAGTACTCTGGCGGGCATGCCTGTTCGAGCGTCATTTCAA-C
CCTCGGGCCCCC--GT-----T-TGGCCCGGTGTTGGGGTGCTACAGGTCCTTCG--------------------
GGACCTGTAGGCCCTCAAAACCAGTGGCGGGCTCGCCATGGCTTCCGAGCGCAGTAGTTG-
TCTC--CTCGCTCAGGTGGTCT-GGCGTG--CC-TCGGCCGTTAAA---CACCCG-C------TGTATAC-
----ACAG-AAGCCTTCCGAGTCCCC-TGGAAC------GGGGCGCC-AGAGAGGG-TGAGAGCCCC
GTATGGTCGGACGCCGAACC-TTTGTAAAGCTCCTTCGACGAGTCGAGTAGTTTGGGAATGCT
GCTCAAAATGGGAGGTAAATTTCTTCTAAAG-CTAAATACCGGCCAGAGACCGATAGCGCACA
AGTAGAGTGATCGAAAGATGAAAAGCACTTTGAAAAGAGGGTTAAATAGCACGTGAAATTGCT
GAAAGGGAAGCGCTTGTGACCAGACTTGCGCCCGGTTGATCATCCAGCGTTCTCGCTGGTG
CACTCTGCCGGGCTCAGGCCAGCATCGGCTCCCCCAGGGGGATAAAGGTCGCGGGAACGT
AGCTCTCTCCGGGGAGTGTTATAGCCCGTGG-CGGAATACCCTTAGGGGGGGTCGAGGCCC
GCGTTC-G-CAAGGATGCTGGCGTAATGGTCACCAGCGACCCGTCTT??????????????????????
???
???
???
???
???
???
???
???
??ACTCGTGAGCACGCCCTGC
TTGCCTACACCCTAGGTGTCAAGCAGCTTATCGTCGCCATCAACAAGATGGACACCACCAAG
TGGTCTGAAGATCGTTACAACGAAATCATCAAGGAGACGTCCAACTTCATCAAGAAGGTCGG
ATACAACCCCAAGCAGGTGGCTTTCGTTCCCATCTCCGGCTTCAACGGTGACAACATGTTGG
CGCCGTCCACCAACTGCCCCTGGTACAAGGGCTGGGAGAAGGAGATC---AAGGGTAACAAG
ATCACTGGCAAGACGCTCCTCGAGGCCATCGATGCCATTGAGCAACCCAAGCGTCCCACAG
ACAAGCCTCTCCGTCTTCCCCTTCAGGATGTCTACAAGATCGGTGGTATTGGCACTGTGCCC
GTCGGCCGTATCGAGACTGGTGTCCTCAAGCCCGGTATGGTCGTTACATTCGCTCCCTCCAA

CGTCACCACTGAAGTCAAGTCCGTCGAGATGCACCACGAGCAGCTCGCCGAGGGTATGCCT
GGTGACAACGTTGGCTTCAACGTGAAGAACGTCTCCGTCAAGGAAATCCGCCGTGGCAACG
TCGCTGGTGACTCCAAGAACGATCCTCCTGCCGGTGCTGCTTCCTTCACTGCCCAGGTCATC
GTCCTCAACCACCCTGGTCAGGTCGGCGCTGGCTACGCCCCCGTCTTGGATTGCCACACCG
CCCACATCGCCTGCAAGTTCTCTGAGATCCTCGAGAAGATTGACCGCCGAACGGGCAAGTC
TGTCGAGAACAACCCCAAGTTCATCAAGTCTGGTGACGC
>Distoseptispora_euseptata_DLUCC_S2024
ACCT--------TTTGTCAA-------TGTTGCTTCGGCGGG-TGGTCCC-AGGGCGGGACCACAGCCCA
TTCAGTTGGGTGCCCGCCGGAGGA---CTGTTCAAAAACTCTTATC----ATAGGTATTATCTGAGT
CTA------AAACA--AAAT--CTAT-AAAAACTTTCAACAACGGATCTCTTGGTTCTGGCATCGATGAA
GAACGCAGCGAAATGCGATAAGTAATGTGAATTGCAGAATTCAGTGAATCATCGAATCTTTGA
ACGCACATTGCGCCCGCCAGCACTCTGGCGGGCATGCCTGTTCGAGCGTCATTTCA-ACCCT
CGGGCCC-CC-------GT--GGCCTGGTGTTGGGGCACTGCCGTCTAG----------------------GGACG
CGCAGGCCCTGAAAAACAGTGGCGGGCTCGCTAGAAC-TCCGGGCGCAGTAG--ATTAA-ATC
TCGTCTAGGAGGTCT-GGCGCG--GT-CTTGCCGTCAAAC-CCCACT--------A-TCATAC-------AA
CAGCCTTCTAAGTCCCC-TAGAAC------GGGGCGCC-ATAGAGGG-TGAGAGCCCCGTACAGTC
GGACACCGAACC-TTTGTAAAGCTCCTTCGACGAGTCGAGTAGTTTGGGAATGCTGCTCAAAA
TGGGAGGTAAATTTCTTCTAAAG-CTAAATACCGGCCAGAGACCGATAGCGCACAAGTAGAGT
GATCGAAAGATGAAAAGCACTTTGAAAAGAGGGTTAAATAGCACGTGAAATTGTTGAAAGGGA
AGCGCTTGTGACCAGACTTGCGCCCGGTGAATCATCCAGCGTTCTCGCTGGTGCACTTTGC
CGGGCTCAGGCCAGCATCGGTTCTCCCAGGGGGATAAAAGTCTCGGGAACGTAGCTCTCTT
CGGGGAGTGTTATAGCCCGTGG-CACAATACC-CTTGGGGGGACCGAGGACCGCGCTCTG-C
AAGGATGCTGGCGTAATGGTCATCAGCGACCCGTCTTTATCTCTCATGTGTTATGTCAGCGTT
GGCACACCAGCAGAACCGATCATCGAGTTCATGATCGCGAGGAATATGGAAGTCCTAGAAGA
ATATGAGCCTTTGCGCTACCCCAATGCGACCAAGATATTCGTTAACGGAACTTGGGTCGGTGT
CCACCAGGATCCCAAACATCTGGTTGGGCTAGTACAGGAGTTGCGACAGAGAGGTGTCATCA
ACTTTGAAGTCTCTCTTGTCCGTGATATCCGAGACAGGGAGTTCAAGATTTTTTCTGACGCTG
GTCGAGTGATGCGGCCCCTCTTCGCTGTGGAGCAAGAGGAT---AACTCTAAGTCC---GGTCTT
CCAAAAGGGGCTCTGAGGCTCACCAAAGAGCACATTCAGAGACTCAGGGCCGATGAAGAAT
TATCCAAAGACGACCCAGAATATTTCGGCTGGGACGGCATCGTTGAGGCCGGCGCCGTCGA
GTATCTCGACGCGGAAGAAGAGGAAACGGCTATGATTTGCATGACGCCAGAAGATTTGGAAA
CATACCGACTCCAGAAGGCCGGCCTGGGTATAGCGGAAGACGACAAAGGAACGG---ACCCG
AATAGAC--ACCCGAGAGC
ACGCTCTGCTTGCCTACACTTTGGGTGTCAAGCAGATTATCTGCGCCATCAACAAGATGGACA
CCACCAAGTGGTCAGAGGACCGTTTCAACGAAATCATCAAGGAGACGTCCAACTTCATCAAG
AAGGTCGGTTACAACCCCAAGCAGGTCGCCTTCGTGCCCATCTCCGGTTTCCACGGCGACA
ACATGCTTGCTCCCTCTACCAACTGCCCCTGGTACAAGGGCTGGGAGAAGGAGGGCGCCAA
GGGTGCCAAGTCTTCTGGCAAGACCCTGCTTGAGGCCATCGACGCCATTGAGCCCCCGAAG
CGTCCCACAGACAAGCCCCTGCGTCTCCCGCTCCAGGATGTCTACAAGATCGGCGGTATCG
GAACGGTCCCTGTCGGCCGTATCGAGACTGGTGTCCTGAAGCCCGGTATGGTCGTCACTTT
CGCTCCCTCCAACGTCACCACTGAAGTCAAGTCCGTCGAGATGCACCACGAGCAGCTGACT
GAGGGTGTCCCTGGTGACAACGTCGGCTTCAACGTGAAGAACGTGTCCGTCAAGGAAATCC
GCCGTGGCAACGTTGCCGGTGATTCCAAGAACGACCCCCCTGCCGGCTGCGCCAACTTCAC
TGCCCAGGTCATTGTCCTCAACCACCCTGGTCAGGTTGGCCCTGGTTACGCTCCCGTCCTG

GACTGCCACACCGCTCACATTGCCTGCAAGTTCTCCGAGATCACCGAGAAGATCGACCGCC
GTACCGGCAAGTCGGTTGAGAACAACCCCAAGTTCATTAAGTCTGGTGACGC
>Distoseptispora_euseptata_MFLUCC_20_0154
ACCT--------TTTGTCAA-------TGTTGCTTCGGCGGG-TGGTCCC-AGGGCGGGACCACAGCCCA
TTTAGTTGGGTGCCCGCCGGAGGA---CTGCTCAAAAACTCTCATC----ATAGGTATTATCTGAGT
CTA------AAACA--AAAT--CTAT-AAAAACTTTCAACAACGGATCTCTTGGTTCTGGCATCGATGAA
GAACGCAGCGAAATGCGATAAGTAATGTGAATTGCAGAATTCAGTGAATCATCGAATCTTTGA
ACGCACATTGCGCCCGCCAGCACTCTGGCGGGCATGCCTGTTCGAGCGTCATTTCA-ACCCT
CGGGCCC-CC-------GT--GGCCTGGTGTTGGGGCACTGCCGTCTAG----------------------GGACG
CGCAGGCCCTGAAAAACAGTGGCGGGCTCGCTAGAAC-TCCGGGCGCAGTAG--ATTAA-ATC
TCGTCTAGGTGGTCT-GGCGCG--GT-CTTGCCGTCAAAAC-CCCACT--------A-TCATAC-------AA
CAGCCTTCTAAGTCCCC-TAGAAC------GGGGCGCC-ATAGAGGG-TGAGAGCCCCGTACAGTC
GGACACCGAACC-TTTGTAAAGCTCCTTCGACGAGTCGAGTAGTTTGGGAATGCTGCTCAAAA
TGGGAGGTAAATTTCTTCTAAAG-CTAAATACCGGCCAGAGACCGATAGCGCACAAGTAGAGT
GATCGAAAGATGAAAAGCACTTTGAAAAGAGGGTTAAATAGCACGTGAAATTGTTGAAAGGGA
AGCGCTTGTGACCAGACTTGCGCCCGGTGAATCATCCAGCGTTCTCGCTGGTGCACTTTGC
CGGGCTCAGGCCAGCATCGGTTCTCCCAGGGGGATAAAAGTCTCGGGAACGTAGCTCTCTT
CGGGGAGTGTTATAGCCCGTGG-CACAATACC-CTTGGGGGGACCGAGGACCGCGCTCTG-C
AAGGATGCTGGCGTAATGGTCATCAGCGACCCGTCTTTATCTCTCATGTGTTATGTCAGCGTT
GGCACACCAGCAGAACCGATCATCGAGTTCATGATCGCGAGGAATATGGAAGTCCTAGAAGA
ATATGAGCCTTTGCGCTACCCCAATGCGACCAAGATATTCGTTAACGGAACTTGGGTCGGTGT
CCACCAGGATCCCAAACATCTGGTTGGGCTAGTACAGGAGTTGCGACAGAGAGGTGTCATCA
ACTTTGAAGTCTCTCTTGTCCGTGATATCCGAGACAGGGAGTTCAAGATTTTTTCTGACGCTG
GTCGAGTGATGCGGCCCCTCTTCGCTGTGGAGCAAGAGGAT---AACTCTAAGTCC---GGTCTT
CCAAAAGGGGCTCTGAGGCTCACCAAAGAGCACATTCAGAGACTCAGGGCCGATGAAGAAT
TATCCAAAGACGACCCAGAATATTTCGGCTGGGACGGCATCGTTGAGGCCGGCGCCGTCGA
GTATCTCGACGCGGAAGAAGAGGAAACGGCTATGATTTGCATGACGCCAGAAGATTTGGAAA
CATACAGACTCCAGAAGGCCGGCCTGGGTATAGCGGAAGACGACGAAGAAGCGG---ACCCG
AATAGACGTTTAAAAACGAAGTTAAACCCTACCACTCACATGTACACGCACTGCGAGATCCAT
CCAAGCATGCTCTTAGGGATTTGCGCAAGCATTATACC??????????????????????????????
??
??
??
??
??
??
??
??
??
??
>Distoseptispora_fasciculata_KUMCC_19_0081
ACCT--------CT-ATATT-------CGTTGCTTTGGCGGG-CGGCTCC-AGGGCGGAGCCACTGCTCGT
TGGGTCGGGCGCCCGCCGAAGGA---CAAC-CAAAGCTCATT-GAATCTGT-GCGGATCTCAGA
GTAAAAA-----TAT-----CAAGCTAT-CAAAACTTTCAGCAACGGATCTCTTGGTTCTGGCATCGAT

GAAGAACGCAGCGAAATGCGATAAGTAATGTGAATTGCAGAATTCAGTGAATCATCGAATCTT
TGAACGCACATTGCGCCCGCCAGCACTCTGGCGGGCATGCCTGTTCGAGCGTCATTTCAA-C
CCTCGGGCCC-TCGTT-------TGGCCCGGTGTTGGGGCGCTACGGGTCCC-GTC-----------------
GGAC-CTGTAGGCCCTCAAAACCAGTGGCGGGCTCGCCAGGACT-CCGAGCGCAGTAGTTC-
TCCT--CTCGCTCAGGCGGCCT-GGCGTG--CT-TCGGCCGTTAAA---TACCCAGC------GGTAAT
CCG----CAT-AAGCCTTCCGAGTCCCC-TGGAAC------GGGGCGCC-AGAGAGGG-TGAGAGCC
CCGTATGGTTGGACGCCGAACC-TCTGTAAAGCTCCTTCGACGAGTCGAGTAGTTTGGGAAT
GCTGCTCAAAATGGGAGGTAAATTTCTTCTAAAG-CTAAATACCGGCCAGAGACCGATAGCGC
ACAAGTAGAGTGATCGAAAGATGAAAAGCACTTTGAAAAGAGGGTCAAATAGCACGTGAAATT
GCTGAAAGGGAAGCGCTTGTGACCAGACTTGCGCCCGGTTGATCATCCAGCGTTCTCGCTG
GTGCACTCTGCCGGGCTCAGGCCAGCATCGGCTCTCCCAGGGGGATAAAGGTCGCGGGAA
CGTAGCTCTCTCCGGGGAGTGTTATAGCCCGTGG-CGGAATGCCCTTAGGGGGGGCCGAGG
CCCGCGTTC-G-CAAGGATGCTGGCGTAATGGTCACCAGCGACCCGTCTT???????????????
???
???
???
???
???
???
???
???
??ACTCGTGAGCACGCTC
TGCTCGCCTACACACTTGGTGTGAAGCAGCTCATCGTCGCCATCAACAAGATGGACACCACC
AAGTGGTCCGAGAGCCGTTACAACGAAATCATCAAGGAGACGTCCAACTTCATCAAGAAGGT
CGGCTACAACCCCAAGCAGGTCGCCTTCGTTCCCATCTCCGGCTTCAACGGCGACAACATG
CTTGCCCCCTCCTCCAACTGCCCCTGGTACAAGGGTTGGGAGAAGGAGATC---AAGGGTGG
CAAGGTCTCTGGCAAGACCCTCTTGGAGGCTATCGATGCCATTGAGCCTCCCAAGCGTCCCA
CAGACAAGCCTCTCCGTCTACCCCTTCAGGATGTCTACAAGATCGGTGGTATCGGAACTGTG
CCTGTCGGCCGTATCGAGACTGGTATCCTCAAGCCCGGTATGGTCGTCACATTCGCTCCCTC
CAACGTCACCACTGAAGTCAAGTCCGTCGAGATGCACCACGAGCAGCTGCAGGAGGGTGTC
CCCGGCGACAACGTCGGCTTCAACGTGAAGAACGTCTCCGTCAAGGAAATCCGCCGAGGCA
ACGTCGCTGGTGACTCCAAGAACGACCCCCCTGCCGGTGCCGCTTCCTTCACCGCTCAGGT
CATCGTCCTCAATCACCCCGGTCAGGTCGGTGCTGGCTACGCTCCCGTCTTGGATTGCCACA
CTGCCCACATCGCTTGCAAGTTCTCTGAGATCCTTGAGAAGATCGACCGCCGAACGGGCAA
GTCTGTTGAGAACAACCCCAAGTTCATCAAGTCTGGTGACGC
>Distoseptispora_fluminicola_DLUCC_0391
ACCT--------TT-ATATT-------CGTTGCTTTGGCGGG-CGGCTCC-AGGGCGGAGCCACTGCTCGT
TGGGTCGGGCGCCCGCCGAAGGA---CAGC-TAAAACCCGTT-AAATCTTT-GCGGATCTCAGA
GTAAAAA-----TAT-----CAAGCTAT-CAAAACTTTCAGCAACGGATCTCTTGGTTCTGGCATCGAT
GAAGAACGCAGCGAAATGCGATAAGTAATGTGAATTGCAGAATTCAGTGAATCATCGAATCTT
TGAACGCATATTGCGCCCGCCAGCACTCTGGCGGGCATGCCTGTTCGAGCGTCATTTCAA-C
CCTCGGGCCC-TCGTT-------TGGCCCGGTGTTGGGGTGCTACGGGTTCT-ATT-----------------G
GAC-CCGTAGGCCCTCAAAACCAGTGGCGGGCTCGCCAGGACT-CCGAGCGCAGTAGTTC-T
CCT--CTCGCTCAGGTGGCCT-GGCGTG--CT-TCGGCCGTTAAA---CATCCAGC------GGTAATTC

G----CAC-AAGCCTTCCGAGTCCCC-TGGAAC------GGGGCGCC-AGAGAGGG-TGAGAGCCCC
GTATGGTTGGACGCCGAACC-TCTGTAAAGCTCCTTCGACGAGTCGAGTAGTTTGGGAATGCT
GCTCAAAATGGGAGGTAAATTTCTTCTAAAG-CTAAATACCGGCCAGAGACCGATAGCGCACA
AGTAGAGTGATCGAAAGATGAAAAGCACTTTGAAAAGAGGGTCAAATAGCACGTGAAATTGCT
GAAAGGGAAGCGCTTGTGACCAGACTTGCGCCCGGTTGATCATCCAGCGTTCTCGCTGGTG
CACTCTGCCGGGCTCAGGCCAGCATCGGCTCTCCCAGGGGGATAAAGGTCGCGGGAACGT
AGCTCTCTCCGGGGAGTGTTATAGCCCGTGG-CGGAATGCCCTTAGGGGGGGCCGAGGCCC
GCGTTC-G-CAAGGATGCTGGCGTAATGGTCACCAGCGACCCGTCTT???????????????????
???
???
???
???
???
???
???
???
???ACTCGTGAGCACGCTCTGC
TTGCCTACACGCTAGGTGTGAAGCAGCTCATCGTTGCCATCAACAAGATGGACACCACCAAG
TGGTCTGAGAGCCGTTACAACGAAATTATCAAGGAGACGTCCAACTTTATCAAGAAGGTCGG
CTACAACCCCAAGCAGGTCGCCTTCGTTCCCATCTCCGGCTTCAACGGCGACAACATGCTTG
CCCCGTCCTCCAACTGCCCCTGGTACAAGGGTTGGGAGAAGGAGATC---AAGGGTGGCAAG
GCCTCTGGCAAGACCCTTCTCGAGGCTATCGATGCCATTGAGCAACCCAAGCGTCCCACAG
ACAAGCCTCTCCGTCTCCCTCTTCAGGATGTCTACAAGATTGGTGGTATCGGAACTGTGCCC
GTCGGCCGTATCGAAACTGGTATCCTCAAGCCGGGTATGGTCGTCACGTTCGCTCCCTCCAA
CGTCACCACTGAAGTCAAGTCCGTCGAGATGCACCACGAGCAGCTGCAGGAGGGTGTCCC
CGGCGACAACGTTGGCTTCAACGTGAAGAACGTCTCCGTCAAGGAAATCCGCCGAGGCAAC
GTCGCTGGTGACTCCAAGAACGATCCTCCTGCTGCCGCCGCTTCCTTCACCGCTCAGGTCA
TCGTCCTCAACCACCCCGGTCAGGTCGGTGCTGGTTACGCTCCCGTCCTGGATTGCCACAC
TGCCCACATTGCCTGCAAGTTCTCTGAGATCCTTGAGAAGATCGACCGCCGAACGGGCAAGT
CTGTTGAGAACAACCCCAAGTTCATCAAGTCTGGTGACGC
>Distoseptispora_fluminicola_DLUCC_0999
ACCT--------TT-ATATT-------CGTTGCTTTGGCGGG-CGGCTCC-AGGGCGGAGCCACTGCTCGT
TGGGTCGGGCGCCCGCCGAAGGA---CAGC-TAAAACCCGTT-AAATATTT-GCGGATCTCAGAG
TAAAAA-----TAT-----CAAGCTAT-CAAAACTTTCAGCAACGGATCTCTTGGTTCTGGCATCGATGA
AGAACGCAGCGAAATGCGATAAGTAATGTGAATTGCAGAATTCAGTGAATCATCGAATCTTTG
AACGCATATTGCGCCCGCCAGCACTCTGGCGGGCATGCCTGTTCGAGCGTCATTTCAA-CCC
TCGGGCCC-TCGTT-------TGGCCCGGTGTTGGGGTGCTACGGGTTCT-ATT-----------------GGA
C-CCGTAGGCCCTCAAAACCAGTGGCGGGCTCGCCAGGACT-CCGAGCGCAGTAGTTC-TCC
T--CTCGCTCAGGCGGCCT-GGCGTG--CT-TCGGCCGTTAAA---TATCCAGC------GGTAATTCG--
--CAC-AAGCCTTCCGAGTCCCC-TGGAAC------GGGGCGCC-AGAGAGGG-TGAGAGCCCCGT
ATGGTTGGACGCCGAACC-TCTGTAAAGCTCCTTCGACGAGTCGAGTAGTTTGGGAATGCTG
CTCAAAATGGGAGGTAAATTTCTTCTAAAG-CTAAATACCGGCCAGAGACCGATAGCGCACAA
GTAGAGTGATCGAAAGATGAAAAGCACTTTGAAAAGAGGGTCAAATAGCACGTGAAATTGCT
GAAAGGGAAGCGCTTGTGACCAGACTTGCGCCCGGTTGATCATCCAGCGTTCTCGCTGGTG

CACTCTGCCGGGCTCAGGCCAGCATCGGCTCTCCCAGGGGGATAAAGGTCGCGGAACGT
AGCTCTCTCCGGGGAGTGTTATAGCCCGTGG-CGGAATGCCCTTAGGGGGGGCCGAGGCCC
GCGTTC-G-CAAGGATGCTGGCGTAATGGTCACCAGCGACCCGTCTT????????????????????
??
??
??
??
??
??
??
??
??ACTCGTGAGCACGCTCTGC
TTGCCTACACGCTAGGTGTGAAGCAGCTCATCGTTGCCATCAACAAGATGGACACCACCAAG
TGGTCTGAGAGCCGTTACAACGAAATTATCAAGGAGACGTCCAACTTTATCAAGAAGGTCGG
CTACAACCCCAAGCAGGTCGCCTTCGTTCCCATCTCCGGCTTCAACGGCGACAACATGCTTG
CCCCGTCCTCCAACTGCCCCTGGTACAAGGGTTGGGAGAAGGAGATC---AAGGGTGGCAAG
GCCTCTGGCAAGACCCTTCTCGAGGCTATCGATGCCATTGAGCAACCCAAGCGTCCCACAG
ACAAGCCTCTCCGTCTCCCTCTTCAGGATGTCTACAAGATTGGTGGTATCGGAACTGTGCCC
GTCGGCCGTATCGAAACTGGTATCCTCAAGCCGGGTATGGTCGTCACGTTCGCTCCCTCCAA
CGTCACCACTGAAGTCAAGTCCGTCGAGATGCACCACGAGCAGCTGCAGGAGGGTGTCCC
CGGCGACAACGTTGGCTTCAACGTGAAGAACGTCTCCGTCAAGGAAATCCGCCGAGGCAAC
GTCGCTGGTGACTCCAAGAACGATCCTCCTGCTGCCGCCGCTTCCTTCACCGCTCAGGTCA
TCGTCCTCAACCACCCCGGTCAGGTCGGTGCTGGTTACGCTCCCGTCCTGGATTGCCACAC
TGCCCACATTGCCTGCAAGTTCTCTGAGATCCTTGAGAAGATCGACCGCCGAACGGGCAAGT
CTGTTGAGAACAACCCCAAGTTCATCAAGTCTGGTGACGC
>Distoseptispora_fusiformis_GZCC_20_0512
ACCT--------GT---C--G------TACTGCTTTGGCGGG-CGGCCCC-TGGGAGGGGCCACTGCCCGT
TGGGTCGGGCGCCCGCCGAAGCA--TCA-C-CACAACTCTGGCACCC--CCGGGCGTCTTCAG
AGCA---------TAGC---ATAAGCTAT-AAAAACTTTCAGCAACGGATCTCTTGGTTCTGGCATCGAT
GAAGAACGCAGCGAAATGCGATAAGTAATGTGAATTGCAGAATTTAGTGAATCATCGAATCTTT
GAACGCACATTGCGCCCGCCAGCATTCTGGCGGGCATGCCTGTCCGAGCGTCATTTCAACC
CCTCGGGCGCT-GAAG-------CG-CCCGGCGTTGGGGCACTGCGCCACCTGTAGGTACACCTG
TACCT---CGGGCGCTGCAGGCCCCGAAAGACAGTGGCGGGACCGTCGGGGCT-CCGGGCG
CAGTAGTTT-ACCA-CCTCGCCCAGGTGTCTC-CAGCGG--CC-CCAGCCGTCAAAAC-ACCCCC
-A------ACAGACGA-------ACCAGCCCGCCGAGTCCCC-TGGAAC------GGGGCGCC-ACAGAGG
G-TGAGAGCCCCGTATGGCAGGACGCCGAGCC-TCTGTAAAGCTCCTTCGACGAGTCGAGTA
GTTTGGGAATGCTGCTCTAAATGGGAGGTAAATTTCTTCTAAAG-CTAAATACCGGCCAGAGAC
CGATAGCGCACAAGTAGAGTGATCGAAAGATGAAAAGCACTTTGAAAAGAGGGTTAAAAAGC
ACGTGAAATTGCTGAAAGGGAAGCGCTTGTGACCAGACTTGCGCCCGGCGGATCAGCTGGC
GTTCTCGCCAGTGCACTCCGCCGGGCTCAGGCCAGCATCGGTTTCGCCAGGGGGACAAAA
GCGCCGGGAACGTGGCTCCCCCCGGGGAGTGTTACAGCCCGGCG-CAGAATGCCCTTAGG
CGGGACCGAGGAACGCGCACCG-CAAGGATGCTGGCGTAATGGTCATCAGCGACCCGTCTT
TCTCGCTTATGTGTTATGTCAGTGTTGGGACGCCATCTGAACCTATCATAGACTTCATGGTGG
CAAGAAACATGGAACTTCTTGAGGAATACGAGCCGCTACGGTACCCCAACGCCACCAAGATT

TTTGTCAATGGCACATGGGTTGGTGTTCACCAAGACCCGCGTCATTTGGTGAACTCGGTGAG
AGACCTGCGTCAAAAGGGTGTTATAAACTTTGAAGTTTCTCTTATCCGGGATATCCGTGATCG
AGAGTTTAAAATTTTCTCCGACGCTGGCCGTGTCATGAGGCCTCTCTTCGCCGTCGAGCAAA
TCGGC---GACCCGACCAAG---AATCGGGAGAAGGGTTCTTTGTGGCTCACCAAGCAACACAT
CGAACGACTTAGAGAAGATGAGATGCTTGACCGAGACCATCCTAAGTATTTTGGTTGGGACG
GATTGACCAAAGCTGGTGTTATCGAGTATGTGGACGCCGAAGAAGAAGAGACTGCCATGATT
TGCATGTCACCAGAAGACCTGGAACAATATAGGCTTCAGAAGGCGGGCGTCGAGGTCGAGG
AAGACGATGACGAAGCCG---ATCGCAACCGTCGTCTGAAAACCAAAATGAACCCGACGACAC
ACATGTACACGCATTGCGAGATTCATCCGAGCATGCTTCTGGGGATTTGCGCCAGCATTATCC
CACTCGTGAGCACGCTCTGCTTGCCTACACCCTGGGTGTGAAGCAGCTCATCGTCGCGATC
AACAAGATGGACACTACCAAGTGGTCCGAGGATCGTTTCAACGAAATCATCAAGGAGACGTC
CAACTTCATCAAGAAGGTCGGCTACAACCCCAAGCAGGTTGCCTTCGTCCCCATCTCGGGCT
TCAACGGCGACAACATGCTTGCTGCCTCCAGCAACTGCCCCTGGTACAAGGGCTGGGAGAA
GGAGG---TCAAGGGTGGCAAGGTCACTGGCAAGACTCTCCTCGAGGCCATTGATGCCATCG
AGCCCCCTAAGCGTCCCACCGACAAGCCCCTCCGCCTTCCCCTTCAGGATGTCTACAAGATT
GGTGGTATCGGAACTGTCCCTGTCGGCCGTATCGAGACGGGTGTTCTCAAGCCCGGTATGG
TCGTCACCTTTGCTCCCTCCAACGTCACCACTGAAGTCAAGTCGGTCGAGATGCACCACGA
GCAGCTTAGTGAGGGTGTTCCCGGCGACAACGTTGGCTTCAACGTGAAGAACGTCTCCGTC
AAGGAAATCCGTCGTGGCAACGTCGCTGGTGACTCCAAGAACGACCCCCCTGCTGGCTGTG
CTTCCTTCACTGCCCAGGTCATCGTTCTCAACCACCCCGGTCAGGTCGGCGCTGGCTACGC
CCCTGTCCTGGACTGCCACACCGCCCATATCGCCTGCAAGTTCTCTGAGATCCTAGAGAAGA
TCGATCGCCGAACGGGCAAGTCGGTTGAGAACAACCCTAAGTTCATCAAGTCGGGCGACGC
>Distoseptispora_guizhouensis_GZCC21_0666
ACCT--------TT-ACGTT-------CGTTGCTTTGGCGGG-CGGCTCC-AGGGCGGAGCCACTGCCCA
TTTGGTTGGGCGCCCGCCGAAGGA---CAAT-CAAAACTCGCT-AAATCTGT-ACGGATCTCAGA
GTAAAAA-----TAT-----CAAGCTAT-TAAAACTTTCAGCAACGGATCTCTTGGTTCTGGCATCGATG
AAGAACGCAGCGAAATGCGATAAGTAATGTGAATTGCAGAATTCAGTGAATCATCGAATCTTT
GAACGCACATTGCGCCCGCCAGCACTCTGGCGGGCATGCCTGTTCGAGCGTCATTTCAA-CC
CTCGGGCCC-TCGTT-------TGGCCCGGTGTTGGGGTGCTACGGGTCCG--TC-----------------CG
AC-CTGTAGGCCCTCAAAAACAGTGGCGGGCTCGCCAGGACT-CCGAGCGTAGTAGTTT-TCC
T--CTCGCTCAGGCGGCCT-GGCGTG--CT-TCGGCCGTTAAA----ACCCCAA------C-----------------
GCCTTCCGAGTCCCC-TGGAAC------GGGGCGCC-AGAGAGGG-TGAGAGCCCCGTATGGTTG
GACGCCGAACC-TTTGTAAAGCTCCTTCGACGAGTCGAGTAGTTTGGGAATGCTGCTCAAAAT
GGGAGGTAAATTTCTTCTAAAG-CTAAATACCGGCCAGAGACCGATAGCGCACAAGTAGAGTG
ATCGAAAGATGAAAAGCACTTTGAAAAGAGGGTCAAATAGCACGTGAAATTGCTGAAAGGGA
AGCGCTTGTGACCAGACTTGCGCCCGGTTGATCATCCAGCGTTCTCGCTGGTGCACTCTGC
CGGGTTCAGGCCAGCATCGGCTCTCCCAGGGGGATAAAGGTCGCGGGAACGTAGCTCTCTC
CGGGGAGTGTTATAGCCCGTGG-CGGAATGCCCTTAGGGGGGGCCGAGGCTCGCGTTC-G-C
AAGGATGCTGGCGTAATGGTCACCAGCGACCCGTCTTTATCGCTCATGTGTTATGTGAGCGT
CGGAACGCCCGCGGAGCCAATCGTCGAGTTCATGATTGCTAGGAACATGGAAGTACTCGAG
GAATATGAGCCCTTGAGGTACCCTAATGCGACCAAAATCTTCGTCAATGGCACATGGGTTGGT
GTTCACCAAGACCCGAAGCATCTTGTGAGCTTGGTTCAGGACTTACGCCGCAGGGGCGTCA
TCAACTTCGAGGTCTCCCTCATTCGGGATATCCGTGATCGAGAGTTCAAGATTTTCTCGGATG
CTGGTCGTGTTATGCGTCCTCTCTTCGCTGTGGAGCAGTCAGAC---AACTCGACAAAG---GGC

CTTGAAAAGGGCTCTTTATTGCTCACAAAGGAGCATATCCAGAGGTTGAGAGCAGATGAGGA
GTTATCCAAGGAAGACTCAGACTATTTCGGCTGGGATGGCCTTCTCAGGGCGGGCGCCATC
GAATATCTTGACGCCGAAGAGGAAGAGACAGCCATGATTTGCATGACTCCAGAAGACCTAGA
GGCTTATCGGCTGCAAAAAGCTGGATTAGACGTGCCGGAAGACGATGACGAGGCCG---ACCC
CAATAGGCGTCTGAAGACAAAATTGAACCCCACCACTCACATGTATACACACTGTGAGATCCA
TCCGAGCATGCTCTTGGGGATCTGCGCGAGCATTATCCCACTCGTGAGCACGCTCTGCTTGC
CTACACCCTGGGTGTGAAGCAGCTCATCGTCGCCATCAACAAGATGGACACCACCAAGTGGT
CTGAGAGCCGTTACAACGAAATTATCAAGGAGACGTCCAACTTCATCAAGAAGGTCGGCTAC
AACCCCAAGCAGGTCGCCTTCGTCCCCATCTCCGGCTTCAACGGCGACAACATGCTTGCCC
CCTCCTCCAACTGCCCCTGGTACAAGGGCTGGGAGAAGGAGATC---AAGGGCGGCAAGGCC
TCTGGCAAGACCCTTCTCGAGGCCATCGATGCCATTGAGCCTCCTAAGCGTCCTACGGACAA
GCCTCTGCGTCTCCCCCTCCAGGATGTCTACAAGATCGGTGGTATTGGAACTGTACCCGTCG
GCCGTATCGAGACTGGTATCCTCAAGCCCGGTATGGTCGTCACGTTTGCTCCCTCCAACGTC
ACCACTGAAGTCAAGTCCGTCGAGATGCACCACGAGCAGCTGCAGGAGGGTGTCCCCGGC
GACAACGTTGGCTTCAACGTGAAGAACGTCTCCGTCAAGGAAATCCGCCGAGGCAACGTTG
CTGGTGACTCCAAGAACGATCCTCCGGCTGCTGCCGCTTCCTTCACCGCCCAGGTCATCGT
CCTCAACCACCCCGGTCAGGTCGGCGCTGGCTACGCTCCCGTCTTGGATTGCCACACTGCC
CACATCGCTTGCAAGTTCTCCGAGATCCTTGAGAAGATCGACCGCCGAACGGGCAAGTCTGT
CGAGAACAACCCCAAGTTCATCAAGTCTGGTGACGC
>Distoseptispora_guttulata_MFLU_17_0852
ACCT--------GTT--TTTT------CGTTGCTTCGGCGGG-CGGCTCC-AGGGCGGAGCCACTGCCCG
TTGGTTCGGGCGCCCGCCGGAGGA---CAAC-CAAAACTAGCC-TAATCTT-GGGTAACTTCAGA
GTATTA----AAAAA----AAA-GCTAT-CAAAACTTTCAGCAACGGATCTCTTGGTTCTGGCATCGAT
GAAGAACGCAGCGAAATGCGATAAGTAATGTGAATTGCAGAATTCAGTGAATCATCGAATCTT
TGAACGCACATTGCGCCCGCCAGCACTCTGGCGGGCATGCCTGTTCGAGCGTCATTTCAA-C
CCTCGGGCCCCTCTTTT---GCGTGGCCCGGTGTTGGGGCGCTACCGCGTCCCTCCTC---------
---GGGGGGACCCGGTAGGCCCTTAAAACCAGTGGCGGGCTCGCCACGGCCTCCGAGCGCA
GTAGTTC-TCTC--CTCGCTCAGGCGGCCT-GGCGCG--CC-TCGGCCGTTAAA---CATACG-C-----
-TGTGTCTC----ACAG-AAGCCTTCCGAGTCCCC-TGGAAC------GGGGCGCC-AGAGAGGG-TG
AGAGCCCCGTACGGTCGGACGCCGAGCC-TCTGTAAAGCTCCTTCGACGAGTCGAGTAGTTT
GGGAATGCTGCTCAAAATGGGAGGTAAATTTCTTCTAAAG-CTAAATACCGGCCAGAGACCGA
TAGCGCACAAGTAGAGTGATCGAAAGATGAAAAGCACTTTGAAAAGAGGGTTAAATAGCACGT
GAAATTGCTGAAAGGGAAGCGCTTGTGACCAGACTTGCGCCCGGTTGATCATCCAGCGTTCT
CGCTGGTGCACTCTGCCGGGCTCAGGCCAGCATCGGCTCCCCCAGGGGGATAAAGGTCGC
GGGAACGTAGCTCTCTCCGGGGAGTGTTATAGCCCGCGG-CGGAATACCCTTAGGGGGGGT
CGAGGCCCGCGTTC-G-CAAGGATGCTGGCGTAATGGTCACCAGCGACCCGTCTT?????????
???
???
???
???
???
???
???
???

???ACTCGTGAGCA
CGCTCTACTTGCCTACACCCTGGGTGTGAAGCAGCTTATCGTGGCCATCAACAAGATGGACA
CCACCAAGTGGTCTGAGGACCGTTACAACGAAATCATCAAGGAGACTTCCAACTTCATCAAG
AAGGTCGGCTACAACCCCAAGCAGGTTGCTTTCGTTCCCATCTCTGGCTTCAACGGCGACAA
CATGCTTACCCCCTCCTCCAACTGCCCCTGGTACAAGGGCTGGGAGAAGGAGGTC---AAGG
GTGGCAAGGTCACTGGCAAGACGCTTCTCGAAGCCATCGATGCCATTGAGCCTCCCAAGCG
CCCCACAGACAAGCCGCTGCGTCTTCCCCTCCAGGATGTCTACAAGATCGGTGGTATCGGAA
CCGTACCTGTCGGCCGTATCGAGACTGGTGTCCTCAAGCCCGGTATGGTCGTCACTTTCGCT
CCCTCCAACGTCACCACTGAAGTCAAGTCTGTCGAGATGCACCACGAACAGCTTCAAGAGG
GCGTTCCTGGCGACAACGTCGGCTTCAACGTGAAGAACGTCTCTGTCAAGGAAATCCGCCG
AGGCAACGTCGCTGGTGACTCCAAGAACGACCCTCCTGCTGGCGCTGCTTCCTTCACCGCC
CAGGTCATTGTCCTGAACCACCCCGGTCAGGTCGGCGCTGGCTACGCTCCCGTCTTGGATT
GCCACACTGCCCACATCGCCTGCAAGTTCTCCGAGATCCTCGAGAAGATCGATCGCCGAAC
GGGCAAGTCTGTCGAGAACAACCCCAAGTTCATCAAGTCTGGTGACGC
>Distoseptispora_hyalina_MFLUCC_17_2128
ACCT--------ATCGTCAA-------TGTTGCTTCGGCGGG-CGGCCCC-TGGGAGGGGCCACGGCCC
ATTGGGTTGGGTGCCCGCCGGAGGA---CGAC-CCAAAACTCGTTTTTTTACAAGGTATCTTCT
GAGTAAA------ACACA--TAAA--TTAT-AAAAACTTTCAACAACGGATCTCTTGGTTCTGGCATCGA
TGAAGAACGCAGCGAAATGCGATAAGTAATGTGAATTGCAGAATTCAGTGAATCATCGAATCT
TTGAACGCACATTGCGCCCGCCAGTATTCTGGCGGGCATGCCTGTTCGAGCGTCATTTCA-A
CCCTCAGGCAC-CT-------GT--GGCCTGGTGTTGGGGCACTGCCGTCCCGCCCC--------------GT
ACGGGGCGCGCAGGCCCTCAAAAACAGTGGCGGGCTCGCTAGGAC-TCCGGGCGCAGTAG
T-ATCAT-ATCTCGTCTAGGTGGTCT-GGCGCG--GT-CTTGCCGTCAAAAC-CCCCA----------AAC
TTTT-------ACAAGCCTTCTAAGTCCCC-TAGAAC------GGGGCGCC-ATAGAGGG-TGAGAGCCC
CGTACAGTTGGACACCGAACC-TCTGTAAAGCTCCTTCGACGAGTCGAGTAGTTTGGGAATG
CTGCTCAAAATGGGAGGTAAATTTCTTCTAAAG-CTAAATACCGGCCAGAGACCGATAGCGCA
CAAGTAGAGTGATCGAAAGATGAAAAGCACTTTGAAAAGAGGGTTAAATAGCACGTGAAATTG
TTGAAAGGGAAGCGCTTGTGACCAGACTTGCGCCCGGTGAATCATCCAGCGTTCTCGCTGG
TGCACTTTGCCGGGCTCAGGCCAGCATCGGTTCTTCCAGGGGGATAAAAGCCTCGGGAACG
TAGCTCCCTTCGGGGAGTGTTATAGCCCGTGGGTACAATACCTCTGGG-GGGACCGAGGACC
GCGCTCTG-CAAGGATGCTGGCGTAATGGTCATCAGCGACCCGTCTTTGTCGCTCATGTGTTA
TGTCAGCGTTGGAACCCCGGCAGAACCGATCATCGAGTTCATGATTGCGAGGAACATGGAAG
TCCTCGAGGAATACGAACCCTTGCGCTACCCCAACGCTACCAAGATATTCGTCAATGGGACG
TGGGTAGGTGTTCATCAGGACCCTAAACATCTGGTTGGGCTGGTACAGGAGTTGCGACAGAG
GGGCGTCATCAACTTTGAGGTCTCTCTTGTCCGTGATATTCGGGACAGGGAGTTCAAGATTTT
CTCTGATGCTGGCCGAGTGATGCGGCCCCTTTTCGCCGTGGAGCAAGAGGAC---AACTCTAA
CTCA---GGGCTTCCAAAGGGGGCTCTCAGACTCACTAAAGAGCACATTCAGAGACTCAGAAC
AGACGAAGAGTTATCCAAAGATGATGAGGGATATTTCGGATGGGATGGTATCGTTGAGGCTGG
TGCAGTTGAATATCTCGACGCGGAAGAAGAGGAAACGGCTATGATTTGCATGACGCCGGAAG
ATCTGGAGGCTTATCGACTCCAGAAAGCCGGGTTGGGTATGGCCGAAGACGACGACGAAGC
GG---ATCCGAACAGACGGCTGAAAACGAAATTAAATCCCACCACTCACATGTACACGCACTGC
GAAATTCATCCAAGTATGCTTCTGGGGATCTGCGCAAGCATCATACCACTCGTGAGCACGCTC
TGCTTGCCTACACCCTGGGTGTCAAGCAGATCATCTGCGCCATCAACAAGATGGACACCACC
AAGTGGTCAGAGGACCGTTTCAACGAAATCATCAAGGAGACGTCCAACTTCATCAAGAAGGT

CGGCTACAACCCCAAGCAGGTCGCCTTCGTCCCCATCTCCGGCTTCCACGGTGACAACATG
TTGTCTCCCTCCACCAACTGCCCCTGGTACAAGGGCTGGGAGAAGGAAGGCGCCAAGGGT
GCCAAGTCATCTGGCAAAACCCTGCTTGAGGCCATCGACGCCATCGAGCCCCCGAAGCGTC
CCACCGACAAGCCCCTGCGTCTCCCGCTCCAGGATGTGTACAAGATTGGTGGTATCGGAACA
GTTCCTGTCGGCCGTATCGAGACTGGTGTCCTCAAGCCCGGTATGGTCGTCACTTTCGCTCC
CTCCAACGTCACCACTGAAGTCAAGTCCGTCGAGATGCACCACGAGCAGCTGTCTGAGGGT
GTCCCCGGTGACAACGTCGGCTTCAACGTGAAGAACGTCTCTGTCAAGGAAATCCGCCGTG
GCAACGTCGCTGGTGACTCCAAGAACGACCCCCCTGCTGGCTGCGCCAACTTCACTGCCCA
GGTCATTGTCCTCAACCACCCTGGTCAGGTTGGTCCCGGCTACGCCCCTGTCTTGGACTGC
CACACTGCCCACATCGCTTGCAAGTTCTCCGAGATCACCGAGAAGATCGACCGCCGTACCG
GCAAGTCGGTTGAGAACAACCCCAAGTTCATCAAGTCCGGTGACGC
>Distoseptispora_hydei_MFLUCC_20_0481
ACCT--------GTC--GTT-------CGTTGCTCCGGCGGG-CGGCTCC-AGGGCGGAGCCACTGTCCG
TTAGGTCGGACGCCCGCCGGGGGA---CAAC-CAAAACCCTTC-TGATCTC-GGGTATCGTCAG
AGTATAT----AAAA------CAAGCTAT-CAAAACTTTCAGCAACGGATCTCTTGGTTCTGGCATCGAT
GAAGAACGCAGCGAAATGCGATAAGTAATGTGAATTGCAGAATTCAGTGAATCATCGAATCTT
TGAACGCACATTGCGCCCGCCAGTACTCTGGCGGGCATGCCTGTTCGAGCGTCATTTCAA-C
CCTCGGGCC-CCTGT------TT-GGCCCGGTGTTGGGGTGCTAC--GTCCTGA--------------------CG
GACC-GTAGGCCCTCAAAACCAGTGGCGGGCT--
--GCCTTCCGAGTCCCC-TGGAAC------GGGGCGCC-AGAGA
GGG-TGAGAGCCCCGTATGGTCGGACGCCGAACC-TTTGTAAAGCTCCTTCGACGAGTCGAG
TAGTTTGGGAATGCTGCTCAAAATGGGAGGTAAATTTCTTCTAAAG-CTAAATACCGGCCAGAG
ACCGATAGCGCACAAGTAGAGTGATCGAAAGATGAAAAGCACTTTGAAAAGAGGGTTAAATAG
CACGTGAAATTGCTGAAAGGGAAGCGCTTGTGACCAGACTTGCGCCCGGTTGATCATCCAG
CGTTCTCGCTGGTGCACTCTGCCGGGCTCAGGCCAGCATCGGCTCTCCCAGGGGGATAAAG
GTCGCGGGAACGTAGCTCTCTCCGGGGAGTGTTATAGCCCGCGG-CGGAATACCCTTAGGG
GGGGTCGAGGCCCGCGTTC-G-CAAGGATGCTGGCGTAATGGTCACCAGCGACCCGTCTTTA
TCACTCATGTGTTATGTCAGTGTCGGAACACCTGCGGAGCCAATCGTCGATTTCATGATTGCT
AGAAACATGGAGGTCCTCGAAGAATACGACCCTCTAAGGTTGCCAAATGCGACCAAAGTCTT
CGTCAACGGCTCGTGGGTGGGTTGCCACCAAGATCCAAGCCATCTCGTGCGCTTGGTGCAG
GAGCTGCGCCGCAAGGGCGTCATCAATTTCGAGGTCTCGCTAATTCGAGACATACGCGATCG
AGAATTCAAGATTTTCTCGGATGCCGGACGTGTCATGCGACCGCTCTTCGCTGTAGAGCAGT
CAGAC---AACTCAGACAAG---GGCATTGAGAAAGGTGCCTTGTTGCTTACCAAGGATCACATA
CAGAGATTGAGAGCAGACGAGGAGTTGCCAAAGGACGATGCAGAATATTTCGGTTGGGACG
GCCTACTCAATGCCGGTGCAATAGAGTATCTTGACGCAGAAGAGGAAGAGACGGCCATGATT
TGCATGACCCCGGAAGATTTAGAGGCCTATCGACTTCAAAAGGCTGGATTCGACGTGCCGGA
TGACGACGACGAAGCCG---ATCCCAATAGGCGGCTCAAGACCAAGCTGAATCCCACTACTCA
CATGTACACCCATTGTGAGATTCACCCCAGTATGCTCTTGGGGATCTGCGCAAGCATCATTCC
??
??
??
??
??
??

???
???
???
???
???????????????????????????
>Distoseptispora_lancangjiangensis_DLUCC_1864
ACCT--------ATCGTCAAA------TGTTGCTTCGGCGGG-CGGCCCC-TGGGAGGGGCCAGGGCC
CGTTGGGTCGGGTGCCCGCCGGAGGA---CAGC-CGAGAACTCTTCTT---ATCAGGTACTGTCC
GAGTAAA------CTTTAA-AAAA--CTATTAAAAACTTTCAACAACGGATCTCTTGGTTCTGGCATCG
ATGAAGAACGCAGCGAAATGCGATAAGTAATGTGAATTGCAGAATTCAGTGAATCATCGAATCT
TTGAACGCACATTGCGCCCGCCAGCACTCTGGCGGGCATGCCTGTTCGAGCGTCATTTCA-A
CCCTCAGGCCC-TA-------GTTTGGCCTGGTGTTGGGGCACTGCCGTCCCGCCC--------------GT
ACGGGGCGAGCAGGCCCTGAAAACCAGTGGCGGGCTCGCTAGCAC-TCCGGGCGTAGTAG
A-ATCAT-ATCTCGTCTAGGTGGTCT-GGCGCG--GT-CTTGCCGTCAAAAC-CCCCC---------AATT
TTAT-------ACAAGCCTTCTAAGTCCCC-TAGAAC------GGGGCGCC-ACAGAGGG-TGAGAGCCC
CGTACAGTTGGACACCGAACC-TTTGTAAAGCTCCTTCGACGAGTCGAGTAGTTTGGGAATGC
TGCTCAAAATGGGAGGTATATTTCTTCTAAAG-CTAAATACCGGCCAGAGACCGATAGCGCACA
AGTAGAGTGATCGAAAGATGAAAAGCACTTTGAAAAGAGGGTTAAATAGCACGTGAAATTGTT
GAAAGGGAAGCGCTTGTGACCAGACTTGCGCCCGGTGAATCATCCAGCGTTCTCGCTGGTG
CACTTTGCCGGGCTCAGGCCAGCATCGGTTCTCCCAGGGGGACAAAGGTCTCGGGAACGTA
GCTCTCTTCGGGGAGTGTTATAGCCCGTGG-CATAATACC-TTTGGGGGGACCGAGGACCGC
GCTCTG-CAAGGATGCTGGCGTAATGGTCATCAGCGACCCGTCTT?????????????????????
???
???
???
???
???
???
???
???
???
???
???
???
???
???
???
???
???
???
???
>Distoseptispora_leonensis_HKUCC_10822
???
???

??
??
??
??
??
????????GCCTTCCGAGTCCCC-TGGAAC------GGGACGCC-ACAGAGGG-TGAGAGCCCCGTA
TGGTTGGACGCCGAGCC-TCTGTAAAGCTCCTTCGACGAGTCGAGTAGTTTGGGAATGCTGC
TCTAAATGGGAGGTAAATTTCTTCTAAAG-CTAAATACCGGCCAGAGACCGATAGCGCACAAGT
AGAGTGATCGAAAGATGAAAAGCACTTTGAAAAGAGGGTTAAAAAGCACGTGAAATTGCTGA
AAGGGAAGCGCTCGTGACCAGACTTGCGCCCGGCGGATCANCCNGCGTTCTCGCCGGTGC
ACTCCGCCGGGTCCAGGCCAGCATCGGTTTCGCCAGGGGGACAAAAGCCACTGGAACGTA
GCTCTCTACGGGGAGTGTTATAGCCTGTGG-CACAATGCCCTTAGGCGGGACCGAGGCCCG
CGCTCTG-CAAGGATGCTGGCGTAATGGTCACCAGCGACCCGTCTTTCTCGCTCATGTGTTAT
GTCAGTGTTGGGACGCCTTCTGAACCTATCGTGGATTTCATGGTGGCTAGAAATATGGAACTT
CTCGAGGAGTACGAGCCGCTGCGGTACCCCAACGCCACCAAGATTTTTGTCAATGGCACATG
GGTTGGAGTACACCAGGACCCACGGCATTTGGTGAATTCGGTGAAGGAGCTGCGGCAAAAA
GGTGTCATCAACTTCGAGGTGTCTCTTATTCGGGATATCCGTGATCGAGAGTTCAAGATTTTC
TCGGACGCTGGCCGTGTCATGAGGCCTCTTTTTGCCGTTGAGCAAATCGGT---GATCCGAGC
AAG---AACCGAGAGAAGGGCTCGTTGTGGCTCACCAAGCAACACATCGAACGACTCAGAGA
GGATGAGATGCTGGACCGAGATCACCCTAAATACTTCGGTTGGGACGGTCTGACCAAAGCCG
GTGTTATCGAGTACGTGGATGCCGAGGAAGAAGAGACTGCCATGATTTGCATGTCACCGGAA
GACTTGGAACAGTACAGGCTTCAAAAGGCGGGTGTCGAGGTTGAAGAAGATGATGACGAGA
CCG---ATCCCAACCGTCGCATCAAGACTAAGATGAATCCAACGATGCACATGTACACCCATTG
TGAGATTCATCCGAGCATGC------------------------------???????????????????????????????????????
??
??
??
??
??
??
??
??
??
??
>Distoseptispora_licualae_MFLUCC_14_1163A
ACCT--------AC--TTGCC------TGTTGCTCCGGCGGG-CGGACCC-TGGGAGGGACCACTGCCCG
TTAGGTCGGGTGTCCGCCGGAGGA---CGTC--TAAAACTCTTTTGT---TCAGGTACCGTCCGAG
CCAA------A-ACAA-GAA---CTAT-AAAAACTTTCAACAACGGATCTCTTGGTTCTGGCATCGATGA
AGAACGCAGCGAAATGCGATAAGTAATGCGAATTGCAGAATTCAGTGAGTCATCGAATCTTTG
AACGCACATTGCGCCCGCCAGCACTCTGGCGGGCATGCCTGTTCGAGCGTCATTTCA-ACCC
TCAGGCCC-CC-------GT--GGCCTGGTGTTGGGGCACTGCCGCGCCGCCC--------------GTTCG
GCGCGCGCAGGCCCTGAAAACCAGTGGCGGGCTCGCCAGCTC-TCCGGGCGCAGTAG----T
TT-GTCTCGCCTCGGCGGTCT-GGCGCG--GT-CTTGCCGTGAAACC-CCACTT--------GAACCT--
-------AAAAGCCTTCCAAGTCCCC-TAGAAC------GGGGCGCC-ACAGAGGG-TGAGAGCCCCGT

ACGGTTGGACGCCGAGCC-TCTGTAAAGCTCCTTCGACGAGTCGGGTAGTTTGGGAATGCTG
CTCAAAATGGGAGGTAAATTTCTCCTAAAG-CTAAATACCGGCCAGAGACCGATAGCGCACAA
GTAGAGTGATCGAAAGATGAAAAGCACTTTGAAAAGAGGGTTAAACAGCACGTGAAATTGTTG
AAAGGGAAGCGCTTGTGACCAGACTTGCGCCCGGCGGATCATCCGGCGTTCTCGCCGGTG
CACTCCGCCGGGCTCAGGCCAGCATCGGTTCTGCCCGGGGGACAAAGGCCGCGGGAACGT
AGCTCTCTTCGGGGAGTGTTATAGCCCGTGG-CGTAATACCCTGGGG-GGGACCGAGGACCG
CGCTCTG-CAAGGATGCTGGCGTAATGGTCATCAGCGACCCGTCTT????????????????????
??
??
??
??
??
??
??
??
??ACTCGTGAGCACGCTCTGCT
TGCCTACACCCTGGGTGTCAAGCAGATCATCTGCGCCATCAACAAGATGGACACCACCAAGT
GGTCAGAGGACCGTTTCAACGAAATTATCAAGGAGACGTCCAACTTCATCAAGAAAGTCGGC
TACAACCCCAAGCAGGTCGCCTTCGTCCCTATCTCAGGCTTCCACGGCGACAACATGTTGGC
TCCCTCCACCAACTGCCCCTGGTACAAGGGCTGGGAGAAGGAGGGTGCCAAGGGTGCTAA
GTCCTCTGGCAAGACCCTGCTTGAGGCCATCGACGCCATTGAGCCCCCCAAGCGTCCCACC
GACAAGCCTCTGCGTCTCCCGCTCCAGGATGTCTACAAGATTGGCGGTATCGGAACTGTCCC
TGTCGGCCGTATCGAGACTGGTGTCCTAAAGCCCGGTATGGTCGTTACCTTTGCTCCCTCCA
ACGTCACCACTGAAGTGAAGTCCGTCGAGATGCACCACGAGCAGCTTCAGGAGGGTGTCCC
CGGCGACAACGTCGGCTTCAACGTGAAGAACGTCTCCGTCAAGGAAATCCGCCGTGGCAAC
GTCGCTGGTGACTCCAAGAATGACCCCCCGGCTGGCTGCGCCAACTTCACCGCCCAGGTCA
TTGTTCTCAACCACCCCGGTCAAGTTGGTCCCGGCTACGCTCCCGTTCTGGACTGCCACACT
GCCCACATCGCCTGCAAGTTCTCTGAGATCACCGAGAAGATCGACCGCCGTACGGGCAAGT
CGGTTGAGAACAACCCCAAGTTCATCAAGTCCGGTGACGC
>Distoseptispora_licualae_MFLUCC_14_1163B
ACCT--------AC--TTGCC------TGTTGCTCCGGCGGG-CGGACCC-TGGGAGGGACCACTGCCCG
TTAGGTCGGGTGTCCGCCGGAGGA---CGTC--TAAAACTCTTTTGT---TCAGGTACCGTCCGAG
CCAA------A-ACAA-GAA---CTAT-AAAAACTTTCAACAACGGATCTCTTGGTTCTGGCATCGATGA
AGAACGCAGCGAAATGCGATAAGTAATGCGAATTGCAGAATTCAGTGAGTCATCGAATCTTTG
AACGCACATTGCGCCCGCCAGCACTCTGGCGGGCATGCCTGTTCGAGCGTCATTTCA-ACCC
TCAGGCCC-CC-------GT--GGCCTGGTGTTGGGGCACTGCCGCGCCGCCC---------------GTTCG
GCGCGCGCAGGCCCTGAAAACCAGTGGCGGGCTCGCCAGCTC-TCCGGGCGCAGTAG----T
TT-GTCTCGCCTCGGCGGTCT-GGCGCG--GT-CTTGCCGTGAAACC-CCACTT--------GAACCT--
-------AAAAGCCTTCCAAGTCCCC-TAGAAC------GGGGCGCC-ACAGAGGG-TGAGAGCCCCGT
ACGGTTGGACGCCGAGCC-TCTGTAAAGCTCCTTCGACGAGTCGGGTAGTTTGGGAATGCTG
CTCAAAATGGGAGGTAAATTTCTCCTAAAG-CTAAATACCGGCCAGAGACCGATAGCGCACAA
GTAGAGTGATCGAAAGATGAAAAGCACTTTGAAAAGAGGGTTAAACAGCACGTGAAATTGTTG
AAAGGGAAGCGCTTGTGACCAGACTTGCGCCCGGCGGATCATCCGGCGTTCTCGCCGGTG
CACTCCGCCGGGCTCAGGCCAGCATCGGTTCTGCCCGGGGGACAAAGGCCGCGGGAACGT

AGCTCTCTTCGGGGAGTGTTATAGCCCGTGG-CGTAATACCCTGGGG-GGGACCGAGGACCG
CGCTCTG-CAAGGATGCTGGCGTAATGGTCATCAGCGACCCGTCTT????????????????????
???
???
???
???
???
???
???
???
???ACTCGTGAGCACGCTCTGCT
TGCCTACACCCTGGGTGTCAAGCAGATCATCTGCGCCATCAACAAGATGGACACCACCAAGT
GGTCAGAGGACCGTTTCAACGAAATTATCAAGGAGACGTCCAACTTCATCAAGAAAGTCGGC
TACAACCCCAAGCAGGTCGCCTTCGTCCCTATCTCAGGCTTCCACGGCGACAACATGTTGGC
TCCCTCCACCAACTGCCCCTGGTACAAGGGCTGGGAGAAGGAGGGTGCCAAGGGTGCTAA
GTCCTCTGGCAAGACCCTGCTTGAGGCCATCGACGCCATTGAGCCCCCCAAGCGTCCCACC
GACAAGCCTCTGCGTCTCCCGCTCCAGGATGTCTACAAGATTGGCGGTATCGGAACTGTCCC
TGTCGGCCGTATCGAGACTGGTGTCCTAAAGCCCGGTATGGTCGTTACCTTTGCTCCCTCCA
ACGTCACCACTGAAGTGAAGTCCGTCGAGATGCACCACGAGCAGCTTCAGGAGGGTGTCCC
CGGCGACAACGTCGGCTTCAACGTGAAGAACGTCTCCGTCAAGGAAATCCGCCGTGGCAAC
GTCGCTGGTGACTCCAAGAATGACCCCCCGGCTGGCTGCGCCAACTTCACCGCCCAGGTCA
TTGTTCTCAACCACCCCGGTCAAGTTGGTCCCGGCTACGCTCCCGTTCTGGACTGCCACACT
GCCCACATCGCCTGCAAGTTCTCTGAGATCACCGAGAAGATCGACCGCCGTACGGGCAAGT
CGGTTGAGAACAACCCCAAGTTCATCAAGTCCGGTGACGC
>Distoseptispora_licualae_MFLUCC_14_1163C
ACCT--------AC--TTGCC------TGTTGCTCCGGCGGG-CGGACCC-TGGGAGGGACCACTGCCCG
TTAGGTCGGGTGTCCGCCGGAGGA---CGTC--TAAAACTCTTTTGT---TCAGGTACCGTCCGAG
CCAA------A-ACAA-GAA---CTAT-AAAAACTTTCAACAACGGATCTCTTGGTTCTGGCATCGATGA
AGAACGCAGCGAAATGCGATAAGTAATGCGAATTGCAGAATTCAGTGAGTCATCGAATCTTTG
AACGCACATTGCGCCCGCCAGCACTCTGGCGGGCATGCCTGTTCGAGCGTCATTTCA-ACCC
TCAGGCCC-CC-------GT--GGCCTGGTGTTGGGGCACTGCCGCGCCGCCC---------------GTTCG
GCGCGCGCAGGCCCTGAAAACCAGTGGCGGGCTCGCCAGCTC-TCCGGGCGCAGTAG----T
TT-GTCTCGCCTCGGCGGTCT-GGCGCG--GT-CTTGCCGTGAAACC-CCACTT--------GAACCT--
-------AAAAGCCTTCCAAGTCCCC-TAGAAC------GGGGCGCC-ACAGAGGG-TGAGAGCCCCGT
ACGGTTGGACGCCGAGCC-TCTGTAAAGCTCCTTCGACGAGTCGGGTAGTTTGGGAATGCTG
CTCAAAATGGGAGGTAAATTTCTCCTAAAG-CTAAATACCGGCCAGAGACCGATAGCGCACAA
GTAGAGTGATCGAAAGATGAAAAGCACTTTGAAAAGAGGGTTAAACAGCACGTGAAATTGTTG
AAAGGGAAGCGCTTGTGACCAGACTTGCGCCCGGCGGATCATCCGGCGTTCTCGCCGGTG
CACTCCGCCGGGCTCAGGCCAGCATCGGTTCTGCCCGGGGGACAAAGGCCGCGGGAACGT
AGCTCTCTTCGGGGAGTGTTATAGCCCGTGG-CGTAATACCCTGGGG-GGGACCGAGGACCG
CGCTCTG-CAAGGATGCTGGCGTAATGGTCATCAGCGACCCGTCTT????????????????????
???
???
???

???
???
???
???
???
???
???
???
???
???
???
???
???
???
???
???
??
>Distoseptispora_lignicola_GZCC_19_0529
ACCT--------TT-TTGTCT------CGTTGCTTCGGCGGG-CGGCTCC-AGGGCGGAGTCACTGCTCG
TTGGGTCGGGTGCCCGCCGGAGGA---CAAC-CAAAAACTCGT-TAATCTCG-GGTAACCTCAG
AGTATTATT---ATAC-----AA-GCTAT-CAAAACTTTCAGCAACGGATCTCTTGGTTCTGGCATCGAT
GAAGAACGCAGCGAAATGCGATAAGTAATGTGAATTGCAGAATTCAGTGAATCATCGAATCTT
TGAACGCACATTGCGCCCGCTAGTATTCTGGCGGGCATGCCTGTTCGAGCGTCATTTCAA-CC
CTCGGGCCC-TCGTT-------TGGCCCGGTGTTGGGGTGCTACGGTCC-G--C------------------CGG
AC-CCGTAGGCCCTCAAAACCAGTGGCGGGCTCTCCATGACC-CCGAGCGCAGTAGTTT-TCT
C--CTCGCTCAGGCGGCCT-GGCGTG--CT-TCGGCCGTTAAA---TACCCA-C------TGT-TTC---AC
ACAG-AAGCCTTCCGAGTCCCC-TGGAAC------GGGGCGCC-AGAGAGGG-TGAGAGCCCCGT
ATGGTTGGACGCCGAACC-TCTGTAAAGCTCCTTCGACGAGTCGAGTAGTTTGGGAATGCTG
CTCAAAATGGGAGGTAAATGTCTTCTAAAG-CTAAATACCGGCCAGAGACCGATAGCGCACAA
GTAGAGTGATCGAAAGATGAAAAGCACTTTGAAAAGAGGGTTAAATAGCACGTGAAATTGCTG
AAAGGGAAGCGCTTGTGACCAGACTTGCGCCCGGTTGATCATCCAGCGTTCTCGCTGGTGC
ACTCTGCCGGGCTCAGGCCAGCATCGGTTCTCCCAGGGGGATAAAGGTCGCGGGAACGTA
GCTCTCTCCGGGGAGTGTTATAGCCCGCGG-CGGAATGCCCTTAGGGGGGACCGAGGCCCG
CGTTC-G-CAAGGATGCTGGCGTAATGGTCACCAGCGACCCGTCTT???????????????????????
???
???
???
???
???
???
???
???
??ACCCGTGAGCACGCTCTGCTT
GCCTACACCCTGGGTGTCAAGCAGCTCATCGTCGCCATCAACAAGATGGACACTACCAAGTG

GTCTGAGGAGCGTTACAACGAAATCATCAAGGAGACGTCCAACTTCATCAAGAAGGTCGGCT
ACAACCCCAAGCAGGTTGCTTTCGTCCCCATCTCCGGCTTCAACGGCGACAACATGTTGGCC
CCCTCTACCAACTGCCCCTGGTACAAGGGCTGGGAGAAGGAGGTC---AAGGGTGGCAAGGT
TACTGGCAAGACCCTCCTTGAGGCCATCGACGCCATTGAGCCTCCCAAGCGTCCCACAGAC
AAGCCCCTGCGTCTTCCCCTCCAGGATGTCTACAAGATTGGTGGTATCGGAACTGTGCCTGT
CGGCCGTATCGAGACTGGTGTCCTCAAGCCCGGTATGGTCGTTACTTTCGCTCCCTCCAACG
TCACCACTGAAGTCAAGTCCGTCGAGATGCACCACGAGCAGCTGGCTGAGGGTGTTCCCGG
CGACAACGTTGGCTTCAACGTGAAGAACGTCTCCGTCAAGGAAATCCGCCGAGGCAACGTC
GCTGGTGACTCCAAGAACGACCCTCCCGCTGGTGCCGCTTCCTTCACCGCCCAGGTCATCG
TCCTCAACCACCCTGGCCAGGTCGGTGCTGGTTACGCTCCCGTCTTGGATTGCCACACTGC
CCACATCGCTTGCAAGTTCTCTGAGATCCTCGAGAAGATCGATCGCCGAACGGGCAAGTCG
GTCGAGAACAACCCCAAGTTCATCAAGTCTGGTGATGC
>Distoseptispora_lignicola_MFLUCC_18_0198
ACCT--------TT-TTGTCT------CGTTGCTTCGGCGGG-CGGCTCC-AGGGCGGAGTCACTGCTCG
TTGGGTCGGGTGCCCGCCGGAGGA---CAAC-CAAAAACTCGT-TAATCTCG-GGTAACCTCAG
AGTATTATT---ATAC-----AA-GCTAT-CAAAACTTTCAGCAACGGATCTCTTGGTTCTGGCATCGAT
GAAGAACGCAGCGAAATGCGATAAGTAATGTGAATTGCAGAATTCAGTGAATCATCGAATCTT
TGAACGCACATTGCGCCCGCTAGTATTCTGGCGGGCATGCCTGTTCGAGCGTCATTTCAA-CC
CTCGGGCCC-TCGTT-------TGGCCCGGTGTTGGGGTGCTACGGTCC-G--C-----------------CGG
AC-CCGTAGGCCCTCAAAACAAGTGGCGGGCTCTCCATGACC-CCGAGCGCAGTAGTTT-TCT
C--CTCGCTCAGGTGGCCT-GGCGTG--CT-TCGGCCGTTAAA---TATCCA-C------TGT-TTC---ACA
CAG-AAGCCTTCCGAGTCCCC-TGGAAC------GGGGCGCC-AGAGAGGG-TGAGAGCCCCGTA
TGGTTGGACGCCGAACC-TCTGTAAAGCTCCTTCGACGAGTCGAGTAGTTTGGGAATGCTGC
TCAAAATGGGAGGTAAATGTCTTCTAAAG-CTAAATACCGGCCAGAGACCGATAGCGCACAAG
TAGAGTGATCGAAAGATGAAAAGCACTTTGAAAAGAGGGTTAAATAGCACGTGAAATTGCTGA
AAGGGAAGCGCTTGTGACCAGACTTGCGCCCGGTTGATCATCCAGCGTTCTCGCTGGTGCA
CTCTGCCGGGCTCAGGCCAGCATCGGTTCTCCCAGGGGGATAAAGGTCGCGGGAACGTAG
CTCTCTCCGGGGAGTGTTATAGCCCGCGG-CGGAATGCCCTTAGGGGGGACCGAGGCCCGC
G-TTCG-CAAGGATGCTGGCGTAATGGTCACCAGCGACCCGTCTT?????????????????????
??
??
??
??
??
??
??
??
??
??
??
??
??
??
??

??
??
??
??
>Distoseptispora_longispora_HFJAU_0705
ACCT--------TTTATATT-------CGTTGCTTTGGCGGG-CGGCTCC-AGGGCGGAGTCACTACCCGT
TGGGTCGGGCGCCCGCCGAAGGA---CAAC-CAAAACTCGCT-AAATCTGT-ACGGATCTCAGA
GTAAAA------TAT-----CAAGCTAT-CAAAACTTTCAGCAACGGATCTCTTGGTTCTGGCATCGATG
AAGAACGCAGCGAAATGCGATAAGTAATGTGAATTGCAGAATTCAGTGAATCATCGAATCTTT
GAACGCACATTGCGCCCGCCAGCACTCTGGCGGGCATGCCTGTTCGAGCGTCATTTCAA-CC
CTCGGGCCC-TCGTT-------TGGCCCGGTGTTGGGGCGCTACGGGTCTG--TC------------------GG
AC-CTGTAGGCCCTCAAAAGTAGTGGCGGGCTCGCCAGGACT-CCGAGCGTAGTAGTTC-TCC
T--CTCGCTCAGGCGGCCT-GGCGTG--CT-TCGGCCGTTAAA---GACCCAAC------TGTAAT-----C
ACAG-AAGCCTTCCGAGTCCCC-TGGAAC------GGGGCGCC-ATAGAGGG-TGAGAGCCCCGTA
TGGTTGGACGCTGAACC-TCTGTAAAGCTCCTTCGACGAGTCGAGTAGTTTGGGAATGCTGC
TCAAAATGGGAGGTAAATTTCTTCTAAAG-CTAAATACCGGCCAGAGACCGATAGCGCACAAGT
AGAGTGATCGAAAGATGAAAAGCACTTTGAAAAGAGGGTTAAATAGCACGTGAAATTGCTGAA
AGGGAAGCGCTTGTGACCAGACTTGCGCCCGGTTGATCATCCAGCGTTCTCGCTGGTGCAC
TCTGCCGGGCTCAGGCCAGCATCGGCTCTCCCAGGGGGATAAAGGTCGCGGGAACGTAGC
TCTCTCCGGGGAGTGTTATAGCCCGTGG-CGGAATGCCCTTAGGGGGGGCCGAGGCCCGCG
-TTCG-CAAGGATGCTGGCGTAATGGTCACCAGCGACCCGTCTT??????????????????????????
??
??
??
??
??
??
??
??
??
??
??
??
??
??
??
??
??
???
>Distoseptispora_martinii_CGMCC_3_18651
ACCT--------AT--AGTT-------CGTTGCTCCGGCGGG-CGGTTCC-AGGGCGGAGCCACTGCCCGT
TAGGTCGGGCGTCCGCCGGAGGA---CAGC-CAAAACTAGCT-AAATCTCG-GGTTAACTCAGA
GTATTATGA--AAA---------GCTAT-CAAAACTTTCAGCAACGGATCTCTTGGTTCTGGCATCGATG

AAGAACGCAGCGAAATGCGATAAGTAATGTGAATTGCAGAATTCAGTGAATCATCGAATCTTT
GAACGCATATTGCGCCCGCCAGCACTCTGGCGGGCATGCCTGTTCGAGCGTCATTTCAA-CC
CTCGGGCCCCTCGTT-------TGGCCCGGTGTTGGGGCGCTACGGTTTCCTTCG---------------GG
GGAC-TCGTAGGCCCTTAAAACCAGTGGCGGGCTCGCCAGGACT-CCGAGCGTAGTAGTTT-T
CTC--CTCGCTCAGGCGGCCT-GGCGTG--CT-TCGGCCGTTA-----------------------------------GCC
TTCCGAGTCCCC-TGGAAC------GGGGCGCC-AGAGAGGG-TGAGAGCCCCGTACGGTCGGA
CGCCGAACC-TCTGTAAAGCTCCTTCGACGAGTCGAGTAGTTTGGGAATGCTGCTCAAAATG
GGAGGTAAATTTCTTCTAAAG-CTAAATACCGGCCAGAGACCGATAGCGCACAAGTAGAGTGA
TCGAAAGATGAAAAGCACTTTGAAAAGAGGGTTAAATAGCACGTGAAATTGCTGAAAGGGAA
GCGCTTGTGACCAGACTTGCGCCCGGTTGATCATCCAGCGTTCTCGCTGGTGCACTCTGCC
GGGCCCAGGCCAGCATCGGCTCTCCCAGGGGGATAAAGGTCGCGGGAACGTAGCTCTCTC
CGGGGAGTGTTATAGCCCGTGG-CGGAATGCCCTTAGGGGGGGCCGAGGCCCGCGTTC-G-C
AAGGATGCTGGCGTAATGGTCATCAGCGACCCGTCTT???????????????????????????????
??
??
??
??
??
??
??
??
??
??
??
??
??
??
??
??
??
??
???
>Distoseptispora_meilingensis_JAUCC_4727
ACCT--------ATCGTCAAA------TGTTGCTTCGGCGGG-CGGCCCC-TGGGAGGGGCCAAGGCCC
GTTGGGTTGGGTGCCCGCCGGAGGA---CAGC-CGAAAACTCTTTT----ATCAGGTACTGTCTGA
GTAAA------CTATA--AAAA--CTAT-AAAAACTTTCAACAACGGATCTCTTGGTTCTGGCATCGATG
AAGAACGCAGCGAAATGCGATAAGTAATGTGAATTGCAGAATTCAGTGAATCATCGAATCTTT
GAACGCACATTGCGCCCGCCAGCACTCTGGCGGGCATGCCTGTTCGAGCGTCATTTCA-ACC
CTCAGGCCC-CA-------GTTTGGCCTGGTGTTGGGGCACTGCCGTCCCGCCC---------------GTAC
GGGGCGCGCAGGTCCTGAAAAACAGTGGCGGGCTCGCTAGCAC-TCCGGGCGTAGTAGA-A
TTAT-ATCTCGTCTAGGTGTTCT-GGCGCG--GT-CTTGCCGTCAAAAC-CCCCA---------ATTTTTA
C-------ACAAGCCTTCTAAGTCCCC-TAGAAC------GGGGCGCC-ACAGAGGG-TGAGAGCCCCG
TACAGTTGGACACCGAACC-TTTGTAAAGCTCCTTCGACGAGTCGAGTAGTTTGGGAATGCTG
CTCAAAATGGGAGGTATATTTCTTCTAAAG-CTAAATACCGGCCAGAGACCGATAGCGCACAAG

TAGAGTGATCGAAAGATGAAAAGCACTTTGAAAAGAGGGTTAAATAGCACGTGAAATTGTTGA
AAGGGAAGCGCTTGTGACCAGACTTGTGCCCGGTGAATCATCCAGCGTTCTCGCTGGTGCA
CTTTGCCGGGCTCAGGCCAGCATCGGTTCTCCCAGGGGGATAAAGGTCTCGGGAACGTAGC
TCTCTTCGGGGAGTGTTATAGCCCGTGG-CATAATACC-TTTGGGGGGACCGAGGACCGCGCT
CTG-CAAGGATGCTGGCGTAATGGTCATCAGCGACCCGTCTT?????????????????????????
??
??
??
??
??
??
??
??
???ACTCGTGAGCACGCCCTGCTTGCC
TACACCCTGGGTGTCAAGCAGCTCATCTGCGCCATCAACAAGATGGACACCACCAAGTGGTC
AGAGGACCGTTTCAACGAAATCATCAAGGAGACGTCCAACTTCATCAAGAAGGTCGGCTACA
ACCCCAAGCAGGTCGCCTTCGTCCCTATCTCTGGCTTCCACGGCGACAACATGTTGGCTGC
CTCCAGCAACTGCCCCTGGTACAAGGGATGGGAGAAGGAGGGCGCCAAGGGTGCCAAGTC
TTCTGGTAAGACCCTGCTCGAGGCCATCGACGCCATCGAGCAACCCAAGCGTCCTACCGAC
AAGCCTCTGCGTCTCCCGCTCCAGGATGTCTACAAGATCGGTGGTATCGGAACTGTCCCTGT
CGGCCGTATCGAGACTGGTATCCTCAAGCCCGGTATGGTCGTCACTTTCGCTCCCTCCAACG
TCACCACTGAAGTCAAGTCCGTCGAGATGCACCACGAGCAGCTCACTGAGGGTGTTCCCGG
CGACAACGTCGGCTTCAACGTGAAGAACGTCTCCGTCAAGGAAATCCGCCGTGGAAACGTC
GCTGGTGACTCCAAGAACGACCCTCCTGCTGGCTGCGCCAACTTCACTGCTCAGGTCATTG
TCCTCAACCACCCTGGTCAGGTCGGCCCTGGTTACGCTCCCGTCTTGGATTGCCACACCGC
CCACATCGCCTGCAAGTTCTCTGAGATCACCGAGAAGATCGACCGCCGTACCGGCAAGTCA
GTTGAGAACAACCCCAAGTTCATCAAGTCTGGTGATGC
>Distoseptispora_meilingensis_JAUCC_4728
ACCT--------ATCGTCAAA------TGTTGCTTCGGCGGG-CGGCCCC-TGGGAGGGGCCAAGGCCC
GTTGGGTTGGGTGCCCGCCGGAGGA---CAGC-CGAAAACTCTTTT----ATCAGGTACTGTCTGA
GTAAA------CTATA--AAAA--CTAT-AAAAACTTTCAACAACGGATCTCTTGGTTCTGGCATCGATG
AAGAACGCAGCGAAATGCGATAAGTAATGTGAATTGCAGAATTCAGTGAATCATCGAATCTTT
GAACGCACATTGCGCCCGCCAGCACTCTGGCGGGCATGCCTGTTCGAGCGTCATTTCA-ACC
CTCAGGCCC-CA-------GTTTGGCCTGGTGTTGGGGCACTGCCGTCCCGCCC---------------GTAC
GGGGCGCGCAGGTCCTGAAAAACAGTGGCGGGCTCGCTAGCAC-TCCGGGCGTAGTAGA-A
TTAT-ATCTCGTCTAGGTGTTCT-GGCGCG--GT-CTTGCCGTCAAAAC-CCCCA---------ATTTTTA
C-------ACAAGCCTTCTAAGTCCCC-TAGAAC------GGGGCGCC-ACAGAGGG-TGAGAGCCCCG
TACAGTTGGACACCGAACC-TTTGTAAAGCTCCTTCGACGAGTCGAGTAGTTTGGGAATGCTG
CTCAAAATGGGAGGTATATTTCTTCTAAAG-CTAAATACCGGCCAGAGACCGATAGCGCACAAG
TAGAGTGATCGAAAGATGAAAAGCACTTTGAAAAGAGGGTTAAATAGCACGTGAAATTGTTGA
AAGGGAAGCGCTTGTGACCAGACTTGTGCCCGGTGAATCATCCAGCGTTCTCGCTGGTGCA
CTTTGCCGGGCTCAGGCCAGCATCGGTTCTCCCAGGGGGATAAAGGTCTCGGGAACGTAGC
TCTCTTCGGGGAGTGTTATAGCCCGTGG-CATAATACC-TTTGGGGGGACCGAGGACCGCGCT
CTG-CAAGGATGCTGGCGTAATGGTCATCAGCGACCCGTCTT?????????????????????????

??
??
??
??
??
??
??
??
??ACTCGTGAGCACGCCCTGCTTGCC
TACACCCTGGGTGTCAAGCAGCTCATCTGCGCCATCAACAAGATGGACACCACCAAGTGGTC
AGAGGACCGTTTCAACGAAATCATCAAGGAGACGTCCAACTTCATCAAGAAGGTCGGCTACA
ACCCCAAGCAGGTCGCCTTCGTCCCTATCTCTGGCTTCCACGGCGACAACATGTTGGCTGC
CTCCAGCAACTGCCCCTGGTACAAGGGATGGGAGAAGGAGGGCGCCAAGGGTGCCAAGTC
TTCTGGTAAGACCCTGCTCGAGGCCATCGACGCCATCGAGCAACCCAAGCGTCCTACCGAC
AAGCCTCTGCGTCTCCCGCTCCAGGATGTCTACAAGATCGGTGGTATCGGAACTGTCCCTGT
CGGCCGTATCGAGACTGGTATCCTCAAGCCCGGTATGGTCGTCACTTTCGCTCCCTCCAACG
TCACCACTGAAGTCAAGTCCGTCGAGATGCACCACGAGCAGCTCACTGAGGGTGTTCCCGG
CGACAACGTCGGCTTCAACGTGAAGAACGTCTCCGTCAAGGAAATCCGCCGTGGAAACGTC
GCTGGTGACTCCAAGAACGACCCTCCTGCTGGCTGCGCCAACTTCACTGCTCAGGTCATTG
TCCTCAACCACCCTGGTCAGGTCGGCCCTGGTTACGCTCCCGTCTTGGATTGCCACACCGC
CCACATCGCCTGCAAGTTCTCTGAGATCACCGAGAAGATCGACCGCCGTACCGGCAAGTCA
GTTGAGAACAACCCCAAGTTCATCAAGTCTGGTGATGC
>Distoseptispora_mengsongensis_HJAUP_C2126
ACCT--------CT-ATATT-------CGTTGCTTTGGCGGG-CGGCTCC-AGGGCGGAGCCACTGCTCGT
TGGGTCGGGCGCCCGCCAAAGGA---CAAC-CAAAACTCATT-GAATCTGT-GCGGATCTCAGA
GTAAAAA-----TAT-----CAAGCTAT-CAAAACTTTCAGCAACGGATCTCTTGGTTCTGGCATCGAT
GAAGAACGCAGCGAAATGCGATAAGTAATGTGAATTGCAGAATTCAGTGAATCATCGAATCTT
TGAACGCACATTGCGCCCGCCAGCACTCTGGCGGGCATGCCTGTTCGAGCGTCATTTCAA-C
CCTCGGGCCC-TCGTT-------TGGCCCGGTGTTGGGGCGCTACGGGTCCC-ATC-----------------G
GAC-CTGTAGGCCCTCAAAACCAGTGGCGGGCTCGCCAGGACT-CCGAGCGCAGTAGTTC-T
CCT--CTCGCTCAGGCGGCCT-GGCGTG--CT-TCGGCCGTTAAA---TACCCAGC------GGTAATT
CG----CAC-ATGCCTTCCGAGTCCCC-TGGAAC------GGGGCGCC-AGAGAGGG-TGAGAGCCC
CGTATGGTTGGACGCCGAACC-TCTGTAAAGCTCCTTCGACGAGTCGAGTAGTTTGGGAATG
CTGCTCAAAATGGGAGGTAAATTTCTTCTAAAG-CTAAATACCGGCCAGAGACCGATAGCGCA
CAAGTAGAGTGATCGAAAGATGAAAAGCACTTTGAAAAGAGGGTCAAATAGCACGTGAAATTG
CTGAAAGGGAAGCGCTTGTGACCAGACTTGCGCCCGGTTGATCATCCAGCGTTCTCGCTGG
TGCACTCTGCCGGGCTCAGGCCAGCATCGGCTCTCCCAGGGGGATAAAGGTCGCGGGAAC
GTAGCTCTCTCCGGGGAGTGTTATAGCCCGTGG-CGGAATGCCCTTAGGGGGGGCCGAGGC
CCGCGTTC-G-CAAGGATGCTGGCGTAATGGTCACCAGCGACCCGTCTT?????????????????
??
??
??
??
??

??
??
??
??ACTCGTGAGCACGCTCT
GCTCGCCTACACACTTGGTGTGAAGCAGCTCATCGTCGCCATCAACAAGATGGACACCACCA
AGTGGTCCGAGAGCCGTTACAACGAAATCATCAAGGAGACGTCCAACTTCATCAAGAAGGTC
GGCTACAACCCCAAGCAGGTCGCCTTCGTTCCCATCTCCGGCTTCAACGGCGACAACATGC
TTGCCCCCTCCTCCAACTGCCCCTGGTACAAGGGTTGGGAGAAGGAGATC---AAGGGTGGC
AAGGTCTCTGGCAAGACCCTCTTGGAGGCTATCGATGCCATTGAGCCTCCCAAGCGTCCCAC
AGACAAGCCTCTCCGTCTACCCCTTCAGGATGTCTACAAGATCGGTGGTATCGGAACTGTGC
CTGTCGGCCGTATCGAGACTGGTATCCTCAAGCCCGGTATGGTCGTCACATTCGCTCCCTCC
AACGTCACCACTGAAGTCAAGTCCGTCGAGATGCACCACGAGCAGCTGCAGGAGGGTGTCC
CCGGCGACAACGTCGGCTTCAACGTGAAGAACGTCTCCGTCAAGGAAATCCGCCGAGGCAA
CGTCGCTGGTGACTCCAAGAACGACCCCCCTGCCGGTGCCGCTTCCTTCACCGCTCAGGTC
ATCGTCCTCAATCACCCCGGTCAGGTCGGTGCTGGCTACGCTCCCGTCTTGGATTGCCACAC
TGCCCACATCGCTTGCAAGTTCTCTGAGATCCTTGAGAAGATCGACCGCCGAACGGGCAAGT
CTGTTGAGAACAACCCCAAGTTCATCAAGTCTGGTGACGC
>Distoseptispora_multiseptata_MFLUCC_15_0609
ACCT--------TT-TCGTT-------CGTTGCTTTGGCGGG-CGGCTCC-AGGGCGGAGCCACTGTCCG
TTGGGTCGGGTGCCCGCCGAAGGA---CAAC-CAAAAACTCGTTAAGTTTTGTACGGATCTCAG
AGTTAAAA-----TAT-----CAAGCTAT-CAAAACTTTCAGCAACGGATCTCTTGGTTCTGGCATCGAT
GAAGAACGCAGCGAAATGCGATAAGTAATGTGAATTGCAGAATTCAGTGAATCATCGAATCTT
TGAACGCACATTGCGCCCGCCAGCACTCTGGCGGGCATGCCTGTTCGAGCGTCATTTCAA-C
CCTCGGGCCC-TAGTT-------TGGCCCGGTGTTGGGGCGCTACGGGTCTC--CC------------------G
GAC-CCGTAGGCCCTTAAAGACAGTGGCGGGCTCGCCAGGACT-CCGAGCGCAGTAGTTC-T
CCT--CTCGCTCAGGCGGCCT-GGCGTG--CT-TCGGCCGTTAAACT-CCCCCAAC------TGTAAA-
----CACAGTAAGCCTTCCGAGTCCCC-TGGAAC------GGGGCGCC-AGAGAGGG-TGAGAGCCC
CGTATGGTTGGACGCCGAACC-TCTGTAAAGCTCCTTCGACGAGTCGAGTAGTTTGGGAATG
CTGCTCAAAATGGGAGGTAAATTTCTTCTAAAG-CTAAATACCGGCCAGAGACCGATAGCGCA
CAAGTAGAGTGATCGAAAGATGAAAAGCACTTTGAAAAGAGGGTCAAATAGCACGTGAAATTG
CTGAAAGGGAAGCGCTTGTGACCAGACTTGCGCCCGGTTGATCATCCAGCGTTCTCGCTGG
TGCACTCTGCCGGGTTCAGGCCAGCATCGGCTCTCCCAGGGGGATAAAGGTCGCGGGAAC
GTAGCTCTCTCCGGGGAGTGTTATAGCCCGTGG-CGGAATGCCCTTAGGGGGGGCCGAGGC
TCGCGTTC-G-CAAGGATGCTGGCGTAATGGTCACCAGCGACCCGTCTT?????????????????
??
??
??
??
??
??
??
??
??ACTCGTGAGCACGCTCT
GCTTGCCTACACCCTGGGTGTGAAGCAGCTTATCGTCGCCATCAACAAGATGGACACCACCA

AGTGGTCCGAGAGCCGTTACAACGAAATTATCAAGGAGACGTCCAACTTCATCAAGAAGGTC
GGCTACAACCCCAAGCAGGTGGCCTTCGTTCCCATCTCTGGCTTCAACGGCGACAACATGCT
TGCCCCCTCCACCAACTGCCCCTGGTACAAGGGTTGGGAGAAGGAGGTC---AAGGGCGGCA
AGGCCACTGGCAAGACTCTTCTTGAGGCCATCGATGCCATTGAGCCTCCAAAGCGTCCTACG
GACAAGCCGCTGCGTCTCCCCCTCCAGGATGTTTACAAGATCGGCGGTATTGGAACTGTGCC
CGTCGGCCGTATCGAGACTGGTATCCTCAAGCCCGGTATGGTCGTCACGTTCGCTCCCTCCA
ACGTTACCACTGAAGTCAAGTCCGTCGAGATGCACCACGAGCAGCTGCAGGAGGGTGTCCC
CGGTGACAACGTCGGCTTCAACGTGAAGAACGTCTCCGTCAAGGAAATTCGCCGAGGCAAC
GTTGCTGGTGACTCCAAGAACGATCCTCCTGCGGGCGCCGCTTCCTTCACCGCCCAGGTCA
TCGTCCTCAACCACCCCGGCCAGGTCGGCGCTGGATACGCTCCCGTCTTGGATTGCCACAC
TGCCCACATCGCCTGCAAGTTCTCCGAGATCCTTGAGAAGATCGACCGCCGAACGGGCAAG
TCTGTTGAGAACAACCCCAAGTTCATCAAGTCTGGTGACGC
>Distoseptispora_multiseptata_MFLU_17_0856
ACCT--------TT-TCGTT-------CGTTGCTTTGGCGGG-CGGCTCC-AGGGCGGAGCCACTGTCCG
TTGGGTCGGGTGCCCGCCGAAGGA---CAAC-CAAAAACTCGTTAAGTTTTGTACGGATCTCAG
AGTTAAAA-----TAT-----CAAGCTAT-CAAAACTTTCAGCAACGGATCTCTTGGTTCTGGCATCGAT
GAAGAACGCAGCGAAATGCGATAAGTAATGTGAATTGCAGAATTCAGTGAATCATCGAATCTT
TGAACGCACATTGCGCCCGCCAGCACTCTGGCGGGCATGCCTGTTCGAGCGTCATTTCAA-C
CCTCGGGCCC-TAGTT-------TGGCCCGGTGTTGGGGCGCTACGGGTCTC--CC------------------G
GAC-CCGTAGGCCCTCAAAGACAGTGGCGGGCTCGCCAGGACT-CCGAGCGCAGTAGTTC-T
CCT--CTCGCTCAGGCGGCCT-GGCGTG--CT-TCGGCCGTTAAACC-CCCCCAAC------TGTAAA-
----CACAGTAAGCCTTCCGAGTCCCC-TGGAAC------GGGGCGCC-AGAGAGGG-TGAGAGCCC
CGTATGGTTGGACGCCGAACC-TCTGTAAAGCTCCTTCGACGAGTCGAGTAGTTTGGGAATG
CTGCTCAAAATGGGAGGTAAATTTCTTCTAAAG-CTAAATACCGGCCAGAGACCGATAGCGCA
CAAGTAGAGTGATCGAAAGATGAAAAGCACTTTGAAAAGAGGGTCAAATAGCACGTGAAATTG
CTGAAAGGGAAGCGCTTGTGACCAGACTTGCGCCCGGTTGATCATCCAGCGTTCTCGCTGG
TGCACTCTGCCGGGTTCAGGCCAGCATCGGCTCTCCCAGGGGGATAAAGGTCGCGGGAAC
GTAGCTCTCTCCGGGGAGTGTTATAGCCCGTGG-CGGAATGCCCTTAGGGGGGGCCGAGGC
TCGCGTTC-G-CAAGGATGCTGGCGTAATGGTCACCAGCGACCCGTCTTTATCGCTCATGTGT
TATGTGAGCGTCGGCACTCCCGCAGAGCCAATTGTCGAGTTCATGATTGCTAGGAACATGGA
AGTACTCGAGGAATATGAGCCTTTAAGGTACCCTAATGCGACCAAAATCTTCGTCAATGGCAC
ATGGGTTGGTGTTCACCAAGACCCGAAGCATCTTGTGAGCTTGGTTCAGGACTTACGCCGGA
GGGGCGTCATCAACTTTGAGGTCTCCCTCATTCGGGACATCCGCGATCGAGAGTTCAAAATT
TTCTCGGATGCTGGTCGTGTTATGCGTCCTCTGTTTGCTGTGGAGCAGTCAGAC---AACTCGA
CGAAG---GGCCTTGAAAAAGGCTCTTTATTACTCACAAAGGAGCATATTCAAAAATTGAGAGCA
GATGAGGATCTACCCAAGGATGATGACGACTATTTCGGCTGGGATGGTCTTCTCAGAGCCGG
CGCTATAGAATATCTTGACGCCGAAGAGGAAGAGACAGCCATGATTTGCATGACTCCAGAAGA
CCTAGAGGCTTATCGGCTGCAAAAAGCTGGATTAGACGTACCGGAAGACGATGACGAGGCC
G---ACCCCAATAGGCGCCTGAAGACAAAACTGAACCCCACCACTCACATGTACACACATTGTG
AGATTCATCCGAGTATGCTCCTGGGGATATGCGCGAGCATCATCCCACTCGTGAGCACGCTC
TGCTTGCCTACACCCTGGGTGTGAAGCAGCTTATCGTCGCCATCAACAAGATGGACACCACC
AAGTGGTCTGAGAGCCGTTACAACGAAATTATCAAGGAGACGTCCAACTTCATCAAGAAGGT
CGGCTACAACCCCAAGCAGGTGGCCTTCGTTCCCATCTCTGGCTTCAACGGCGACAACATG
CTTGCCCCCTCCACCAACTGCCCCTGGTACAAGGGTTGGGAGAAGGAGGTC---AAGGGCGG

CAAGGCCACTGGCAAGACTCTTCTTGAGGCCATCGATGCCATTGAGCCTCCAAAGCGTCCTA
CGGACAAGCCGCTGCGTCTCCCCCTCCAGGATGTTTACAAGATCGGCGGTATTGGAACTGTG
CCCGTCGGCCGTATCGAGACTGGTATCCTCAAGCCCGGTATGGTCGTCACGTTCGCTCCCTC
CAACGTTACCACTGAAGTCAAGTCCGTCGAGATGCACCACGAGCAGCTGCAGGAGGGTGTC
CCCGGTGACAACGTCGGCTTCAACGTGAAGAACGTCTCCGTCAAGGAAATTCGCCGAGGCA
ACGTTGCTGGTGACTCCAAGAACGATCCTCCTGCGGGCGCCGCTTCCTTCACCGCTCAGGT
CATCGTCCTCAACCACCCCGGCCAGGTCGGCGCTGGATACGCTCCCGTCTTGGATTGCCAC
ACTGCCCACATCGCCTGCAAGTTCTCCGAGATCCTTGAGAAGATCGACCGCCGAACGGGCA
AGTCTGTTGAGAACAACCCCAAGTTCATCAAGTCTGGTGACGC
>Distoseptispora_nabanheensis_HJAUP_C2003
ACCT--------TT-ATATT-------CGTTGCTTTGGCGGG-CGGCTCC-AGGGCGGAGCCACTGCTCGT
TGGGTCGGGCGCCCGCCGAAGGA---CAAC-CAAAA-CTCTTTAAATCTTT-GCGGATCTCAGAG
TAAAAA-----TAT-----CAAGCTAT-CAAAACTTTCAGCAACGGATCTCTTGGTTCTGGCATCGATGA
AGAACGCAGCGAAATGCGATAAGTAATGTGAATTGCAGAATTCAGTGAATCATCGAATCTTTG
AACGCACATTGCGCCCGCCAGCACTCTGGCGGGCATGCCTGTTCGAGCGTCATTTCAA-CCC
TCGGGCCC-TCGTT-------CGGCCCGGTGTTGGGGCGCTACGGGTCCTATCG-----------------G-A
C-CTGTAGGCCCTTAAAATTAGTGGCGGGCTCGCCAGGACT-CCGAGCGTAGTAGTTT-TCCT--
CTCGTTCAGGCGGTCT-GGCGTG--CT-TCGGCCGTTAAA---AACCCAGC------GGTAAT-TCGCA
CAA----GCCTTCCGAGTCCCC-TGGAAC------GGGGCGCC-AGAGAGGG-TGAGAGCCCCGTAT
GGTTGGACGCCGAACC-TCTGTAAAGCTCCTTCGACGAGTCGAGTAGTTTGGGAATGCTGCT
CAAAATGGGAGGTAAATTTCTTCTAAAG-CTAAATACCGGCCAGAGACCGATAGCGCACAAGT
AGAGTGATCGAAAGATGAAAAGCACTTTGAAAAGAGGGTCAAATAGCACGTGAAATTGCTGA
AAGGGAAGCGCTTGTGACCAGACTTGCGCCCGGTTGATCATCCAGCGTTCTCGCTGGTGCA
CTCTGCCGGGCTCAGGCCAGCATCGGCTCTCCCAGGGGGATAAAGGTCGCGGGAACGTAG
CTCTCTCCGGGGAGTGTTATAGCCCGTGG-CGGAATGCCCTTAGGGGGGGCCGAGGCCCGC
GTTC-G-CAAGGATGCTGGCGTAATGGTCACCAGCGACCCG-TCT???????????????????????
???
???
???
???
???
???
???
???
??ACTCGTGAGCACGCTCTTCTTG
CCTACACGCTAGGTGTGAAGCAGCTCATCGTTGCCATCAACAAGATGGACACCACCAAGTGG
TCTGAGAGCCGTTACAACGAAATTATCAAGGAGACGTCCAACTTCATCAAGAAGGTCGGCTA
CAACCCCAAGCAGGTCGCCTTCGTTCCCATCTCCGGCTTCAACGGCGACAACATGCTTGCC
CCGTCCTCCAACTGCCCCTGGTACAAGGGTTGGGAGAAGGAGATC---AAGGGTGGCAAGGC
CTCTGGCAAGACCCTCCTCGAGGCTATCGACGCCATTGAGCCTCCCAAGCGTCCCACAGAC
AAGCCTCTCCGTCTCCCCCTTCAGGATGTCTACAAGATCGGTGGTATCGGAACTGTGCCCGT
CGGCCGTATCGAGACTGGTGTCCTCAAGCCCGGTATGGTCGTCACGTTCGCTCCCTCCAAC
GTCACCACTGAAGTCAAGTCCGTCGAGATGCACCACGAGCAGCTGCAGGAGGGTGTCCCC
GGCGACAACGTTGGCTTCAACGTGAAGAACGTCTCCGTCAAGGAAATCCGCCGAGGCAACG

TCGCTGGTGACTCCAAGAACGATCCTCCTGCTGGTGCCGCTTCCTTCACCGCTCAGGTCATC
GTCCTCAACCACCCCGGTCAGGTCGGTGCTGGTTACGCTCCCGTCTTGGATTGCCACACTG
CCCACATTGCCTGCAAGTTCTCTGAGATCCTTGAGAAGATCGACCGCCGAACGGGCAAGTCT
GTTGAGAACAACCCCAAGTTCATCAAGTCTGGTGACGC
>Distoseptispora_neorostrata_MFLUCC_18_0376
ACCT--------GT---TCTG------TACTGCTCCGGCGGG-TGGCTCC-AGGGCGGAGCCACTGCCCG
TTCGCTCGGGTGCCCGCCGAAGC---TCACT-ACAAACTCTGTCATC--GCGGGGTGTCATCAG
AGCAAGA------CATA-----AAGCTAT-AAAAACTTTCAGCAACGGATCTCTTGGTTCTGGCATCGAT
GAAGAACGCAGCGAAATGCGATAAGTAATGTGAATTGCAGAATTCAGTGAATCATCGAATCTT
TGAACGCACATTGCGCCCGCCAGCACTCTGGCGGGCATGCCTGTCCGAGCGTCATTTCAAA
CCCTCAGGCCCCTCCAG-------CGGCCTGGCGTTGGGGCGCTACGCCGCCTATAG--------------
--------GCGTAGGCCCTCAAAACCAGTGGCGGGACCGCGAGGCTT-CCGGGCGCAGTAGTTT-A
CCA-CCTCGCCCAGGCGGT-C-TAGCGG--CC-TCTGCCGTTAAAAT-ACCCCCAC------ATCAGA-
-----------GCCTTCTGAGTCCCC-TGGAAC------GGGGCGCC-ATAGAGGG-TGAGAGCCCCGTAT
AGTCGGCCACCGAGCC-TCTGTAAAGCTCCTTCGACGAGTCGAGTAGTTTGGGAATGCTGCT
CTAAATGGGAGGTAAATTTCTTCTAAAG-CTAAATACCGGCCAGAGACCGATAGCGCACAAGTA
GAGTGATCGAAAGATGAAAAGCACTTTGAAAAGAGGGTTAAATAGCACGTGAAATTGCTGAAA
GGGAAGCGCTTGTGACCAGACTTGCGCCCGGCGGATCATCCGGCGTTCTCGCCGGTGCAC
TCCACCGGGTCCAGGCCAGCATCGGTTTCGCCAGGGGGACAAAAGCCGTGGGAACGTAGC
TCTCTTCGGGGAGTGTTATAGCCCATGG-CACAATGCCCTTAGGCGGGACCGAGGTTCGCGC
TCTG-CAAGGATGCTGGCGTAATGGTCATCAGCGACCCGTCTT????????????????????????
??
??
??
??
??
??
??
??
??
??
??
??
??
??
??
??
??
??
??
>Distoseptispora_nonrostrata_KUNCC_21_10730
ACCT--------GTC--GTT-------CGTTGCTTCGGCGGG-CGGCTCC-AGGGCGGAGCCACTGCCCG
TTAGGTCGGGCGCCCGCCGGAGGA---CACA-CAAAACTCGTC--GATCTA-GTGTATCATCAGA
GTATTA----AAAAA-----CAAGCTAT-CAAAACTTTCAGCAACGGATCTCTTGGTTCTGGCATCGAT

GAAGAACGCAGCGAAATGCGATAAGTAATGTGAATTGCAGAATTCAGTGAATCATCGAATCTT
TGAACGCACATTGCGCCCGCCAGTACTCTGGCGAGCATGCCTGTTCGAGCGTCATTTCAA-C
CCTCGGGCCCCCC-GT-----T-TGGCCCGGTGTTGGGGTGCTACAGGTCCTCCA-------------------
GGACCTGTAGGCCCTCAAAACCAGTGGCGGGCTCGCCATGGCTTCCGAGCGCAGTAGTTG-
TCTC--CTCGCTCAGGTGGTCT-GGCGTG--CT-TCGGCCGTTAAA---CACCCG-C------TGTATAC-
----ACAG-AAGCCTTCCGAGTCCCC-TGGAAC------GGGGCGCC-AGAGAGGG-TGAGAGCCCC
GTATGGTCGGACGCCGAACC-TTTGTAAAGCTCCTTCGACGAGTCGAGTAGTTTGGGAATGCT
GCTCAAAATGGGAGGTAAATTTCTTCTAAAG-CTAAATACCGGCCAGAGACCGATAGCGCACA
AGTAGAGTGATCGAAAGATGAAAAGCACTTTGAAAAGAGGGTTAAATAGCACGTGAAATTGCT
GAAAGGGAAGCGCTTGTGACCAGACTTGCGCCCGGTTGATCATCCAGCGTTCTCGCTGGTG
CACTCTGCCGGGCTCAGGCCAGCATCGGCTCTCCCAGGGGGATAAAGGTCGCGGGAACGT
AGCTCTCTCCGGGGAGTGTTATAGCCCGTGG-CGGAATACCCTTAGGGGGGGTCGAGGCCC
GCGTTC-G-CAAGGATGCTGGCGTAATGGTCACCAGCGACCCGTCTTTATCACTCATGTGTTAT
GTCAGCGTCGGAACACCTGCGGAGCCAATCGTCGATTTCATGGTTGCCAGAAACATGGAGC
TCCTCGAAGAATACGATCCTCTAAGGCTACCGAATGCGACCAAAGTCTTTGTCAACGGTTCGT
GGGTGGGTTGCCACCAAGATCCGAGCCATCTTGTGCGCTTGGTCCAGGAGTTGCGCCGCAA
GGGCGTTATCAATTTCGAGGTCTCGCTAATCAGAGATATACGCGATCGAGAGTTCAAGATTTT
CTCGGATGCGGGACGTGTCATGAGACCGCTTTTCGCTGTAGAGCAGTCAGAC---AACTCAGA
AAAG---GGCATTGAAAAAGGTGCCTTGTTGCTCACCAAGGAGCACATTCAGAGGCTAAGAGC
AGACGAGGAACTGTCCAAGGACGACGCAGCATATTTCGGTTGGGACGGCTTGCTCAACGCT
GGCGCTATAGAGTATCTTGACGCGGAAGAGGAAGAGACGGCGATGATTTGCATGACGCCAGA
AGATCTAGAGGCTTATCGACTACAAAAAGCCGGCTTCGACGTGCCGGATGAGGACGACGAA
GCGG---ATCCCAACAGGCGTCTCAAAACGAAGCTGAATCCCACTACCCATATGTACACACATT
GTGAGATTCATCCTAGCATGCTCTTAGGGATCTGCGCAAGCATTATCCCACTCGTGAGCACGC
CCTGCTTGCCTACACCCTAGGTGTCAAGCAGCTTATCGTCGCCATCAACAAGATGGACACCA
CCAAGTGGTCTGAGGATCGTTACAACGAAATCATCAAGGAGACGTCCAACTTCATCAAGAAG
GTCGGATACAACCCCAAGCAGGTGGCTTTCGTTCCCATCTCCGGCTTCAACGGTGACAACAT
GTTGGCGCCGTCCACCAACTGCCCCTGGTACAAGGGCTGGGAGAAGGAGATC---AAGGGTA
ACAAGATCACTGGCAAGACGCTCCTCGAGGCCATCGATGCCATTGAGCAACCCAAGCGTCC
CACAGACAAGCCTCTCCGTCTTCCCCTTCAGGATGTCTACAAGATCGGTGGTATTGGCACTG
TGCCCGTCGGCCGTATCGAGACTGGTGTCCTCAAGCCCGGTATGGTCGTTACATTCGCTCCC
TCCAACGTCACCACTGAAGTCAAGTCCGTCGAGATGCACCACGAGCAGCTCTCCGAGGGTA
TGCCTGGTGACAACGTTGGCTTCAACGTGAAGAACGTCTCCGTCAAGGAAATCCGCCGTGG
CAACGTCGCTGGTGACTCCAAGAACGATCCTCCTGCCGGTGCTGCTTCCTTCACTGCCCAG
GTCATCGTCCTCAACCACCCTGGTCAGGTCGGCGCTGGCTACGCCCCCGTCTTGGATTGCC
ACACCGCCCACATCGCCTGCAAGTTCTCTGAGATCCTCGAGAAGATTGACCGCCGAACGGG
CAAGTCTGTCGAGAACAACCCCAAGTTCATCAAGTCTGGTGACGC
>Distoseptispora_obclavata_MFLUCC_18_0329
ACCT--------ATC--GTT-------CGTTGCTCCGGCGGG-CGGCTCC-AGGGCGGAGCCACTGCCAG
TTGGGTCGGGCGCCCGCCGGAGGA---CAAC-TAAAACTCGTC-TAGTCTC-GGGTAACGTCAG
AGTACAA----AAAA-----AAAAGCTAT-CAAAACTTTCAGCAACGGATCTCTTGGTTCTGGCATCGA
TGAAGAACGCAGCGAAATGCGATAAGTAATGTGAATTGCAGAATTCAGTGAATCATCGAATCT
TTGAACGCACATTGCGCCCGCCAGCACTCTGGCGGGCATGCCTGTTCAAGCGTCATTTCAA-
CCCTCGGGCC-TCCCCT----GTGTGGCCCGGTGTTGGGGCGCTACCTTCCCCCC----------------

AGGGGTTCCGGTAGGCCCTTAAAACCAGTGGCGGGCTCGCCATGGCTTCCGAGCGCAGTAG
TTC-TCTC--CTCGCTCAGGCGGTCT-GGCTGG--CC-TCTGCCGTTAAA---CATACG-C------TGTA
TTAC----GCAA-AAGCCTTCCGAGTCCCC-TGGAAC------GGGGCGCC-AGAGAGGG-TGAGAGC
CCCGTACGGTCGGACGCCGAACC-TTTGTAAAGCTCCTTCGACGAGTCGAGTAGTTTGGGAA
TGCTGCTCAAAATGGGAGGTAAATTTCTTCTAAAG-CTAAATACCGGCCAGAGACCGATAGCG
CACAAGTAGAGTGATCGAAAGATGAAAAGCACTTTGAAAAGAGGGTTAAATAGCACGTGAAAT
TGCTGAAAGGGAAGCGCTTGTGACCAGACTTGCGCCCGGTTGATCATCCAGCGTTCTCGCT
GGTGCACTCTGCCGGGCTCAGGCCAGCATCGGCTCCTCCAGGGGGATAAAGGTCGCGGGA
ACGTAGCTCTCTCCGGGGAGTGTTATAGCCCGCGG-CGGAATACCCTTAGGGGGGGTCGAG
GCCCGCG-TTCG-CAAGGATGCTGGCGTAATGGTCACCAGCGACCCGTCTT??????????????
??
??
??
??
??
??
??
??
??
??
??
??
??
??
??
??
??
??
??
????????
>Distoseptispora_obpyriformis_DLUCC_0867
ACCT--------GTC--GTT-------CGTTGCTCCGGCGGG-CGGCTCC-AGGGCGGAGCCACTGTCCG
TTGGGTCGGACGCCCGCCGGGGGA---CAAC-CAAAACTCTCC-TGATCTA-GGGTATCGTCAG
AGTATAA----AAAAA----ACAAGCTAT-CAAAACTTTCAGCAACGGATCTCTTGGTTCTGGCATCG
ATGAAGAACGCAGCGAAATGCGATAAGTAATGTGAATTGCAGAATTCAGTGAATCATCGAATCT
TTGAACGCACATTGCGCCCGCCAGTACTCTGGCGGGCATGCCTGTTCGAGCGTCATTTCAA-
CCCTCGGGCC-CCTCT------TTTGGCCCGGTGTTGGGGTTCTAC--GTCCTGA------------------C
GGACCAGTAGGCCCTCAAAACCAGTGGCGGGTACGCCATGGCTTCCGAGCGCAGTAGTTT-T
CTC--CTCGCTCAGGTGGTCT-GGCGCG--CT-CCGGCCGTTAAAC--CCCGCT-G------TGTAT-----
--ACAG-AAGCCTTCCGAGTCCCC-TGGAAC------GGGGCGCC-AGAGAGGG-TGAGAGCCCCG
TATGGTCGGACGCCGAACC-TATGTAAAGCTCCTTCGACGAGTCGAGTAGTTTGGGAATGCTG
CTCAAAATGGGAGGTAAATTTCTTCTAAAG-CTAAATACCGGCCAGAGACCGATAGCGCACAA
GTAGAGTGATCGAAAGATGAAAAGCACTTTGAAAAGAGGGTTAAATAGCACGTGAAATTGCTG
AAAGGGAAGCGCTTGTGACCAGACTTGCGCCCGGTTGATCATCCAGCGTTCTCGCTGGTGC

ACTCTGCCGGGCTCAGGCCAGCATCGGCTCCTCCAGGGGGATAAAGGTCGCGGAACGTA
GCTCTCTACGGGGAGTGTTATAGCCCGTGG-CGGAATACCCTTAGGGGGGGTCGAGGCACG
CG-TTCG-CAAGGATGCTGGCGTAATGGTCACCAGCGACCCGTCTTTATCACTCATGTGTTATG
TCAGTGTCGGAACACCCGCGGAGCCAATCGTCGATTTCATGGTTGCTAGAAACATGGAGGTC
CTCGAAGAATATGACCCCCTCAGGCTACCGAACGCGACCAAAGTTTTTGTCAACGGTTCGTG
GGTGGGTTGCCACCAAGATCCAAGCCATCTCGTGCGCTTGGTGCAGGAGTTGCGCCGCAAG
GGCGTCATCAATTTCGAGGTCTCGCTGATCCGAGATATACGCGATCGAGAATTCAAGATTTTC
TCGGATGCTGGACGTGTCATGCGACCGCTCTTCGCTGTAGAGCAGTCTGAC---AACTCAGAC
AAG---GGCATTGAAAAAGGTGCTCTGTTGCTCACCAAGGAGCACATTCAGAGGTTGAGAGCA
GATGAGGAATTGTCCAAGGACGATCCAGAATATTTCGGTTGGGACGGCCTACTCAATGCCGG
CGCTATAGAGTACCTTGACGCGGAAGAGGAAGAGACGGCCATGATTTGCATGACACCAGAAG
ATCTAGAGGCTTACCGACTGCAAAAGGCCGGATTCGACGTGCCGGATGACGACGAGGACGC
CG---ATCCCAACAGGCGGCTCAAGACAAAGTTGAATCCCACTACTCACATGTACACACATTGT
GAGATTCATCCCAGCATGCTCTTGGGGATCTGCGCAAGCATTATCCCACTCGTGAGCACGCC
CTGCTTGCCTACACCCTGGGTGTCAAGCAGCTCATCGTCGCCATCAACAAGATGGACACCAC
CAAGTGGTCTGAGGATCGTTACAACGAAATCATCAAGGAGACGTCCAACTTCATCAAGAAGG
TCGGATACAACCCCAAGCAGGTGGCTTTCGTTCCCATCTCTGGCTTCAACGGCGACAACATG
TTGGCCCCCTCCACCAACTGCCCCTGGTACAAGGGCTGGGAGAAGGAGATC---AAGGGCAA
CAAGATCACTGGCAAGACCCTCCTCGAGGCCATCGATGCCATTGAGCAGCCCAAGCGTCCC
ACAGACAAGCCTCTGCGCCTTCCCCTTCAGGATGTCTACAAGATCGGCGGTATCGGCACTGT
GCCCGTCGGCCGTATCGAGACTGGTATCATCAAGCCCGGTATGGTCGTCACATTCGCTCCCT
CCAACGTCACCACTGAAGTCAAGTCCGTCGAGATGCACCACGAGCAGCTGTCCGAGGGTAT
GCCCGGTGACAACGTCGGCTTCAACGTGAAGAACGTCTCCGTCAAGGAAATCCGCCGTGGC
AACGTCGCCGGTGACTCCAAGAACGACCCCCCTGCCGGTGCTGCTTCCTTCACCGCCCAG
GTCATCGTCCTCAACCACCCCGGTCAGGTCGGCGCTGGCTACGCTCCCGTCTTGGATTGCC
ACACTGCCCACATCGCCTGCAAGTTCTCCGAGATCCTTGAGAAGATCGACCGCCGAACGGG
CAAGTCTGTTGAGAACAACCCCAAGTTCATCAAGTCTGGTGACGC
>Distoseptispora_obpyriformis_MFLUCC_17_1694
???
???
???
???
???
???
???
????????GCCTTCCGAGTCCCC-TGGAAC------GGGGCGCC-AGAGAGGG-TGAGAGCCCCGT
ATGGTCGGACGCCGAACC-TATGTAAAGCTCCTTCGACGAGTCGAGTAGTTTGGGAATGCTG
CTCAAAATGGGAGGTAAATTTCTTCTAAAG-CTAAATACCGGCCAGAGACCGATAGCGCACAA
GTAGAGTGATCGAAAGATGAAAAGCACTTTGAAAAGAGGGTTAAATAGCACGTGAAATTGCTG
AAAGGGAAGCGCTTGTGACCAGACTTGCGCCCGGTTGATCATCCAGCGTTCTCGCTGGTGC
ACTCTGCCGGGCTCAGGCCAGCATCGGCTCCTCCAGGGGGATAAAGGTCGCGGAACGTA
GCTCTCTACGGGGAGTGTTATAGCCCGTGG-CGGAATACCCTTAGGGGGGGTCGAGGCACG
CG-TTCG-CAAGGATGCTGGCGTAATGGTCACCAGCGACCCGTCTTTATCACTCATGTGTTATG
TCAGTGTCGGAACACCCGCGGAGCCAATCGTCGATTTCATGGTTGCTAGAAACATGGAGGTC

CTCGAAGAATATGACCCCCTCAGGCTACCGAACGCGACCAAAGTTTTTGTCAACGGTTCGTG
GGTGGGTTGCCACCAAGATCCAAGCCATCTCGTGCGCTTGGTGCAGGAGTTGCGCCGCAAG
GGCGTCATCAATTTCGAGGTCTCGCTGATCCGAGATATACGCGATCGAGAATTCAAGATTTTC
TCGGATGCTGGACGTGTCATGCGACCGCTCTTCGCTGTAGAGCAGTCTGAC---AACTCAGAC
AAG---GGCATTGAAAAAGGTGCTCTGTTGCTCACCAAGGAGCACATTCAGAGGTTGAGAGCA
GATGAGGAATTGTCCAAGGACGATCCAGAATATTTCGGTTGGGACGGCCTACTCAATGCCGG
CGCTATAGAGTACCTTGACGCGGAAGAGGAAGAGACGGCCATGATTTGCATGACACCAGAAG
ATCTAGAGGCTTACCGACTGCAAAAGGCCGGATTCAACGTCCCGGATGTCGACGAGGACGC
CG---ATCCCAACAGGCGGCTCAAGACAAAGTTGAATCCCACTACTCACATGTACACACATTGT
GAGATTCATCCCAGCATGCTCTTGGGGATCTGCGCAAGCATTATCCCACTCGTGAGCACGCC
CTGCTTGCCTACACCCTGGGTGTCAAGCAGCTCATCGTCGCCATCAACAAGATGGACACCAC
CAAGTGGTCTGAGGATCGTTACAACGAAATCATCAAGGAGACGTCCAACTTCATCAAGAAGG
TCGGATACAACCCCAAGCAGGTGGCTTTCGTTCCCATCTCTGGCTTCAACGGCGACAACATG
TTGGCCCCCTCCACCAACTGCCCCTGGTACAAGGGCTGGGAGAAGGAGATC---AAGGGCAA
CAAGATCACTGGCAAGACCCTCCTCGAGGCCATCGATGCCATTGAGCAGCCCAAGCGTCCC
ACAGACAAGCCTCTGCGCCTTCCCCTTCAGGATGTCTACAAGATCGGCGGTATCGGCACTGT
GCCCGTCGGCCGTATCGAGACTGGTATCATCAAGCCCGGTATGGTCGTCACATTCGCTCCCT
CCAACGTCACCACTGAAGTCAAGTCCGTCGAGATGCACCACGAGCAGCTGTCCGAGGGTAT
GCCCGGTGACAACGTCGGCTTCAACGTGAAGAACGTCTCCGTCAAGGAAATCCGCCGTGGC
AACGTCGCCGGTGACTCCAAGAACGACCCCCCTGCCGGTGCTGCTTCCTTCACCGCCCAG
GTCATCGTCCTCAACCACCCCGGTCAGGTCGGCGCTGGCTACGCTCCCGTCTTGGATTGCC
ACACTGCCCACATCGCCTGCAAGTTCTCCGAGATCCTTGAGAAGATCGACCGCCGAACGGG
CAAGTCTGTTGAGAACAACCCCAAGTTCATCAAGTCTGGTGACGC
>Distoseptispora_pachyconidia_KUMCC_21_10724
ACCT--------CT-ATACT-------CGTTGCTTTGGCGGG-CGGCTCC-AGGGCGGAGCCACTGCTCGT
TGGGTCGGGCGCCCGCCGAAGGA---CAAC-CAAAACTCGTTGGAACCTTT-GTGGATCTCAGA
GTAAAAA-----TAT-----CAAGCTAT-TAAAACTTTCAGCAACGGATCTCTTGGTTCTGGCATCGATG
AAGAACGCAGCGAAATGCGATAAGTAATGTGAATTGCAGAATTCAGTGAATCATCGAATCTTT
GAACGCACATTGCGCCCGCCAGCACTCTGGCGGGCATGCCTGTTCGAGCGTCATTTCAA-CC
CTCGGGCCC-TCGTT-------TGGCCTGGTGTTGGGGCGCTACGGGTCTCAAAT------------------GG
AC-CTGTAGGCCCTCAAAATCAGTGGCGGGCTCGCCAGGACT-CCGAGCGTAGTAGTTC-TCC
T--CTCGCTCAGGCGGTCT-GGCGTG--CT-TCGGCCGTTAAA---TACCCAGC------GGTAAT-CGC
GCACAC-AAGCCTTCCGAGTCCCC-TGGAAC------GGGGCGCC-AGAGAGGG-TGAGAGCCCC
GTATGGTCGGACGCCGAACC-TCTGTAAAGCTCCTTCGACGAGTCGAGTAGTTTGGGAATGC
TGCTCAAAATGGGAGGTAAATTTCTTCTAAAG-CTAAATACCGGCCAGAGACCGATAGCGCAC
AAGTAGAGTGATCGAAAGATGAAAAGCACTTTGAAAAGAGGGTCAAATAGCACGTGAAATTGC
TGAAAGGGAAGCGCTTGTGACCAGACTTGCGCCCGGTTGATCATCCAGCGTTCTCGCTGGT
GCACTCTGCCGGGCTCAGGCCAGCATCGGCTCTCCCAGGGGGATAAAGGTCGCGGGAACG
TAGCTCTCTCCGGGGAGTGTTATAGCCCGTGG-CGGAATGCCCTTAGGGGGGGCCGAGGCC
CGCGTTC-G-CAAGGATGCTGGCGTAATGGTCACCAGCGACCCGTCTTTATCGCTCATGTGTT
ATGTGAGCGTGGGAACGCCCGCAGAGCCAATCGTCGAGTTCATGATTGCTAGGAACATGGAA
GTACTCGAGGAATATGAGCCCTTGAGGTACCCTAATGCGACCAAAATCTTCGTCAATGGCACA
TGGGTTGGTGTTCACCAAGACCCGAAGCATCTTGTGAGCTTGGTTCAGGACTTACGCCGCA
GAGGCGTCATCAACTTTGAGGTCTCTCTCATTCGGGATATCCGCGATCGAGAGTTCAAGATTT

TCTCGGATGCTGGTCGTGTTATGCGTCCTCTGTTCGCTGTGGAGCAGTCGGAC---AACTCGA
CAAAG---GGTCTTGAAAAGGGATCTTTATTGCTCACAAAGGAGCACATTCAAAAATTGAGAGC
TGATGAGGAGTTATCCAAGGATGACCCAGATTATTTCGGCTGGGACGGCCTTCTCAGAGCGG
GCGCTATCGAATATCTTGACGCCGAAGAGGAAGAGACAGCCATGATTTGCATGACTCCAGAA
GATCTAGAGGCTTATCGGCTGCAAAAAGCTGGATTAGACGTGCCGGAAGACGATGACGAGG
CCG---ACCCCAATAGGCGTCTGAAGACGAAACTGAACCCCACGACTCACATGTATACACATTG
TGAGATCCATCCGAGCATGCTCTTGGGGATCTGTGCGAGCATTATCCCACTCGTGAGCACGC
TCTGCTCGCCTACACGCTGGGTGTGAAGCAGCTCATTGTCGCCATCAACAAGATGGACACCA
CCAAGTGGTCTGAGAGCCGTTACAACGAAATTATCAAGGAGACGTCCAACTTCATCAAGAAG
GTTGGCTACAACCCCAAGCAGGTCGCCTTCGTTCCCATCTCCGGCTTCAACGGCGACAACAT
GCTTGCCCCCTCCTCCAACTGCCCCTGGTACAAGGGCTGGGAGAAGGAGATC---AAGGGTG
GCAAGGCCACTGGCAAGACCCTCCTCGAGGCTATCGATGCCATTGAGCCTCCCAAGCGTCC
CACAGACAAGCCTCTCCGCCTTCCCCTTCAGGATGTCTACAAGATCGGTGGTATCGGAACTG
TGCCCGTCGGCCGTATCGAGACTGGTATCCTCAAGCCCGGTATGGTCGTCACGTTCGCTCCC
TCCAACGTCACCACTGAAGTCAAGTCCGTCGAGATGCACCACGAGCAGCTGTCGGAGGGTG
TCCCCGGCGACAACGTTGGTTTCAACGTGAAGAACGTCTCCGTCAAGGAAATCCGCCGAGG
CAACGTCGCTGGTGACTCCAAGAACGATCCTCCTGCTGGTGCCGCTTCCTTCACCGCTCAG
GTCATCGTCCTCAACCACCCCGGTCAGGTCGGTGCTGGTTACGCTCCCGTCTTGGATTGCC
ACACTGCCCACATTGCTTGCAAGTTCTCTGAGATCCTTGAGAAGATTGACCGCCGAACGGGC
AAGTCTGTTGAGAACAACCCCAAGTTCATCAAGTCTGGTGACGC
>Distoseptispora_palmarum_MFLUCC_18_1446
ACCT--------TC---TCTC------ATGCGCTCCGGTGGG-CGGTTCC-AGGGCGGAGCCACTGCCCG
TTGGCTCGGGTGTCCGCCGGAGCGAACTCTT-TAAAACTCTGCTGTTA--CAGTGAGG-ATCAG
AGAGAT--------AA----GCAAACTAT-AAAAACTTTCAGCAACGGATCTCTTGGCTCTGGCATCGAT
GAAGAACGCAGCGAAATGCGATAAATAATGTGAATTGCAGACTTCTG-GAATCATCGAGTTTTT
GAACGCACATTGCGCCCGTCAGTATTCTGGCGGGCATGCCTGTCCGAGCGTCATTTCAAACC
CTCGGGCCTCCTGTA-------GG-CCTGGTGTTGGGGCGCTGTGGGACTCCCCGTA------------CG
GCGGAGCCCGCAGGCCCTCAAATGCAGTGGCGGAGCTGTTTGTGTT-CCTGGTGCAGTAGA
TT-ATTA---TCACCC-GGGCCCCA-AGCGGA--CT-TC--CTGCCAACAG-AC-CTATA------TCTCACA
A----------GGCCTTCTGAGTCCCC-TGGAAC------GGGGCGCC-AGAGAGGG-TGAGAGCCCCGT
ACGGTCGGACACCGAGCC-TCTGTAAAGCTCCTTCGACGAGTCGAGTAGTTTGGGAATGCTG
CTCTAAATGGGAGGTAAATTTCTCCTAAAG-CTAAATATAGGCCAGAGACCGATAGCGCACAAG
TAGAGTGATCGAAAGATGAAAAGCACTTTGAAAAGAGGGTTAAATAGCACGTGAAATTGCTGA
AAGGGAAGCGCTTGTGACCAGACTTGCGCCCGGTGGATCAGCTGGCGTTCTCGCCAGTGC
ACTCCGCCGGGTTCAGGCCAGCATCGGTTCCGACGGGGGGACAAAGGCCTCTGGAACGTA
GCTCTCTTCGGGGAGTGTTATAGCCAGTGG-TGCCATACCCCCAGGCGGGACCGAGGATCG
CGCTCTG-CAAGGATGCTGGCGTAATGGTCACCAGCGACCCGTCTTTATCTCTGATGTGTTAT
GTCAGTGTGGGAACAACGGCCGACCCTATTCGAGAATTCATGGTTGCCCGGAATATGGAAAT
TCTCGAGGAGTATGAGCCACTTCGGTATCCGAACGCCACAAAGATCTTCATCAATGGCTCATG
GGTTGGTGTTCACCAAGATCCAAAGAGCCTCGTGAATGCGGTGAGAGCAATGCGGCAAAAC
GGCATTATTCAATTCGAGGTCACCCTCGTCAGAGACATCCGCGATCGAGAGTTCAAGATATTT
TCTGACGCGGGTCGCGTTATGCGCCCCCTTTTCGCTGTTGAACAAGACGATCCGAGCGAAG
AAAACGGTGGTCGGGAGAAGGGTAGTTTGCTACTCACAAAAGAACACATCCAGCGACTGAG
AGATGACGAGCGCTTGGATCCCAGTGATCCGAACTACTTTGGCTGGGATGGATTGACTAGAG

AAGGCGCAATTGAGTATCTGGACGCCGAAGAGGAGGAAACCGCAATGATTTGCATGACCCCT
GAGGATCTGGAGCAATATCGTCTTCAGAAGCAGGGTATCGCACCCGACGAGGACGATGGTG
ACGA------CCTCAATAAGCGCCTCAAGACGAGGATGAATCCTACCACACATATGTACACCCACT
GCGAGATCCATCCGAGTATGCTCCTGGGGATATGCGCCAGCATCATCCCACCCGTGAGCACG
CCCTGCTTGCCTACACCCTGGGTGTGAAGCAGCTCATCGTCGCCATCAACAAGATGGACACC
ACGAAGTGGTCTGAGGATCGTTTCAACGAAATCATCAAGGAGACCTCCAACTTCATCAAGAA
GGTCGGCTACAACCCCAAGACTGTTGCCTTCGTCCCCATCTCGGGCTTCAACGGTGACAACA
TGCTTGCGCCTTCAAGCAACTGCCCCTGGTACAAGGGATGGGAGAAGGAAG---TCAAGGGT
GGCAAGGTCACTGGCAAGACCCTCCTCGAGGCCATTGACGCCATTGAGCCCCCCAAGCGTC
CCACCGACAAGCCTCTCCGTCTGCCCCTTCAGGATGTGTACAAGATTGGTGGTATCGGAACA
GTCCCTGTCGGCCGTATTGAGACTGGTGTCCTCAAGCCCGGTATGGTCGTCACGTTCGCTC
CCTCCAACGTCACCACTGAAGTCAAGTCCGTCGAGATGCACCACGAACAGCTCACTGAGGG
TGTTCCCGGCGACAACGTTGGCTTCAACGTGAAGAACGTCTCCGTCAAGGAAATCCGCCGT
GGCAACGTCGCTGGTGACTCCAAGAACGACCCTCCTATGGGCTGCGCTTCCTTCACTGCTC
AGGTCATCGTGTTGAACCACCCTGGCCAGGTTGGTGCTGGATACGCCCCTGTGCTCGACTG
CCACACCGCCCACATCGCCTGCAAGTTCTCTGAAATTCTTGAGAAGATCGATCGTCGAACAG
GCAAGTCAGTTGAGAACAACCCCAAGTTCATCAAGTCGGGTGACGC
>Distoseptispora_phangngaensis_MFLUCC_16_0857
ACCT--------TT-TTGTT-------CGTTGCTTTGGCGGG-CGGCTCC-AGGGCGGAGCCACTGCCCG
TTGGGTCGGGCGCCCGCCGAAGGA---CAAC-CGAAACTCGCT-AAGTTTTT-ATGGATATCAGA
GTAAAA------TAT-----CAAGCTAT-CAAAACTTTCAGCAACGGATCTCTTGGTTCTGGCATCGATG
AAGAACGCAGCGAAATGCGATAAGTAATGTGAATTGCAGAATTCAGTGAATCATCGAATCTTT
GAACGCACATTGCGCCCGCCAGCACTCTGGCGGGCATGCCTGTTCGAGCGTCATTTCAA-CC
CTCGGGCCC-TCGTT-------TGGTCCGGTGTTGGGGCGCTACGGGTCCG--TC------------------GG
AC-CCGTAGGCCCTCAAAAACAGTGGCGGGCTCGCCAGGACT-CCGAGCGCAGTAGTTT-TC-
T--CTCGCTCAGGCGGCCT-GGCGTG--CT-TCGGCCGTTAAA---GACCCAAC------TGTAAT-----C
ACAG-AAGCCTTCCGAGTCCCC-TGGAAC------GGGGCGCC-AGAGAGGG-TGAGAGCCCCGT
ATGGTTGGACGCCGAACC-TCTGTAAAGCTCCTTCGACGAGTCGAGTAGTTTGGGAATGCTG
CTCAAAATGGGAGGTAAATTTCTTCTAAAG-CTAAATACCGGCCAGAGACCGATAGCGCACAA
GTAGAGTGATCGAAAGATGAAAAGCACTTTGAAAAGAGGGTCAAATAGCACGTGAAATTGCT
GAAAGGGAAGTGCTTGTGACCAGACTTGCGCCCGGTTGATCATCCAGCGTTCTCGCTGGTG
CACTCTGCCGGGCTCAGGCCAGCATCGGCTCTCCCAGGGGGATAAAGGTCGCGGAACGT
AGCTCTCTCCGGGGAGTGTTATAGCCCGTGG-CGGAATGCCCTTAGGGGGGCCGAGGCCC
GCGTTC-G-CAAGGATGCTGGCGTAATGGTCACCAGCGACCCGTCTT???????????????????
??
??
??
??
??
??
??
??
??ACTCGTGAGCACGCTCTGC
TTGCCTACACCCTGGGTGTGAAGCAGCTCATCGTCGCCATCAACAAGATGGACACCACCAAG

TGGTCTGAGAGCCGTTACAACGAAATTATCAAGGAGACGTCCAACTTCATCAAGAAGGTCGG
CTACAACCCCAAGCAGGTCGCCTTCGTTCCCATCTCTGGCTTCAACGGTGACAACATGCTTA
CCCCCTCCTCCAACTGCCCCTGGTACAAGGGCTGGGAGAAGGAGGTC---AAGGGCGGCAAG
GCCAGTGGCAAGACCCTTCTCGAGGCCATCGATGCCATTGAGCCTCCCAAGCGTCCTACAG
ACAAGCCTCTGCGCCTCCCCCTCCAGGATGTCTACAAGATCGGCGGTATTGGAACTGTGCCC
GTCGGCCGTATCGAGACTGGTGTCCTCAAGCCCGGTATGGTCGTCACGTTCGCTCCCTCCA
ACGTCACCACTGAAGTCAAGTCCGTCGAGATGCACCACGAACAGCTGCAGGAGGGTGTCCC
CGGCGACAACGTCGGCTTCAACGTGAAGAACGTCTCCGTCAAGGAAATCCGCCGAGGCAAC
GTTGCTGGTGACTCCAAGAACGATCCTCCTGCTGGTGCCGCTTCCTTCACCGCCCAGGTCAT
CGTCCTCAACCACCCCGGTCAGGTCGGCGCTGGCTACGCTCCCGTCTTGGACTGCCACACT
GCCCACATCGCTTGCAAGTTCTCCGAGATTCTTGAGAAGATTGACCGCCGAACGGGCAAGTC
TGTCGAGAACAACCCCAAGTTCATCAAGTCTGGTGACGC
>Distoseptispora_rayongensis_MFLUCC_18_0415
ACCT--------ATC--GTT-------CGTTGCTCCGGCGGG-CGGCTCC-AGGGCGGAGCCACTGCCAG
TTGGGTCGGGCGCCCGCCGGAGGA---CAAC-CAAAACTCGCC-TAGTCTC-GGGTAACGTCAG
AGTAAAA----AAAAAAATTAAAAGCTAT-CAAAACTTTCAGCAACGGATCTCTTGGTTCTGGCATC
GATGAAGAACGCAGCGAAATGCGATAAGTAATGTGAATTGCAGAATTCAGTGAATCATCGAAT
CTTTGAACGCACATTGCGCCCGCCAGCACTCTGGCGGGCATGCCTGTTCGAGCGTCATTTC
AA-CCCTCGGGCC-CCCCCT----GTGTGGCCCGGTGTTGGGGCGCTACCGTCCCCCTT--C------
------CGGAGGGTCAGGTAGGCCCTGAAAACCAGTGGCGGGCTCGCCATGGCCTCCGAGCGC
AGTAGTTC-TCTC--CTCGCTCAGGCGGTCT-GGCGCG--CC-TCGGCCGTTAAA---CACACG-C--
----TGTATCAC----GCAG-AAGCCTTCCGAGTCCCC-TGGAAC------GGGGCGCC-AGAGAGGG-T
GAGAGCCCCGTATGGTCGGACGCCGAACC-TTTGTAAAGCTCCTTCGACGAGTCGAGTAGTT
TGGGAATGCTGCTCAAAATGGGAGGTAAATTTCTTCTAAAG-CTAAATACCGGCCAGAGACCG
ATAGCGCACAAGTAGAGTGATCGAAAGATGAAAAGCACTTTGAAAAGAGGGTTAAATAGCACG
TGAAATTGCTGAAAGGGAAGCGCTTGTGACCAGACTTGCGCCCGGTTGATCATCCAGCGTTC
TCGCTGGTGCACTCTGCCGGGCTCAGGCCAGCATCGGCTCCCCCAGGGGGATAAAGGTCG
CGGGAACGTAGCTCTCTCCGGGGAGTGTTATAGCCCGTGG-CGGAATACCCTTAGGGGGGG
TCGAGGCCCGCGTTC-G-CAAGGATGCTGGCGTAATGGTCACCAGCGACCCGTCTTTCTCGC
TCATGTGTTATGTCAGCGTTGGCACACCTGCAGAGCCAATTGTCGAGTTCATGATTGCTAGGA
ACATGGAGGTACTCGAGGAATATGAGCCCTTGAGGTACCCGAATGCAACCAAAATCTTCGTTA
ATGGTACGTGGGTGGGTGTTCACCAAGATCCAAAGCATCTTGTGGGCTTGGTCCAGGACTTA
CGCCGCAGGGGCGTCATCAACTTCGAGGTCTCACTCATCCGAGACATACGCGATCGAGAATT
CAAGATTTTCTCGGATGCTGGACGAGTCATGCGACCGCTTTTCGCTGTAGAGCAGTCAGAC--
-AACTCAAAAAAC---GGCCTTGAAAAGGGTTCTTTGTTGCTCACAAAGGACCATATTCAGAAGT
TGAGGGCAGACGAAGACATATCGAAAGACGACCCAGACTACTTCGGCTGGGACGGCCTACT
CAAAGCCGGCGCCATCGAGTATCTTGACGCGGAAGAGGAAGAGACAGCGATGATTTGCATG
ACGCCGGAAGACCTGGAGGCCTACCGACTCCAAAAAGCCGGATTAGACGTGCCCGAAGATG
AAGATGAAGCCG---ACCCCAACAGGCGTCTGAAGACCAAATTGAACCCAACCACTCATATGTA
CACACATTGTGAGATCCATCCGAGCATGCTCTTGGGGATCTGCGCGAGCATCATCCCACTCG
TGAGCACGCTCTGCTTGCCTACACCCTGGGTGTGAAGCAGCTCATCGTCGCCATCAACAAGA
TGGATACCACCAAGTGGTCTGAGGACCGTTACAACGAAATCATCAAGGAGACGTCCAACTTC
ATCAAGAAGGTTGGCTACAACCCCAAGCAGGTCGCCTTCGTTCCCATCTCCGGCTTCAACGG
CGATAACATGCTTACCCCCTCCTCCAACTGCCCCTGGTACAAGGGCTGGGAGAAGGAGATC--

-AAGGGTGGCAAGGTCACTGGCAAGACGCTTCTCGAGGCCATCGATGCCATTGAGCCTCCCA
AGCGTCCCACAGACAAGCCTCTGCGTCTTCCCCTCCAGGATGTCTACAAGATCGGTGGTATC
GGAACCGTACCTGTCGGCCGTATCGAGACTGGTGTCCTCAAGCCCGGTATGGTCGTCACGT
TCGCTCCCTCCAACGTCACCACTGAAGTCAAGTCTGTCGAGATGCACCACGAGCAGCTTCAA
GAGGGCGTTCCTGGCGACAACGTCGGCTTCAACGTGAAGAACGTCTCTGTCAAGGAAATCC
GCCGTGGCAACGTCGCTGGTGACTCCAAGAACGATCCTCCTGCTGGTGCTGCTTCCTTCAC
CGCCCAGGTCATCGTCCTCAACCACCCCGGTCAGGTTGGTGCCGGCTACGCTCCTGTTCTG
GACTGCCACACTGCCCACATCGCCTGCAAGTTCGCCGAGATCCTCGAGAAGATCGACCGCC
GAACGGGCAAGTCCGTCGAGAACAACCCCAAGTTCATCAAGTCTGGTGACGC
>Distoseptispora_rayongensis_MFLUCC_18_0417
ACCT--------ATC--GTT-------CGTTGCTCCGGCGGG-CGGCTCC-AGGGCGGAGCCACTGCCAG
TTGGGTCGGGCGCCCGCCGGAGGA---CAAC-CAAAACTCGCC-TAGTCTC-GGGTAACGTCAG
AGTAAAA----AAAA--ATTAAAAGCTAT-CAAAACTTTCAGCAACGGATCTCTTGGTTCTGGCATC
GATGAAGAACGCAGCGAAATGCGATAAGTAATGTGAATTGCAGAATTCAGTGAATCATCGAAT
CTTTGAACGCACATTGCGCCCGCCAGCACTCTGGCGGGCATGCCTGTTCGAGCGTCATTTC
AA-CCCTCGGGCC-CCCCCT----GTGTGGCCCGGTGTTGGGGCGCTACCGTCCCCCTT--C------
------CGGAGGGTCAGGTAGGCCCTGAAAACCAGTGGCGGGCTCGCCATGGCCTCCGAGCGC
AGTAGTTC-TCTC--CTCGCTCAGGCGGTCT-GGCGCG--CC-TCGGCCGTTAAA---CACACG-C--
----TGTATCAC----GCAG-AAGCCTTCCGAGTCCCC-TGGAAC------GGGGCGCC-AGAGAGGG-T
GAGAGCCCCGTATGGTCGGACGCCGAACC-TTTGTAAAGCTCCTTCGACGAGTCGAGTAGTT
TGGGAATGCTGCTCAAAATGGGAGGTAAATTTCTTCTAAAG-CTAAATACCGGCCAGAGACCG
ATAGCGCACAAGTAGAGTGATCGAAAGATGAAAAGCACTTTGAAAAGAGGGTTAAATAGCACG
TGAAATTGCTGAAAGGGAAGCGCTTGTGACCAGACTTGCGCCCGGTTGATCATCCAGCGTTC
TCGCTGGTGCACTCTGCCGGGCTCAGGCCAGCATCGGCTCCCCCAGGGGGATAAAGGTCG
CGGGAACGTAGCTCTCTCCGGGGAGTGTTATAGCCCGTGG-CGGAATACCCTTAGGGGGGG
TCGAGGCCCGCGTTC-G-CAAGGATGCTGGCGTAATGGTCACCAGCGACCCGTCTTTCTCGC
TCATGTGTTATGTCAGCGTTGGCACACCTGCAGAGCCAATTGTCGAGTTCATGATTGCTAGGA
ACATGGAGGTACTCGAGGAATATGAGCCCTTGAGGTACCCGAATGCAACCAAAATCTTCGTTA
ATGGTACGTGGGTGGGTGTTCACCAAGATCCAAAGCATCTTGTGGGCTTGGTCCAGGACTTA
CGCCGCAGGGGCGTCATCAACTTCGAGGTCTCACTCATCCGAGACATACGCGATCGAGAATT
CAAGATTTTCTCGGATGCTGGACGAGTCATGCGACCGCTTTTCGCTGTAGAGCAGTCAGAC--
-AACTCAAAAAAC---GGCCTTGAAAAGGGTTCTTTGTTGCTCACAAAGGACCATATTCAGAAGT
TGAGGGCAGACGAAGACATATCGAAAGACGACCCAGACTACTTCGGCTGGGACGGCCTACT
CAAAGCCGGCGCCATCGAGTATCTTGACGCGGAAGAGGAAGAGACAGCGATGATTTGCATG
ACGCCGGAAGACCTGGAGGCCTACCGACTCCAAAAAGCCGGATTAGACGTGCCCGAAGATG
AAGATGAAGCCG---ACCCCAACAGGCGTCTGAAGACCAAATTGAACCCAACCACTCATATGTA
CACACATTGTGAGATCCATCCGAGCATGCTCTTGGGGATCTGCGCGAGCATCATCCCACTCG
TGAGCACGCTCTGCTTGCCTACACCCTGGGTGTGAAGCAGCTCATCGTCGCCATCAACAAGA
TGGATACCACTAAGTGGTCTGAGGACCGTTACAACGAAATCATCAAGGAGACGTCCAACTTCA
TCAAGAAGGTTGGCTACAACCCCAAGCAGGTCGCCTTCGTTCCCATCTCCGGCTTCAACGG
CGATAACATGCTTACCCCCTCCTCCAACTGCCCCTGGTACAAGGGCTGGGAGAAGGAGATC--
-AAGGGTGGCAAGGTCACTGGCAAGACGCTTCTCGAGGCCATCGATGCCATTGAGCCTCCCA
AGCGTCCCACAGACAAGCCTCTGCGTCTTCCCCTCCAGGATGTCTACAAGATCGGTGGTATC
GGAACCGTACCTGTCGGCCGTATCGAGACTGGTGTCCTCAAGCCCGGTATGGTCGTCACGT

TCGCTCCCTCCAACGTCACCACTGAAGTCAAGTCTGTCGAGATGCACCACGAGCAGCTTCAA
GAGGGCGTTCCTGGCGACAACGTCGGCTTCAACGTGAAGAACGTCTCTGTCAAGGAAATCC
GCCGTGGCAACGTCGCTGGTGACTCCAAGAACGATCCTCCTGCTGGTGCTGCTTCCTTCAC
CGCCCAGGTCATCGTCCTCAACCACCCCGGTCAGGTTGGTGCCGGCTACGCTCCTGTTCTG
GACTGCCACACTGCCCACATCGCCTGCAAGTTCGCCGAGATCCTCGAGAAGATCGACCGCC
GAACGGGCAAGTCCGTCGAGAACAACCCCAAGTTCATCAAGTCTGGTGACGC
>Distoseptispora_rostrata_DLUCC_0885
ACCT--------GTA--TTT------CGTTGCTCCGGCGGG-CGGCTCC-AGGGCGGAGCCACTGTCCGT
TAGGTCGGACGCCCGCCGGGGGA---CAAC-CAAAACTCTTC-TGATCTC-GGGTATCGTCAGA
GTACAC----AAAA------CAAGCTAT-CAAAACTTTCAGCAACGGATCTCTTGGTTCTGGCATCGAT
GAAGAACGCAGCGAAATGCGATAAGTAATGTGAATTGCAGAATTCAGTGAATCATCGAATCTT
TGAACGCACATTGCGCCCGCCAGTACTCTGGCGGGCATGCCTGTTCGAGCGTCATTTCAA-C
CCTCGGGCC-CCTGT------TT-GGCCCGGTGTTGGGGTGCTAC--GTCCTGA--------------------CG
GACC-GTAGGCCCTCAAAATCAGTGGCGGGCTCGCCATGGCCTCCGAGCGCAGTAGTTG-TC
TC--CTCGCTCAGGTGGTCT-GGCGCG--CT-CCGGCCGTTAAAA--CACCCG-C------TGTAA-TT---
-GCAG-AAGCCTTCCGAGTCCCC-TGGAAC------GGGGCGCC-AGAGAGGG-TGAGAGCCCCG
TATGGTCGGACGCCGAGCC-TCTGTAAAGCTCCTTCGACGAGTCGAGTAGTTTGGGAATGCT
GCTCAAAATGGGAGGTAAATTTCTTCTAAAG-CTAAATACCGGCCAGAGACCGATAGCGCACA
AGTAGAGTGATCGAAAGATGAAAAGCACTTTGAAAAGAGGGTTAAATAGCACGTGAAATTGCT
GAAAGGGAAGCGCTTGTGACCAGACTTGCGCCCGGTTGATCATCCAGCGTTCTCGCTGGTG
CACTCTGCCGGGCACAGGCCAGCATCGGCTCTCCCAGGGGGATAAAGGTCGCGGGAACGT
AGCTCTCTCCGGGGAGTGTTATAGCCCGCGG-CGGAATACCCTTAGGGGGGGTCGAGGCCC
GCG-TTCG-CAAGGATGCTGGCGTAATGGTCACCAGCGACCCGTCTT??????????????????
??
??
??
??
??
??
??
??
??ACTCGTGAGCACGCCCTGC
TTGCCTACACCCTTGGTGTCAAGCAGCTCATCGTCGCCATCAACAAGATGGACACCACCAAG
TGGTCTGAGGATCGTTACAACGAAATCATCAAGGAGACGTCCAACTTCATCAAGAAGGTTGG
ATACAACCCCAAGCAGGTGGCTTTCGTCCCCATCTCCGGCTTCAACGGTGACAACATGTTGA
CCCCCTCCTCCAACTGCCCCTGGTACAAGGGTTGGGAGAAGGAGATC---AAGGGTAGCAAGA
TCACTGGCAAGACGCTGCTCGAGGCCATCGATGCTATTGAGCAACCCAAGCGTCCCACAGA
CAAGCCTCTGCGCCTGCCCCTTCAGGATGTCTACAAGATCGGTGGTATCGGCACTGTACCGG
TCGGCCGTATCGAGACTGGTGTCCTCAAGCCCGGTATGGTCGTTACATTCGCTCCCTCCAAC
GTCACCACTGAAGTCAAGTCCGTCGAGATGCACCACGAGCAGCTTGCCGAGGGTCTCCCCG
GTGACAACGTCGGCTTCAACGTGAAGAACGTCTCTGTCAAGGAAATCCGCCGTGGCAACGT
CGCCGGTGACTCCAAGAACGATCCTCCTGCCGGTGCTGCTTCCTTCACCGCCCAGGTCATC
GTCCTCAACCACCCTGGTCAGGTCGGCGCTGGCTACGCTCCCGTCTTGGATTGCCACACTG
CCCACATCGCCTGCAAGTTCTCTGAGATCCTCGAGAAGATTGACCGCCGAACGGGCAAGTC

TGTCGAGAACAACCCCAAGTTCATCAAGTCTGGTGACGC
>Distoseptispora_rostrata_MFLUCC_16_0969
ACCT--------GTA--TTT-------CGTTGCTCCGGCGGG-CGGCTCC-AGGGCGGAGCCACTGTCCGT
TAGGTCGGACGCCCGCCGGGGGA---CAAC-CAAAACTCTTC-TGATCTC-GGGTATCGTCAGA
GTACAC----AAAA------CAAGCTAT-CAAAACTTTCAGCAACGGATCTCTTGGTTCTGGCATCGAT
GAAGAACGCAGCGAAATGCGATAAGTAATGTGAATTGCAGAATTCAGTGAATCATCGAATCTT
TGAACGCACATTGCGCCCGCCAGTACTCTGGCGGGCATGCCTGTTCGAGCGTCATTTCAA-C
CCTCGGGCC-CCTGT------TT-GGCCCGGTGTTGGGGTGCTAC--GTCCTGA--------------------CG
GACC-GTAGGCCCTCAAAATCAGTGGCGGGCTCGCCATGGCCTCCGAGCGCAGTAGTTG-TC
TC--CTCGCTCAGGTGGTCT-GGCGCG--CT-CCGGCCGTTAAAA--CACCCG-C------TGTAA-TT---
-ACAG-AAGCCTTCCGAGTCCCC-TGGAAC------GGGGCGCC-AGAGAGGG-TGAGAGCCCCGT
ATGGTCGGACGCCGAGCC-TCTGTAAAGCTCCTTCGACGAGTCGAGTAGTTTGGGAATGCTG
CTCAAAATGGGAGGTAAATTTCTTCTAAAG-CTAAATACCGGCCAGAGACCGATAGCGCACAA
GTAGAGTGATCGAAAGATGAAAAGCACTTTGAAAAGAGGGTTAAATAGCACGTGAAATTGCTG
AAAGGGAAGCGCTTGTGACCAGACTTGCGCCCGGTTGATCATCCAGCGTTCTCGCTGGTGC
ACTCTGCCGGGCACAGGCCAGCATCGGCTCTCCCAGGGGGATAAAGGTCGCGGGAACGTA
GCTCTCTCCGGGGAGTGTTATAGCCCGCGG-CGGAATACCCTTAGGGGGGGTCGAGGCCCG
CG-TTCG-CAAGGATGCTGGCGTAATGGTCACCAGCGACCCGTCTTTATCACTCATGTGTTATG
TCAGCGTCGGAACACCTGCGGAGCCAATCGTCGATTTCATGATTGCTAGAAACATGGAGGTC
CTCGAAGAATACGACCCTCTAAGGCTGCCAAATGCGACCAAAGTCTTCGTCAACGGCTCGTG
GGTGGGTTGCCACCAAGATCCAAGCCATCTCGTGCGCTTGGTGCAGGAGCTGCGCCGCAA
GGGCGTCATCAATTTCGAGGTCTCGCTGATTCGAGATATACGCGATCGAGAATTCAAGATTTT
CTCAGATGCCGGACGTGTCATGCGACCGCTTTTCGCTGTAGAGCAGTCAGAC---AACTCAGA
AAAG---GGCATTGAGAAAGGCGCCTTGTTTCTTACCAAGGATCACATTCAGAGACTGAGAGCA
GACGAGGAATTGCCAAAGGACGATGCAGAATATTTCGGTTGGGACGGCCTGCTCAATGCCG
GTGCAATAGAGTATCTTGACGCAGAAGAGGAAGAGACGGCCATGATTTGCATGACACCGGAA
GATTTAGAGGCTTATCGACTTCAAAAGGCTGGATTCGACGTGCCGGATGACGAC----------------
--ACTCGTGAGCACG
CTCTGCTTGCCTACACCCTTGGTGTCAAGCAGCTCATCGTCGCCATCAACAAGATGGACACC
ACCAAGTGGTCTGAGGATCGTTACAACGAAATCATCAAGGAGACGTCCAACTTCATCAAGAA
GGTCGGATACAACCCCAAGCAGGTGGCTTTCGTTCCCATCTCCGGCTTCAACGGTGACAACA
TGTTGACCCCCTCCTCCAACTGCCCCTGGTACAAGGGCTGGGAGAAGGAGATC---AAGGGC
AGCAAGATCACTGGCAAGACGCTGCTCGAGGCCATCGATGCTATTGAGCAGCCCAAGCGTC
CCACAGACAAGCCTCTGCGCCTGCCCCTTCAGGATGTCTACAAGATCGGTGGTATCGGCACT
GTACCGGTCGGCCGTATCGAGACTGGTGTCCTCAAGCCGGGTATGGTCGTTACATTCGCTCC
CTCCAACGTCACCACTGAAGTCAAGTCCGTCGAGATGCACCACGAGCAGCTTGCCGAGGGT
CTCCCCGGTGACAACGTCGGCTTCAACGTGAAGAACGTCTCCGTCAAGGAAATCCGCCGTG
GCAACGTCGCCGGTGACTCCAAGAACGATCCTCCTGCCGGTGCTGCTTCCTTCACCGCCCA
GGTCATCGTCCTGAACCACCCCGGTCAGGTCGGCGCTGGCTACGCTCCCGTCTTGGATTGC
CACACTGCCCACATCGCCTGCAAGTTCTCTGAGATCCTCGAGAAGATCGACCGCCGAACGG
GCAAGTCTGTCGAGAACAACCCCAAGTTCATCAAGTCTGGTGACGC
>Distoseptispora_saprophytica_MFLUCC_18_1238
ACCT--------TT---TTTC-------TGCGCTCCGGTGGG-CGGTTCC-AGGGCGGAGCCACTGCCCGT
TGGGTTGGGTGCCCGCCGGAGCGAACCCCT--AAACTCTTGCTGTTA--CAGTGAGG-ATCAGA

GATAA--------AAT---ACAAGCTAT-AAAAACTTTCAGCAACGGATCTCTTGGCTCTGGCATCGATG
AAGAACGCAGCGAAATGCGATAAATAATGTGAATTGCAGACTTCTG-GAATCATCGAGTTTTTG
AACGCACATTGCGCCCGTCAGTATTCTGGCGGGCATGCCTGTCCGAGCGTCATTTCAA-CCC
TCAGGCCCCCGTAA-------GG-TCTGGTGTTGGGGCGCTGCGAG-CTCGCTGT-------------GGC
CGAGCGCGCAGGCCCTCAAATGTAGTGGCGGAACCGCTGGGGCT-CCGGGCGCAGTAGATT
-GCTA---TCGCCCCGAGCCTCC-CGGCGG--GA-GC--CTGCCATCAA-GCTCCATC------TTTACC
AA----------GGCCTTCTGAGTCCCC-TGGAAC------GGGGCGCC-AGAGAGGG-TGAGAGCCCCG
TACGGTCGGACACCGAGCC-TCTGTAAAGCTCCTTCGACGAGTCGAGTAGTTTGGGAATGCT
GCTCTAAATGGGAGGTAAATTTCTCCTAAAG-CTAAATATAGGCCAGAGACCGATAGCGCACAA
GTAGAGTGATCGAAAGATGAAAAGCACTTTGAAAAGAGGGTTAAATAGCACGTGAAATTGCTG
AAAGGGAAGCGCTTGTGACCAGACTTGCGCCCGGTGGATCAGCTGGCGTTCTCGCCAGTG
CACTCTGCCGGGCTCAGGCCAGCATCGGTTCCGACGGGGGACAAAGGCCGCTGGAACGT
AGCTCCCCTCGGGGAGTGTTATAGCCAGCGG-TGTCATACCCCCAGCCGGGACCGAGGATC
GCGCTCTG-CAAGGATGCTGGCGTAATGGTCACCAGCGACCCGTCTTTGTCTCTTATGTGTTA
TGTCAGCGTAGGGACGACGGCTGAGCCTATCAAGGACTTTTTGATTGCCAAACAGATGGAAA
TTCTTGAAGAGTACGAGCCACTTCGGTATCCCAACGCGACAAAGATTTTCATCAACGGTTCGT
GGGTCGGTGTGCATCAAGACCCACAGAATCTTGTGTCTGCGGTGAGATCCCTCCGGCGGAG
GGGCATCGTCCAGTTCGAGGTCACGCTTGTTAGAGACATTCGCGACCGAGAATTCAAGATTT
TCTCCGATGCAGGCCGGGTGATGCGCCCCCTCTTCGCGGTTGAACAAGAGGAACCAAACGA
GGAGAATAGCGGACGGGAGAAAGGAAGTCTGTTGCTTACGAAAGAGCATATTCAGCGACTTC
GAGATGACGAACGCCTGGATCCGAGTGATCCCAACTACTTTGGCTGGGATGGGCTTACCAG
GGATGGTGCTATTGAATATCTAGATGCCGAGGAGGAGGAAACGGCGATGATCTGTATGACGC
CTGAGGACCTGGAGC---
--ACTCGTGAGCACGCCCTGCTTGCCTACACC
CTGGGTGTGAAGCAGCTCATCGTTGCCATCAACAAGATGGACACCACCAAATGGTCTGAGGA
TCGTTTCAATGAAATCATCAAGGAGACCTCCAACTTCATCAAGAAGGTTGGATACAACCCCAA
GACTGTTGCTTTCGTCCCAATCTCCGGCTTCAACGGCGACAACATGCTTGCGCCCTCAAGCA
ACTGCCCCTGGTACAAGGGCTGGGAGAAGGAAG---TCAAGGGCGGCAAGGTTACCGGCAAG
ACCCTCCTCGAGGCTATTGACGCCATCGAGCCCCCTAAGCGCCCCACCGACAAGCCTCTCC
GTCTTCCCCTCCAGGATGTGTACAAGATTGGTGGTATCGGAACTGTCCCCGTCGGCCGTATC
GAGACTGGTGTCCTCAAGCCTGGTATGGTCGTGACTTTCGCTCCCTCCAACGTCACCACTGA
AGTCAAGTCCGTCGAGATGCACCACGAGCAGCTTACTGAGGGTGTTCCCGGCGACAACGTT
GGCTTCAACGTGAAGAACGTCTCCGTCAAGGAAATCCGCCGTGGCAACGTCGCCGGTGACT
CCAAAAACGACCCTCCAATGGGCTGCGCTTCCTTCACTGCCCAGGTCATTGTGTTGAACCAC
CCTGGCCAGGTTGGTGCCGGTTACGCCCCTGTTCTTGATTGCCACACCGCCCACATCGCCT
GCAAATTCTCCGAGATCCTCGAGAAGATCGACCGCCGAACGGGCAAGTCGGTTGAGAACAA
CCCCAAGTTCATCAAGTCGGGCGACGC
>Distoseptispora_septata_GZCC_22_0078
ACCT--------TT-ACGTT-------CGTTGCTTTGGCGGG-CGGCTCC-AGGGCGGAGCCACTGTCCG
TTGGGTCGGACGCCCGCCGAAGGA---CAAC-CAAAACTCGCT-AAATTTGT-ACGGATCTCAGA
GTAAACA-----TAT-----CAAGCTAT-CAAAACTTTCAGCAACGGATCTCTTGGTTCTGGCATCGAT
GAAGAACGCAGCGAAATGCGATAAGTAATGTGAATTGCAGAATTCAGTGAATCATCGAATCTT
TGAACGCACATTGCGCCCGCCAGCACTCTGGCGGGCATGCCTGTTCGAGCGTCATTTCAA-C
CCTCGGGCCC-TCGTT-------TGGCCCGGTGTTGGGGCGCTACGGGTCCG--TC----------------G

GAC-CTGTAGGCCCTCAAAAGCAGTGGCGGGCTCGCCAGGACT-CCGAGCGTAGTAGTTT-TC
CT--CTCGCTCAGGCGGCCT-GGCGTG--CT-TCGGCCGTTAAA----ACCCAAC------TGTAAT-----C
ACAG-AAGCCTTCCGAGTCCCC-TGGAAC------GGGGCGCC-AGAGAGGG-TGAGAGCCCCGT
ATGGTTGGACGCCGAACC-TTTGTAAAGCTCCTTCGACGAGTCGAGTAGTTTGGGAATGCTG
CTCAAAATGGGAGGTAAATTTCTTCTAAAG-CTAAATACCGGCCAGAGACCGATAGCGCACAA
GTAGAGTGATCGAAAGATGAAAAGCACTTTGAAAAGAGGGTTAAATAGCACGTGAAATTGCTG
AAAGGGAAGCGCTTGTGACCAGACTTGCGCCCGGTTGATCATCCAGCGTTCTCGCTGGTGC
ACTCTGCCGGGCTCAGGCCAGCATCGGCTCTCCCAGGGGGATAAAGGTCGCGGGAACGTA
GCTCTCTCCGGGGAGTGTTATAGCCCGTGG-CGGAATGCCCTTAGGGGGGGCCGAGGCTCG
CGTTC-G-CAAGGATGCTGGCGTAATGGTCACCAGCGACCCGTCTTTATCGCTCATGTGTTATG
TGAGCGTCGGAACGCCCGCAGAGCCAATCGTCGAGTTCATGATTGCTAGGAACATGGAAGTA
CTCGAGGAATATGAACCCTTGAGGTACCCTAATGCGACCAAAATCTTCGTCAATGGCACATGG
GTTGGTGTTCACCAAGACCCGAAGCATCTTGTGAGCTTGGTTCAGGACTTACGCCGCAGGG
GCGTCATCAACTTCGAGGTCTCCCTCATTCGGGATATCCGCGATCGAGAGTTCAAGATTTTCT
CGGATGCTGGTCGCGTTATGCGTCCTCTGTTCGCTGTGGAGCAGTCAGAC---AACTCGACAA
AG---GGCCTTGAAAAGGGCTCTTTGTTGCTCACAAAGGAGCATATTCAGAAGTTGAGAGCAGA
TGAGGAGTTATCCAAGGATGACTCAGACTATTTCGGCTGGGATGGCCTTCTCAGGGCGGGC
GCCATCGAATATCTTGACGCCGAAGAGGAAGAGACAGCCATGATTTGCATGACTCCAGAAGA
TTTAGAGGCTTATCGCCTGCAAAAAGCTGGATTAGACGTGCCGGAAGACGATGACGAGGCCG
---ACCCCAATAGGCGTCTGAAGACGAAATTGAACCCCACCACTCATATGTATACACACTGTGA
GATCCATCCGAGCATGCTCTTGGGGATCTGCGCGAGCATTATTCCACTCGTGAGCACGCTCT
GCTTGCATACACCCTGGGTGTGAAGCAGCTCATCGTCGCCATCAACAAGATGGACACTACCA
AGTGGTCCGAGAGCCGTTACAACGAAATTATCAAGGAGACGTCCAACTTCATCAAGAAGGTC
GGCTACAACCCCAAGCAGGTCGCCTTCGTTCCCATCTCTGGCTTCAACGGCGACAACATGCT
TGCCCCCTCCGCCAACTGCCCCTGGTACAAGGGCTGGGAGAAGGAGATC---AAGGGCGGCA
AGGCCTCTGGCAAGACCCTTCTCGAGGCCATCGATGCCATTGAGCCTCCTAAGCGTCCTACG
GACAAGCCTCTGCGTCTCCCCCTCCAGGATGTCTACAAGATCGGTGGTATTGGAACTGTGCC
CGTCGGCCGTATCGAGACTGGTATCCTCAAGCCCGGTATGGTCGTCACATTCGCTCCCTCCA
ACGTCACCACTGAAGTCAAGTCCGTCGAGATGCACCACGAGCAGCTGCAGGAGGGTGTCCC
CGGCGACAACGTTGGCTTCAACGTGAAGAACGTCTCCGTCAAGGAAATCCGCCGAGGCAAC
GTCGCTGGTGACTCCAAGAACGATCCTCCGGCTGGTGCCGCTTCCTTCACCGCCCAGGTCA
TCGTCCTCAACCACCCCGGTCAGGTCGGCGCTGGCTACGCTCCCGTCTTGGATTGCCACAC
TGCCCACATCGCTTGCAAGTTCTCCGAGATTCTTGAAAAGATCGACCGCCGAACGGGCAAGT
CTGTTGAGAACAACCCCAAGTTCATCAAGTCTGGTGACGC
>Distoseptispora_sinensis_HJAUP_C2044
ACCT--------TT-ATATT-------CGTTGCTTTGGCGGG-CGGCTCC-AGGGCGGAGCCACTGCCCGT
TGGGTCGGGCGCCCGCCGAAGGA---TAAC-CAAAAACTCGTTAAATCTGT-ACGGATCTCAGA
GTAAAA------TAT-----CAAGCTAT-CAAAACTTTCAGCAACGGATCTCTTGGTTCTGGCATCGATG
AAGAACGCAGCGAAATGCGATAAGTAATGTGAATTGCAGAATTCAGTGAATCATCGAATCTTT
GAACGCACATTGCGCCCGCCAGCACTCTGGCGGGCATGCCTGTTCGAGCGTCATTTCAA-CC
CTCGGGCCC-TCGTT-------TGGCCTGGTGTTGGGGCGCTACGGGTCCCATCG-----------------G-
AC-CTGTAGGCCCTTAAAATCAGTGGCGGGCTCGCCAGGACT-CCGAGCGTAGTAGTTC-TCC
T--CTCGCTTAGGCGGTCT-GGCGTG--CT-TCGGCCGTTAAA---TACCCAGC------GGTAAT-CGC
GCACAC-AAGCCTTCCGAGTCCCC-TGGAAC------GGGGCGCC-AGAGAGGG-TGAGAGCCCC

GTATGGTTGGACGCCGAACC-TCTGTAAAGCTCCTTCGACGAGTCGAGTAGTTTGGGAATGCT
GCTCAAAATGGGAGGTAAATTTCTTCTAAAG-CTAAATACCGGCCAGAGACCGATAGCGCACA
AGTAGAGTGATCGAAAGATGAAAAGCACTTTGAAAAGAGGGTCAAATAGCACGTGAAATTGCT
GAAAGGGAAGCGCTTGTGACCAGACTTGCGCCCGGTTGATCATCCAGCGTTCTCGCTGGTG
CACTCTGCCGGGCTCAGGCCAGCATCGGCTCTCCCAGGGGGATAAAGGTCGCGGAACGT
AGCTCTCTCCGGGGAGTGTTATAGCCCGTGG-CGGAATGCCCTTAGGGGGGGCCGAGGCCC
GCGTTC-G-CAAGGATGCTGGCGTAATGGTCACCAGCGACCCGTCTT???????????????????
??
??
??
??
??
??
??
??
??ACTCGTGAGCACGCTCTGC
TCGCCTATACGCTTGGTGTGAAGCAGCTCATCGTCGCCATCAACAAGATGGACACCACCAAG
TGGTCTGAGAGCCGTTACAACGAAATTATCAAGGAGACCTCCAACTTCATCAAGAAGGTTGG
CTACAACCCCAAGCAGGTCGCCTTCGTTCCCATCTCCGGCTTCAACGGCGACAACATGCTTG
CCGCCTCCTCCAACTGCCCCTGGTACAAGGGCTGGGAGAAGGAGATC---AAGGGTGGCAAG
GCCACTGGCAAGACCCTCCTCGAGGCTATCGATGCCATTGAGCCTCCCAAGCGTCCCACAG
ACAAGCCTCTCCGTCTCCCCCTTCAGGATGTCTACAAGATCGGTGGTATCGGAACTGTGCCC
GTCGGCCGTATCGAGACTGGTATCCTCAAGCCCGGTATGGTCGTCACGTTCGCTCCCTCCAA
CGTCACCACTGAAGTCAAGTCCGTCGAGATGCACCACGAGCAGCTGCAGGAGGGTGTCCC
CGGCGACAACGTTGGCTTCAACGTGAAGAACGTCTCCGTCAAGGAAATCCGCCGAGGCAAC
GTCGCTGGTGACTCCAAGAACGATCCTCCTGCTGGTGCCGCTTCCTTCACCGCTCAGGTCAT
CGTCCTCAACCACCCCGGTCAGGTCGGTGCTGGTTACGCTCCCGTCTTGGATTGCCACACA
GCCCACATTGCTTGCAAGTTCGCTGAGATCCTTGAGAAGATCGACCGCCGAACGGGCAAGT
CTGTTGAGAACAACCCCAAGTTCATCAAGTCTGGTGACGC
>Distoseptispora_songkhlaensis_MFLUCC_18_1234
ACCT--------GCT--TTT-------CGTTGCTTCGGCGGG-CGGCTCC-AGGGCGGAGCCACTGCCCGT
TTGTTCGGGCGCCCGCCGGAGGA---CAAC-CAAAACTAGCC-TGGTCTTTGGGTAACGTCAGA
GTATTA----AAAAA----AAAAGCTAT-CAAAACTTTCAGCAACGGATCTCTTGGTTCTGGCATCGAT
GAAGAACGCAGCGAAATGCGATAAGTAATGTGAATTGCAGAATTCAGTGAATCATCGAATCTT
TGAACGCACATTGCGCCCGCCAGCACTCTGGCGGGCATGCCTGTTCGAGCGTCATTTCAA-C
CCTCGGGCCCCCCTT-----GTGTGGCCCGGTGTTGGGGCGCTACCGTCCCCCTC---------------
GGGGGACCCGGTAGGCCCTTAAAACCAGTGGCGGGCTCGCCACGGCCTCCGAGCGCAGTA
GTCA-TCTC--CTCGCTCAGGCGGCCT-GGCGCG--CC-TCTGCCGTTAAA---CATACA-C------TGT
ACCAC----ACAG-AAGCCTTCCGAGTCCCC-TGGAAC------GGGGCGCC-AGAGAGGG-TGAGA
GCCCCGTACGGTCGGACGCCGAACC-TCTGTAAAGCTCCTTCGACGAGTCGAGTAGTTTGGG
AATGCTGCTCAAAATGGGAGGTAAATTTCTTCTAAAG-CTAAATACCGGCCAGAGACCGATAGC
GCACAAGTAGAGTGATCGAAAGATGAAAAGCACTTTGAAAAGAGGGTTAAATAGCACGTGAAA
TTGCTGAAAGGGAAGCGCTTGTGACCAGACTTGCGCCCGGTTGATCATCCAGCGTTCTCGC
TGGTGCACTCTGCCGGGCTCAGGCCAGCATCGGCTCCCCCAGGGGGATAAAGGTCGCGGG

AACGTAGCTCTCTCCGGGGAGTGTTATAGCCCGCGG-CGGAATACCCTTAGGGGGGGTCGA
GGCCCGCGTTC-G-CAAGGATGCTGGCGTAATGGTCACCAGCGACCCGTCTT?????????????
??
??
??
??
??
??
??
??
???ACTCGTGAGCACGC
TCTGCTTGCCTACACCCTGGGTGTGAAGCAGCTCATCGTCGCTATCAACAAGATGGACACCA
CCAAGTGGTCTGAGGATCGTTACAACGAAATCATCAAGGAGACGTCCAACTTCATCAAGAAG
GTCGGCTACAACCCCAAGCAAGTTGCTTTCGTTCCCATCTCCGGCTTCAACGGCGACAACAT
GCTTGCCCCCTCCACCAACTGCCCCTGGTACAAGGGCTGGGAGAAGGAGGTC---AAGGGTG
GCAAAGTCACTGGCAAGACGCTTCTCGAAGCCATCGATGCCATTGAGCCTCCCAAGCGTCC
CACAGACAAGCCGCTGCGTCTTCCCCTCCAGGATGTCTACAAGATCGGTGGTATCGGAACCG
TACCTGTCGGCCGTATCGAGACTGGTGTCCTCAAGCCCGGTATGGTCGTCACTTTCGCTCCC
TCCAACGTCACCACTGAAGTGAAGTCGGTCGAGATGCACCACGAGCAGCTTCAAGAGGGTG
TTCCTGGCGACAACGTCGGCTTCAACGTGAAGAACGTCTCTGTCAAGGAAATCCGCCGAGG
CAACGTCGCTGGTGACTCCAAGAACGATCCTCCTGCTGGCGCTGCTTCCTTCACCGCCCAG
GTTATTGTCTTGAACCACCCCGGTCAGGTTGGTGCCGGCTACGCTCCCGTCTTGGACTGCCA
CACTGCCCACATCGCCTGCAAGTTCTCCGAGATTCTCGAGAAGATCGATCGCCGAACGGGC
AAGTCCGTCGAGAACAACCCCAAGTTCATCAAGTCCGGTGACGC
>Distoseptispora_suoluoensis_MFLUCC_17_0224
ACCT--------ATCGTCAAA------TGTTGCTTCGGCGGG-CGGCCCC-TGGGAGGGGCCAAGGCCC
GTTGGGTTGGGTGCCCGCCGGAGGA---CAGC-CGAAAACTCTTTTTT--ATCAGGTACTGTCTG
AGTAAA------CTATAA-AAA---CTAT-AAAAACTTTCAACAACGGATCTCTTGGTTCTGGCATCGAT
GAAGAACGCAGCGAAATGCGATAAGTAATGTGAATTGCAGAATTCAGTGAATCATCGAATCTT
TGAACGCACATTGCGCCCGCCAGCACTCTGGCGGGCATGCCTGTTCGAGCGTCATTTCA-AC
CCTCAGGCCT-TA-------GTTTGGCCTGGTGTTGGGGCACTGCCGTCCCGCCC---------------GTA
CGGGGCGCGCAGGCCCTGAAAACCAGTGGCGGGCTCGCTAGCAC-TCCGGGCGTAGTAGA-
ATCAT-ATCTCGTCTAGGTGGTCT-GGCGCG--GT-CTTGCCGTCAAAAC-CCCCC---------A-TTTT
AT-------ACAAGCCTTCTAAGTCCCC-TAGAAC------GGGGCGCC-ACAGAGGG-TGAGAGCCCC
GTACAGTTGGACACCGAACC-TTTGTAAAGCTCCTTCGACGAGTCGAGTAGTTTGGGAATGCT
GCTCAAAATGGGAGGTATATTTCTTCTAAAG-CTAAATACCGGCCAGAGACCGATAGCGCACAA
GTAGAGTGATCGAAAGATGAAAAGCACTTTGAAAAGAGGGTTAAATAGCACGTGAAATTGTTG
AAAGGGAAGCGCTTGTGACCAGACTTGCGCCCGGTGAATCATCCAGCGTTCTCGCTGGTGC
ACTTTGCCGGGCTCAGGCCAGCATCGGTTCTCCCAGGGGGATAAAGGTCTCGGGAACGTAG
CTCTCTTCGGGGAGTGTTATAGCCCGTGG-CATAATACCTTTGGG-GGGACCGAGGACCGCG
CTCTG-CAAGGATGCTGGCGTAATGGTCATCAGCGACCCGTCTT??????????????????????
??
??
??

??
??
??
??
??
??ACTCGTGAGCACGCCCTGCTTG
CCTACACCCTGGGTGTCAAGCAACTCATCTGCGCCATCAACAAGATGGACACCACCAAGTGG
TCAGAGGACCGTTTCAACGAAATCATCAAGGAGACGTCCAACTTCATCAAGAAGGTCGGCTA
CAACCCCAAGCAGGTCGCCTTCGTCCCTATCTCTGGCTTCCACGGCGACAACATGTTGGCTG
CCTCCACCAACTGCCCCTGGTACAAGGGCTGGGAGAAGGAGGGCGCCAAGGGTGCCAAGT
CTTCTGGTAAGACCCTGCTCGAGGCCATCGACGCCATCGAGCAACCCAAGCGTCCTACCGA
CAAGCCTCTGCGTCTCCCGCTCCAGGATGTCTACAAGATCGGTGGTATCGGAACTGTCCCTG
TCGGCCGTATCGAGACTGGTGTCCTCAAGCCCGGTATGGTCGTCACTTTCGCTCCCTCCAAC
GTCACCACTGAAGTCAAGTCCGTCGAGATGCACCACGAGCAGCTTACTGAGGGTGTTCCCG
GCGACAACGTCGGCTTCAACGTGAAGAACGTTTCCGTCAAGGAAATCCGCCGTGGAAACGT
CGCTGGTGACTCCAAGAACGACCCTCCTGCTGGCTGCGCCAACTTCACTGCTCAGGTCATT
GTCCTCAACCACCCTGGTCAGGTCGGCCCTGGTTACGCTCCCGTCTTGGATTGCCACACCG
CCCACATCGCCTGCAAGTTCTCTGAGATCACCGAGAAGATCGACCGCCGTACCGGCAAGTC
AGTTGAGAACAACCCCAAGTTCATCAAGTCTGGTGACGC
>Distoseptispora_suoluoensis_MFLU_17_0854
ACCT--------ATCGTCAAA------TGTTGCTTCGGCGGG-CGGCCCC-TGGGAGGGGCCAAGGCCC
GTTGGGTTGGGTGCCCGCCGGAGGA---CAGC-CGAAAACTCTTTTTT--ATCAGGTACTGTCTG
AGTAAA------CTATAA-AAA---CTAT-AAAAACTTTCAACAACGGATCTCTTGGTTCTGGCATCGAT
GAAGAACGCAGCGAAATGCGATAAGTAATGTGAATTGCAGAATTCAGTGAATCATCGAATCTT
TGAACGCACATTGCGCCCGCCAGCACTCTGGCGGGCATGCCTGTTCGAGCGTCATTTCA-AC
CCTCAGGCCT-TA-------GTTTGGCCTGGTGTTGGGGCACTGCCGTCCCGCCC---------------GTA
CGGGGCGCGCAGGCCCTGAAAACCAGTGGCGGGCTCGCTAGCAC-TCCGGGCGTAGTAGA-
ATCAT-ATCTCGTCTAGGTGGTCT-GGCGCG--GT-CTTGCCGTCAAAAC-CCCCC---------A-TTTT
AT-------ACAAGCCTTCTAAGTCCCC-TAGAAC------GGGGCGCC-ACAGAGGG-TGAGAGCCCC
GTACAGTTGGACACCGAACC-TTTGTAAAGCTCCTTCGACGAGTCGAGTAGTTTGGGAATGCT
GCTCAAAATGGGAGGTATATTTCTTCTAAAG-CTAAATACCGGCCAGAGACCGATAGCGCACAA
GTAGAGTGATCGAAAGATGAAAAGCACTTTGAAAAGAGGGTTAAATAGCACGTGAAATTGTTG
AAAGGGAAGCGCTTGTGACCAGACTTGCGCCCGGTGAATCATCCAGCGTTCTCGCTGGTGC
ACTTTGCCGGGCTCAGGCCAGCATCGGTTCTCCCAGGGGGATAAAGGTCTCGGGAACGTAG
CTCTCTTCGGGGAGTGTTATAGCCCGTGG-CATAATACCTTTGGG-GGGACCGAGGACCGCG
CTCTG-CAAGGATGCTGGCGTAATGGTCATCAGCGACCCGTCTTTATCGCTCATGTGTTATGT
CAGCGTTGGCACACCGGCAGAACCGATTATCGAGTTTATGATCGCGAGGAACATGGAAGTCC
TCGAGGAATATGAGCCGTTGCGCTATCCCAACGCAACTAAGATATTTGTCAACGGGACTTGGG
TGGGCGTTCACCAGGACCCCAAACACTTGGTTGGGCTGGTGCAGGAGTTGCGCCAGAGGG
GAGTTATCAACTTTGAGGTTTCCCTTGTGCGTGATATCCGAGACAGGGAGTTCAAGATCTTCT
CCGATGCAGGTCGGGTGATGCGACCCCTTTTCGCCGTGGAGCAGGAGGAC---AATTCTAAGT
CG---GGGCTTCCAAAGGGATCTTTGAGACTCACCAAAGAGCATATCCAGAGGCTCAGAGCGG
ACGAAGACTTGTCCAAAGAAGATCCGGAATACTTCGGATGGGACGGCATCGTTGAGGCTGG
CGCAGTCGAATATCTCGACGCGGAAGAAGAGGAAACGGCTATGATATGCATGACGCCGGAAG

ACCTGGAGGCTTACAGACTCCAAAAAGCCGGGTTGGGCATGGCAGAAGATGACGAAGAAGC
GG---ACCCAAACAGACGCCTGAAAACGAAATTAAACCCTACCACTCACATGTACACACACTGC
GAGATTCATCCCAGTATGCTATTGGGAATCTGCGCAAGCATCATACC???????????????????????
???
???
???
???
???
???
???
???
???
???
???????
>Distoseptispora_tectonae_MFLUCC_12_0291
ACCT--------TT-ATATT-------CGTTGCTTTGGCGGG-CGGCTCC-AGGGCGGAGCCACTGCCCGT
TGGGTCGGGCGCCCGCCGAAGGA---CAAC-CAAAAACTCGTTAAATCTGT-ACGGATCTCAGA
GTAAAA------TAT-----CAAGCTAT-CAAAACTTTCAGCAACGGATCTCTTGGTTCTGGCATCGATG
AAGAACGCAGCGAAATGCGATAAGTAATGTGAATTGCAGAATTCAGTGAATCATCGAATCTTT
GAACGCACATTGCGCCCGCCAGCACTCTGGCGGGCATGCCTGTTCGAGCGTCATTTCAA-CC
CTCGGGCCC-TCGTT-------TGGCCTGGTGTTGGGGCGCTACGGGTCTCACCG------------------G
GAC-CTGTAGGCCCTCAAAATCAGTGGCGGGCTCGCCAGGACT-CCGAGCGTAGTAGTTC-TC
CT--CTCGCTCAGGCGGTCT-GGCGTG--CT-TCGGCCGTTAAA---TACCCAGC------GGTAAT-CG
CACACA---AGCCTTCCGAGTCCCC-TGGAAC------GGGGCGCC-AGAGAGGG-TGAGAGCCCC
GTATGGTTGGACGCCGAACC-TCTGTAAAGCTCCTTCGACGAGTCGAGTAGTTTGGGAATGCT
GCTCAAAATGGGAGGTAAATTTCTTCTAAAG-CTAAATACCGGCCAGAGACCGATAGCGCACA
AGTAGAGTGATCGAAAGATGAAAAGCACTTTGAAAAGAGGGTCAAATAGCACGTGAAATTGCT
GAAAGGGAAGCGCTTGTGACCAGACTTGCGCCCGGTTGATCATCCAGCGTTCTCGCTGGTG
CACTCTGCCGGGCTCAGGCCAGCATCGGCTCTCCCAGGGGGATAAAGGTCGCGGGAACGT
AGCTCTCTCCGGGGAGTGTTATAGCCCGTGG-CGGAATGCCCTTAGGGGGGGCCGAGGCCC
GCG-TTCG-CAAGGATGCTGGCGTAATGGTCACCAGCGACCCGTCTTTATCGCTCATGTGTTAT
GTGAGCGTGGGAACGCCCGCAGAGCCAATCGTCGAGTTCATGATTGCTAGGAACATGGAAG
TACTCGAGGAATATGAGCCCTTGAGGTACCCTAATGCGACCAAAATCTTCGTTAATGGCACAT
GGGTTGGTGTTCACCAAGACCCAAAGCATCTTGTGAGCTTGGTTCAGGACTTACGCCGCAGA
GGCGTCATCAACTTTGAGGTCTCTCTCATTCGGGATATCCGCGATCGAGAGTTCAAGATTTTC
TCGGATGCTGGTCGTGTTATGCGTCCTCTGTTCGCTGTGGAGCAGTCAGAC---AACTCGACA
AAG---GGTCTTGAAAAGGGGTCTTTACTGCTCACAAAGGAGCATATTCAAAAATTGAGAGCTG
ATGAGGAGTTATCCAAGGACGACCCAGACTATTTTGGCTGGGATGGCCTTCTCAGAGCGGGC
GCTATCGAATATCTTGACGCCGAAGAGGAAGAGACAGCCATGATTTGCATGACTCCAGAAGAT
CTAGAGGCTTATCGGCTGCAAAAAGCTGGATTAGACGTGCCGGAAGACGATGACGAGGCCG-
--ACCCCAATAGGCGTCTGAAGACGAAACTGAACCCCACGACTCACATGTATACACATTGCGA
GATTCATCCAAGCATGCTCTTGGGGATCTGTGCGAGCATTATCCCACTCGTGAGCACGCTCT
GCTCGCCTACACGCTTGGTGTGAAGCAGCTCATCGTCGCCATCAACAAGATGGACACCACCA
AGTGGTCTGAGAGCCGTTACAACGAAATTATCAAGGAGACCTCCAACTTCATCAAGAAGGTC

GGCTACAACCCCAAGCAGGTCGCCTTCGTTCCCATCTCCGGCTTCAACGGCGACAACATGC
TTGCCCCCTCCTCCAACTGCCCCTGGTACAAGGGCTGGGAGAAGGAGATC---AAGGGTGGC
AAGGCCACTGGCAAGACCCTCCTCGAGGCTATCGATGCCATTGAGCCTCCCAAGCGTCCCA
CAGACAAGCCTCTCCGTCTCCCCCTTCAGGATGTCTACAAGATCGGTGGTATCGGAACTGTG
CCCGTCGGCCGTATCGAGACTGGTATCCTCAAGCCCGGTATGGTCGTCACGTTCGCTCCCTC
CAATGTCACCACTGAAGTCAAGTCCGTCGAGATGCACCACGAGCAGCTGCAGGAGGGTGTC
CCCGGCGACAACGTTGGCTTCAACGTGAAGAACGTCTCCGTCAAGGAAATCCGCCGAGGCA
ACGTCGCTGGTGACTCCAAGAACGATCCTCCTGCTGGTGCCGCTTCCTTCACCGCTCAGGT
CATCGTCCTCAACCACCCCGGTCAGGTCGGTGCTGGTTACGCTCCCGTCTTGGATTGCCAC
ACAGCCCACATTGCTTGCAAGTTCGCTGAGATCCTTGAGAAGATCGACCGCCGAACGGGCA
AGTCTGTTGAGAACAACCCCAAGTTCATCAAGTCTGGTGACGC
>Distoseptispora_tectonae_MFLUCC_15_0981
ACCT--------TT-ATATT-------CGTTGCTTTGGCGGG-CGGCTCC-AGGGCGGAGCCACTGCCCGT
TGGGTCGGGCGCCCGCCGAAGGA---CAAC-TAAAAACTCGTTAAACTTAT-ACGGATCTCAGA
GTAAAAA-----TAT-----CAAGCTAT-TAAAACTTTCAGCAACGGATCTCTTGGTTCTGGCATCGATG
AAGAACGCAGCGAAATGCGATAAGTAATGTGAATTGCAGAATTCAGTGAATCATCGAATCTTT
GAACGCACATTGCGCCCGCCAGCACTCTGGCGGGCATGCCTGTTCGAGCGTCATTTCAA-CC
CTCGGGCCC-TCGTT-------TGGCCTGGTGTTGGGGCGCTACGGGTCTCACCG----------------G-
AC-CTGTAGGCCCTCAAAATCAGTGGCGGGCTCGCCAGGACT-CCGAGCGTAGTAGTTC-TCC
T--CTCGCTCAGGCGGTCT-GGCGTG--CT-TCGGCCGTTAAA---TACCCAGC------GGTAAT-CGC
GCACAT-AAGCCTTCCGAGTCCCC-TGGAAC------GGGGCGCC-AGAGAGGG-TGAGAGCCCC
GTACGGTTGGACGCCGAACC-TCTGTAAAGCTCCTTCGACGAGTCGAGTAGTTTGGGAATGC
TGCTCAAAATGGGAGGTAAATTTCTTCTAAAG-CTAAATACCGGCCAGAGACCGATAGCGCAC
AAGTAGAGTGATCGAAAGATGAAAAGCACTTTGAAAAGAGGGTCAAATAGCACGTGAAATTGC
TGAAAGGGAAGCGCTTGTGACCAGACTTGCGCCCGGTTGATCATCCAGCGTTCTCGCTGGT
GCACTCTGCCGGGCTCAGGCCAGCATCGGCTCTCCCAGGGGGATAAAGGTCGCGGGAACG
TAGCTCTCTCCGGGGAGTGTTATAGCCCGTGG-CGGAATGCCCTTAGGGGGGGCCGAGGCC
CGCGTTC-G-CAAGGATGCTGGCGTAATGGTCACCAGCGACCCGTCTT??????????????????
??
??
??
??
??
??
??
??
???ACTCGTGAGCACGCTCTG
CTCGCCTACACGCTTGGTGTGAAGCAGCTCATCGTCGCCATCAACAAGATGGACACCACCAA
GTGGTCTGAGAGCCGTTACAACGAAATTATCAAGGAGACCTCCAACTTCATCAAGAAGGTCG
GCTACAACCCCAAGCAGGTCGCCTTCGTTCCCATCTCCGGCTTCAACGGCGACAACATGCTT
GCCCCCTCCTCCAACTGCCCCTGGTACAAGGGCTGGGAGAAGGAGATC---AAGGGTGGCAA
GGCCACTGGCAAGACCCTCCTCGAGGCTATCGATGCCATTGAGCCTCCCAAGCGTCCCACA
GACAAGCCTCTCCGTCTCCCCCTTCAGGATGTCTACAAGATCGGTGGTATCGGAACTGTGCC
CGTCGGCCGTATCGAGACTGGTATCCTCAAGCCCGGTATGGTCGTCACGTTCGCTCCCTCCA

ACGTCACCACTGAAGTCAAGTCCGTCGAGATGCACCACGAGCAGCTGCAGGAGGGTGTCCC
CGGCGACAACGTTGGCTTCAACGTGAAGAACGTCTCCGTCAAGGAAATCCGCCGAGGCAAC
GTCGCTGGTGACTCGAAGAACGATCCTCCTGCTGGTGCCGCTTCCTTCACCGCTCAGGTCA
TCGTCCTCAACCACCCCGGTCAGGTCGGTGCTGGTTACGCTCCCGTCTTGGATTGCCACAC
AGCCCACATTGCTTGCAAGTTCGCTGAGATCCTTGAGAAGATCGACCGCCGAACGGGCAAG
TCTGTTGAGAACAACCCCAAGTTCATCAAGTCTGGTGACGC
>Distoseptispora_tectonae_MFLUCC_16_0946
ACCT--------TT-ATATT-------CGTTGCTTTGGCGGG-CGGCTCC-AGGGCGGAGCCACTGCCCGT
TGGGTCGGGCGCCCGCCGAAGGA---CAAC-CAAAAACTCGTTAAATCTGT-ACGGATCTCAGA
GTAAAA------TAT-----CAAGCTAT-CAAAACTTTCAGCAACGGATCTCTTGGTTCTGGCATCGATG
AAGAACGCAGCGAAATGCGATAAGTAATGTGAATTGCAGAATTCAGTGAATCATCGAATCTTT
GAACGCACATTGCGCCCGCCAGCACTCTGGCGGGCATGCCTGTTCGAGCGTCATTTCAA-CC
CTCGGGCCC-TCGTT-------TGGCCTGGTGTTGGGGCGCTACGGGTCTC-ACC-----------------AT
AC-CTGTAGGCCCTCAAAATCAGTGGCGGGCTCGCCAGGACT-CCGAGCGTAGTAGTTC-TCC
T--CTCGCTCAGGCGGTCT-GGCGTG--CT-TCGGCCGTTAAA---TACCCAGC------GGTAAT-CG--
CACAC-AAGCCTTCCGAGTCCCC-TGGAAC------GGGGCGCC-AGAGAGGG-TGAGAGCCCCG
TATGGTTGGACGCCGAACC-TCTGTAAAGCTCCTTCGACGAGTCGAGTAGTTTGGGAATGCTG
CTCAAAATGGGAGGTAAATTTCTTCTAAAG-CTAAATACCGGCCAGAGACCGATAGCGCACAA
GTAGAGTGATCGAAAGATGAAAAGCACTTTGAAAAGAGGGTCAAATAGCACGTGAAATTGCT
GAAAGGGAAGCGCTTGTGACCAGACTTGCGCCCGGTTGATCATCCAGCGTTCTCGCTGGTG
CACTCTGCCGGGCTCAGGCCAGCATCGGCTCTCCCAGGGGGATAAAGGTCGCGGGAACGT
AGCTCTCTCCGGGGAGTGTTATAGCCCGTGG-CGGAATGCCCTTAGGGGGGGCCGAGGCCC
GCG-TTCG-CAAGGATGCTGGCGTAATGGTCACCAGCGACCCGTCTTTATCGCTCATGTGTTAT
GTGAGCGTGGGAACGCCCGCAGAGCCAATCGTCGAGTTCATGATTGCTAGGAACATGGAAG
TACTCGAGGAATATGAGCCCTTGAGGTACCCTAATGCGACCAAAATCTTCGTTAATGGCACAT
GGGTTGGTGTTCACCAAGACCCAAAGCATCTTGTGAGCTTGGTTCAGGACTTACGCCGCAGA
GGCGTCATCAACTTTGAGGTCTCTCTCATTCGGGATATCCGCGATCGAGAGTTCAAGATTTTC
TCGGATGCTGGTCGTGTTATGCGTCCTCTGTTCGCTGTGGAGCAGTCAGAC---AACTCGACA
AAG---GGTCTTGAAAAGGGGTCTTTATTGCTCACAAAGGAGCATATTCAAAAATTGAGAGCTGA
TGAGGAGTTATCCAAGGACGACCCAGACTATTTTGGCTGGGATGGCCTTCTCAGAGCGGGC
GCTATCGAATATCTTGACGCCGAAGAGGAAGAGACAGCCATGATTTGCATGACTCCAGAAGAT
CTAGAGGCTTATCGGCTGCAAAAAGCTGGATT---
--ACTCGTGAGCACGCTCTGCTCGCCTA
CACGCTTGGTGTGAAGCAGCTCATCGTCGCCATCAACAAGATGGACACCACCAAGTGGTCT
GAGAGCCGTTACAACGAAATTATCAAGGAGACCTCCAACTTCATCAAGAAGGTCGGCTACAA
CCCCAAGCAGGTCGCCTTCGTTCCCATCTCCGGCTTCAACGGCGACAACATGCTTGCCCCC
TCCTCCAACTGCCCCTGGTACAAGGGCTGGGAGAAGGAGATC---AAGGGTGGCAAGGCCAC
TGGCAAGACCCTCCTCGAGGCTATCGATGCCATTGAGCCTCCCAAGCGTCCCACAGATAAGC
CTCTCCGTCTCCCCCTTCAGGATGTCTACAAGATCGGTGGTATCGGAACTGTGCCCGTCGGC
CGTATCGAGACTGGTATCCTCAAGCCCGGTATGGTCGTCACGTTCGCTCCCTCCAACGTCAC
CACTGAAGTCAAGTCCGTCGAGATGCACCACGAGCAGCTGCAGGAGGGTGTCCCCGGCGA
CAACGTTGGCTTCAACGTGAAGAACGTCTCCGTCAAGGAAATCCGCCGAGGCAACGTCGCT
GGTGACTCCAAGAACGATCCTCCTGCTGGTGCCGCTTCCTTCACCGCTCAGGTCATCGTCCT
CAACCACCCCGGTCAGGTCGGTGCTGGTTACGCTCCCGTCTTGGATTGCCACACAGCCCAC

ATTGCTTGCAAGTTCGCTGAGATCCTTGAGAAGATCGACCGCCGAACGGGCAAGTCTGTTGA
GAACAACCCCAAGTTCATCAAGTCTGGTGACGC
>Distoseptispora_tectonae_MFLU_20_0262
ACCT--------TT-ATATT-------CGTTGCTTTGGCGGG-CGGCTCC-AGGGCGGAGCCACTGCCCGT
TGGGTCGGGCGCCCGCCGAAGGA---CAAC-CAAAAACTCGTTAAATCTGT-ACGGATCTCAGA
GTAAAA------TAT-----CAAGCTAT-CAAAACTTTCAGCAACGGATCTCTTGGTTCTGGCATCGATG
AAGAACGCAGCGAAATGCGATAAGTAATGTGAATTGCAGAATTCAGTGAATCATCGAATCTTT
GAACGCACATTGCGCCCGCCAGCACTCTGGCGGGCATGCCTGTTCGAGCGTCATTTCAA-CC
CTCGGGCCC-TCGTT-------TGGCCTGGTGTTGGGGCGCTACGGGTCTCACCG------------------G
GAC-CTGTAGGCCCTCAAAATCAGTGGCGGGCTCGCCAGGACT-CCGAGCGTAGTAGTTC-TC
CT--CTCGCTCAGGCGGTCT-GGCGTG--CT-TCGGCCGTTAAA---TACCCAGC------GGTAAT-CG
CACACA---AGCCTTCCGAGTCCCC-TGGAAC------GGGGCGCC-AGAGAGGG-TGAGAGCCCC
GTATGGTTGGACGCCGAACC-TCTGTAAAGCTCCTTCGACGAGTCGAGTAGTTTGGGAATGCT
GCTCAAAATGGGAGGTAAATTTCTTCTAAAG-CTAAATACCGGCCAGAGACCGATAGCGCACA
AGTAGAGTGATCGAAAGATGAAAAGCACTTTGAAAAGAGGGTCAAATAGCACGTGAAATTGCT
GAAAGGGAAGCGCTTGTGACCAGACTTGCGCCCGGTTGATCATCCAGCGTTCTCGCTGGTG
CACTCTGCCGGGCTCAGGCCAGCATCGGCTCTCCCAGGGGGATAAAGGTCGCGGGAACGT
AGCTCTCTCCGGGGAGTGTTATAGCCCGTGG-CGGAATGCCCTTAGGGGGGGCCGAGGCCC
GCG-TTCG-CAAGGATGCTGGCGTAATGGTCACCAGCGACCCGTCTT???????????????????
???
???
???
???
???
???
???
???
???
???
???
???
???
???
???
???
???
???
???
???
>Distoseptispora_tectonigena_MFLUCC_12_0292
ACCT--------TT-ATATT-------CGTTGCTTTGGCGGG-CGGCTCC-AGGGCGGAGCCACTGCTCGT
TGGGTCGGGCGCCCGCCGAAGGA---CAAC-CAAAA-CTCATTGAATCTTTTGCGGATCTCAGA
GTAAAAA-----TAT-----CAAGCTAT-TAAAACTTTCAGCAACGGATCTCTTGGTTCTGGCATCGATG
AAGAACGCAGCGAAATGCGATAAGTAATGTGAATTGCAGAATTCAGTGAATCATCGAATCTTT

GAACGCACATTGCGCCCGCCAGCACTCTGGCGGGCATGCCTGTTCGAGCGTCATTTCAA-CC
CTCGGGCCC-TCGTT-------TGGCCCGGTGTTGGGGCGCTACGGGTCCGATCG-----------------G
-AC-CTGTAGGCCCTTAAAATCAGTGGCGGGCTCGCCAGGACT-CCGAGCGTAGTAGTTT-TCC
T--CTCGTTCAGGCGGTCT-GGCGTG--CC-TCGGCCGTTAAA---TATCCAGC------GGTAAT-CGC
ACACA----GCCTTCCGAGTCCCC-TGGAAC------GGGGCGCC-AGAGAGGG-TGAGAGCCCCGT
ATGGTTGGACGCCGAACC-TCTGTAAAGCTCCTTCGACGAGTCGAGTAGTTTGGGAATGCTG
CTCAAAATGGGAGGTAAATTTCTTCTAAAG-CTAAATACCGGCCAGAGACCGATAGCGCACAA
GTAGAGTGATCGAAAGATGAAAAGCACTTTGAAAAGAGGGTCAAATAGCACGTGAAATTGCT
GAAAGGGAAGCGCTTGTGACCAGACTTGCGCCCGGTTGATCATCCAGCGTTCTCGCTGGTG
CACTCTGCCGGGCTCAGGCCAGCATCGGCTCTCCCAGGGGGATAAAGGTCGCGGGAACGT
AGCTCTCTCCGGGGAGTGTTATAGCCCGTGG-CGGAATGCCCTTAGGGGGGGCCGAGGCCC
GCGTTC-G-CAAGGATGCTGGCGTAATGGTCACCAGCGACCCGTCTTTATCGCTCATGTGTTAT
GTGAGCGTGGGAACGCCCGCAGAGCCAATCGTCGAGTTCATGATTGCTAGGAACATGGAAG
TACTCGAGGAATATGAGCCCTTGAGGTACCCGAATGCGACCAAAATCTTCGTCAATGGCACAT
GGGTTGGTGTTCACCAAGACCCGAAGCATCTTGTGAGCTTGGTTCAGGACTTACGCCGCAG
AGGCGTCATCAACTTTGAGGTCTCTCTCATTCGGGATATCCGCGATCGAGAGTTCAAGATTTT
CTCGGATGCTGGCCGTGTTATGCGTCCTCTGTTCGCTGTGGAGCAGTCGGAC---AACTCGAC
AAAG---GGTCTTGAGAAGGGATCTTTATTGCTCACAAAGGAGCATATTCAAAAGTTGAGAGCT
GATGAGGAGTTATCCAAGGATGACCCAGATTATTTCGGCTGGGATGGCCTTCTCAGAGCGGG
CGCTATCGAATATCTTGACGCCGAAGAGGAAGAGACAGCCATGATTTGCATGACTCCAGAAG
ATCTAGAGGCTTATCGGCTGCAAAAAGCTGGATTAGACGTGCCGGAAGACGATGACGAGGCC
G---ACCCCAATAGGCGTCTGAAGACGAAACTGAACCCTACTACTCACATGTATACACATTGTGA
GATCCATCCGAGCATGCTCTTGGGGATCTGTGCGAGCATTATCCC??????????????????????
??
??
??
??
??
??
??
??
??
??
?????
>Distoseptispora_thailandica_MFLUCC_16_0270
ACCT--------TT-TTGTT-------CGTTGCTTTGGCGGG-CGGCTCC-AGGGCGGAGCCACTGCCCG
TTGGGTCGGGCGCCCGCCGAAGGA---CAAC-CGAAACTCGCT-AAGTTTTT-ATGGATATCAGA
GTAAAA------TAT-----CAAGCTAT-CAAAACTTTCAGCAACGGATCTCTTGGTTCTGGCATCGATG
AAGAACGCAGCGAAATGCGATAAGTAATGTGAATTGCAGAATTCAGTGAATCATCGAATCTTT
GAACGCACATTGCGCCCGCCAGCACTCTGGCGGGCATGCCTGTTCGAGCGTCATTTCAA-CC
CTCGGGCCC-TCGTT-------TGGTCCGGTGTTGGGGCGCTACGGGTCCG--TC-----------------GG
AC-CCGTAGGCCCTCAAAAACAGTGGCGGGCTCGCCAGGACT-CCGAGCGCAGTAGTTT-TC-
T--CTCGCTCAGGCGGCCT-GGCGTG--CT-TCGGCCGTTAAA---GACCCAAC------TGTAAT-----C
ACAG-AAGCCTTCCGAGTCCCC-TGGAAC------GGGGCGCC-AGAGAGGG-TGAGAGCCCCGT

ATGGTTGGACGCCGAACC-TCTGTAAAGCTCCTTCGACGAGTCGAGTAGTTTGGGAATGCTG
CTCAAAATGGGAGGTAAATTTCTTCTAAAG-CTAAATACCGGCCAGAGACCGATAGCGCACAA
GTAGAGTGATCGAAAGATGAAAAGCACTTTGAAAAGAGGGTCAAATAGCACGTGAAATTGCT
GAAAGGGAAGCGCTTGTGACCAGACTTGCGCCCGGTTGATCATCCAGCGTTCTCGCTGGTG
CACTCTGCCGGGCTCAGGCCAGCATCGGCTCTCCCAGGGGGATAAAGGTCGCGGAACGT
AGCTCTCTCCGGGGAGTGTTATAGCCCGTGG-CGGAATGCCCTTAGGGGGGGCCGAGGCCC
GCG-TTCG-CAAGGATGCTGGCGTAATGGTCACCAGCGACCCGTCTT???????????????????
???
???
???
???
???
???
???
???
???ACTCGTGAGCACGCTCTGC
TTGCCTACACCCTGGGTGTGAAGCAGCTCATCGTCGCCATCAACAAGATGGACACCACCAAG
TGGTCTGAGAGCCGTTACAACGAAATTATCAAGGAGACGTCCAACTTCATCAAGAAGGTCGG
CTACAACCCCAAGCAGGTCGCCTTCGTTCCCATCTCTGGCTTCAACGGTGACAACATGCTTA
CCCCCTCCTCCAACTGCCCCTGGTACAAGGGCTGGGAGAAGGAGGTC---AAGGGCGGCAAG
GCCAGTGGCAAGACCCTTCTCGAGGCCATCGATGCCATTGAGCCTCCCAAGCGTCCTACAG
ACAAGCCTCTGCGCCTCCCCCTCCAGGATGTCTACAAGATCGGCGGTATTGGAACTGTGCCC
GTCGGCCGTATCGAGACTGGTGTCCTCAAGCCCGGTATGGTCGTCACGTTCGCTCCCTCCA
ACGTCACCACTGAAGTCAAGTCCGTCGAGATGCACCACGAACAGCTGCAGGAGGGTGTCCC
CGGCGACAACGTCGGCTTCAACGTGAAGAACGTCTCCGTCAAGGAAATCCGCCGAGGCAAC
GTTGCTGGTGACTCCAAGAACGATCCTCCTGCTGGTGCCGCTTCCTTCACCGCCCAGGTCAT
CGTCCTCAACCACCCCGGTCAGGTCGGCGCTGGCTACGCTCCCGTCTTGGACTGCCACACT
GCCCACATCGCTTGCAAGTTCTCCGAGATTCTTGAGAAGATTGACCGCCGAACGGGCAAGTC
TGTCGAGAACAACCCCAAGTTCATCAAGTCTGGTGACGC
>Distoseptispora_thysanolaenae_KUN_HKAS_102247
ACCT--------ATC--GTT-------CGTTGCTCCGGCGGG-CGGCTCC-AGGGCGGAGCCACTGCCCG
TTGGGTCGGGCGCCCGCCGGAGGA---CAAC-CAAAACTCGTC-TGATCTC-GGGTATCGTCAG
AGTACTA----AGAAA-----AAAGCTAT-CAAAACTTTCAGCAACGGATCTCTTGGTTCTGGCATCGA
TGAAGAACGCAGCGAAATGCGATAAGTAATGTGAATTGCAGAATTCAGTGAATCATCGAATCT
TTGAACGCACATTGCGCCCGCCAGTACTCTGGCGGGCATGCCTGTTCGAGCGTCATTTCAA-
CCCTCGGGCC-CTCTTG-----TTTGGCCCGGTGTTGGGGTGCTACAGGTCCGCT------------------
-GGGACCTGTAGGCCCTCGAAATCAGTGGCGGGGTCGCC-TGGCCTCCGAGCGCAGTAGTT
C-TCTC--CTCGCTCAGGCGGTCC-GGCGTG--CC-TCGGCCGTTAAA---CCCCCA-C------TGTGT
-CT----ACAC-AAGCCTTCCGAGTCCCC-TGGAAC------GGGGCGCC-AGAGAGGG-TGAGAGCC
CCGTATGGTCGGACGCCGAGCC-TCTGTAAAGCTCCTTCGACGAGTCGAGTAGTTTGGGAAT
GCTGCTCAAAATGGGAGGTAAATTTCTTCTAAAG-CTAAATACCGGCCAGAGACCGATAGCGC
ACAAGTAGAGTGATCGAAAGATGAAAAGCACTTTGAAAAGAGGGTTAAATAGCACGTGAAATT
GCTGAAAGGGAAGCGCTTGTGACCAGACTTGCGCCCGGTTGATCATCCAGCGTTCTCGCTG
GTGCACTCTGCCGGGCCCAGGCCAGCATCGGCTCTCCCAGGGGGATAAAGGTCGCGGGAA

CGTAGCTCTCTCCGGGGAGTGTTATAGCCCGCGG-CGGAATACCCTTAGGGGGGGCCGAGG
CCCGCGTTC-G-CAAGGATGCTGGCGTAATGGTCACCAGCGACCCGTCTT??????????????
??
??
??
??
??
??
??
??
??ACACGTGAGCATGCTC
TGCTTGCCTACACCCTAGGTGTAAAGCAGCTCATCGTCGCCATCAACAAGATGGACACCACC
AAGTGGTCTGAGGATCGTTACAACGAAATCATCAAGGAGACGTCCAACTTCATCAAGAAGGT
CGGCTACAATCCGAAGCAGGTCGCCTTCGTCCCCATCTCGGGCTTCAACGGTGACAACATG
CTTGCCCCCTCCAGCAACTGCCCCTGGTACAAGGGTTGGGAGAAGGAGGTC---AAGGGCGG
CAAGGTCACTGGCAAGACCCTTCTCGAAGCCATCGATGCCATTGAGCCCCCCAAGCGTCCC
ACAGACAAGCCTCTGCGTCTTCCCCTTCAGGATGTCTACAAGATTGGTGGTATCGGAACTGTA
CCTGTCGGCCGTATCGAGACTGGTATCCTCAAGCCGGGCATGGTCGTCACGTTCGCTCCCT
CCAACGTCACTACTGAAGTGAAGTCCGTCGAGATGCACCACGAGCAGCTTCAAGAGGGCGT
TCCTGGCGACAACGTCGGCTTCAACGTGAAGAACGTCTCCGTCAAGGAAATCCGCCGAGGC
AACGTCGCTGGTGACTCCAAGAACGACCCTCCTGCTGGTGCTGCTTCCTTCACTGCTCAGG
TCATCGTCCTCAACCACCCGGGCCAGGTCGGTGCTGGTTACGCCCCTGTCTTGGATTGCCA
CACTGCCCACATCGCCTGCAAGTTCTCTGAGATCCTCGAGAAGATCGACCGCCGAACGGGC
AAGTCCGTCGAGAACAACCCCAAGTTCATCAAGTCTGGTGACGC
>Distoseptispora_thysanolaenae_KUN_HKAS_112710
ACCT--------ATC--GTT-------CGTTGCTCCGGCGGG-CGGCTCC-AGGGCGGAGCCACTGCCCG
TTGGGTCGGGCGCCCGCCGGAGGA---CAAC-CAAAACTCGTC-TGATCTC-GGGTATCGTCAG
AGTACTA----AGAAA-----AAAGCTAT-CAAAACTTTCAGCAACGGATCTCTTGGTTCTGGCATCGA
TGAAGAACGCAGCGAAATGCGATAAGTAATGTGAATTGCAGAATTCAGTGAATCATCGAATCT
TTGAACGCACATTGCGCCCGCCAGTACTCTGGCGGGCATGCCTGTTCGAGCGTCATTTCAA-
CCCTCGGGCC-CTCTTG-----TTTGGCCCGGTGTTGGGGTGCTACAGGTCCGCT------------------
-GGGACCTGTAGGCCCTCGAAATCAGTGGCGGGGTCGCC-TGGCCT---------------------------------
---GCCTTCCGAGTCCCC-TGGAAC------GGGGC
GCC-AGAGAGGG-TGAGAGCCCCGTATGGTCGGACGCCGAGCC-TCTGTAAAGCTCCTTCGA
CGAGTCGAGTAGTTTGGGAATGCTGCTCAAAATGGGAGGTAAATTTCTTCTAAAG-CTAAATAC
CGGCCAGAGACCGATAGCGCACAAGTAGAGTGATCGAAAGATGAAAAGCACTTTGAAAAGAG
GGTTAAATAGCACGTGAAATTGCTGAAAGGGAAGCGCTTGTGACCAGACTTGCGCCCGGTTG
ATCATCCAGCGTTCTCGCTGGTGCACTCTGCCGGGCCCAGGCCAGCATCGGCTCTCCCAGG
GGGATAAAGGTCGCGGGAACGTAGCTCTCTCCGGGGAGTGTTATAGCCCGCGG-CGGAATAC
CCTTAGGGGGGGCCGAGGCCCGCG-TTCG-CAAGGATGCTGGCGTAATGGTCACCAGCGAC
CCGTCTT???
??
??
??

??
??
??
??
??
???ACACGTGAGCATGCTCTGCTTGCCTACACCCTAGGTGTAAAGCAGCTCATCGTCGCCATC
AACAAGATGGACACCACCAAGTGGTCTGAGGATCGTTACAACGAAATCATCAAGGAGACGTC
CAACTTCATCAAGAAGGTCGGCTACAATCCGAAGCAGGTCGCCTTCGTCCCCATCTCGGGCT
TCAACGGTGACAACATGCTTGCCCCCTCCAGCAACTGCCCCTGGTACAAGGGTTGGGAGAA
GGAGGTC---AAGGGCGGCAAGGTCACTGGCAAGACCCTTCTCGAAGCCATCGATGCCATTG
AGCCCCCCAAGCGTCCCACAGACAAGCCTCTGCGTCTTCCCCTTCAGGATGTCTACAAGATT
GGTGGTATCGGAACTGTACCTGTCGGCCGTATCGAGACTGGTATCCTCAAGCCGGGCATGGT
CGTCACGTTCGCTCCCTCCAACGTCACTACTGAAGTGAAGTCTGTCGAGATGCACCACGAGC
AGCTTCAAGAGGGCGTTCCTGGCGACAACGTCGGCTTCAACGTGAAGAACGTCTCCGTCAA
GGAAATCCGCCGAGGCAACGTCGCTGGTGACTCCAAGAACGACCCTCCTGCTGGTGCTGCT
TCCTTCACTGCTCAGGTCATCGTCCTCAACCACCCGGGCCAGGTCGGTGCTGGTTACGCCC
CTGTCTTGGATTGCCACACTGCCCACATCGCCTGCAAGTTCTCTGAGATCCTCGAGAAGATC
GACCGCCGAACGGGCAAGTCCGTCGAGAACAACCCCAAGTTCATCAAGTCTGGTGACGC
>Distoseptispora_tropica_GZCC_22_0076
ACCA--------TTGATAACTAAACG-TCCTGTTCCGGCGGG-CGGCCCC-AGGGCGGGGCCACAG
CCCATTCAGTTGGGTGCCCGCCGGAGGA---CAGCACCAAACCATTCTGTAAATCGTGGCA-TC
TCCGGGTACAAT-----TGC-------AAGCAT-AAAAACTTTCAGCAACGGATCTCTTGGCTCTGGCAT
CGATGAAGAACGCAGCGAAATGCGATAAGTAATGCGAATTGCAGAATTCAGCGAGTCATCGAA
TCTTTGAACGCACATTGCGCCCGCCAGCACTCTGGCGGGCATGCCTGTCCGAGCGTCATTT
CAC-CCCTCAGGCCCCCCG---------CGGCCTGGTGTTGGGGCGCTACGGTGCGCCGGCCC----
ACAGCGGGCCGGCGGCGCCGTAGGCCCTGAAATACAGTGGCGGCCTCCCCATGTTT-CCGG
GCGCAGTAGTTC-TCCT--CTCGCCCGGGAGGCATGGTGGTG--CG-CTGGCCGTTAAGCGACC
CCCCTC------TGTAATAC-------AGAAGCCTACCGAGTCCCC-TGGAAT------GGGGCGCC-ACAG
AGGG-TGAGAGCCCCGTACGGTAGGACGCCCAGCC-CGTGTAAAGCTCCTTCGACGAGTCGA
GTAGTTTGGGAATGCTGCTCAAAATGGGAGGTAAATTTCTCCTAAAG-CTAAATACTGGCCAGA
GACCGATAGCGCACAAGTAGAGTGATCGAAAGATGAAAAGCACTTTGAAAAGAGGGTTAAATA
GCACGTGAAATTGCTGAAAGGGAAGCGCTTGCGACCAGACTTGCGCCCGGCTGATCAGCCA
GCGTTCTCGCTGGTGCACTCTGCCGGGCTCAGGCCAGCATCGGCTCCTCCAGGGGGACAA
AACCCGCCGGAACGTGGCTCCCTTAGGGGAGTGTTATAGCCGGCGG-CAGAATACCCCTCG
GGGGGGCCGAGGCCCGCGCAACTGCACGGATGCTGGCGTAATGGTCGCCAGCGACCCGTC
TTTATCACTCATGTGTTACGTGAGCGTGGGCACGCCAGCCGAGCCGATCGTCGAGTTTATGA
TTGGGAGAAATATGGAGGTGCTGGAAGAGTATGAACCCTTGAGGTATCCTAATGCAACCAAGA
TCTTTGTCAATGGCACATGGGTAGGTGTTCACCAGGATCCGAAACACTTGGTAAGCCTGGTG
CAGGAGCTGCGGCGAAAGAGCGTCATCAACTTCGAGGTTTCTCTCGTACGCGATATCCGGGA
TCGGGAATTCAAGATCTTCTCCGATGCTGGTCGTGTCATGAGGCCGCTCTTCGCAGTAGAAC
AGTCGGAT---GACTCAAAAGAC---GGCTTAGAGAAGGGCTCATTACGACTAACCAAAGAACAC
ATTCAGAGGCTCAGAACGGACGAGGAGCTGGACAAGGATGATCCAGACTACTTCGGCTGGG
AGGGCCTGCTCAAAGCCGGTGCCATCGAATACCTAGACGCGGAAGAAGAGGAGACGGCCAT
GATTTGCATGACACCGGAGGACTTGGAGGCTTATAGACTGCAGAAGGCTGGCCTGGACGTA

CCGGAAGACGACGAAGACGCAG---ATCCGAACAGGCGCCTCAAGACGAAGATGAACCCCAC
TACTCACATGTACACACATTGTGAGATCCACCCCAGTATGCTTCTGGGAATCTGCGCCAGCAT
CATTCCACTCGTGAGCACGCTCTGCTTGCCTTCACGTTGGGTGTCAAGCAACTTATCGTCGC
CATCAACAAGATGGACACGGCCAAGTGGAGTGAGGCACGTTATCAGGAAATCATCAAGGAGA
CGTCCAACTTCATCAAGAAGGTCGGCTACAACCCCAAGGAGGTTCCCTTCGTCCCCATCTCG
GGTTTCAACGGCGACAACATGCTGGATGTCTCCACAAATTGCCCCTGGTACAAGGGTTGGGA
GAAGGAAATC---AAGGGTACAAAGTCCGATGGCAAGACCCTCTTCGAGGCTATTGATGCCATC
CAACCCCCTAAGCGTCCCACTGACAAGCCCCTTCGACTTCCCCTCCAGGATGTCTACAAGAT
CGGTGGTATCGGAACTGTCCCTGTCGGCCGTATTGAGACTGGTACCCTCAAGCCCGGTATGG
TCGTCACTTTCGCTCCCTCCAATGTCACCACTGAAGTCAAGTCTGTTGAGATGCACCACCAG
CAGCTCCAACAGGGTCTCCCTGGCGACAACGTCGGCTTCAACGTGAAGAATGTCTCCGTCA
AGGAAATCCGCCGTGGCAACGTTGCCGGTGACTCCAAGGACGACCCTCCACAAGGTGCTG
CTTCGTTTACCGCTCAGGTCATTGTCCTCAACCATCCCGGTCAGATCGGTGCTGGTTACGCT
CCTGTCCTGGATTGCCACACTGCCCACATCGCTTGCAAGTTCGCCGAGATTCAGGAGAAGAT
CGACCGCCGAACAGGCAAGTCCGTTGAAAACAGCCCCAAGTTCGTCAAGTCTGGTGACGC
>Distoseptispora_verrucosa_GZCC_20_0434
ACCT--------ATCGTCAAA------TGTTGCTTCGGCGGG-CGGCCCC-TGGGAGGGGCCAAGGCCC
GTTGGGTTGGGTGCCCGCCGGAGGA---CAGC-CGAAAACTCTTTT----ATCAGGTACTGTCTGA
GTATA------CTATA--AAAA--CTAT-AAAAACTTTCAACAACGGATCTCTTGGTTCTGGCATCGATG
AAGAACGCAGCGAAATGCGATAAGTAATGTGAATTGCAGAATTCAGTGAATCATCGAATCTTT
GAACGCACATTGCGCCCGCCAGCACTCTGGCGGGCATGCCTGTTCGAGCGTCATTTCA-ACC
CTCAGGCCC-TA-------GTTTGGCCTGGTGTTGGGGCACTGCCGTCCCGCCC---------------GTAC
GGGGCGCGCAGGCCCTGAAAACCAGTGGCGGGCTCGCTAGCAC-TCCGGGCGTAGTAGA-A
TCAT-ATCTCGTCTAGGTGGTCT-GGCGCG--GT-CTTGCCGTCAAAAC-CCCCC---------ATTTTTA
C-------ACAAGCCTTCTAAGTCCCC-TAGAAC------GGGGCGCC-ACAGAGGG-TGAGAGCCCCG
TACAGTTGGACACCGAACC-TTTGTAAAGCTCCTTCGACGAGTCGAGTAGTTTGGGAATGCTG
CTCAAAATGGGAGGTATATTTCTTCTAAAG-CTAAATACCGGCCAGAGACCGATAGCGCACAAG
TAGAGTGATCGAAAGATGAAAAGCACTTTGAAAAGAGGGTTAAATAGCACGTGAAATTGTTGA
AAGGGAAGCGCTTGTGACCAGACTTGTGCCCGGTGAATCATCCAGCGTTCTCGCTGGTGCA
CTTTGCCGGGCTCAGGCCAGCATCGGTTCTCCCAGGGGGATAAAGGTCTCGGGAACGTAGC
TCTCTTCGGGGAGTGTTATAGCCCGTGG-CATAATACCTTTGGG-GGGACCGAGGACCGCGCT
CTG-CAAGGATGCTGGCGTAATGGTCATCAGCGACCCGTCTTTGTCGCTCATGTGTTATGTCA
GCGTTGGCACACCGGCAGAACCGATTATCGAGTTTATGATCGCGAGGAATATGGAGGTCCTC
GAGGAATATGAGCCTTTGCGCTATCCCAACGCAACTAAGATATTTGTCAACGGGACTTGGGTG
GGCGTTCACCAGGACCCCAAACACTTGGTTGGGCTGGTACAGGAGTTGCGCCAGAGGGGA
GTTATCAACTTTGAGGTTTCCCTTGTGCGTGATATCCGAGACAGGGAGTTCAAGATCTTCTCC
GATGCTGGTCGGGTGATGCGACCGCTTTTCGCCGTGGAGCAGGAGGAC---AATTCTAAGTCG
---GGGCTCCCGAAGGGATCTTTGAGGCTCACCAAAGAGCATATCCAGAGGCTCAGAGCGGA
CGAAGACTTGTCCAAAGAAGATCCGGAATACTTCGGATGGGACGGCATTGTTGAGGCTGGC
GCAGTCGAATATCTCGACGCGGAAGAAGAGGAAACGGCTATGATATGCATGACGCCGGAAGA
CCTGGAGGCTTACAGACTCCAAAAAGCCGGGTTGGGCATGGCAGAAGACGACGAAGAAGC
GG---ACCCAAACAGACGCCTGAAAACGAAATTAAACCCTACCACTCACATGTACACACACTGC
GAGATTCATCCCAGTATGCTATTGGGAATCTGCGCAAGCATCATACCACTCGTGAGCACGCCC
TGCTTGCCTACACCCTGGGTGTCAAGCAGCTCATCTGCGCCATCAACAAGATGGACACCACC

AAGTGGTCAGAGGACCGTTTCAACGAAATCATCAAGGAGACGTCCAACTTCATCAAGAAGGT
CGGCTACAACCCCAAGCAGGTCGCCTTCGTCCCTATCTCTGGCTTCCACGGCGACAACATGT
TGGCTGCCTCCAGCAACTGCCCCTGGTACAAGGGCTGGGAGAAGGAGGGTGCCAAGGGTG
CCAAGTCTTCTGGTAAGACCCTGCTCGAGGCCATCGACGCCATCGAGGAACCCAAGCGTCC
TACCGACAAGCCTCTGCGTCTCCCGCTCCAGGATGTCTACAAGATCGGTGGTATCGGAACTG
TCCCTGTCGGCCGTATCGAGACTGGTATCCTCAAGCCTGGTATGGTCGTCACTTTCGCTCCC
TCCAACGTCACCACTGAAGTCAAGTCCGTCGAGATGCACCACGAGCAGCTTACTGAGGGTG
TTCCCGGCGACAACGTCGGCTTCAACGTGAAGAACGTCTCCGTCAAGGAAATCCGCCGTGG
AAACGTCGCTGGTGACTCCAAGAACGACCCCCCCTCTGGCTGCGCCAACTTCACTGCCCAG
GTCATTGTCCTCAACCACCCTGGTCAGGTCGGCCCTGGTTACGCTCCTGTCTTGGATTGCCA
CACCGCACACATTGCCTGCAAGTTCTCTGAGATCACCGAGAAGATCGACCGCCGTACCGGC
AAGTCAGTTGAGAACAACCCCAAGTTCATCAAGTCTGGTGACGC
>Distoseptispora_wuzhishanensis_GZCC_22_0077
ACCT--------TT-ATATT-------CGTTGCTTTGGCGGG-CGGCTCC-AGGGCGGAGCCACTGCTCGT
TGGGTCGGGCGCCCGCCGAAGGA---CAAC-CAAAACTCATC-GAATCTGT-GCGGATTTCAGA
GTAAAAAAA---TAT-----CAAGCTAT-CAAAACTTTCAGCAACGGATCTCTTGGTTCTGGCATCGAT
GAAGAACGCAGCGAAATGCGATAAGTAATGTGAATTGCAGAATTCAGTGAATCATCGAATCTT
TGAACGCACATTGCGCCCGCCAGCACTCTGGCGGGCATGCCTGTTCGAGCGTCATTTCAA-C
CCTCGGGCCC-TCGTT-------TGGCCTGGTGTTGGGGCGCTACGGGTCTC-ACC------------------G
GAC-CTGTAGGCCCCCAAAATCAGTGGCGGGCTCGCCAGGACT-CCGAGCGTAGTAGTTC-TC
CT--CTCGCTCAGGTGGTCT-GGCGTG--CT-TCGGCCGTTAAA---TACCCAGC------GGTAAT-CG
C---TAC-AAGCCTTCCGAGTCCCC-TGGAAC------GGGGCGCC-AGAGAGGG-TGAGAGCCCCG
TATGGTTGGACGCCGAACC-TCTGTAAAGCTCCTTCGACGAGTCGAGTAGTTTGGGAATGCTG
CTCAAAATGGGAGGTAAATTTCTTCTAAAG-CTAAATACCGGCCAGAGACCGATAGCGCACAA
GTAGAGTGATCGAAAGATGAAAAGCACTTTGAAAAGAGGGTCAAATAGCACGTGAAATTGCT
GAAAGGGAAGCGCTTGTGACCAGACTTGCGCCCGGTTGATCATCCAGCGTTCTCGCTGGTG
CACTCTGCCGGGCTCAGGCCAGCATCGGCTCTCCCAGGGGGATAAAGGTCGCGGGAACGT
AGCTCTCTCCGGGGAGTGTTATAGCCCGTGG-CGGAATGCCCTTAGGGGGGGCCGAGGCCC
GCGTTC-G-CAAGGATGCTGGCGTAATGGTCACCAGCGACCCGTCTT???????????????????
???
???
???
???
???
???
???
???
???ACTCGTGAGCACGCTCTGC
TCGCCTACACACTTGGTGTGAAGCAGCTTATCGTCGCCATCAACAAGATGGACACCACCAAG
TGGTCCGAGAGCCGTTACAACGAAATTATCAAGGAGACGTCCAACTTCATCAAGAAGGTCGG
CTACAACCCCAAGCAGGTCGCCTTCGTTCCCATCTCCGGCTTCAATGGCGACAACATGCTTG
CCCCCTCCTCCAACTGCCCCTGGTACAAGGGTTGGGAGAAGGAGGTC---AAGGGTGGCAAG
GCCTCTGGCAAGACCCTCTTGGAGGCCATCGATGCCATTGAGCCTCCCAAGCGTCCCACAG
ACAAGCCTCTCCGTCTCCCCCTTCAGGATGTCTACAAGATTGGTGGTATCGGAACTGTGCCT

GTCGGCCGTATCGAGACTGGTGTCCTCAAGCCCGGTATGGTCGTCACATTCGCTCCCTCCAA
CGTCACCACTGAAGTCAAGTCCGTCGAGATGCACCACGAGCAGCTGCAGGAGGGTGTCCC
CGGCGACAACGTCGGCTTCAACGTGAAGAACGTCTCCGTCAAGGAAATCCGCCGAGGCAAC
GTCGCTGGTGACTCCAAGAACGACCCTCCTGCCGGTGCCGCTTCCTTCACCGCTCAGGTCA
TCGTCCTCAACCACCCCGGTCAGGTCGGTGCTGGCTACGCTCCCGTCTTGGATTGCCACAC
TGCCCACATCGCTTGCAAGTTCTCTGAGATCCTTGAGAAGATCGACCGCCGAACGGGCAAGT
CTGTTGAGAACAACCCCAAGTTCATCAAGTCTGGTGACGC
>Distoseptispora_xishuangbannaensis_KUMCC_17_0290
ACCT--------CT-ATATT-------CGTTGCTTTGGCGGG-CGGTTCC-AGGGCGGAACCACTGCTCGT
TGGGTCGGGCGCCCGCCGGAGGA---CAAC-CAAAACTCGTT-AAATCTTT-GCGGATCTCAGA
GTAAAAA-----TAT-----TAAGCTAT-CAAAACTTTCAGCAACGGATCTCTTGGTTCTGGCATCGATG
AAGAACGCAGCGAAATGCGATAAGTAATGTGAATTGCAGAATTCAGTGAATCATCGAATCTTT
GAACGCACATTGCGCCCGCCAGCACTCTGGCGGGCATGCCTGTTCGAGCGTCATTTCAA-CC
CTCGGGCCC-TCGCT-------TGGCCCGGTGTTGGGGCGCTACGGGTCCC-ATC-----------------G
GAC-CTGTAGGCCCTCAAAACCAGTGGCGGGCTCGCCAGGACT-CCGAGCGCAGTAGTTC-T
CCT--CTCGCTCAGGCGGCCT-GGCGTG--CT-TCGGCCGTTAAA---TACCCAGC------GGTAATT
CG----CAC-AAGCTTGCGACGGCCTC-GAGTACCTGACCGGAGCGCC-AGAGAGGGGTGAGA
GCCC-GTATG------TGACGCGAC-TCTGTAAG---CTCTCGACGAGTCGAGTAGTTTGGGAATGCT
GCTCAAAATGGGAGGTAAATTTCTTCTAAAG-CTAAATACCGGCCAGAGACCGATAGCGCACA
AGTAGAGTGATCGAAAGATGAAAAGCACTTTGAAA-GAGGGTCAAATAGCACGTGAAATTGCT
GAAAGGGAAGCGCTTGTGACCAGACTTGCGCCCGGTTGATCATCCAGCGTTCTCGCTGGTG
CACTCTGCCGGGCTCAGGCCAGCATCGGCTCTCCCAGGGGGATAAAGGTCGCGGGAACGT
AGCTCTCTCCGGGGAGTGTTATAGCCCGTGG-CGGAATGCCCTTAGGGGGGGCCGAGGCCC
GCGTTC-G-CAAGGATGCTGGCGTAATGGTCACCAGCGACCCGTCTTTATCGCTCATGTGTTAT
GTGAGCGTGGGAACGCCCGCAGAGCCAATCGTCGAGTTCATGATTGCTAGGAACATGGAAG
TACTCGAGGAATATGAGCCCTTGAGGTACCCTAATGCGACCAAAATCTTCGTCAATGGCACGT
GGGTTGGTGTTCACCAAGACCCGAAGCATCTTGTGAGCTTGGTTCAGGACTTACGCCGCAG
AGGCGTCATCAACTTTGAGGTCTCTCTCATTCGGGATATCCGCGATCGAGAGTTCAAGATTTT
CTCGGATGCTGGTCGTGTTATGCGTCCTCTGTTCGCTGTGGAGCAGTCGGAC---AACTCGAC
AAAG---GGCCTTGAAAAGGGGTCTTTATTGCTCACAAAGGAGCATATTCAAAAATTGAGAGCT
GATGAGGAGTTATCCAAGGATGACCCAGACTATTTCGGCTGGGATGGCCTTCTCAGAGCGGG
CGCTATTGAATATCTTGACGCCGAAGAGGAAGAGACAGCCATGATCTGCATGACTCCAGAAG
ATCTAGAGGCTTATCGGCTGCAGAAAGCTGGATTAGACGTGCCGGAAGACGACGACGAGGC
CG---ACCCCAATAGGCGTCTGAAGACGAAACTGAACCCCACGACTCACATGTACACACATTGT
GAGATCCATCCGAGCATGCTCTTAGGGATCTGTGCGAGCATTATCCCACTCGTGAGCACGCT
CTGCTCGCCTACACGCTTGGTGTGAAGCAGCTCATCGTCGCCATCAACAAGATGGACACCAC
CAAGTGGTCCGAGAGCCGTTACAACGAAATTATCAAGGAGACGTCCAACTTCATCAAGAAGG
TTGGCTACAACCCCAAGCAGGTCGCCTTCGTTCCCATCTCCGGCTTCAACGGCGACAACATG
CTTACCCCCTCCTCCAACTGCCCCTGGTACAAGGGTTGGGAGAAGGAGGTC---AAGGGTGG
CAAGGCCTCTGGCAAGACCCTCTTGGAGGCTATCGATGCCATTGAGCCTCCCAAGCGTCCC
ACAGACAAGCCTCTCCGTCTCCCCCTTCAGGATGTCTACAAGATCGGTGGTATCGGAACTGT
ACCTGTCGGCCGTATCGAGACTGGTATCCTCAAGCCTGGCATGGTCGTCACGTTCGCTCCCT
CCAACGTCACCACTGAAGTCAAGTCCGTCGAGATGCACCACGAGCAGCTGCAGGAGGGTGT
CCCTGGCGACAACGTCGGCTTCAACGTGAAGAACGTCTCCGTCAAGGAAATCCGCCGAGGC

AACGTCGCTGGTGATTCCAAGAACGACCCTCCTGCTGGTGCCGCTTCCTTCACCGCTCAGG
TCATCGTCCTCAACCACCCCGGTCAGGTCGGTGCTGGTTACGCTCCCGTCTTGGATTGCCAC
ACTGCCCACATCGCTTGCAAGTTCTCTGAGATCCTTGAGAAGATCGACCGCCGAACGGGCAA
GTCTGTTGAGAACAACCCCAAGTTCATCAAGTCTGGTGACGC
>Distoseptispora_yongxiuensis_JAUCC_4725
ACCT--------ATCGTCAAA------TGTTGCTTCGGCGGG-CGGCCCC-TGGGAGGGGCCAAGGCCC
GTTGGGTCGGGTGCCCGCCGGAGGA---CAGC-CGAAAACTCTTTTT---ATCAGGTACTGTCTG
AGTAAA------CTATA--AAAA--CTAT-AAAAACTTTCAACAACGGATCTCTTGGTTCTGGCATCGAT
GAAGAACGCAGCGAAATGCGATAAGTAATGTGAATTGCAGAATTCAGTGAATCATCGAATCTT
TGAACGCACATTGCGCCCGCCAGCACTCTGGCGGGCATGCCTGTTCGAGCGTCATTTCA-AC
CCTCAGGCCC-TA-------GTTTGGCCTGGTGTTGGGGCACTGCCGTCCCGCCC--------------GTA
CGGGGCGCGCAGGCCCTGAAAACCAGTGGCGGGCTCGCTAGCAC-TCCGGGCGTAGTAGA-
ATCAT-ATCTCGTCTAGGTGGTCT-GGCGCG--GT-CTTGCCGTCAAAAC-CCCCCC--------A-TTTT
AT-------ACAAGCCTTCTAAGTCCCC-TAGAAC------GGGGCGCC-ACAGAGGG-TGAGAGCCCC
GTACAGTTGGACACCGAACC-TTTGTAAAGCTCCTTCGACGAGTCGAGTAGTTTGGGAATGCT
GCTCAAAATGGGAGGTATATTTCTTCTAAAG-CTAAATACCGGCCAGAGACCGATAGCGCACAA
GTAGAGTGATCGAAAGATGAAAAGCACTTTGAAAAGAGGGTTAAATAGCACGTGAAATTGTTG
AAAGGGAAGCGCTTGTGACCAGACTTGCGCCCGGTGAATCATCCAGCGTTCTCGCTGGTGC
ACTTTGCCGGGCTCAGGCCAGCATCGGTTCTCCCAGGGGGATAAAGGTCTCGGGAACGTAG
CTCTCTTCGGGGAGTGTTATAGCCCGTGG-CATAATACC-TTTGGGGGGACCGAGGACCGCG
CTCTG-CAAGGATGCTGGCGTAATGGTCATCAGCGACCCGTCTT??????????????????????
??
??
??
??
??
??
??
??
???ACTCGTGAGCACGCCCTGCTTG
CCTACACCCTGGGTGTCAAGCAGCTCATCTGCGCCATCAACAAGATGGACACCACCAAGTG
GTCAGAGGACCGTTTCAACGAAATCATCAAGGAGACGTCCAACTTCATCAAGAAGGTCGGCT
ACAACCCCAAGCAGGTCGCCTTCGTCCCTATCTCTGGCTTCCACGGCGACAACATGTTGGCT
GCCTCCACCAACTGCCCCTGGTACAAGGGCTGGGAGAAGGAGGGCGCCAAGGGTGCCAAG
TCTTCTGGTAAGACCCTGCTCGAGGCCATCGACGCCATCGAGCAACCCAAGCGTCCTACCG
ACAAGCCTCTGCGTCTCCCGCTCCAGGATGTCTACAAGATCGGTGGTATCGGAACCGTCCCT
GTCGGCCGTATCGAGACTGGTATCCTCAAGCCCGGTATGGTCGTTACTTTCGCTCCCTCCAA
CGTCACCACTGAAGTCAAGTCCGTCGAGATGCACCACGAGCAGCTTACTGAGGGTGTTCCC
GGCGACAACGTCGGCTTCAACGTGAAGAACGTCTCCGTCAAGGAAATCCGCCGTGGAAACG
TCGCTGGTGACTCCAAGAACGACCCTCCTTCTGGCTGCGCCAACTTCACTGCTCAGGTCATT
GTCCTCAACCACCCTGGTCAGGTCGGCCCTGGTTACGCTCCCGTCTTGGATTGCCACACCG
CCCACATCGCCTGCAAGTTCTCTGAGATCACCGAGAAGATCGACCGCCGTACCGGCAAGTC
AGTTGAGAACAACCCCAAGTTCATCAAGTCTGGTGACGC
>Distoseptispora_yongxiuensis_JAUCC_4726

ACCT--------ATCGTCAAA------TGTTGCTTCGGCGGG-CGGCCCC-TGGGAGGGGCCAAGGCCC
GTTGGGTCGGGTGCCCGCCGGAGGA---CAGC-CGAAAACTCTTTTT---ATCAGGTACTGTCTG
AGTAAA------CTATA--AAAA--CTAT-AAAAACTTTCAACAACGGATCTCTTGGTTCTGGCATCGAT
GAAGAACGCAGCGAAATGCGATAAGTAATGTGAATTGCAGAATTCAGTGAATCATCGAATCTT
TGAACGCACATTGCGCCCGCCAGCACTCTGGCGGGCATGCCTGTTCGAGCGTCATTTCA-AC
CCTCAGGCCC-TA-------GTTTGGCCTGGTGTTGGGGCACTGCCGTCCCGCCC--------------GTA
CGGGGCGCGCAGGCCCTGAAAACCAGTGGCGGGCTCGCTAGCAC-TCCGGGCGTAGTAGA-
ATCAT-ATCTCGTCTAGGTGGTCT-GGCGCG--GT-CTTGCCGTCAAAAC-CCCCCC--------A-TTTT
AT-------ACAAGCCTTCTAAGTCCCC-TAGAAC------GGGGCGCC-ACAGAGGG-TGAGAGCCCC
GTACAGTTGGACACCGAACC-TTTGTAAAGCTCCTTCGACGAGTCGAGTAGTTTGGGAATGCT
GCTCAAAATGGGAGGTATATTTCTTCTAAAG-CTAAATACCGGCCAGAGACCGATAGCGCACAA
GTAGAGTGATCGAAAGATGAAAAGCACTTTGAAAAGAGGGTTAAATAGCACGTGAAATTGTTG
AAAGGGAAGCGCTTGTGACCAGACTTGCGCCCGGTGAATCATCCAGCGTTCTCGCTGGTGC
ACTTTGCCGGGCTCAGGCCAGCATCGGTTCTCCCAGGGGGATAAAGGTCTCGGGAACGTAG
CTCTCTTCGGGGAGTGTTATAGCCCGTGG-CATAATACC-TTTGGGGGGACCGAGGACCGCG
CTCTG-CAAGGATGCTGGCGTAATGGTCATCAGCGACCCGTCTT???????????????????????
??
??
??
??
??
??
??
??
??ACTCGTGAGCACGCCCTGCTTG
CCTACACCCTGGGTGTCAAGCAGCTCATCTGCGCCATCAACAAGATGGACACCACCAAGTG
GTCAGAGGACCGTTTCAACGAAATCATCAAGGAGACGTCCAACTTCATCAAGAAGGTCGGCT
ACAACCCCAAGCAGGTCGCCTTCGTCCCTATCTCTGGCTTCCACGGCGACAACATGTTGGCT
GCCTCCACCAACTGCCCCTGGTACAAGGGCTGGGAGAAGGAGGGCGCCAAGGGTGCCAAG
TCTTCTGGTAAGACCCTGCTCGAGGCCATCGACGCCATCGAGCAACCCAAGCGTCCTACCG
ACAAGCCTCTGCGTCTCCCGCTCCAGGATGTCTACAAGATCGGTGGTATCGGAACCGTCCCT
GTCGGCCGTATCGAGACTGGTATCCTCAAGCCCGGTATGGTCGTTACTTTCGCTCCCTCCAA
CGTCACCACTGAAGTCAAGTCCGTCGAGATGCACCACGAGCAGCTTACTGAGGGTGTTCCC
GGCGACAACGTCGGCTTCAACGTGAAGAACGTCTCCGTCAAGGAAATCCGCCGTGGAAACG
TCGCTGGTGACTCCAAGAACGACCCTCCTTCTGGCTGCGCCAACTTCACTGCTCAGGTCATT
GTCCTCAACCACCCTGGTCAGGTCGGCCCTGGTTACGCTCCCGTCTTGGATTGCCACACCG
CCCACATCGCCTGCAAGTTCTCTGAGATCACCGAGAAGATCGACCGCCGTACCGGCAAGTC
AGTTGAGAACAACCCCAAGTTCATCAAGTCTGGTGACGC
>Distoseptispora_yunjushanensis_JAUCC_4723
ACCT--------ATC--GTT-------CGTTGCTCCGGCGGG-CGGCTCC-AGGGCGGAGCCACTGCCAG
TTGGGTCGGGCGCCCGCCGGAGGA---CAAC-CAAAACTAGCC-TATTCTC-GGGTAACGTCAG
AGTAATA----AAAAA----AAAAGCTAT-CAAAACTTTCAGCAACGGATCTCTTGGTTCTGGCATCG
ATGAAGAACGCAGCGAAATGCGATAAGTAATGTGAATTGCAGAATTCAGTGAATCATCGAATCT
TTGAACGCACATTGCGCCCGCCAGCACTCTGGCGGGCATGCCTGTTCGAGCGTCATTTCAA-

CCCTCGGGCCACCCCCT----GTGTGGCCCGGTGTTGGGGCGCTACCTTCCCCCC---C-----------
-CGGGGGACCTGGTAGGCCCTGAAAACCAGTGGCGGGCTCGCCATGGCCTCCGAGCGCAG
TAGTTC-TCTC--CTCGCTCAGGCGGTCT-GGCGCG--CC-TCGGCCGTTAAA---CACACG-C------
TGTATCAC----GCAG-AAGCCTTCCGAGTCCCC-TGGAAC------GGGGCGCC-AGAGAGGG-TGA
GAGCCCCGTACGGTCGGACGCCGAACC-TCTGTAAAGCTCCTTCGACGAGTCGAGTAGTTTG
GGAATGCTGCTCAAAATGGGAGGTAAATTTCTTCTAAAG-CTAAATACTGGCCAGAGACCGATA
GCGCACAAGTAGAGTGATCGAAAGATGAAAAGCACTTTGAAAAGAGGGTTAAATAGCACGTG
AAATTGCTGAAAGGGAAGCGCTTGTGACCAGACTTGCGCCCGGTTGATCATCCAGCGTTCTC
GCTGGTGCACTCTGCCGGGCTCAGGCCAGCATCGGCTCTCCCAGGGGGATAAAGGTCGCG
GGAACGTAGCTCTCTCCGGGGAGTGTTATAGCCCGTGG-CGGAATACCCTTAGGGGGGTC
GAGGCCCGCG-TTCG-CAAGGATGCTGGCGTAATGGTCACCAGCGACCCGTCTT??????????
???
???
???
???
???
???
???
???
??ACTCGTGAGCAC
GCTCTGCTTGCCTACACCCTGGGTGTGAAGCAGCTCATCGTCGCCATCAACAAGATGGACAC
CACCAAGTGGTCTGAGGATCGTTACAACGAAATCATCAAGGAGACGTCCAACTTCATCAAGA
AGGTCGGCTACAACCCCAAGCAGGTCGCTTTCGTTCCCATCTCCGGCTTCAACGGCGACAA
CATGCTTGCCCCCTCCTCCAACTGCCCCTGGTACAAGGGCTGGGAGAAGGAGATC---AAGG
GTGGCAAGGTCAGTGGCAAGACGCTTCTCGAGGCCATCGATGCCATTGAGCCTCCCAAGCG
TCCCACAGACAAGCCTCTGCGTCTTCCCCTCCAGGATGTCTACAAGATCGGTGGTATCGGAA
CCGTACCTGTCGGCCGTATCGAGACTGGTATCCTCAAGCCCGGTATGGTCGTCACGTTCGCT
CCTTCCAACGTCACCACTGAAGTCAAGTCTGTCGAGATGCACCACGAGCAGCTTCAAGAGG
GCATGCCTGGCGACAACGTCGGCTTCAACGTGAAGAACGTCTCTGTCAAGGAAATCCGCCG
TGGCAACGTCGCTGGTGACTCCAAGAACGATCCTCCTGCTGGTGCTGCTTCCTTCACCGCC
CAGGTCATCGTCCTCAACCACCCTGGTCAGGTTGGTGCTGGCTACGCTCCCGTCCTGGACT
GCCACACTGCCCACATCGCCTGCAAGTTCTCCGAGATCCTCGAGAAGATCGACCGCCGAAC
GGGCAAGTCCGTCGAGAACAACCCCAAGTTCATCAAGTCTGGTGACGC
>Distoseptispora_yunjushanensis_JAUCC_4724
ACCT--------ATC--GTT-------CGTTGCTCCGGCGGG-CGGCTCC-AGGGCGGAGCCACTGCCAG
TTGGGTCGGGCGCCCGCCGGAGGA---CAAC-CAAAACTAGCC-TATTCTC-GGGTAACGTCAG
AGTAATA----AAAAA----AAAAGCTAT-CAAAACTTTCAGCAACGGATCTCTTGGTTCTGGCATCG
ATGAAGAACGCAGCGAAATGCGATAAGTAATGTGAATTGCAGAATTCAGTGAATCATCGAATCT
TTGAACGCACATTGCGCCCGCCAGCACTCTGGCGGGCATGCCTGTTCGAGCGTCATTTCAA-
CCCTCGGGCCACCCCCT----GTGTGGCCCGGTGTTGGGGCGCTACCTTCCCCCC---C-----------
-CGGGGGACCTGGTAGGCCCTGAAAACCAGTGGCGGGCTCGCCATGGCCTCCGAGCGCAG
TAGTTC-TCTC--CTCGCTCAGGCGGTCT-GGCGCG--CC-TCGGCCGTTAAA---CACACG-C------
TGTATCAC----GCAG-AAGCCTTCCGAGTCCCC-TGGAAC------GGGGCGCC-AGAGAGGG-TGA
GAGCCCCGTACGGTCGGACGCCGAACC-TCTGTAAAGCTCCTTCGACGAGTCGAGTAGTTTG

GGAATGCTGCTCAAAATGGGAGGTAAATTTCTTCTAAAG-CTAAATACTGGCCAGAGACCGATA
GCGCACAAGTAGAGTGATCGAAAGATGAAAAGCACTTTGAAAAGAGGGTTAAATAGCACGTG
AAATTGCTGAAAGGGAAGCGCTTGTGACCAGACTTGCGCCCGGTTGATCATCCAGCGTTCTC
GCTGGTGCACTCTGCCGGGCTCAGGCCAGCATCGGCTCTCCCAGGGGGATAAAGGTCGCG
GGAACGTAGCTCTCTCCGGGGAGTGTTATAGCCCGTGG-CGGAATACCCTTAGGGGGGGTC
GAGGCCCGCG-TTCG-CAAGGATGCTGGCGTAATGGTCACCAGCGACCCGTCTT??????????
??
??
??
??
??
??
??
??
???ACTCGTGAGCAC
GCTCTGCTTGCCTACACCCTGGGTGTGAAGCAGCTCATCGTCGCCATCAACAAGATGGACAC
CACCAAGTGGTCTGAGGATCGTTACAACGAAATCATCAAGGAGACGTCCAACTTCATCAAGA
AGGTCGGCTACAACCCCAAGCAGGTCGCTTTCGTTCCCATCTCCGGCTTCAACGGCGACAA
CATGCTTGCCCCCTCCTCCAACTGCCCCTGGTACAAGGGCTGGGAGAAGGAGATC---AAGG
GTGGCAAGGTCAGTGGCAAGACGCTTCTCGAGGCCATCGATGCCATTGAGCCTCCCAAGCG
TCCCACAGACAAGCCTCTGCGTCTTCCCCTCCAGGATGTCTACAAGATCGGTGGTATCGGAA
CCGTACCTGTCGGCCGTATCGAGACTGGTATCCTCAAGCCCGGTATGGTCGTCACGTTCGCT
CCTTCCAACGTCACCACTGAAGTCAAGTCTGTCGAGATGCACCACGAGCAGCTTCAAGAGG
GCATGCCTGGCGACAACGTCGGCTTCAACGTGAAGAACGTCTCTGTCAAGGAAATCCGCCG
TGGCAACGTCGCTGGTGACTCCAAGAACGATCCTCCTGCTGGTGCTGCTTCCTTCACCGCC
CAGGTCATCGTCCTCAACCACCCTGGTCAGGTTGGTGCTGGCTACGCTCCCGTCCTGGACT
GCCACACTGCCCACATCGCCTGCAAGTTCTCCGAGATCCTCGAGAAGATCGACCGCCGAAC
GGGCAAGTCCGTCGAGAACAACCCCAAGTTCATCAAGTCTGGTGACGC
>Distoseptispora_yunnansis_MFLUCC_20_0153
ACCT--------ACCGTCAA-------TGTTGCTTCGGCGGG-CGGCCCC-TGGGAGGGGTCACGGCCC
ATTGGGTTGGGTGCCCGCCGGAGGA---CGTT-CCCAAACTCGTTTA--TACAAGGTATCTTCTG
AGTAAA------AACCT--AAAA--TAAT-AAAAACTTTCAACAACGGATCTCTTGGTTCTGGCATCGAT
GAAGAACGCAGCGAAATGCGATAAGTAATGTGAATTGCAGAATTCAGTGAATCATCGAATCTT
TGAACGCACATTGCGCCCGCCAGTATTCTGGCGGGCATGCCTGTTCGAGCGTCATTTCA-AC
CCTCAGGCCC-CC-------GT--GGCCTGGTGTTGGGGCACTGCCGTCCCGCCC---------------GTA
CGGGGCGCGCAGGCCCTGAAAAACAGTGGCTGGCTCGCCAGGAC-TCCGGGCGTAGTA---A
ACAT--TCTCGTCTAGGTGGTCT-GGCGCG--GT-CTTGCCGTCAAAAC-CCCCC----------ATATCT
C-------ACAAGCCTTCCGAGTCCCC-TGGAAC------GGGGCGCC-AGAGAGGG-TGAGAGCCCC
GTATGGTTGGACGCCGAACC-TCTGTAAAGCTCCTTCGACGAGTCGAGTAGTTTGGGAATGCT
GCTCAAAATGGGAGGTAAATGTCTTCTAAAG-CTAAATACCGGCCAGAGACCGATAGCGCACA
AGTAGAGTGATCGAAAGATGAAAAGCACTTTGAAAAGAGGGTTAAATAGCACGTGAAATTGCT
GAAAGGGAAGCGCTTGTGACCAGACTTGTGCCCGGTTGATCATCCAGCGTTCTCGCTGGTG
CACTCTGCCGGGCTCAGGCCAGCATCGGTTCTCCCAGGGGGATAAAGGTCGCGGGAACGTA
GCTCTCTCCGGGGAGTGTTATAGCCCGCGG-CGGAATGCCCTTAGGGGGGACCGAGGCCCG

CG-TTCG-CAAGGATGCTGGCGTAATGGTCACCAGCGACCCGTCTTTATCGCTCATGTGTTATG
TCAGCGTTGGAACCCCAGCAGAACCGATCATCGAGTTCATGATCGCGAGGAACATGGAAGTC
CTCGAGGAATACGAGCCCTTGCGCTATCCCAACGCTACCAAGATATTCGTCAACGGGACCTG
GGTAGGCGTTCACCAGGACCCCAAACATCTAGTGGGGCTGGTACAGGAGTTGCGACAGAGA
GGCGTCATCAACTTTGAGGTCTCCCTTGTCCGTGATATCCGAGACAGAGAGTTCAAGATTTTC
TCTGATGCTGGCCGAGTGATGCGGCCCCTTTTCGCCGTGGAGCAGGAGGAC---AACTCTAAG
TCG---GGGCTCCCGAAGGGGGCTCTGAGGCTCACCAAAGAGCACATTCAGAGGCTCAGAGC
AGATGAAGAGTTATCCAAAGATGATCATGAATATTTTGGATGGGACGGCATCGTTGAGGCTGG
TGCAGTTGAATATCTCGACGCGGAAGAAGAGGAAACGGCTATGATTTGCATGACGCCGGAAG
ATCTGGAGGCTTACCGACTCCAAAAAGCCGGATTGGGTATGGCAGAAGACGACGACGAAGC
GG---ATCCGAACAGACGTCTGAAAACGAAATTAAATCCCACCACTCACATGTACACGCACTGT
GAAATCCATCCAAGTATGCTCCTGGGAATCTGTGCAAGCATCATACCACCCGTGAGCACGCC
CTGCTTGCCTACACCCTGGGTGTCAAGCAGATCATCTGTGCCATCAACAAGATGGACACCAC
CAAGTGGTCAGAGGACCGTTTCAACGAAATCATCAAGGAGACGTCCAACTTCATCAAGAAGG
TCGGCTACAACCCCAAGCAGGTCGCCTTCGTCCCTATCTCCGGCTTCCACGGTGACAACATG
TTGACTGCCTCCACCAACTGCCCCTGGTACAAGGGCTGGGAGAAGGAAGGCGCCAAGGGT
GCGAAGTCATCTGGCAAGACCCTGCTTGAGGCCATCGACGCCATCGAGCCCCCCAAGCGTC
CCACCGACAAGCCCCTGCGTCTGCCGCTCCAGGATGTGTACAAGATTGGCGGTATCGGAAC
AGTCCCTGTCGGCCGTATCGAGACCGGTGTCCTCAAGCCCGGCATGGTCGTCACTTTCGCT
CCTTCCAACGTCACCACTGAAGTCAAGTCCGTCGAGATGCACCACGAGCAGCTGACTGAGG
GTGTCCCCGGTGACAACGTCGGCTTCAACGTGAAGAACGTCTCCGTTAAGGAAATCCGCCG
TGGCAACGTCGCTGGTGACTCCAAGAACGACCCTCCTTCTGGCTGCGCCAACTTCACTGCC
CAGGTCATTGTCCTCAACCACCCTGGTCAGGTCGGTCCCGGTTACGCCCCCGTCTTGGATT
GCCACACTGCCCACATCGCCTGCAAGTTCTCCGAGATCACCGAGAAGATCGACCGCCGTAC
CGGCAAGTCGGTTGAGAACAACCCCAAGTTCATCAAGTCTGGAGACGC
>Fluminicola_saprophytica_MFLUCC_15_0976
ACCC--------TT-GTCTT-------AGTTACTTCGGTGGG-CGATCCCTCTGGCTGGGATTGTAGCCGT
GAGGC-----GCCCGCCGGAGGT---AATC--AAAAACTCT----TGTTATAGCAGTCTTCTGAGTACTA
------TAC-----AAATAAAT-CAAAACTTTCAACAACGGATCTCTTGGCTCTAGCATCGATGAAGAAC
GCAGCGAAATGCGATAAGTAATGCGAATTGCAGAATTCCGCGAGTCATCGAATCTTTGAACGC
ACATTGCGCCTGCTAGCATTCTGGCAGGCATGCCTGTCCGAGCGTCATTTCGA-CCCTCAGG
CCTTTA----------TTGCCTGTTGTTGGGGCGCTTCTAGGGGATTAA--------------CCATGATCCGCT
AGAGCCCTGAAAGACAGTGGCGGGCTCGCCAGGTCA-CCGAGCGTAGTAATTCTT-----CTCG
CTTAGGGGGCACTGACGGG--TG-CTAGCCGTGAAAAA-C---CACC------TATAATTCA--------ATG
CCTTCTGAGTTCCC-TGGAAC------GGGACGCC-AAAGAGGG-TGAGAGCCCCGTATAGTCGGA
CACCAAGCC-TCTGTAAAGCTCCTTCGACGAGTCGAGTAGTTTGGGAATGCTGCTCAAAATGG
GAGGTAAATCTCTTCTAAAG-CTAAATACCGGCTAGAGACCGATAGCGCACAAGTAGAGTGATC
GAAAGATGAAAAGCACTTTGAAAAGAGGGTTAAACAGCACGTGAAATTGTTGAAAGGGAAGC
GCTTGTGACCAGACTTGCGCCCGGTTGATCATCCAGCGTTCTCGCTGGTGCACTCTGCCGG
GCTCAGGCCAGCATCGGTTCTCGCGGGGGGATAAAAGCTTCGGGAATGTAGCTCCCTC--GG
GAGTGTTATAGCCCGCTG-CAGAATACCCTCGCG-GGGACCGAGGTTCGCGCTCCG-CAAGG
ATGCTGGCGTAATGGTCATCAGCGACCCGTCTTTATCCCTGATGTGTTACGTCAGTGTAGGCA
CACCGGCTGACCCTATCGTCGAGTTCATGATCGCCAGGAATATGGAGGTACTTGAGGAGTAC
GAGCCTCTGCGATATCCCAACGCAACGAAAGTATTCGTCAACGGCAGTTGGGTCGGGGTGC

ATCAAGATCCAAAGCACTTGGTGGGATTGGTCCAGAACCTGCGACGGCGTAATGTGATCCAG
GCCGAGGTCTCGCTTGTTAGAGACATTCGAGACCGAGAGTTCAAGATCTTCTCTGACGCCGG
GCGTGTGATGCGACCACTCTTCGCCGTAGAACAAGAGGAC---AACCCCGAGAAG---GATCAA
ATTAAGGGGTCTTTGATTCTCAAGAAGCATCATATCCAGCGGCTCGAGGACGACGAGGCTTT
GGATGACACCGATCCAAAGTACTTTGGCTGGGATGGTATTGCATTGACTGGTGCAATCGAGTA
CCTAGATGCCGAGGAAGAAGAGACTGCCATGATTTGCATGACACCCGAGGATCTTGAGAATT
ACCGACTCCAGAAGGCCGGCTACGAAGTCCCTGACGACTATGGTGACGAGGGCGACCTCAA
CAAGCGGTTGAAGACGAAGATGAATCCAACCACACACATGTACACTCATTGTGAGATTCATCC
TAGTATGCTTCTGGGTATCTGCGCAAGCATCATCCCACTCGCGAGCACGCTCTGCTTGCCTAC
ACCCTGGGTGTCAAGCAGCTGATCGTCGCCATCAACAAGATGGACACCACCAAGTGGTCCG
AGGATCGGTACAACGAGATCATCAAGGAGACCTCCAACTTCATCAAGAAGGTCGGCTACAAC
CCCAAGAACGTTGCCTTCGTGCCCATCTCGGGCTTCAACGGTGACAACATGCTGGCTCCCT
CCACCAACTGCCCGTGGTACAAGGGCTGGAAGAAGGAGA---TCAAGGGTGGCGAGGTCACC
GGCAAGACCCTGCTGGAGGCCATTGACGCCATTGAGCCCCCTCGCCGTCCCACCGACAAG
CCACTGCGTCTTCCTCTGCAGGACGTCTACAAGATTGGCGGTATTGGAACTGTTCCCGTCGG
CCGTATCGAGACTGGTATCCTGAAGCCCGGTATGGTCGTCACTTTCGCTCCCTCCAACGTCA
CCACTGAAGTCAAGTCCGTCGAGATGCACCACGAGCAGCTGTCCGAGGGTGTTCCGGGCG
ACAACGTCGGCTTCAACGTCAAGAACGTCTCCGTCAAGGAGATTCGTCGTGGCAACGTTGC
TGGTGACTCCAAGAACGACCCCCCTGCCGGTGCTGCTTCCTTCACCGCCCAGGTTATCGTT
CTGAACCACCCTGGCCAGGTTGGTGCTGGTTACGCTCCCGTTCTGGACTGCCACACCGCCC
ACATCGCCTGCAAGTTCGCCGAGATCACCGAGAAGATTGACCGCCGTACTGGTAAGTCGGTC
GAGAACTCGCCCAAGTTCATCAAGTCCGGTGACGC
>Myrmecridium_banksiae_CBS_132536
ACCC-----------GTCCAA------CGT-GCTTCGGCAGG-CAGCCCTGTGGGACAGAGCCTCGCCCC
CTCCGGGGGGTGCCTGCCGCTGGC---CAAC-CAAAA-CTCCAGCTG--TTTA-GTACCATCTGA
GTCTT------ATACAA-AA-----TAAACAAAACTTTCAACAACGGATCTCTTGGTTCTGGCATCGATG
AAGAACGCAGCGAAATGCGATAAGTAATGTGAATTGCAGAATTCAGTGAATCATCGAATCTTT
GAACGCACATTGCGCCCACTAGTATTCTGGTGGGCATGCCTGTTCGAGCGTCATTTCA-ACCC
TCAAGCCT--------------GGCTTGGTGTTGGGGCTCTGC--------------------------------GTTTGCAGGC
CCTCAAATCCAGTGGCGGACACGCTAGGTC-TCCGAGCGCAGTAGT-TTCTT---CTCGCTTCG
GGCGTCC-GGCGTG--GG-CTTGCC-TTGCACC-CC--------------ATTTTA-------TCAAGCCTTCTGA
GTTCCC-TGGAAC------GGGACGCC-GGAGAGGG-TGAGAGCCCCGTACAGTTGGACACCAAG
CC-TTTGTAAAGCTCCTTCGACGAGTCGAGTAGTTTGGGAATGCTGCTCAAAATGGGAGGTAA
ATGCCTTCTAAAG-CTAAATACCGGCCAGAGACCGATAGCGCACAAGTAGAGTGATCGAAAGA
TGAAAAGCACTTTGAAAAGAGGGTTAAAAAGTACGTGAAATTGTTGAAAGGGAAGGGCTCGT
GACCAGACTTGCGCCCGTCGGATCATCCGGCCTTCTGGCCGGTGCACTCCGGCGGGCTCA
GGCCAGCATCGGTTCTTCTGGGGGGACAAAAGCGGCGGGAACGTAGCTCCTCC--GGGAGT
GTTATAGCCCGCCG-CAGAATACCCCCGGG-GGGACCGAGGACCGCGCTTCGGCAAGGATG
CTGGCGTAATGGTCACCAGCCACCCGTCTT??
??
??
??
??
??

??
??
??
??
??
??
??
??
??
??
??
??
??
???
>Myrmecridium_schulzeri_CBS_100_54
ACCC-----------GTCCAC------CGT-GCTTCGGCAGG-CAGTCCTGTGGGACAGGGCCTCGCCCC
CGCGAGGGGGTGCCTGCCGCTGGC---CAAC-CAAAAATTCTAGCTG--TTTTTGTACCATCTGA
GTCTT------CCACAA-A------TAAACAAAACTTTCAACAACGGATCTCTTGGTTCTGGCATCGATG
AAGAACGCAGCGAAATGCGATAAGTAATGTGAATTGCAGAATTCAGTGAATCATCGAATCTTT
GAACGCACATTGCGCCCACTAGTATTCTGGTGGGCATGCCTGTTCGAGCGTCATTTCA-ACCC
TCAAGCCT--------------GGCTTGGTGTTGGGGCTCTGC--------------------------------GTCTGCAGTC
CCTTAAATCCAGTGGCGGACACGCTAGGTC-TCCGAGCGCAGTAGT-TTCTC---CTCGCTCAG
GGCGTCC-GGCGTG--GG-CTTGCC-TCGCACC-C---------------ATCTTT-------ACAAGCCTTCTGA
GTTCCC-TGGAAC------GGGACGCC-GGAGAGGG-TGAGAGCCCCGTACAGTTGGACACCAAG
CC-TTTGTAAAGCTCCTTCGACGAGTCGAGTAGTTTGGGAATGCTGCTCAAAATGGGAGGTAA
ATGCCTTCTAAAG-CTAAATACCGGCCAGAGACCGATAGCGCACAAGTAGAGTGATCGAAAGA
TGAAAAGCACTTTGAAAAGAGGGTTAAACAGTACGTGAAATTGTTGAAAGGGAAGCGCTCGT
GACCAGACTTGCGCCCGGCGGATCATCCGGCCTTCTGGCCGGTGCACTCCGCCGGGCTCA
GGCCAGCATCGGTTTTCCTGGGGGGATAAAAGTGGCGGGAACGTAGCTCCTCC--GGGAGTG
TTATAGCCCGCCG-CAGCATACCCCCGGG-GGGACCGAGGACCGCGCTTCGGCAAGGATGCT
GGCGTAATGGTCACCAGCGACCCGTCTT???
??
??
??
??
??
??
??
??
??
??
??
??
??

???
???
???
???
???
???
>Papulosa_amerospora_AFTOL_ID_748
???
???
???
???
???
???
???
????????GCCTTCTGAGTTCCC-TGGAAC------GGGACGCC-ATAGAGGG-TGAGAGCCCCGTAT
AGTCGGACACCAAGCC-TCTGTAAAGCTCCTTCGACGAGTCGAGTAGTTTGGGAATGCTGCT
CAAAATGGGAGGTAAATCTCTTCTAAAG-CTAAATACCGGCTAGAGACCGATAGCGCACAAGTA
GAGTGATCGAAAGATGAAAAGCACTTTGAAAAGAGGGTTAAACAGCACGTGAAATTGTTGAA
AGGGAAGCGCTTGTGACCAGACTTGTGCCCGGTGAATCATCCAGCGTTCTCGCTGGTGCAC
TTTGCCGGGTTCAGGCCAGCATCGGTTCTCGCAGGGGGATAAAAGCCTTGGGAATGTAGCT
CCCTC--GGGAGTGTTATAGCCCGTTG-CATAATACCCTTGTG-GGGACCGAGGTTCGCGCTCC
G-CAAGGATGCTGGCGTAATGGTCATCAGCGACCCGTCTTTATCCCTAATGTGTTACGTCAGC
GTCGGCACTCCAGCAGAGCCCATCGTGGAGTTCATGATCGCGAGAAATATGGAAGTCCTCGA
GGAGTACGAACCTCTGCGATATCCGAATGCAACCAAGGTCTTCGTCAATGGCAGTTGGGTAG
GTGTGCACCAAGACCCGAAGCATTTGGTCGGGTTGGTTCAGAATCTCCGGCGGAGAGGCGT
CATTCAGGGCGAGGTCTCGCTTGTTAGAGACATTCGAGACCGAGAGTTCAAGATCTTNTCCG
ATGCCGGACGGGTGATGCGACCATTGTTTGCGGTTGAGCAAGAGGAC---AATGCAGAGACT--
-GGACAAGTCAAGGGTTCTCTGATCCTTACTAAGCAGCATGTCAAGCGACTGGAGGCCGATG
AGTCTCTGGATGACACGCATCAAGACTATTTCGGCTGGGACGGTGTTTGCGAGGCTGGAGC
CATAGAGTATTTGGATGCTGAGGAGGAGGAGACCGCAATGATTTGCATGACGCCAGAAGACT
TGGAGAATTACCGCCTGCAGAAGGCGGGCCACGAGATTCTCGAGGACTATGGCGAGGAGG--
-ATCTCAACAAGCGCCTAAAGACGAAGCTGAATCCTACTACCCACATGTATACTCACTGCGAGA
TCCATCCCAGCATGCTCTTGGGTATTTGTGCCAGCATCATCCCACTCGCGAGCACGCTCTGC
TTGCCTACACCCTGGGTGTCAAGCAGCTGATCGTCGCCATCAACAAGATGGACACCACCAAG
TGGTCCGAGGATCGGTACAACGAGATCATCAAGGAGACCTCCAACTTTATCAAGAAGGTCGG
CTATAACCCCAAGCAGGTTGCCTTCGTTCCCATTTCGGGTTTCAACGGCGACAACATGCTGG
CCCCTTCCACCAACTGCCCCTGGTACAAGGGCTGGAAGAAGGAGG---GCAAGGGTGGTGAT
GTCACCGGCAAGACTCTCCTCGAGGCTATTGATGCCGTTGAGCCTCCCAAGCGTCCCACGG
ACAAGCCTCTGCGCCTTCCCCTACAGGATGTCTACAAGATTGGTGGTATCGGAACTGTCCCT
GTCGGCCGTATCGAGACTGGTGTTCTCAAGCCCGGTATGGTCGTCACCTTCGCTCCTTCCAA
CGTCACCACTGAAGTCAAATCCGTCGAAATGCACCACGAGCAGCTTGCTGAGGGTGTTCCG
GGCGACAACGTCGGCTTCAACGTGAAGAACGTTTCCGTCAAGGAAATCCGTCGTGGCAATG
TTGCTGGTGACTCCAAGAACGACCCCCCTGCTGGTGCTGCTTCCTTCACCGCTCAGGTCAT
CGTCCTCAACCACCCCGGTCAGGTCGGTGCTGGCTACGCCCCCGTCTTGGATTGCCATACT

GCCCACATCGCTTGCAAGTTCGCCGAGATCACCGAGAAGATCGATCGCCGAACTGGTAAGTC
GGTTGAGAACGCCCCCAAGTTCATCAAGTCTGGNGACGC
>Pleurophragmium_bambusinum_MFLUCC_12_0850
AACC-ACTGTGAATCATACCAAGCCGTTGCCTCTTCGGCGGGCGGTCCCCTGGGAGGGGAC
CATTGTCTCTTCGCTGAGGCGCCCGCCGGGG----GCAGA--ATAAACTCTCGTATCTCT-AGTG
GATCTCTGAGCACTTTTT----------AAAAAATAATCACAACTTTCAACAACGGATCTCTTGGCTCT
GGCATCGATGAAGAACGCAGCGAAATGCGATACGTAATGCGAATTGCAGAATTCAGCGAGTC
ATCGAATCTTTGAACGCACATTGCGCCCGCTAGCACTCTAGCGGGCACGCCTGTCCGAGCGT
CATTTCAACCCCTCAGGCCTCCCTTG-----CGTTGCCTGGCGTTGGGGCCCTGCGTCGC--------
-----------------GAGTCGCAGGCCCTGAAAATCAGTGGCGGGCCTACCTGGAAGCCCTGGCGTA
GTAGTTT----TCTCTCGCCTGGGGAGCCACAGGAGGTCCTTCCTGCCGTAAAACCCCTCTACA
A-GCCTGCGAACTCTCGCAGCAGAA-GCCTACCAAGTCCAC-TGGAAC------GTGGCGCC-ACA
GAGGG-TGAGAGCCCCGTACTGTAGGACCCCGAGCC-TCTGTGAAGCTCCTCCGACGAGTC
GAGTAGTTTGGGAATGCTGCTCTAAATGGGAGGTAAATTTCTCCTAAAG-CTAAATACCGGCCA
GAGACCGATAGCGCACAAGTAGAGTGATCGAAAGATGAAAAGCACTTTGAAAAGAGGGTTAA
ACAGCACGTGAAATTGTTGAAAGGGAAGCGCCTGTGACCAGACTCGTGCCCGGCGGATCAT
CCAGCCTTCTGGCTGGTGCACTGCGCCGGGTCCGGGCCAGCATCGGCTCTCCCTGGGGGA
TAAAGGCCCTGGGAACGTAGCTCTCTCCGGGGAGTGTTATAGCCCATCG-CACAATGCC-TCA
GGGGGGGCCGAGGACCGCGCTTCGGCAAGGATGCTGGCGTAATGGTCATCAGCGACCCGT
CTT??
??
??
??
??
??
??
??
???AC
GCGCGAGCACGCTCTGCTCGCCTACACCCTCGGTGTGCGGCAGATCATTGTCGCCATCAAC
AAGATGGACACCACCAAGTGGTCCGAGGATCGTTACAACGAAATCATCAAGGAGACGTCCAA
CTTCATCAAGAAGGTCGGCTTCAACCCCAAGCAGATCGCTTTTGTGCCCATCTCCGGCTTCA
ACGGCGACAACATGCTTACGGCCTCCACCAACTGCCCCTGGTACAAGGGGTGGGAGAAGGA
GG---TCAAGGGTGGCAAGGTCACCGGCAAGACTCTCCTCGAGGCCATCGATGCCATCGAGC
CTCCCCGCCGTCCCACTGACAAGCCCCTCCGTCTGCCGCTTCAGGATGTCTACAAGATCGG
TGGTATCGGGACTGTACCGGTCGGCCGTATCGAGACCGGTATTCTCAAGCCCGGTATGGTCG
TGACCTTCGCTCCTTCCAACGTCACCACTGAAGTCAAGTCCGTCGAGATGCACCACGAACAG
CTTGCTGAGGGTCTCCCGGGCGACAACGTCGGCTTCAACGTGAAGAACGTGTCGGTCAAG
GAAATCCGCCGTGGCAACGTCGCCGGTGACTCCAAGAACGACCCGCCCGCTGGCGCCGCC
TCCTTCACCGCCCAGGTTATCGTTCTGAACCACCCCGGCCAGGTTGGTGCTGGATACGCCC
CAGTGCTGGACTGCCACACGGCCCACATTGCCTGCAAGTTCGCCGAGATCACGGAGAAGAT
CGACCGCCGAACTGGTAAGTCGGTCGAGAACAACCCTAAGTTCATCAAGTCTGGTGACGC
>Pseudostanjehughesia_aquitropica_MFLUCC_16_0569
ACCT--------GT-TTC----------GTTGCTTTGGCAGG-CGGCCCC-AGGGCGGGGCCG-TGGCCGTC
AGGT-----GCCTGCCAGAGGA---T--T---AGAACTCTT--GTTTTTGGTGGTC-TTCAGAGTATC-------

-AGAC-A-AA--TTAGTCAAAACTTTCAACAACGGATCTCTTGGCTCTAGCATCGATGAAGAACGC
AGCGAAATGCGATAAGTAATGCGAATTGCAGAATTACGTGAGTCATCGAATCTTTGAACGCAC
ATTGCGCCTGCCGGTACTCCGGCAGGCATGCCTGTTCGAGCGTCATTTCA-ACCCTCAGGCC
CTGG---------CTTGCCTGGTGTTGGGGCG---CTAC------------------------GGTCCGCCGTAGCCCC
TGAAAGACAGTGGCGGGCTTCCCGGAAA-CCCTAGCGCAGTAGT-----GTATCTCGCTCGGGG
CGTTTTGGGGGG--TG-CTGGCCGTTAAACC-ACCC----------T--ATATT-------CTAAGCCTTCTAAG
TTCCC-TGGAAC------GGGACGCC-GGAGAGGG-TGAGAGCCCCGTATAGTCGGCAGCCAAGC
C-TCTGTAAAGCTCCTTCGACGAGTCGAGTAGTTTGGGAATGCTGCTCAAAATGGGAGGTAAA
TTTCTTCTAAAG-CTAAATACCGGCTAGAGACCGATAGCGCACAAGTAGAGTGATCGAAAGATG
AAAAGCACTTTGAAAAGAGGGTTAAACAGCACGTGAAATTGTTGAAAGGGAAGCGCTTGTGA
CCAGACTTGCGCCCCGCGGATCACCCGTCGTTCTCGCCGGGGCACTCCACGGGGCTCAGG
CCAGCATCGGTTTCGCCGGGGGGATAAAAGTTTAGGGAACGTGGCTCCCCC--GGGAGTGTT
ATAGCCCTCTA-CAGAATACCCCCAGC-GGGACCGAGGTTCGCGCTCTG-CAAGGATGCTGGC
GTAATGGTCATCAGCGACCCGTCTT???
??
??
??
??
??
??
??
??
??????????????????????????ACTCGCGAGCACGCTCTGCTGGCCTTCACCCTCGGTGTGC
GGCAGCTGATTGTCGCCATCAACAAGATGGACACCACCAAGTGGTCCGAGGATCGGTACAA
CGAAATCATCAAGGAGACTTCCAACTTCATCAAGAAGGTCGGCTACAATCCCAAGCAGGTTG
CCTTCGTCCCCATCTCGGGCTTCAACGGCGACAACATGCTTACGCCTTCTACCAACTGCCCC
TGGTACAAGGGCTGGAAGAAGGAAA---CCAAGGGTGGTGAGGTCACTGGCAAGACCCTGCT
TGAGGCTATTGATGCCGTCGAGCCTCCCAAGCGTCCCACCGACAAGCCCCTCCGCCTCCCC
CTCCAGGATGTCTACAAGATCGGTGGTATCGGAACCGTTCCCGTTGGCCGTATCGAGACCGG
TGTCCTCAAGCCCGGTATGGTCGTCACTTTCGCTCCGTCCAACGTCACCACTGAAGTCAAGT
CCGTCGAGATGCACCACGAGCAGCTTACTGAGGGTGTTCCCGGTGACAACGTCGGCTTTAA
CGTGAAGAACGTTTCCGTCAAGGAAATCCGTCGTGGAAACGTTGCTGGTGACTCCAAGAAC
GACCCCCCTGCCGGTGCTGCGTCGTTCACCGCCCAAGTCATCGTCCTCAACCACCCTGGTC
AAGTTGGTGCCGGCTACGCCCCCGTCTTGGATTGCCACACCGCCCACATCGCCTGCAAGTT
CGCCGAGATCACCGAGAAGATTGACCGCCGAACTGGCAAGTCAGTTGAGGCCAACCCCAAG
TTCATCAAGTCCGGTGACGC
>Pseudostanjehughesia_lignicola_MFLUCC_15_0352
ACCT--------GT-ATC----------GTTGCTTTGGCAGG-CGGCCCC-AGGGCGGGGCCG-CGGCCGC
AAGGC-----GCCTGCCGAGGGG---C--T---AGAACTCTT--GCCTTTAGCGGTC-T-CTGAGTACT----
----ATA--A-AA--TAAGTTAAAACTTTCAACAACGGATCTCTTGGCTCTAGCATCGATGAAGAACG
CAGCGAAATGCGATAAGTAATGCGAATTGCAGAATTCCGCGAGTCATCGAATCTTTGAACGCA
CATTGCGCCTGCTGGTACTCCGGCAGGCATGCCTGTTCGAGCGTCATTTCGCACCCTCAGG
CTCTAG-----------TGCCTGGTGTTGGGGAG---CTAC------------------------GGTCCGCCGTAGGCC
CTGAAAGACAGTGGCGGGCTCTCCAAGAA-GCCGGGC-TAGTAA------ATGCCT-GCTCGG--CG

TACGGGAGGG--TG-CCGGCCGTTAAACC-TTCC----------GCAAGGAC-------CACTGCCTTCTGA
GTTCCC-TGGAAC------GGGACGCC-GGAGAGGG-TGAGAGCCCCGTATAGTCGGCAGCCAAG
CC-AGTGTAAAGCTCCTTCGACGAGTCGAGTAGTTTGGGAATGCTGCTCAAAATGGGAGGTAA
ATTTCTTCTAAAG-CTAAATACCGGCTAGAGACCGATAGCGCACAAGTAGAGTGATCGAAAGAT
GAAAAGCACTTTGAAAAGAGGGTTAAACAGCACGTGAAATTGTTGAAAGGGAAGCGCTCGTG
ACCAGACTTGCGCCC-GCGGCTCTCCCGGTGTTCTCGCCGGAGTATTCCGCCGGGCTCAGG
CCAGCATCGGTTTCGACGGGGCGATAAAAGCTTGGGGAACGTAGCTCCTCC--GGGAGTGTT
ATAGCCCTCTG-CAAAATAGCCCTGAC-GGGACCGAGGTTCGCGCTCTG-CAAGGATGCTGGC
GTAATGGTCACCAGCGACCCGTCTTTGTCTCTCATGTGTTATGTGAGCGTCGGCACACCGGC
AGAGCCAATCAACGAGTTCATGATTGCTAGGAACATGGAGGTTCTCGAAGAGTACGAGCCGT
TGCGATACCCGAACGCGACAAAAGTGTTCATAAACGGCACGTGGGTGGGTGTACATCAGGAC
CCGAAGCATCTCGTCACGCTTGTTCAGGGTCTCCGGCGAAAAGGTATCATTCAAGGCGAGGT
TTCTCTTGTTAGAGACATTCGAGACCGCGAATTCAAGATCTTTTCCGACGCCGGACGCGTGAT
GCGGCCCCTGTTTGCTGTCGAGCAGGAGGAC---AATGCAGAGACG---GGTCAATCAAAGGGT
TCCCTAATCCTGACGAAGGCGCACATCAAGCGACTGGAGGCTGACGAGACCTTGGACAACA
CCCATGAAGACTACTTCGGCTGGGATGGTGTCTGCGAAGCTGGCGCTATCGAATATCTCGAT
GCGGA--
--ACTCGCGAGCACGCTCT
GCTTGCCTTCACCCTTGGTGTGCGGCAGCTGATCGTCGCCATCAACAAGATGGACACCGCC
AAGTGGGCTGAGGATCGGTACAACGAAATTATCAAGGAGACTTCCAACTTCATCAAGAAGGT
CGGCTACAACCCCAAGCAGGTTGCCTTCGTCCCCATCTCGGGCTTCAACGGCGACAACATG
CTTGCGGCCTCATCCAACTGCCCCTGGTACAAGGGGTGGAAGAAGGAGA---CCAAGGGTGG
TGAGGTCACTGGCAAGACCCTGCTTGAGGCCATCGATGCCGTTGAGCCTCCCAAGCGTCCC
ACCGACAAGCCCCTCCGTCTTCCCCTTCAGGATGTCTACAAGATCGGCGGTATTGGGACAGT
TCCCGTCGGCCGTATCGAGACTGGTATCCTCAAGCCCGGTATGGTCGTTACTTTCGCTCCCA
CCATGGTCACCACTGAAGTCAAGTCCGTCGAGATGCACCACGAGCAGCTTACTGAGGGTGT
CCCCGGCGACAACGTCGGCTTCAACGTGAAGAACGTTTCCGTCAAGGAAATCCGTCGTGGA
AACGTTGCCGGTGACTCCAAGAACGACCCCCCGGCTGGCGCTGCCTCGTTCACCGCTCAG
GTCATCGTCCTCAACCACCCCGGCCAAGTCGGTGCTGGCTATGCCCCCGTCTTGGACTGCC
ACACTGCCCACATCGCCTGCAAGTTCTCCGAGATCCAGGAGAAGATCGATCGCCGAACCGG
CAAGTCGGTTGAGTCCAACCCCAAGTTCATCAAGTCCGGTGACGC
>Wongia_griffinii_DAR_80512
ACC-------------TATT-------CGTTGCTTCGGTGGGGTGGCCCCTCCGGGGGTCCTCTGGGCCGC
AAGGC-----GCCCGCCGGAGGT---TATTTTAAACACTCTG---TCTTCTATTGTACCTCTGAATAAA
AAA---ATAA-----AAAACAAT-CAAAACTTTCAACAACGGATCTCTTGGCTCTAGCATCGATGAAG
AACGCAGCGAAATGCGATAAGTAATGCGAATTGCAGAATTCCGCGAGTCATCGAATCTTTGAA
CGCACATTGCGCCTGCCAGTATTCTGGCAGGCATGCCTGTCCGAGCGTCATTTCGA-CCCCC
AGGCCTTCG----------TTGCCTGGTGTTGGGGCA-TTCAGGGTGGTC----------------CGACGGACC
CCCTGAGCCCTGAAAATCAGTGGCGGGCCTGCCAGGTCA-CCGAGCGCAGTAATCTCT-----C
TCGCTCAGGGCCC-CTGGCGGG--TG-CTAGCCGTGAAAAA-CACACACC------TACGAA-CA-----
---GTGCCTTCTGAGTTCCC-TGGAAC------GGGACGCC-ACAGAGGG-TGAGAGCCCCGTATAG
TCGGACACCAAGCC-TCTGTAAAGCTCCTTCGACGAGTCGAGTAGTTTGGGAATGCTGCTCA
AAATGGGAGGTAAATCTCTTCTAAAG-CTAAATACAGGCTAGAGACCGATAGCGCACAAGTAGA
GTGATCGAAAGATGAAAAGCACTTTGAAAAGAGGGTTAAAAAGCACGTGAAATTGTTGAAAG

GGAAGCGCTCGTGACCAGACTTGCGCCCGGTTGATCATCCAGCGTTCTCGCTGGTGCACTC
TGCCGGGCTCAGGCCAGCATCGGTTCTTGCGGGGGGATAAAAGCTGTGGGAATGTAGCTCC
CTC--GGGAGTGTTATAGCCCGCTG-CAGAATACCCCCGCG-GGGACCGAGGTACGCGTTCCG
-CAAGGATGCTGGCGTAATGGTCACCAGCGACCCGTCTT?????????????????????????????
???
???
???
???
???
???
???
???
???
???
???
???
???
???
???
???
???
???
??
